# Supplementary material for: Nickel-catalyzed reductive thiolation and selenylation of unactivated alkyl bromides
Source: Nat Commun. 2018 Jun 8;9:2240. doi: 10.1038/s41467-018-04646-2 (PMC5993785; doi:10.1038/s41467-018-04646-2)
Supplement: Supplementary file 1 — Supplementary Information [file 41467_2018_4646_MOESM1_ESM.pdf]

Nickel-catalyzed reductive thiolation and selenylation of unactivated alkyl bromides

Fang *et al.*

Supplementary Methods

Unless otherwise noted, all commercially available compounds were used as provided without further purification. Solvents for chromatography were analytical grade and used without further purification. Anhydrous MeCN, DMF, DMA, DMSO, NMP and THF (99.9%, Extra Dry with molecular sieves, Water≤50 ppm, in resealable bottle under Ar) were purchased from Adamas-beta®. Alkyl-selenosulfonates were purchased from Suzhou Chukai PharmaTech Co., Ltd. Analytical thin-layer chromatography (TLC) was performed on silica gel, visualized by I_2 or irradiation with UV light. For column chromatography, 300-400 mesh silica gel was used. Flash chromatography was performed with SepaBean® machine of Santai Technologies. ^{1}H-NMR and ^{13}C-NMR were recorded on a BRUKER 300 MHz or 400 MHz spectrometer or Varian Inova-600 at 600 MHz. Chemical shifts (δ) were reported referenced to an internal tetramethylsilane standard or the $CDCl_3$ residual peak (δ 7.26) for ^{1}H NMR. Chemical shifts of ^{13}C NMR were reported relative to $CDCl_3$ (δ 77.16). Data were reported in the following order: chemical shift (δ) in ppm; multiplicities were indicated s (singlet), bs (broad singlet), d (doublet), t (triplet), m (multiplet); coupling constants (J) were in Hertz (Hz). Melting points were measured on an Electrothermal digital melting point apparatus and were uncorrected. IR spectra were recorded on a BRUKER VERTEX 70 spectrophotometer and were reported in terms of frequency of absorption (cm^{-1}). *In situ* IR spectroscopy was performed on Mettler-Toledo ReactIR 15 equipped with a diamond ATR probe and a MCT detector. Spectra were acquired using Mettler-Toledo iC IR software version 7.0.297 in the range of 650–2200 cm^{-1} with a 4 cm^{-1} resolution. HRMS spectra were obtained by using GCT Premier TOF-MS with CI source or BRUKER microTOF-Q III instrument with ESI source.

Supplementary Table 1. Optimization of Nickel-catalysts for the synthesis of 3a.

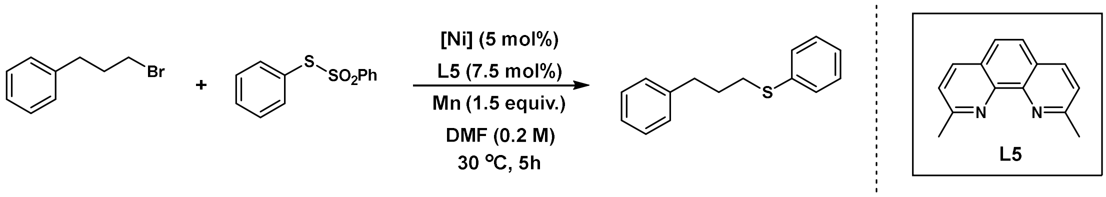

Entry	[Ni] (5 mol%)	GC-Yield (%)
1	$NiCl_2 \cdot DME$	98
2	$NiBr_2 \cdot DME$	97
3	$NiCl_2$	N.D.
4	$NiBr_2$	98
5	NiF_2	N.D.
6	$Ni(OAc)_2$	trace
7	$Ni(acac)_2$	89
8	$NiCl_2(PCy_3)_2$	98
9	$NiCl_2(PPh_3)_2$	99

10	$NiCl_2$(dppp)	81
11	$NiCl_2$(dppf)	98
12	$Ni(cod)_2$	98

Supplementary Table 2. Optimization of ligands for the synthesis of 3a.

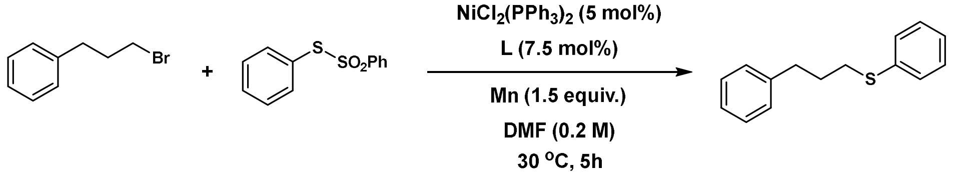

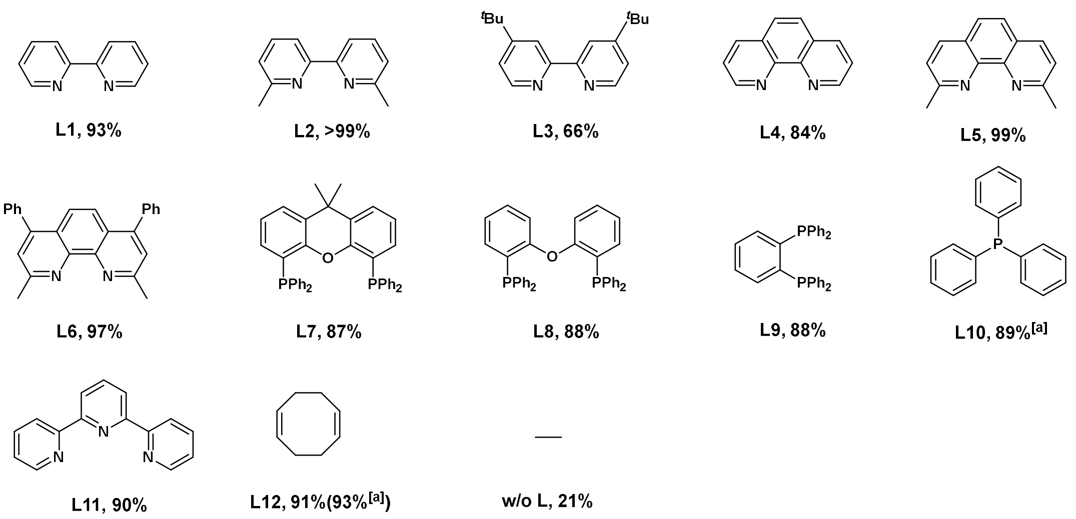

[a] 15 mmol% ligand was used.

Supplementary Table 3. Optimization of solvents for the synthesis of 3a.

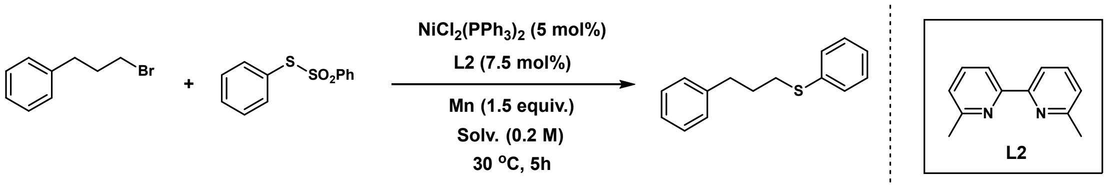

Entry	Solv. (1 mL)	GC-Yield (%)
1	DMF	>99
2	DMA	92
3	NMP	96
4	DMSO	45
5	THF	N.D.
6	MeCN	N.D.

Supplementary Table 4. Optimization of reducers for the synthesis of 3a.

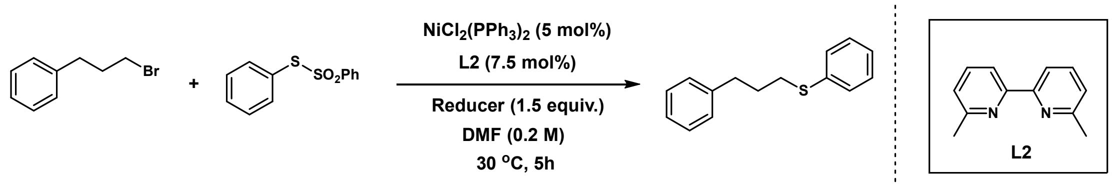

Entry	Reducer	GC-Yield (%)
1	Mn	>99
2	Zn	21

Supplementary Table 5. Optimization of Nickel-catalysts for the synthesis of 5a.

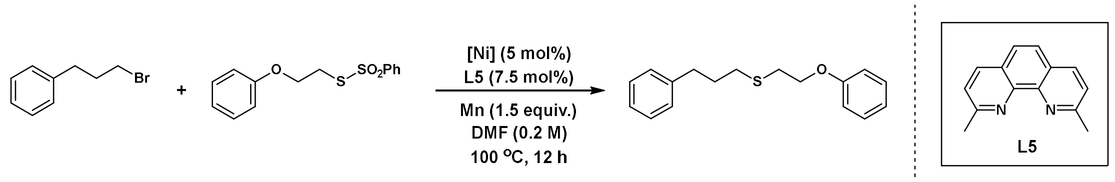

Entry	[Ni] (5 mol%)	GC-Yield (%)
1	$NiCl_2 \cdot DME$	74
2	$NiBr_2 \cdot DME$	72
3	$NiCl_2$	67
4	$NiBr_2$	81
5	NiF_2	32
6	$Ni(OAc)_2$	51
7	$Ni(acac)_2$	61
8	$Ni(cod)_2$	56

Supplementary Table 6. Optimization of ligands for the synthesis of 5a.

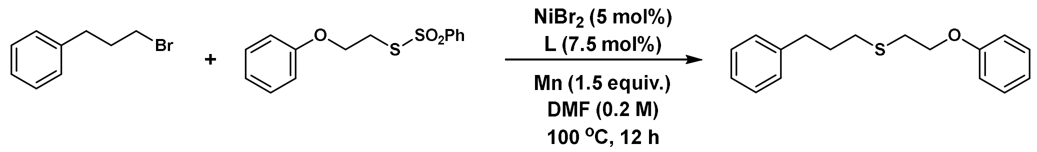

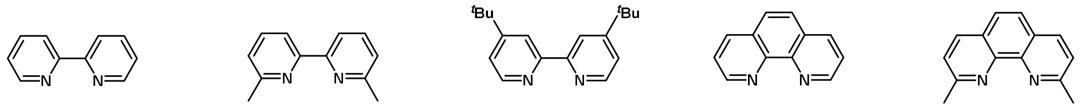

L1, 65% | L2, 78% | L3, 71% | L4, 65% | L5, 81%

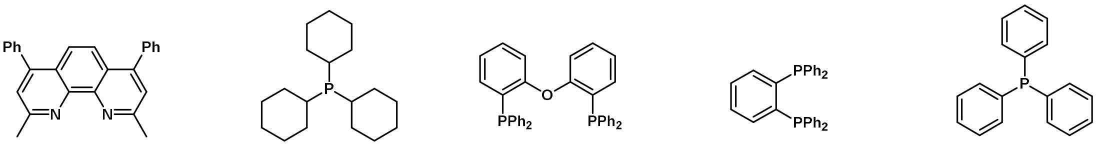

L6, 69% | L7, 44%[a] | L8, 25% | L9, 37% | L10, 32%[a]

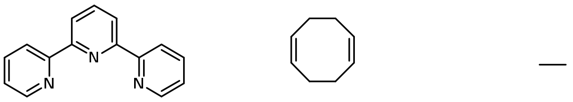

L11, 71% | L12, 35%[a] | w/o L, 13%

[a] 15 mmol% ligand was used.

Supplementary Table 7. Optimization of solvents for the synthesis of 5a.

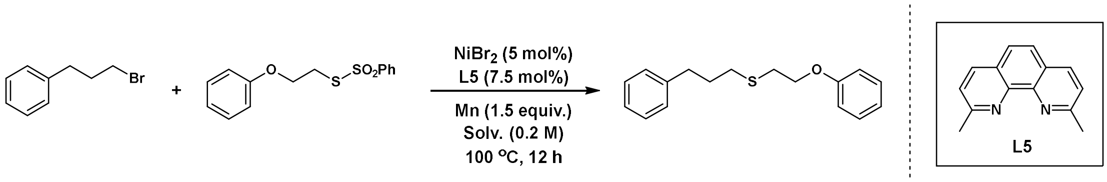

Entry	Solv. (1 mL)	GC-Yield (%)
1	DMF	81
2	DMA	68
3	NMP	58
4	DMSO	35
5	THF	N.D.
6	Toluene	N.D.
7	MeCN	60
8	DMF-MeCN (4:1)	76
9	DMF-MeCN (3:2)	87
10	DMF-MeCN (1:1)	98
11	DMF-MeCN (2:3)	>99
12	DMF-MeCN (1:4)	92
13	DMF-MeCN (3:7)	93

Supplementary Table 8. Optimization of temperature for the synthesis of 5a.

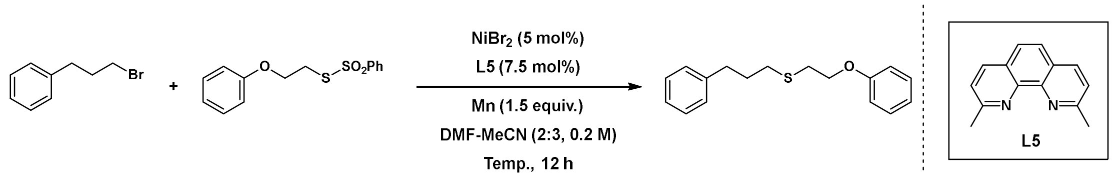

Entry	Temp. (ºC)	GC-Yield (%)
1	100	>99
2	90	93
3	80	90
4	70	80

Supplementary Table 9. Optimization of reducers for the synthesis of 5a.

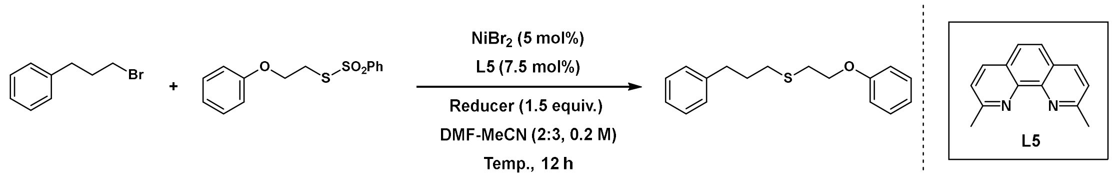

Entry	Reducer	GC-Yield (%)
1	Mn	>99
2	Zn	83

Procedure for the preparation of $PhSO_2SNa$.[1] Sodium benzenesulfinate (10 g, 61 mmol) and sulfur (1.95 g, 61 mmol) were dissolved in anhydrous pyridine (60 mL) to give a yellow solution. The reaction was stirred under argon and after 1 h gave a white suspension. Et_2O was added to the suspension, and the reaction was filtered and washed with anhydrous diethyl ether. Recrystallization from anhydrous ethanol afforded $PhSO_2SNa$ (10.5 g, 88%) as a white crystalline solid.

General procedure for the preparation of $PhSO_2SAr$.[2] A mixture of $PhSO_2Na$ (4 equiv), disulphide (1 equiv) and NBS (2 equiv) in MeCN was stirred at room temperature. After the completion of the reaction, as monitored by TLC, the reaction mixture was washed with water and extracted with ethyl acetate. The organic phase was separated and dried over anhydrous Na_2SO_4 and filtered. The filtrate was concentrated and the resulting residue was purified by column chromatography to provide the desired aryl-thiosulfonates.

General procedure for the preparation of $PhSO_2SAlkyl$.[3] To a solution of $PhSO_2SNa$ (1 equiv) in DMF was added Alkyl bromide (2 equiv) and the reaction mixture was stirred at room temperature. After the completion of the reaction, as monitored by TLC, the reaction mixture was diluted with ethyl acetate and washed with

water. The organic phase was separated and dried over anhydrous Na_2SO_4 and filtered. The filtrate was concentrated and the resulting residue was purified by column chromatography to provide the desired alkyl-thiosulfonates.

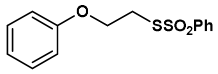

***S*-(2-phenoxyethyl) benzenesulfonothioate (4a)**. According to GPB, isolated as a white solid in 84% yield. **^{1}H NMR** (400 MHz, $CDCl_3$) δ 7.95 (dd, *J* = 7.8, 1.6 Hz, 2H), 7.68 – 7.59 (m, 1H), 7.55 (dd, *J* = 8.4, 7.0 Hz, 2H), 7.30 – 7.21 (m, 2H), 6.96 (t, *J* = 7.4 Hz, 1H), 6.80 (d, *J* = 7.7 Hz, 2H), 4.16 (t, *J* = 6.4 Hz, 2H), 3.37 (t, *J* = 6.4 Hz, 2H).

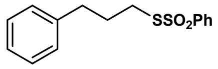

***S*-(3-phenylpropyl) benzenesulfonothioate (4b)**. According to GPB, isolated as a pale yellow in 86% yield. **^{1}H NMR** (400 MHz, $CDCl_3$) δ 7.92 – 7.84 (m, 2H), 7.62 (t, *J* = 7.4 Hz, 1H), 7.52 (t, *J* = 7.7 Hz, 2H), 7.28 – 7.14 (m, 3H), 7.10 – 7.03 (m, 2H), 2.97 (t, *J* = 7.3 Hz, 2H), 2.62 (t, *J* = 7.4 Hz, 2H), 1.94 (p, *J* = 7.4 Hz, 2H).

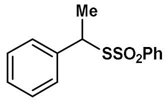

***S*-(1-phenylethyl) benzenesulfonothioate (4c)**. $PhSO_2SNa$ (1.0 equiv) and (1-bromoethyl)benzene (1.5 equiv) were mixed in DMF, stirred at rt for 12h, isolated as a pale yellow oil in 83% yield. **^{1}H NMR** (400 MHz, $CDCl_3$) δ 7.68 (dd, *J* = 8.3, 1.3 Hz, 2H), 7.55 – 7.44 (m, 1H), 7.37 (t, *J* = 7.7 Hz, 2H), 7.20 – 7.09 (m, 5H), 4.64 (q, *J* = 7.2 Hz, 1H), 1.66 (d, *J* = 7.2 Hz, 3H).

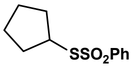

***S*-cyclopentyl benzenesulfonothioate (4d)**. $PhSO_2SNa$ (1 equiv), bromocyclopentane (1.2 equiv) and TBAI (5 mol%) were mixed in DMF, stirred at 80 °C for 10h, isolated as a pale yellow oil in 54% yield. **^{1}H NMR** (400 MHz, $CDCl_3$) δ 7.95 (dd, *J* = 7.7, 1.7 Hz, 2H), 7.68 – 7.59 (m, 1H), 7.56 (dd, *J* = 8.3, 6.9 Hz, 2H), 3.59 (p, *J* = 6.9, 1H)2.02 (tdt, *J* = 8.2, 5.9, 3.6 Hz, 2H), 1.72 – 1.47 (m, 6H).

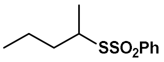

***S*-(pentan-2-yl) benzenesulfonothioate (4e)**. $PhSO_2SNa$ (1 equiv), 2-bromopentane (1.2 equiv) and TEBAC (1 equiv) were mixed in DMF, stirred at 80 °C for 12h, isolated as a pale yellow oil in 42% yield. **^{1}H NMR** (400 MHz, $CDCl_3$) δ 7.99 (dd, *J* = 8.3, 1.4 Hz, 2H), 7.72 – 7.63 (m, 1H), 7.59 (dd, *J* = 8.3, 7.0 Hz, 2H), 3.40 (h, *J* = 6.9 Hz, 1H), 1.61 – 1.50 (m, 2H), 1.41 – 1.24 (m, 5H), 0.83 (t, *J* = 7.3 Hz, 3H).

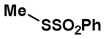

***S*-methyl benzenesulfonothioate (4f)**. According to GPB, isolated as a yellow oil in 90% yield. **^{1}H NMR** (400 MHz, $CDCl_3$) δ 7.93 (dd, J = 8.4, 1.3 Hz, 2H), 7.70 – 7.61 (m, 1H), 7.57 (dd, J = 8.3, 6.9 Hz, 2H), 2.51 (s, 3H).

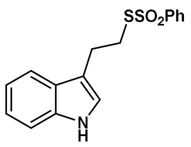

***S*-(2-(1H-indol-3-yl)ethyl) benzenesulfonothioate (4g)**. $PhSO_2SNa$ (1.2 equiv) and 3-(2-bromoethyl)-1H-indole (1.0 equiv) were mixed in DMF, stirred at rt for 12h, isolated as a brown oil in 77% yield. **^{1}H NMR** (400 MHz, $CDCl_3$) δ 8.15 (s, 1H), 7.95 – 7.88 (m, 2H), 7.58 (t, *J* = 7.4 Hz, 1H), 7.53 – 7.44 (m, 3H), 7.32 (d, *J* = 8.1 Hz, 1H), 7.17 (t, *J* = 7.3 Hz, 1H), 7.08 (t, *J* = 7.4 Hz, 1H), 6.90 (d, *J* = 2.3 Hz, 1H), 3.29 (t, *J* = 7.4 Hz, 2H), 3.06 (t, *J* = 7.5 Hz, 2H).

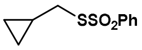

***S*-(cyclopropylmethyl) benzenesulfonothioate (4h)**. $PhSO_2SNa$ (1.0 equiv) and (bromomethyl)cyclopropane (1.5 equiv) were mixed in DMF, stirred at rt for 12h, isolated as a pale yellow oil in 86% yield. **^{1}H NMR** (400 MHz, $CDCl_3$) δ 8.15 (s, 1H), 7.95 – 7.88 (m, 2H), 7.58 (t, *J* = 7.4 Hz, 1H), 7.53 – 7.44 (m, 3H), 7.32 (d, *J* = 8.1 Hz, 1H), 7.17 (t, *J* = 7.3 Hz, 1H), 7.08 (t, *J* = 7.4 Hz, 1H), 6.90 (d, *J* = 2.3 Hz, 1H), 3.29 (t, *J* = 7.4 Hz, 2H), 3.06 (t, *J* = 7.5 Hz, 2H).

General procedure A for the reductive aryl-thiolation of unactivated bromides with $PhSO_2SAr$. In glovebox**,** an oven-dried screw-capped 8-mL vial equipped with a magnetic stir bar was charged with $NiCl_2(PPh_3)_2$ (16.4 mg, 0.025 mmol), 6,6'-Dimethyl-2,2'-dipyridyl (**L2**, 6.9 mg, 0.0375 mmol) and Mn powder (41.2 mg, 0.75 mmol). DMF (1 mL) was added via syringe and the mixture was stirred at room temperature for 10 min. The alkyl bromide **1** (0.5 mmol) was added, followed by the addition of aryl-thiosulfonate **2** (0.55 mmol) in one portion. Additional DMF (1.5 mL) was subsequently added via syringe. The resulting solution was stirred for 5 h at 30 °C. After this time, the crude reaction mixture was diluted with ethyl acetate (100 mL) and washed with water (20 mL × 3). The organic layer was dried over Na_2SO_4, filtered, and concentrated. The residue was purified by flash chromatography.

General procedure B for the reductive alkyl-thiolation of unactivated bromides with $PhSO_2SAlkyl$. In glovebox**,** an oven-dried screw-capped 8-mL vial equipped with a magnetic stir bar was charged with $NiBr_2$ (3.3 mg, 0.015 mmol), neocuproine (**L5**, 4.6 mg, 0.0225 mmol) and Mn powder (24.8 mg, 0.45 mmol). DMF (0.6 mL) was added via syringe and the mixture was stirred at room temperature for 10 min. The alkyl bromide **1** (0.33 mmol) was added, followed by the addition of alkyl-thiosulfonate **2** (0.3 mmol) in one portion. MeCN (0.9 mL) was subsequently added via syringe. The resulting solution was stirred for 12 h at 100 °C. After this time, the crude reaction mixture was diluted with ethyl acetate (100 mL) and washed with water (20 mL × 3). The organic layer was dried over Na_2SO_4, filtered, and concentrated. The residue was purified by flash chromatography.

General procedure C for the reductive alkyl-selenolation of unactivated bromides with $PhSO_2SeAlkyl$. In glovebox**,** an oven-dried screw-capped 8-mL vial equipped with a magnetic stir bar was charged with $NiBr_2$ (3.3 mg, 0.015 mmol), neocuproine (**L5**, 4.6 mg, 0.0225 mmol) and Mn powder (24.8 mg, 0.45 mmol). DMF (0.5 mL) was added via syringe and the mixture was stirred at room temperature for 10 min. The alkyl bromide **1** (0.33 mmol) was added, followed by the addition of alkyl-selenosulfonate **4** (0.3 mmol) in one portion. Additional DMF (1.0 mL) was subsequently added via syringe. The resulting solution was stirred for 12 h at

30 °C. After this time, the crude reaction mixture was diluted with ethyl acetate (100 mL) and washed with water (20 mL × 3). The organic layer was dried over Na_2SO_4, filtered, and concentrated. The residue was purified by flash chromatography.

Scale-up synthetic procedure for the synthesis of 5a. In glovebox**,** an oven-dried round-bottomed 20-mL flask equipped with a magnetic stir bar was charged with $NiBr_2$ (1 mol%, 6.6 mg, 0.03 mmol), neocuproine (**L5**, 9.2 mg, 0.045 mmol) and Mn powder (248 mg, 4.50 mmol). DMF (3 mL) was added via syringe and the mixture was stirred at room temperature for 10 min. The (3-bromopropyl)benzene **1a** (657 mg, 510 μL 3.3 mmol) was added, followed by the addition of S-(2-phenoxyethyl) benzenesulfonothioate **4a** (883 mg, 3.0 mmol) in one portion. MeCN (4.5 mL) was subsequently added via syringe. The resulting solution was stirred for 16 h at 100 °C. After this time, the crude reaction mixture was diluted with ethyl acetate (100 mL) and washed with water (20 mL × 3). The organic layer was dried over Na_2SO_4, filtered, and concentrated. The residue was purified by flash chromatography.

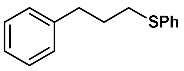

phenyl(3-phenylpropyl)sulfane (**3a**). The title compound was synthesized according to the General Procedure A, from (3-bromopropyl)benzene (99.5 mg, 76 μL, 0.50 mmol) and *S*-phenyl benzenesulfonothioate (137.7 mg, 0.55 mmol). Reaction time: 5 h. The product was purified by column chromatography on silica gel (Petroleum ether).

Pale yellow oil, 108.6 mg (95%).

^{1}H NMR (400 MHz, $CDCl_3$) δ 7.30 – 7.19 (m, 6H), 7.18 – 7.08 (m, 4H), 2.87 (t, *J* = 7.3 Hz, 2H), 2.71 (t, *J* = 7.5 Hz, 2H), 1.93 (p, *J* = 7.4 Hz, 2H).

^{13}C NMR (100 MHz, $CDCl_3$) δ 140.9, 136.3, 128.7, 128.5, 128.1, 128.1, 125.6, 125.4, 34.3, 32.5, 30.3.

FT-IR (ATR) 3060, 3025, 2925, 2854, 1496, 736, 690 cm^{-1}.

HRMS (CI) m/z ($M+H^+$) calcd for $C_{15}H_{17}S$: 229.1051, found: 229.1055.

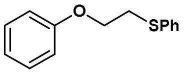

(2-phenoxyethyl)(phenyl)sulfane (**3b**). The title compound was synthesized according to the General Procedure A, from (2-bromoethoxy)benzene (100.5 mg, 0.50 mmol) and *S*-phenyl benzenesulfonothioate (137.7 mg, 0.55 mmol). Reaction time: 5 h. The product was purified by column chromatography on silica gel (Petroleum ether).

Off-white soild, 112.8 mg (98%).

^{1}H NMR (400 MHz, $CDCl_3$) δ 7.41 – 7.33 (m, 2H), 7.21 (ddt, *J* = 25.0, 16.0, 7.3 Hz, 5H), 6.91 (t, *J* = 7.4 Hz, 1H), 6.86 – 6.78 (m, 2H), 4.09 (t, *J* = 7.0 Hz, 2H), 3.24 (t, *J* = 7.0 Hz, 2H).

^{13}C NMR (100 MHz, $CDCl_3$) δ 158.4, 135.5, 129.8, 129.5, 129.1, 126.5, 121.1, 114.6, 66.6, 32.8.

FT-IR (ATR) 2965, 2920, 1585, 1240, 732, 687 cm^{-1}.

HRMS (CI) m/z ($M+H^+$) calcd for $C_{14}H_{15}OS$: 231.0844, found: 231.0847.

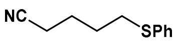

5-(phenylthio)pentanenitrile (**3c**). The title compound was synthesized according to the General Procedure A, from 5-bromopentanenitrile (81.0 mg, 58 μL, 0.50 mmol) and *S*-phenyl benzenesulfonothioate

(137.7 mg, 0.55 mmol). Reaction time: 5 h. The product was purified by column chromatography on silica gel (0→10% ethyl acetate/Petroleum ether).

Pale yellow oil, 84.4 mg (88%).

^{1}H NMR (400 MHz, $CDCl_3$) δ 7.28 (t, J = 7.9 Hz, 2H), 6.95 (t, J = 7.4 Hz, 1H), 6.89 (d, J = 8.1 Hz, 2H), 4.13 (t, J = 6.6 Hz, 2H), 2.89 (t, J = 6.6 Hz, 2H), 2.71 – 2.62 (m, 2H), 2.35 (q, J = 6.0, 4.7 Hz, 2H), 1.77 (p, J = 3.3 Hz, 4H).

^{13}C NMR (100 MHz, $CDCl_3$) δ 158.4, 129.6, 121.1, 119.4, 114.6, 67.8, 31.7, 31.1, 28.4, 24.3, 16.8.

FT-IR (ATR) 2931, 2246, 1583, 1480, 1438, 738, 690 cm^{-1}.

HRMS (CI) m/z $(M+H^+)$ calcd for $C_{11}H_{14}NS$: 192.0847, found: 192.0840.

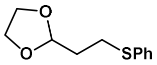

2-(2-(phenylthio)ethyl)-1,3-dioxolane (**3d**). The title compound was synthesized according to the General Procedure A, from 2-(2-bromoethyl)-1,3-dioxolane (90.5 mg, 59 μL, 0.50 mmol) and *S*-phenyl benzenesulfonothioate (137.7 mg, 0.55 mmol). Reaction time: 5 h. The product was purified by column chromatography on silica gel (0→10% ethyl acetate/Petroleum ether).

Pale yellow oil, 91.2 mg (87%).

^{1}H NMR (400 MHz, $CDCl_3$) δ 7.36 – 7.23 (m, 4H), 7.19 – 7.10 (m, 1H), 4.97 (t, *J* = 4.5 Hz, 1H), 3.97 – 3.80 (m, 4H), 3.06 – 2.97 (m, 2H), 2.03 – 1.96 (m, 2H).

^{13}C NMR (100 MHz, $CDCl_3$) δ 136.3, 129.1, 128.9, 125.9, 103.1, 65.0, 33.5, 27.8.

FT-IR (ATR) 2926, 2883,1583, 1480, 1130, 737, 690 cm^{-1}.

HRMS (CI) m/z $(M+H^+)$ calcd for $C_{11}H_{15}O_2S$: 211.0793, found: 211.0782.

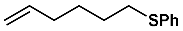

hex-5-en-1-yl(phenyl)sulfane (**3e**). The title compound was synthesized according to the General Procedure A, from 6-bromohex-1-ene (81.5 mg, 67 μL, 0.50 mmol) and *S*-phenyl benzenesulfonothioate (137.7 mg, 0.55 mmol). Reaction time: 5 h. The product was purified by column chromatography on silica gel (Petroleum ether).

Pale yellow oil, 87.0 mg (90%).

^{1}H NMR (400 MHz, $CDCl_3$) δ 7.33 – 7.23 (m, 4H), 7.17 – 7.11 (m, 1H), 5.77 (ddt, *J* = 16.9, 10.2, 6.6 Hz, 1H), 5.03 – 4.91 (m, 2H), 2.91 (t, *J* = 7.3 Hz, 2H), 2.06 (q, *J* = 7.1 Hz, 2H), 1.65 (q, *J* = 7.6, 7.1 Hz, 2H), 1.53 (q, *J* = 7.9 Hz, 2H).

^{13}C NMR (100 MHz, $CDCl_3$) δ 138.5, 137.0, 129.0, 128.9, 125.8, 114.8, 33.5, 33.3, 28.7, 28.1.

FT-IR (ATR) 2926, 2854, 1480, 1438, 910, 735, 689 cm^{-1}.

HRMS (CI) m/z $(M+H^+)$ calcd for $C_{12}H_{17}S$: 193.1051, found: 193.1047.

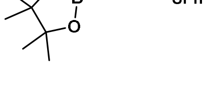

4,4,5,5-tetramethyl-2-(3-(phenylthio)propyl)-1,3,2-dioxaborolane (**3f**). The title compound was synthesized according to the General Procedure A, from 2-(3-bromopropyl)-4,4,5,5-tetramethyl-1,3,2-dioxaborolane (124.5 mg, 106 μL, 0.50 mmol) and *S*-phenyl benzenesulfonothioate (137.7 mg, 0.55 mmol).

Reaction time: 5 h. The product was purified by column chromatography on silica gel (0→10% ethyl acetate/Petroleum ether).

Pale yellow oil, 116.5 mg (84%).

^{1}H NMR (400 MHz, $CDCl_3$) δ 7.32 (dd, *J* = 8.3, 1.3 Hz, 2H), 7.24 (dd, *J* = 8.6, 6.9 Hz, 2H), 7.15 – 7.09 (m, 1H), 2.95 – 2.89 (m, 2H), 1.81 – 1.73 (m, 2H), 1.23 (s, 12H), 0.92 (t, *J* = 7.7 Hz, 2H).

^{13}C NMR (100 MHz, $CDCl_3$) δ 137.2, 128.8, 128.6, 125.5, 83.1, 35.6, 24.9, 24.0. (The carbon directly attached to the boron atom was not observed due to quadrupolar relaxation.)

FT-IR (ATR) 2977, 2926, 1371, 1143, 737, 690 cm^{-1}.

HRMS (CI) m/z ($M+H^+$) calcd for $C_{15}H_{24}BO_2S$: 279.1590, found: 279.1597.

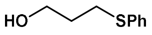

3-(phenylthio)propan-1-ol (**3g**). The title compound was synthesized according to the General Procedure A, from 3-bromopropan-1-ol (69.5 mg, 45 μL, 0.50 mmol) and *S*-phenyl benzenesulfonothioate (137.7 mg, 0.55 mmol). Reaction time: 5 h. The product was purified by column chromatography on silica gel (0→20% ethyl acetate/Petroleum ether).

Colorless oil, 79.5 mg (94%).

^{1}H NMR (400 MHz, $CDCl_3$) δ 7.35 – 7.22 (m, 4H), 7.20 – 7.11 (m, 1H), 3.71 (t, *J* = 6.1 Hz, 2H), 3.00 (t, *J* = 7.1 Hz, 2H), 2.37 (s, 1H), 1.85 (p, *J* = 6.6 Hz, 2H).

^{13}C NMR (100 MHz, $CDCl_3$) δ 136.3, 129.1, 128.9, 126.0, 61.2, 31.7, 30.2.

FT-IR (ATR) 3345, 2927, 2877, 1583, 1479, 1438, 736, 689 cm^{-1}.

HRMS (CI) m/z ($M+H^+$) calcd for $C_9H_{13}OS$: 169.0687, found: 169.0693.

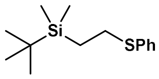

tert-butyldimethyl(2-(phenylthio)ethyl)silane (**3h**). The title compound was synthesized according to the General Procedure A, from (2-bromoethyl)(tert-butyl)dimethylsilane (111.6 mg, 107 μL, 0.50 mmol) and *S*-phenyl benzenesulfonothioate (137.7 mg, 0.55 mmol). Reaction time: 5 h. The product was purified by column chromatography on silica gel (Petroleum ether).

Pale yellow oil, 107.4 mg (80%).

^{1}H NMR (400 MHz, $CDCl_3$) δ 7.46 (dd, *J* = 8.3, 1.3 Hz, 2H), 7.36 (dd, *J* = 8.6, 6.9 Hz, 2H), 7.28 – 7.23 (m, 1H), 3.89 (t, *J* = 7.1 Hz, 2H), 3.16 (t, *J* = 7.1 Hz, 2H), 0.99 (s, 9H), 0.15 (s, 6H).

^{13}C NMR (100 MHz, $CDCl_3$) δ 136.5, 129.2, 129.0, 126.0, 62.5, 36.0, 26.0, 18.4, -5.2.

FT-IR (ATR) 2954, 2928, 2856, 1254, 1086, 890, 775 cm^{-1}.

HRMS (CI) m/z ($M+H^+$) calcd for $C_{14}H_{25}OSSi$: 269.1395, found: 269.1392.

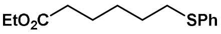

ethyl 6-(phenylthio)hexanoate (**3i**). The title compound was synthesized according to the General Procedure A, from ethyl 6-bromohexanoate (111.6 mg, 89 μL, 0.50 mmol) and *S*-phenyl benzenesulfonothioate (137.7 mg, 0.55 mmol). Reaction time: 5 h. The product was purified by column chromatography on silica gel (0→10% ethyl acetate/Petroleum ether).

Pale yellow oil, 116.0 mg (92%).

^{1}H NMR (400 MHz, $CDCl_3$) δ 7.33 – 7.22 (m, 4H), 7.18 – 7.11 (m, 1H), 4.11 (q, *J* = 7.1 Hz, 2H), 2.90 (t, *J* = 7.3 Hz, 2H), 2.27 (t, *J* = 7.4 Hz, 2H), 1.68 – 1.59 (m, 4H), 1.49 – 1.41 (m, 2H), 1.23 (t, *J* = 7.1 Hz, 3H).

^{13}C NMR (100 MHz, $CDCl_3$) δ 173.5, 136.7, 128.9, 128.8, 125.7, 60.2, 34.1, 33.3, 28.8, 28.2, 24.5, 14.2.

FT-IR (ATR) 2933, 2860, 1731, 1178, 737, 690 cm^{-1}.

HRMS (CI) m/z ($M+H^+$) calcd for $C_{14}H_{21}O_2S$: 253.1262, found: 253.1260.

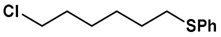

(6-chlorohexyl)(phenyl)sulfane (**3j**). The title compound was synthesized according to the General Procedure A, from 1-bromo-6-chlorohexane (99.8 mg, 75 µL, 0.50 mmol) and *S*-phenyl benzenesulfonothioate (137.7 mg, 0.55 mmol). Reaction time: 5 h. The product was purified by column chromatography on silica gel (Petroleum ether).

Pale yellow oil, 102.9 mg (90%).

^{1}H NMR (400 MHz, $CDCl_3$) δ 7.32 – 7.23 (m, 4H), 7.17 – 7.11 (m, 1H), 3.49 (t, *J* = 6.7 Hz, 2H), 2.92 – 2.87 (m, 2H), 1.78 – 1.70 (m, 2H), 1.68 – 1.61 (m, 2H), 1.43 (p, *J* = 3.7 Hz, 4H).

^{13}C NMR (100 MHz, $CDCl_3$) δ 136.9, 129.0, 128.9, 125.8, 45.0, 33.5, 32.5, 29.0, 28.1, 26.5.

FT-IR (ATR) 2931, 2856, 1584, 1479, 1438, 736, 689 cm^{-1}.

HRMS (CI) m/z ($M+H^+$) calcd for $C_{12}H_{18}ClS$: 229.0818, found: 229.0824.

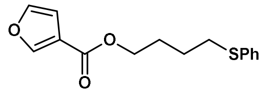

4-(phenylthio)butyl furan-3-carboxylate (**3k**). The title compound was synthesized according to the General Procedure A, from 4-bromobutyl furan-3-carboxylate (123.6 mg, 0.50 mmol) and *S*-phenyl benzenesulfonothioate (137.7 mg, 0.55 mmol). Reaction time: 5 h. The product was purified by column chromatography on silica gel (0→2% ethyl acetate/Petroleum ether).

Colorless oil, 134.3 mg (97%).

^{1}H NMR (400 MHz, $CDCl_3$) δ 7.95 (s, 1H), 7.39 (t, *J* = 1.8 Hz, 1H), 7.35 – 7.30 (m, 2H), 7.25 (t, *J* = 7.6 Hz, 2H), 7.18 – 7.12 (m, 1H), 6.70 (d, *J* = 1.8 Hz, 1H), 4.24 (t, *J* = 6.3 Hz, 2H), 2.95 (t, *J* = 7.1 Hz, 2H), 1.89 – 1.82 (m, 2H), 1.78 – 1.71 (m, 2H).

^{13}C NMR (100 MHz, $CDCl_3$) δ 163.0, 147.6, 143.7, 136.3, 129.2, 128.9, 126.0, 119.4, 109.8, 63.8, 33.2, 27.7, 25.6.

FT-IR (ATR) 2955, 1719, 1305, 1158, 737, 690 cm^{-1}.

HRMS (CI) m/z (M^+) calcd for $C_{15}H_{16}O_3S$: 276.0820, found: 276.0815.

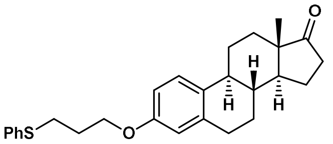

(8R,9S,13S,14S)-13-methyl-3-(3-(phenylthio)propoxy)-6,7,8,9,11,12,13,14,15,16-decahydro-17H-cyclopenta[a]phenanthren-17-one (**3l**). The title compound was synthesized according to the General Procedure A, from (8R,9S,13S,14S)-3-(3-bromopropoxy)-13-methyl-6,7,8,9,11,12,13,14,15,16-decahydro-17H-cyclopenta[a]phenanthren-17-one (78.3 mg, 0.20 mmol) and *S*-phenyl benzenesulfonothioate (55.1 mg,

0.22 mmol). Reaction time: 5 h. The product was purified by column chromatography on silica gel (0→5% ethyl acetate/Petroleum ether).

White solid, 82.4 mg (98%).

^{1}H NMR (400 MHz, $CDCl_3$) δ 7.33 (s, 2H), 7.26 (t, *J* = 7.6 Hz, 2H), 7.16 (td, *J* = 8.0, 7.2, 2.4 Hz, 2H), 6.69 (dd, *J* = 8.5, 2.8 Hz, 1H), 6.63 (d, *J* = 2.7 Hz, 1H), 4.03 (t, *J* = 6.0 Hz, 2H), 3.09 (t, *J* = 7.1 Hz, 2H), 2.87 (dt, *J* = 7.4, 4.6 Hz, 2H), 2.48 (dd, *J* = 18.8, 8.6 Hz, 1H), 2.37 (dq, *J* = 8.9, 3.4 Hz, 1H), 2.24 – 1.93 (m, 7H), 1.71 – 1.50 (m, 3H), 1.50 – 1.36 (m, 3H), 0.89 (s, 3H).

^{13}C NMR (100 MHz, $CDCl_3$) δ 220.9, 156.9, 137.8, 136.3, 132.2, 129.2, 129.0, 126.4, 126.0, 114.6, 112.2, 66.0, 50.4, 48.0, 44.0, 38.4, 35.9, 31.6, 30.2, 29.7, 28.9, 26.6, 26.0, 21.6, 13.9.

FT-IR (ATR) 2924, 1732, 1232, 735, 689 cm^{-1}.

HRMS (CI) m/z ($M+H^+$) calcd for $C_{27}H_{33}O_2S$: 421.2201, found: 421.2197.

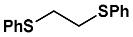

1,2-bis(phenylthio)ethane (**3m**). The title compound was synthesized according to the General Procedure A, from 1,2-dibromoethane (47.0 mg, 22 μL, 0.25 mmol) and *S*-phenyl benzenesulfonothioate (137.7 mg, 0.55 mmol). Reaction time: 5 h. The product was purified by column chromatography on silica gel (Petroleum ether).

White solid, 59.9 mg (97%).

^{1}H NMR (400 MHz, $CDCl_3$) δ 7.33 – 7.22 (m, 8H), 7.22 – 7.16 (m, 2H), 3.07 (s, 4H).

^{13}C NMR (100 MHz, $CDCl_3$) δ 135.1, 130.1, 129.1, 126.6, 33.4.

FT-IR (ATR) 2968, 2924, 1578, 1476, 731, 687 cm^{-1}.

HRMS (CI) m/z ($M+H^+$) calcd for $C_{14}H_{15}S_2$: 247.0615, found: 247.0610.

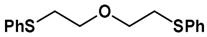

(oxybis(ethane-2,1-diyl))bis(phenylsulfane) (**3n**). The title compound was synthesized according to the General Procedure A, from 1-bromo-2-(2-bromoethoxy)ethane (58.0 mg, 32 μL, 0.25 mmol) and *S*-phenyl benzenesulfonothioate (137.7 mg, 0.55 mmol). Reaction time: 5 h. The product was purified by column chromatography on silica gel (Petroleum ether).

Colourless oil, 70.0 mg (96%).

^{1}H NMR (400 MHz, $CDCl_3$) δ 7.36 – 7.32 (m, 4H), 7.26 (dd, *J* = 8.4, 6.8 Hz, 4H), 7.19 – 7.14 (m, 2H), 3.61 (t, *J* = 6.9 Hz, 4H), 3.07 (t, *J* = 6.9 Hz, 4H).

^{13}C NMR (100 MHz, $CDCl_3$) δ 135.9, 129.5, 129.0, 126.3, 69.7, 33.2.

FT-IR (ATR) 2923, 2860, 1479, 1090, 735, 689 cm^{-1}.

HRMS (CI) m/z ($M+H^+$) calcd for $C_{16}H_{19}OS_2$: 291.0877, found: 291.0865.

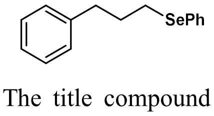

phenyl(3-phenylpropyl)selane (**3o**). The title compound was synthesized according to the General Procedure A, from (3-bromopropyl)benzene (99.5 mg, 76 μL, 0.50 mmol) and *Se*-phenyl benzenesulfonothioate (163.5 mg, 0.55 mmol). Reaction time: 5 h. The product was purified by column chromatography on silica gel (Petroleum ether).

Pale yellow oil, 136.1 mg (99%).

^{1}**H NMR** (400 MHz, $CDCl_3$) δ 7.44 (dd, *J* = 7.4, 2.2 Hz, 2H), 7.26 – 7.10 (m, 8H), 2.86 (t, *J* = 7.3 Hz, 2H), 2.69 (t, *J* = 7.5 Hz, 2H), 1.99 (p, *J* = 7.4 Hz, 2H).

^{13}**C NMR** (100 MHz, $CDCl_3$) δ 141.3, 132.6, 130.4, 129.1, 128.6, 128.4, 126.8, 126.0, 35.8, 31.7, 27.2.

FT-IR (ATR) 2931, 2853, 1476, 732, 690 cm^{-1}.

HRMS (CI) m/z ($M+H^+$) calcd for $C_{15}H_{17}Se$: 277.0495, found: 277.0496.

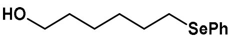

6-(phenylselanyl)hexan-1-ol (**3p**). The title compound was synthesized according to the General Procedure A, from 6-bromohexan-1-ol (90.5 mg, 66 μL, 0.50 mmol) and *Se*-phenyl benzenesulfonothioate (163.5 mg, 0.55 mmol). Reaction time: 5 h. The product was purified by column chromatography on silica gel (0→20% ethyl acetate/Petroleum ether).

White solid, 109.5 mg (85%).

^{1}**H NMR** (400 MHz, $CDCl_3$) δ 7.51 – 7.43 (m, 2H), 7.26 – 7.18 (m, 3H), 3.58 (t, *J* = 6.6 Hz, 2H), 2.89 (t, *J* = 7.4 Hz, 2H), 2.03 (s, 1H), 1.70 (p, *J* = 7.4 Hz, 2H), 1.56 – 1.49 (m, 2H), 1.44 – 1.32 (m, 4H).

^{13}**C NMR** (100 MHz, $CDCl_3$) δ 132.4, 130.6, 129.0, 126.6, 62.7, 32.5, 30.1, 29.6, 27.8, 25.2.

FT-IR (ATR) 3335, 2928, 2855, 1477, 1051, 733, 690 cm^{-1}.

HRMS (CI) m/z ($M+H^+$) calcd for $C_{12}H_{19}OSe$: 259.0601, found: 259.0603.

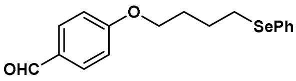

4-(4-(phenylselanyl)butoxy)benzaldehyde (**3q**). The title compound was synthesized according to the General Procedure A, from 4-(4-bromobutoxy)benzaldehyde (128.6 mg, 0.50 mmol) and *Se*-phenyl benzenesulfonothioate (163.5 mg, 0.55 mmol). Reaction time: 5 h. The product was purified by column chromatography on silica gel (0→2.5% ethyl acetate/Petroleum ether).

Off-white solid, 159.8 mg (96%).

^{1}**H NMR** (400 MHz, $CDCl_3$) δ 9.84 (s, 1H), 7.79 (d, *J* = 8.7 Hz, 2H), 7.51 – 7.45 (m, 2H), 7.26 – 7.19 (m, 3H), 6.93 (d, *J* = 8.7 Hz, 2H), 3.99 (t, *J* = 5.8 Hz, 2H), 2.95 (t, *J* = 6.7 Hz, 2H), 1.89 (dtt, *J* = 10.2, 7.2, 3.0 Hz, 4H).

^{13}**C NMR** (100 MHz, $CDCl_3$) δ 190.7, 163.9, 132.5, 131.9, 130.1, 129.8, 129.0, 126.8, 114.7, 67.6, 29.0, 27.3, 26.5.

FT-IR (ATR) 2938, 2872, 1688, 1598, 1252, 1156, 735, 691 cm^{-1}.

HRMS (ESI) m/z ($M+Na^+$) calcd for $C_{17}H_{18}NaO_2Se$: 357.0370, found: 357.0785.

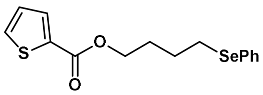

4-(phenylselanyl)butyl thiophene-2-carboxylate (**3r**). The title compound was synthesized according to the General Procedure A, from 4-bromobutyl thiophene-2-carboxylate (131.6 mg, 0.50 mmol) and *Se*-phenyl benzenesulfonothioate (163.5 mg, 0.55 mmol). Reaction time: 5 h. The product was purified by column chromatography on silica gel (0→2% ethyl acetate/Petroleum ether).

Pale yellow oil, 167.9 mg (99%).

^{1}**H NMR** (400 MHz, $CDCl_3$) δ 7.74 (dd, *J* = 3.7, 1.3 Hz, 1H), 7.55 – 7.42 (m, 3H), 7.26 – 7.16 (m, 3H), 7.05 (dd, *J* = 5.0, 3.7 Hz, 1H), 4.27 (t, *J* = 6.0 Hz, 2H), 2.93 (t, *J* = 6.9 Hz, 2H), 1.88 – 1.78 (m, 4H).

^{13}**C NMR** (100 MHz, $CDCl_3$) δ 162.1, 133.8, 133.3, 132.6, 132.3, 130.0, 129.0, 127.7, 126.8, 64.4, 28.7, 27.2, 26.5.

FT-IR (ATR) 2937, 1704, 1418, 1257, 1073, 720, 669 cm^{-1}.

HRMS (CI) m/z ($M+H^+$) calcd for $C_{15}H_{17}O_2SSe$: 341.0114, found: 341.0108.

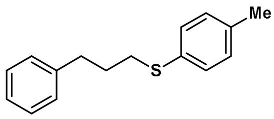

(3-phenylpropyl)(p-tolyl)sulfane (**3s**). The title compound was synthesized according to the General Procedure A, from (3-bromopropyl)benzene (99.5 mg, 76 μL, 0.50 mmol) and *S*-(p-tolyl) benzenesulfonothioate (145.4 mg, 0.55 mmol). Reaction time: 5 h. The product was purified by column chromatography on silica gel (Petroleum ether).
Pale yellow oil, 114.6 mg (94%).

^{1}**H NMR** (400 MHz, $CDCl_3$) δ 7.22 (t, *J* = 8.1 Hz, 4H), 7.17 – 7.12 (m, 3H), 7.05 (d, *J* = 7.9 Hz, 2H), 2.87 – 2.82 (m, 2H), 2.71 (t, *J* = 7.5 Hz, 2H), 2.28 (s, 3H), 1.90 (q, *J* = 7.3 Hz, 2H).

^{13}**C NMR** (100 MHz, $CDCl_3$) δ 141.4, 136.0, 132.8, 130.1, 129.7, 128.6, 128.4, 126.0, 34.7, 33.7, 30.8, 21.1.

FT-IR (ATR) 2920, 1492, 1075, 801, 742, 698 cm^{-1}.

HRMS (CI) m/z ($M+H^+$) calcd for $C_{16}H_{19}S$: 243.1207, found: 243.1205.

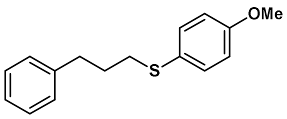

(4-methoxyphenyl)(3-phenylpropyl)sulfane (**3t**). The title compound was synthesized according to the General Procedure A, from (3-bromopropyl)benzene (99.5 mg, 76 μL, 0.50 mmol) and *S*-(4-methoxyphenyl) benzenesulfonothioate (140.2 mg, 0.5 mmol) and (3-bromopropyl)benzene (119.4 mg, 92 μL, 0.60 mmol). Reaction time: 5 h. The product was purified by column chromatography on silica gel (Petroleum ether).
Yellow oil, 119.6 mg (92%).

^{1}**H NMR** (400 MHz, $CDCl_3$) δ 7.34 – 7.30 (m, 2H), 7.26 (dd, *J* = 8.3, 6.4 Hz, 2H), 7.19 – 7.12 (m, 3H), 6.85 – 6.78 (m, 2H), 3.76 (s, 3H), 2.83 – 2.78 (m, 2H), 2.71 (t, *J* = 7.6 Hz, 2H), 1.88 (p, *J* = 7.4 Hz, 2H).

^{13}**C NMR** (100 MHz, $CDCl_3$) δ 158.9, 141.5, 133.3, 128.6, 128.5, 126.6, 126.0, 114.6, 55.4, 35.2, 34.7, 30.9.

FT-IR (ATR) 2923, 1492, 1241, 1030, 824, 743, 698 cm^{-1}.

HRMS (CI) m/z ($M+H^+$) calcd for $C_{16}H_{19}OS$: 259.1157, found: 259.1161.

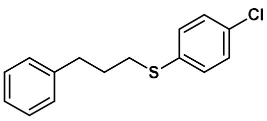

(4-chlorophenyl)(3-phenylpropyl)sulfane (**3u**). The title compound was synthesized according to the General Procedure A, from (3-bromopropyl)benzene (99.5 mg, 76 μL, 0.50 mmol) and *S*-(4-chlorophenyl)

benzenesulfonothioate (156.6 mg, 0.55 mmol). Reaction time: 5 h. The product was purified by column chromatography on silica gel (Petroleum ether).
White solid, 120.5 mg (92%).

^{1}H NMR (400 MHz, $CDCl_3$) δ 7.26 (t, J = 7.4 Hz, 2H), 7.22 – 7.10 (m, 7H), 2.85 (t, J = 7.3 Hz, 2H), 2.71 (t, J = 7.5 Hz, 2H), 1.92 (p, J = 7.4 Hz, 2H).

^{13}C NMR (100 MHz, $CDCl_3$) δ 141.1, 135.2, 131.8, 130.4, 129.0, 128.5, 128.5, 126.1, 34.7, 33.1, 30.6.

FT-IR (ATR) 2987, 2923, 1475, 1094, 1010, 809, 744, 698 cm^{-1}.

HRMS (CI) m/z ($M+H^+$) calcd for $C_{15}H_{16}ClS$: 263.0661, found: 263.0667.

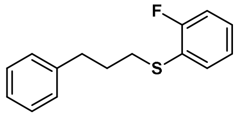

(2-fluorophenyl)(3-phenylpropyl)sulfane (**3v**). The title compound was synthesized according to the General Procedure A, from (3-bromopropyl)benzene (99.5 mg, 76 μL, 0.50 mmol) and *S*-(2-fluorophenyl) benzenesulfonothioate (147.6 mg, 0.55 mmol). Reaction time: 5 h. The product was purified by column chromatography on silica gel (Petroleum ether).
Pale yellow oil, 122.2 mg (99%).

^{1}H NMR (400 MHz, $CDCl_3$) δ 7.33 – 7.11 (m, 7H), 7.03 (q, J = 8.7, 7.8 Hz, 2H), 2.88 (t, J = 7.3 Hz, 2H), 2.73 (t, J = 7.6 Hz, 2H), 1.92 (p, J = 7.5 Hz, 2H).

^{13}C NMR (100 MHz, $CDCl_3$) δ 162.7, 160.3, 141.3, 131.9 (d, J_{C-F} = 2 Hz), 128.5 (d, J_{C-F} = 7 Hz), 128.2 (d, J_{C-F} = 8 Hz), 126.0, 124.5 (d, J_{C-F} = 4 Hz), 123.3 (d, J_{C-F} = 18 Hz), 115.6 (d, J_{C-F} = 22 Hz), 34.6, 32.6, 30.8.

FT-IR (ATR) 2924, 1471, 1219, 1072, 744, 698 cm^{-1}.

HRMS (CI) m/z ($M+H^+$) calcd for $C_{15}H_{16}FS$: 247.0957, found: 247.0964.

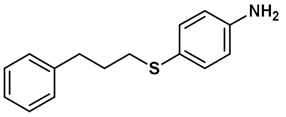

4-((3-phenylpropyl)thio)aniline (**3w**). The title compound was synthesized according to the General Procedure A, from (3-bromopropyl)benzene (99.5 mg, 76 μL, 0.50 mmol) and *S*-(4-aminophenyl) benzenesulfonothioate (145.9 mg, 0.55 mmol). Reaction time: 12 h. The product was purified by column chromatography on silica gel (0→10% ethyl acetate/Petroleum ether).
Orange red oil, 105.9 mg (87%).

^{1}H NMR (400 MHz, $CDCl_3$) δ 7.26 – 7.11 (m, 7H), 6.60 – 6.50 (m, 2H), 3.61 (s, 2H), 2.75 (t, J = 7.2 Hz, 2H), 2.71 – 2.66 (m, 2H), 1.85 (p, J = 7.3 Hz, 2H).

^{13}C NMR (100 MHz, $CDCl_3$) δ 145.9, 141.6, 133.9, 128.5, 128.3, 125.8, 123.2, 115.6, 35.7, 34.6, 30.8.

FT-IR (ATR) 3367, 2923, 1619, 1597, 1494, 1276, 821, 743, 698 cm^{-1}.

HRMS (CI) m/z ($M+H^+$) calcd for $C_{15}H_{18}NS$: 244.1160, found: 244.1155.

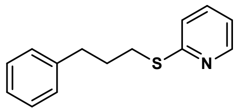

2-((3-phenylpropyl)thio)pyridine (**3x**). The title compound was synthesized according to the General Procedure A, from (3-bromopropyl)benzene (99.5 mg, 76 μL, 0.50 mmol) and *S*-(pyridin-2-yl) benzenesulfonothioate (138.2 mg, 0.55 mmol). Reaction time: 12 h. The product was purified by column chromatography on silica gel (0→10% ethyl acetate/Petroleum ether).

Pale yellow oil, 78.3 mg (68%).

^{1}H NMR (400 MHz, $CDCl_3$) δ 8.39 (dt, *J* = 5.0, 1.3 Hz, 1H), 7.41 (td, *J* = 7.7, 1.9 Hz, 1H), 7.28 – 7.23 (m, 2H), 7.20 – 7.11 (m, 4H), 6.91 (ddd, *J* = 7.4, 4.9, 1.0 Hz, 1H), 3.17 (t, *J* = 7.2 Hz, 2H), 2.79 – 2.73 (m, 2H), 2.03 (p, *J* = 7.5 Hz, 2H).

^{13}C NMR (100 MHz, $CDCl_3$) δ 159.3, 149.5, 141.5, 135.9, 128.6, 128.4, 125.9, 122.2, 119.3, 34.9, 31.1, 29.5.

FT-IR (ATR) 2923, 1577, 1453, 1413, 1123, 754, 698 cm^{-1}.

HRMS (CI) m/z ($M+H^+$) calcd for $C_{14}H_{16}NS$: 230.1003, found: 230.1004.

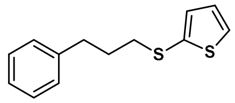

2-((3-phenylpropyl)thio)thiophene (**3y**). The title compound was synthesized according to the General Procedure A, from (3-bromopropyl)benzene (99.5 mg, 76 μL, 0.50 mmol) and *S*-(thiophen-2-yl) benzenesulfonothioate (141.0 mg, 0.55 mmol). Reaction time: 12 h. The product was purified by column chromatography on silica gel (0→2.5% ethyl acetate/Petroleum ether).

Yellow oil, 118.2 mg (99%).

^{1}H NMR (400 MHz, $CDCl_3$) δ 7.29 – 7.22 (m, 3H), 7.18 – 7.12 (m, 3H), 7.09 (dd, *J* = 3.5, 1.3 Hz, 1H), 6.93 (dd, *J* = 5.4, 3.5 Hz, 1H), 2.76 (t, *J* = 7.2 Hz, 2H), 2.73 – 2.68 (m, 2H), 1.90 (p, *J* = 7.4 Hz, 2H).

^{13}C NMR (100 MHz, $CDCl_3$) δ 141.4, 134.5, 133.6, 129.2, 128.5, 128.5, 127.6, 126.0, 38.2, 34.4, 30.9.

FT-IR (ATR) 2924, 1495, 1435, 1215, 845, 742 cm^{-1}.

HRMS (CI) m/z ($M+H^+$) calcd for $C_{13}H_{15}S_2$: 235.0615, found: 235.0612.

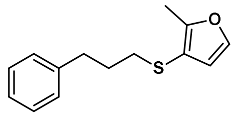

2-methyl-3-((3-phenylpropyl)thio)furan (**3z**). The title compound was synthesized according to the General Procedure A, from (3-bromopropyl)benzene (99.5 mg, 76 μL, 0.50 mmol) and *S*-(2-methylfuran-3-yl) benzenesulfonothioate (139.9 mg, 0.55 mmol). Reaction time: 12 h. The product was purified by column chromatography on silica gel (0→2.5% ethyl acetate/Petroleum ether).

Pale yellow oil, 113.3 mg (97%).

^{1}H NMR (400 MHz, $CDCl_3$) δ 7.26 (ddd, *J* = 7.6, 5.7, 2.0 Hz, 3H), 7.19 – 7.13 (m, 3H), 6.31 (d, *J* = 2.0 Hz, 1H), 2.73 – 2.68 (m, 2H), 2.61 (t, *J* = 7.2 Hz, 2H), 2.33 (s, 3H), 1.84 (p, *J* = 7.4 Hz, 2H).

^{13}C NMR (100 MHz, $CDCl_3$) δ 154.8, 141.6, 140.6, 128.6, 128.5, 126.0, 115.1, 110.3, 35.3, 34.6, 31.1, 12.0.

FT-IR (ATR) 2919, 1418, 1222, 1087, 732, 698 cm^{-1}.

HRMS (CI) m/z ($M+H^+$) calcd for $C_{14}H_{17}OS$: 233.1000, found: 233.0994.

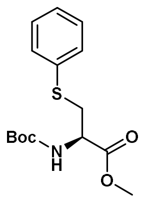

methyl N-(tert-butoxycarbonyl)-S-phenyl-L-cysteinate (**3aa**). The title compound was synthesized according to the General Procedure A, from (R)-3-bromo-2-((tert-butoxycarbonyl)amino)propanoate (84.6 mg, 0.30 mmol) and *S*-phenyl benzenesulfonothioate (82.6 mg, 0.33 mmol). Reaction time: 6 h. The product was purified by column chromatography on silica gel (0→20% ethyl acetate/Petroleum ether).

Pale yellow oil, 86.9 mg (93%).

^{1}H NMR (400 MHz, $CDCl_3$) δ 7.43 – 7.39 (m, 2H), 7.28 (t, J = 7.6 Hz, 2H), 7.23 – 7.19 (m, 1H), 5.39 (d, J = 8.0 Hz, 1H), 4.57 (dt, J = 9.3, 4.9 Hz, 1H), 3.53 (s, 3H), 3.37 (d, J = 4.9 Hz, 2H), 1.42 (s, 9H).

^{13}C NMR (100 MHz, $CDCl_3$) δ 171.1, 155.0, 134.8, 131.1, 129.1, 127.1, 80.2, 53.3, 52.4, 37.3, 28.3.

FT-IR (ATR) 3373, 2976, 1711, 1500, 1160, 741, 691 cm^{-1}.

HRMS (CI) m/z (M^+) calcd for $C_{15}H_{21}NO_4S$: 311.1191, found: 311.1187.

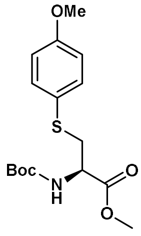

methyl N-(tert-butoxycarbonyl)-S-(4-methoxyphenyl)-L-cysteinate (**3ab**). The title compound was synthesized according to the General Procedure A, from methyl (R)-3-bromo-2-((tert-butoxycarbonyl)amino)propanoate (84.6 mg, 0.30 mmol) and *S*-(4-methoxyphenyl) benzenesulfonothioate (92.5 mg, 0.33 mmol). Reaction time: 6 h. The product was purified by column chromatography on silica gel (0→20% ethyl acetate/Petroleum ether).

Pale yellow oil, 84.5 mg (82%).

^{1}H NMR (400 MHz, $CDCl_3$) δ 7.41 – 7.37 (m, 2H), 6.85 – 6.81 (m, 2H), 5.37 (d, *J* = 8.2 Hz, 1H), 4.50 (dt, *J* = 9.3, 5.0 Hz, 1H), 3.78 (s, 3H), 3.54 (s, 3H), 3.25 (d, *J* = 5.0 Hz, 2H), 1.42 (s, 9H).

^{13}C NMR (100 MHz, $CDCl_3$) δ 171.2, 159.5, 155.0, 134.6, 124.8, 114.7, 80.0, 55.4, 53.3, 52.3, 38.8, 28.3.

FT-IR (ATR) 3374, 2972, 1712, 1493, 1243, 1161, 827 cm^{-1}.

HRMS (CI) m/z ($M+H^+$) calcd for $C_{16}H_{24}NO_5S$: 342.1375, found: 342.1361.

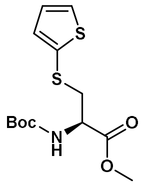

methyl N-(tert-butoxycarbonyl)-S-(thiophen-2-yl)-L-cysteinate (**3ac**). The title compound was synthesized according to the General Procedure A, from methyl (R)-3-bromo-2-((tert-butoxycarbonyl)amino)propanoate (84.6 mg, 0.30 mmol) and *S*-(thiophen-2-yl) benzenesulfonothioate (84.6

mg, 0.33 mmol). Reaction time: 12 h. The product was purified by column chromatography on silica gel (0→20% ethyl acetate/Petroleum ether).

Pale yellow oil, 88.2 mg (92%).

^{1}H NMR (400 MHz, $CDCl_3$) δ 7.36 (dd, *J* = 5.3, 1.3 Hz, 1H), 7.16 (dd, *J* = 3.6, 1.3 Hz, 1H), 6.96 (dd, *J* = 5.4, 3.6 Hz, 1H), 5.36 (d, *J* = 8.1 Hz, 1H), 4.53 (dt, *J* = 8.8, 5.3 Hz, 1H), 3.61 (s, 3H), 3.23 (qd, *J* = 14.0, 4.9 Hz, 2H).

^{13}C NMR (100 MHz, $CDCl_3$) δ 171.1, 155.1, 135.1, 133.0, 130.3, 127.7, 80.2, 53.2, 52.5, 41.2, 28.4.

FT-IR (ATR) 3365, 2976, 2928, 1745, 1711, 1160, 702 cm^{-1}.

HRMS (CI) m/z (M^+) calcd for $C_{13}H_{19}NO_4Si$: 317.0755, found: 317.0760.

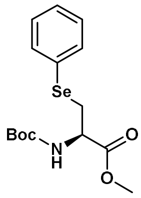

methyl (R)-2-((tert-butoxycarbonyl)amino)-3-(phenylselanyl)propanoate (**3ad**). The title compound was synthesized according to the General Procedure A, from methyl (R)-3-bromo-2-((tert-butoxycarbonyl)amino)propanoate (84.6 mg, 0.30 mmol) and *Se*-phenyl benzenesulfonoselenoate (98.1 mg, 0.33 mmol). Reaction time: 6 h. The product was purified by column chromatography on silica gel (0→20% ethyl acetate/Petroleum ether).

Pale yellow oil, 101.4 mg (94%).

^{1}H NMR (400 MHz, $CDCl_3$) δ 7.56 – 7.52 (m, 2H), 7.27 – 7.24 (m, 3H), 5.37 (d, *J* = 8.2 Hz, 1H), 4.66 (dt, *J* = 9.0, 4.8 Hz, 1H), 3.49 (s, 3H), 3.33 (d, *J* = 5.0 Hz, 2H), 1.41 (s, 9H).

^{13}C NMR (100 MHz, $CDCl_3$) δ 171.2, 155.0, 133.8, 129.2, 129.0, 127.6, 80.1, 53.4, 52.3, 30.8, 28.4.

FT-IR (ATR) 3374, 2975, 1711, 1160, 737, 691 cm^{-1}.

HRMS (CI) m/z ($M+H^+$) calcd for $C_{15}H_{22}NO_4S$: 360.0714, found: 360.0732.

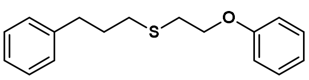

(2-phenoxyethyl)(3-phenylpropyl)sulfane (**5a**). The title compound was synthesized according to the General Procedure B, from *S*-(2-phenoxyethyl) benzenesulfonothioate (88.3 mg, 0.30 mmol) and (3-bromopropyl)benzene (65.7 mg, 51 μL, 0.33 mmol). Reaction time: 12 h. The product was purified by column chromatography on silica gel (0→1% ethyl acetate/Petroleum ether).

Pale yellow oil, 80.2 mg (98%).

^{1}H NMR (400 MHz, $CDCl_3$) δ 7.28 – 7.22 (m, 4H), 7.16 (dd, *J* = 7.7, 5.7 Hz, 3H), 6.92 (t, *J* = 7.4 Hz, 1H), 6.85 (d, *J* = 8.1 Hz, 2H), 4.07 (t, *J* = 6.8 Hz, 2H), 2.84 (t, *J* = 6.8 Hz, 2H), 2.70 (t, *J* = 7.6 Hz, 2H), 2.59 (t, *J* = 7.3 Hz, 2H), 1.91 (p, *J* = 7.4 Hz, 2H).

^{13}C NMR (100 MHz, $CDCl_3$) δ 158.5, 141.4, 129.5, 128.5, 128.4, 126.0, 121.0, 114.6, 67.7, 34.8, 32.0, 31.3, 30.9.

FT-IR (ATR) 2922, 1599, 1495, 1240, 1016, 750, 690 cm^{-1}.

HRMS (CI) m/z (M^+) calcd for $C_{17}H_{20}OS$: 272.1235, found: 272.1232.

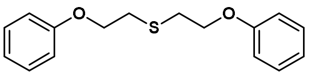

bis(2-phenoxyethyl)sulfane (**5b**). The title compound was synthesized according to the General Procedure B, from *S*-(2-phenoxyethyl) benzenesulfonothioate (88.3 mg, 0.30 mmol) and (2-bromoethoxy)benzene (66.3, 0.33 mmol). Reaction time: 12 h. The product was purified by column chromatography on silica gel (0→1% ethyl acetate/Petroleum ether).

Pale yellow oil, 75.2 mg (91%).

^{1}H NMR (400 MHz, $CDCl_3$) δ 7.26 (t, J = 7.8 Hz, 4H), 6.94 (t, J = 7.4 Hz, 2H), 6.88 (d, J = 8.1 Hz, 4H), 4.16 (t, J = 6.7 Hz, 4H), 2.99 (t, J = 6.6 Hz, 4H).

^{13}C NMR (100 MHz, $CDCl_3$) δ 158.5, 129.6, 121.1, 114.7, 68.0, 31.8.

FT-IR (ATR) 2967, 2927, 1599, 1496, 1236, 1033, 751, 687 cm^{-1}.

HRMS (CI) m/z (M^+) calcd for $C_{16}H_{18}O_2S$: 274.1028, found: 274.1029.

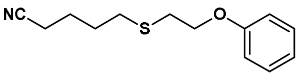

5-((2-phenoxyethyl)thio)pentanenitrile (**5c**). The title compound was synthesized according to the General Procedure B, from *S*-(2-phenoxyethyl) benzenesulfonothioate (88.3 mg, 0.30 mmol) and 5-bromopentanenitrile (48.6 mg, 39 μL, 0.33 mmol). Reaction time: 12 h. The product was purified by column chromatography on silica gel (0→10% ethyl acetate/Petroleum ether).

Pale yellow oil, 59.5 mg (84%).

^{1}H NMR (400 MHz, $CDCl_3$) δ 7.28 (t, *J* = 7.9 Hz, 2H), 6.95 (t, *J* = 7.4 Hz, 1H), 6.89 (d, *J* = 8.1 Hz, 2H), 4.13 (t, *J* = 6.6 Hz, 2H), 2.89 (t, *J* = 6.6 Hz, 2H), 2.71 – 2.62 (m, 2H), 2.35 (q, *J* = 6.0, 4.7 Hz, 2H), 1.77 (p, *J* = 3.3 Hz, 4H).

^{13}C NMR (100 MHz, $CDCl_3$) δ 158.4, 129.6, 121.1, 119.4, 114.6, 67.8, 31.7, 31.1, 28.4, 24.3, 16.8.

FT-IR (ATR) 2925, 2867, 2245, 1599, 1495, 1239, 753, 692 cm^{-1}.

HRMS (CI) m/z (M^+) calcd for $C_{13}H_{17}NOS$: 235.1031, found: 235.1037.

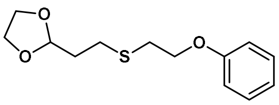

2-(2-((2-phenoxyethyl)thio)ethyl)-1,3-dioxolane (**5d**). The title compound was synthesized according to the General Procedure B, from *S*-(2-phenoxyethyl) benzenesulfonothioate (88.3 mg, 0.30 mmol) and 2-(2-bromoethyl)-1,3-dioxolane (59.7 mg, 39 μL, 0.33 mmol). Reaction time: 12 h. The product was purified by column chromatography on silica gel (0→2% ethyl acetate/Petroleum ether).

Pale yellow oil, 71.7 mg (94%).

^{1}H NMR (400 MHz, $CDCl_3$) δ 7.30 – 7.24 (m, 2H), 6.97 – 6.87 (m, 3H), 4.97 (t, *J* = 4.6 Hz, 1H), 4.13 (t, *J* = 6.8 Hz, 2H), 3.98 – 3.91 (m, 2H), 3.89 – 3.82 (m, 2H), 2.90 (t, *J* = 6.8 Hz, 2H), 2.78 – 2.68 (m, 2H), 1.99 (td, *J* = 7.7, 4.6 Hz, 2H).

^{13}C NMR (100 MHz, $CDCl_3$) δ 158.5, 129.5, 121.0, 114.6, 103.2, 67.7, 65.0, 34.3, 31.2, 27.0.

FT-IR (ATR) 2960, 2923, 2884, 1599, 1496, 753, 691 cm^{-1}.

HRMS (CI) m/z ($M+H^+$) calcd for $C_{13}H_{19}O_3S$: 255.1055, found: 255.1045.

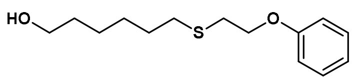

6-((2-phenoxyethyl)thio)hexan-1-ol (**5e**). The title compound was synthesized according to the General Procedure B, from *S*-(2-phenoxyethyl) benzenesulfonothioate (88.3 mg, 0.30 mmol) and 6-bromohexan-1-ol (59.8 mg, 44 μL, 0.33 mmol). Reaction time: 12 h. The product was purified by column chromatography on silica gel (0→20% ethyl acetate/Petroleum ether).
Pale yellow oil, 61.0 mg (80%).

^{1}H NMR (400 MHz, $CDCl_3$) δ 7.31 – 7.24 (m, 2H), 7.00 – 6.83 (m, 3H), 4.12 (t, *J* = 6.9 Hz, 2H), 3.61 (t, *J* = 6.6 Hz, 2H), 2.88 (t, *J* = 6.9 Hz, 2H), 2.61 (t, *J* = 7.4 Hz, 2H), 1.80 (s, 1H), 1.66 – 1.53 (m, 4H), 1.45 – 1.34 (m, 4H).

^{13}C NMR (100 MHz, $CDCl_3$) δ 158.5, 129.5, 121.0, 114.6, 67.8, 62.8, 32.7, 32.6, 31.0, 29.7, 28.6, 25.4.

FT-IR (ATR) 3342, 2926, 2856, 1599, 1240, 1015, 752, 690 cm^{-1}.

HRMS (CI) m/z (M^+) calcd for $C_{14}H_{22}O_2S$: 254.1341, found: 254.1339.

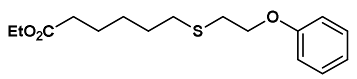

ethyl 6-((2-phenoxyethyl)thio)hexanoate (**5f**). The title compound was synthesized according to the General Procedure B, from *S*-(2-phenoxyethyl) benzenesulfonothioate (88.3 mg, 0.30 mmol) and ethyl 6-bromohexanoate (73.6 mg, 59 μL, 0.33 mmol). Reaction time: 12 h. The product was purified by column chromatography on silica gel (0→10% ethyl acetate/Petroleum ether).
Pale yellow oil, 74.3 mg (84%).

^{1}H NMR (400 MHz, $CDCl_3$) δ 7.30 – 7.24 (m, 2H), 6.94 (t, *J* = 7.4 Hz, 1H), 6.89 (d, *J* = 8.8 Hz, 2H), 4.12 (tt, *J* = 7.1, 3.8 Hz, 4H), 2.88 (t, *J* = 6.9 Hz, 2H), 2.62 (t, *J* = 7.4 Hz, 2H), 2.29 (t, *J* = 7.5 Hz, 2H), 1.63 (ddq, *J* = 10.7, 7.4, 3.4 Hz, 4H), 1.47 – 1.40 (m, 2H), 1.25 (t, *J* = 7.1 Hz, 3H).

^{13}C NMR (100 MHz, $CDCl_3$) δ 173.6, 158.5, 129.5, 121.0, 114.6, 67.8, 60.3, 34.2, 32.5, 31.0, 29.4, 28.3, 24.6, 14.3.

FT-IR (ATR) 2926, 1731, 1600, 1241, 1031, 753, 691 cm^{-1}.

HRMS (CI) m/z ($M+H^+$) calcd for $C_{16}H_{24}O_3S$: 296.1446, found: 296.1449.

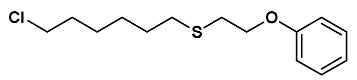

(6-chlorohexyl)(2-phenoxyethyl)sulfane (**5g**). The title compound was synthesized according to the General Procedure B, from *S*-(2-phenoxyethyl) benzenesulfonothioate (88.3 mg, 0.30 mmol) and 1-bromo-6-chlorohexane (65.8 mg, 50 μL, 0.33 mmol). Reaction time: 12 h. The product was purified by column chromatography on silica gel (Petroleum ether).
Pale yellow oil, 54.4 mg (66%).

^{1}H NMR (400 MHz, $CDCl_3$) δ 7.30 – 7.26 (m, 2H), 6.95 (t, *J* = 7.4 Hz, 1H), 6.89 (d, *J* = 8.1 Hz, 2H), 4.13 (t, *J* = 6.9 Hz, 2H), 3.52 (t, *J* = 6.7 Hz, 2H), 2.89 (t, *J* = 6.9 Hz, 2H), 2.62 (t, *J* = 7.4 Hz, 2H), 1.77 (p, *J* = 6.7 Hz, 2H), 1.66 – 1.61 (m, 2H), 1.44 (dq, *J* = 7.3, 4.0 Hz, 4H).

^{13}C NMR (100 MHz, $CDCl_3$) δ 158.5, 129.6, 121.1, 114.6, 67.8, 45.1, 32.7, 32.5, 31.1, 29.6, 28.1, 26.6.

FT-IR (ATR) 2925, 2857, 1600, 1496, 1241, 752, 691 cm^{-1}.

HRMS (CI) m/z (M^+) calcd for $C_{14}H_{21}ClOS$: 272.1002, found: 272.1014.

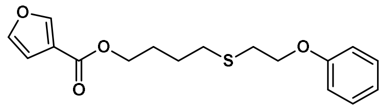

4-((2-phenoxyethyl)thio)butyl furan-3-carboxylate (**5h**). The title compound was synthesized according to the General Procedure B, from *S*-(2-phenoxyethyl) benzenesulfonothioate (88.3 mg, 0.30 mmol) and 4-bromobutyl furan-3-carboxylate (81.5 mg, 0.33 mmol). Reaction time: 12 h. The product was purified by column chromatography on silica gel (0→2% ethyl acetate/Petroleum ether).

Pale yellow oil, 53.5 mg (56%).

^{1}H NMR (400 MHz, $CDCl_3$) δ 8.00 (s, 1H), 7.41 (s, 1H), 7.30 – 7.25 (m, 2H), 6.95 (t, J = 7.4 Hz, 1H), 6.89 (d, J = 8.2 Hz, 2H), 6.73 (s, 1H), 4.27 (t, J = 6.3 Hz, 2H), 4.14 (t, J = 6.8 Hz, 2H), 2.90 (t, J = 6.8 Hz, 2H), 2.69 (t, J = 7.2 Hz, 2H), 1.88 – 1.81 (m, 2H), 1.76 (q, J = 8.7, 7.8 Hz, 2H).

^{13}C NMR (100 MHz, $CDCl_3$) δ 163.2, 158.5, 147.8, 143.8, 129.6, 121.1, 119.5, 114.6, 109.9, 67.8, 64.0, 32.4, 31.1, 27.9, 26.3.

FT-IR (ATR) 2955, 2922, 1718, 1496, 1305, 1240, 1158, 753, 691 cm^{-1}.

HRMS (CI) m/z (M^+) calcd for $C_{17}H_{20}O_2S$: 320.1082, found: 320.1079.

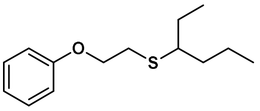

hexan-3-yl(2-phenoxyethyl)sulfane (**5i**). The title compound was synthesized according to the General Procedure B, from *S*-(2-phenoxyethyl) benzenesulfonothioate (88.3 mg, 0.30 mmol) and 3-bromohexane (148.6 mg, 132 μL, 0.90 mmol). Reaction temperature: 80 °C. Reaction time: 12 h. The product was purified by preparative TLC on silica gel (Petroleum ether).

Pale yellow oil, 49.3 mg (69%).

^{1}H NMR (400 MHz, $CDCl_3$) δ 7.31 – 7.24 (m, 2H), 6.97 – 6.87 (m, 3H), 4.10 (t, J = 7.2 Hz, 2H), 2.87 (t, J = 7.2 Hz, 2H), 2.65 (p, J = 6.4 Hz, 1H), 1.69 – 1.40 (m, 6H), 0.99 (t, J = 7.3 Hz, 3H), 0.92 (t, J = 7.1 Hz, 3H).

^{13}C NMR (100 MHz, $CDCl_3$) δ 158.6, 129.6, 121.0, 114.7, 68.0, 48.0, 36.8, 29.4, 27.7, 20.2, 14.2, 11.2.

FT-IR (ATR) 2958, 2927, 2871, 1600, 1496, 1239, 750, 690 cm^{-1}.

HRMS (CI) m/z ($M+H^+$) calcd for $C_{14}H_{23}OS$: 239.1470, found: 239.1475.

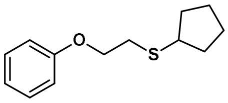

cyclopentyl(2-phenoxyethyl)sulfane (**5j**). The title compound was synthesized according to the General Procedure B, from S-cyclopentyl benzenesulfonothioate (72.7 mg, 0.30 mmol) and (2-bromoethoxy)benzene (60.3 mg, 0.33 mmol). Reaction time: 12 h. The product was purified by column chromatography on silica gel (Petroleum ether).

Pale yellow oil, 60.4 mg (90%).

^{1}H NMR (400 MHz, $CDCl_3$) δ 7.30 – 7.24 (m, 2H), 6.97 – 6.87 (m, 3H), 4.13 (t, *J* = 7.1 Hz, 2H), 3.20 (p, *J* = 6.9 Hz, 1H), 2.92 (t, *J* = 7.1 Hz, 2H), 2.01 (dt, *J* = 12.0, 6.3 Hz, 2H), 1.74 (td, *J* = 9.4, 8.1, 4.8 Hz, 2H), 1.55 (dtd, *J* = 13.2, 6.8, 3.8 Hz, 4H).

^{13}C NMR (100 MHz, $CDCl_3$) δ 158.6, 129.6, 121.0, 114.6, 67.8, 44.4, 34.1, 30.9, 24.9.

FT-IR (ATR) 2956, 2868, 1600, 1496, 1240, 751, 690 cm^{-1}.

HRMS (CI) m/z ($M+H^+$) calcd for $C_{13}H_{19}OS$: 223.1157, found: 223.1151.

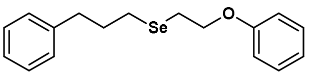

(2-phenoxyethyl)(3-phenylpropyl)selane (**5k**). The title compound was synthesized according to the General Procedure C, from *Se*-(2-phenoxyethyl) benzenesulfonoselenoate (102.4 mg, 0.30 mmol) and (3-bromopropyl)benzene (65.7 mg, 51 μL, 0.33 mmol). Reaction time: 12 h. The product was purified by column chromatography on silica gel (0→1% ethyl acetate/Petroleum ether).

Pale yellow oil, 76.0 mg (79%).

^{1}H NMR (400 MHz, $CDCl_3$) δ 7.30 – 7.24 (m, 4H), 7.20 – 7.15 (m, 3H), 6.98 – 6.91 (m, 1H), 6.89 – 6.83 (m, 2H), 4.17 (t, J = 7.2 Hz, 2H), 2.88 (t, J = 7.1 Hz, 2H), 2.74 – 2.69 (m, 2H), 2.66 (t, J = 7.4 Hz, 2H), 2.01 (p, J = 7.4 Hz, 2H).

^{13}C NMR (100 MHz, $CDCl_3$) δ 158.5, 141.4, 129.6, 128.6, 128.5, 126.1, 121.0, 114.7, 68.4, 35.9, 32.3, 24.1, 22.1.

FT-IR (ATR) 2927, 2854, 1598, 1494, 1237, 748, 690 cm^{-1}.

HRMS (CI) m/z (M^+) calcd for $C_{17}H_{20}OSe$: 320.0679, found: 320.0686.

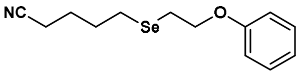

5-((2-phenoxyethyl)selanyl)pentanenitrile (**5l**). The title compound was synthesized according to the General Procedure C, from *Se*-(2-phenoxyethyl) benzenesulfonoselenoate (102.4 mg, 0.30 mmol) and 5-bromopentanenitrile (53.5 mg, 39 μL, 0.33 mmol). Reaction time: 12 h. The product was purified by column chromatography on silica gel (0→10% ethyl acetate/Petroleum ether).

Pale yellow oil, 73.3 mg (86%).

^{1}H NMR (400 MHz, $CDCl_3$) δ 7.32 – 7.24 (m, 2H), 7.00 – 6.81 (m, 3H), 4.20 (t, J = 6.9 Hz, 2H), 2.90 (t, J = 6.9 Hz, 2H), 2.69 (t, J = 7.0 Hz, 2H), 2.34 (t, J = 6.9 Hz, 2H), 1.88 – 1.71 (m, 4H).

^{13}C NMR (100 MHz, $CDCl_3$) δ 158.4, 129.6, 121.1, 119.4, 114.6, 68.4, 29.4, 25.4, 23.3, 22.3, 16.8.

FT-IR (ATR) 2932, 2865, 2245, 1598, 1494, 1235, 752, 691 cm^{-1}.

HRMS (CI) m/z (M^+) calcd for $C_{13}H_{17}NOS$: 283.0475, found: 283.0481.

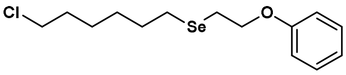

(6-chlorohexyl)(2-phenoxyethyl)selane (**5m**). The title compound was synthesized according to the General Procedure C, from *Se*-(2-phenoxyethyl) benzenesulfonoselenoate (102.4 mg, 0.30 mmol) and 1-bromo-6-chlorohexane (65.8 mg, 50 μL, 0.33 mmol). Reaction time: 12 h. The product was purified by column chromatography on silica gel (0→1% ethyl acetate/Petroleum ether).

Pale yellow oil, 76.9 mg (80%).

^{1}H NMR (400 MHz, $CDCl_3$) δ 7.32 – 7.24 (m, 2H), 6.99 – 6.85 (m, 3H), 4.19 (t, J = 7.2 Hz, 2H), 3.53 (d, J = 6.7 Hz, 2H), 2.89 (t, J = 7.2 Hz, 2H), 2.66 (t, J = 7.4 Hz, 2H), 1.80 – 1.67 (m, 4H), 1.44 (dh, J = 10.8, 5.5, 4.6 Hz, 4H).

^{13}C NMR (100 MHz, $CDCl_3$) δ 158.5, 129.6, 121.0, 114.6, 68.4, 45.1, 32.5, 30.5, 29.2, 26.5, 24.6, 22.1.

FT-IR (ATR) 2928, 2855, 1598, 1495, 1238, 751, 690 cm^{-1}.

HRMS (CI) m/z (M^+) calcd for $C_{14}H_{21}ClOSe$: 320.0446, found: 320.0456.

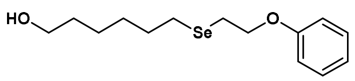

6-((2-phenoxyethyl)selanyl)hexan-1-ol (**5n**). The title compound was synthesized according to the General Procedure C, from *Se*-(2-phenoxyethyl) benzenesulfonoselenoate (102.4 mg, 0.30 mmol) and 6-bromohexan-1-ol (59.8 mg, 44 μL, 0.33 mmol). Reaction time: 12 h. The product was purified by column chromatography on silica gel (0→20% ethyl acetate/Petroleum ether).

Pale yellow oil, 69.5 mg (77%).

^{1}**H NMR** (400 MHz, $CDCl_3$) δ 7.31 – 7.25 (m, 2H), 6.99 – 6.92 (m, 1H), 6.92 – 6.87 (m, 2H), 4.19 (t, J = 7.2 Hz, 2H), 3.62 (t, J = 6.6 Hz, 2H), 2.89 (t, J = 7.2 Hz, 2H), 2.67 (t, J = 7.5 Hz, 2H), 1.70 (p, J = 7.1 Hz, 2H), 1.56 (p, J = 6.8 Hz, 3H), 1.41 (qq, J = 9.2, 5.0 Hz, 4H).

^{13}**C NMR** (100 MHz, $CDCl_3$) δ 158.5, 129.6, 121.0, 114.7, 68.4, 62.9, 32.7, 30.7, 29.7, 25.4, 24.7, 22.1.

FT-IR (ATR) 3357, 2927, 2854, 1598, 1495, 1237, 751, 690 cm^{-1}.

HRMS (CI) m/z (M^+) calcd for $C_{14}H_{22}O_2Se$: 302.0785, found: 302.0791.

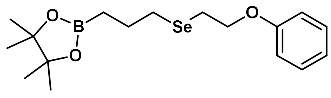

4,4,5,5-tetramethyl-2-(3-((2-phenoxyethyl)selanyl)propyl)-1,3,2-dioxaborolane (**5o**). The title compound was synthesized according to the General Procedure C, from *Se*-(2-phenoxyethyl) benzenesulfonoselenoate (102.4 mg, 0.30 mmol) and 2-(3-bromopropyl)-4,4,5,5-tetramethyl-1,3,2-dioxaborolane (82.2 mg, 64 μL, 0.33 mmol). Reaction time: 12 h. The product was purified by column chromatography on silica gel (0→10% ethyl acetate/Petroleum ether).

Pale yellow oil, 81.1 mg (73%).

^{1}**H NMR** (400 MHz, $CDCl_3$) δ 7.30 – 7.24 (m, 2H), 7.01 – 6.84 (m, 3H), 4.18 (t, J = 7.4 Hz, 2H), 2.89 (t, J = 7.3 Hz, 2H), 2.72 – 2.60 (m, 2H), 1.81 (p, J = 7.6 Hz, 2H), 1.23 (s, 12H), 0.90 (t, J = 7.7 Hz, 2H).

^{13}**C NMR** (100 MHz, $CDCl_3$) δ 158.5, 129.5, 120.9, 114.7, 83.1, 68.4, 27.2, 25.4, 24.9, 21.9. (The carbon directly attached to the boron atom was not observed due to quadrupolar relaxation.)

FT-IR (ATR) 2976, 2929, 1599, 1496, 1370, 1142, 751, 690 cm^{-1}.

HRMS (CI) m/z (M^+) calcd for $C_{17}H_{27}BO_3Se$: 370.1218, found: 370.1230.

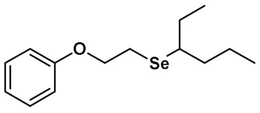

hexan-3-yl(2-phenoxyethyl)selane (**5p**). The title compound was synthesized according to the General Procedure C, from *Se*-(2-phenoxyethyl) benzenesulfonoselenoate (102.4 mg, 0.30 mmol) and 3-bromohexane (148.6 mg, 132 μL, 0.90 mmol). Reaction time: 12 h. The product was purified by preparative TLC on silica gel (Petroleum ether).

Pale yellow oil, 51.1 mg (60%).

^{1}**H NMR** (400 MHz, $CDCl_3$) δ 7.31 – 7.25 (m, 2H), 6.98 – 6.87 (m, 3H), 4.17 (t, J = 7.5 Hz, 2H), 2.94 – 2.83 (m, 3H), 1.74 – 1.57 (m, 4H), 1.53 – 1.40 (m, 2H), 1.01 (t, J = 7.3 Hz, 3H), 0.92 (t, J = 7.3 Hz, 3H).

^{13}**C NMR** (100 MHz, $CDCl_3$) 158.6, 129.6, 121.0, 114.7, 68.6, 44.5, 37.8, 28.8, 21.0, 20.9, 14.1, 12.2.

FT-IR (ATR) 2924, 2855, 1242, 743, 698 cm^{-1}.

HRMS (CI) m/z (M^+) calcd for $C_{14}H_{22}OSe$: 286.0836, found: 286.0846.

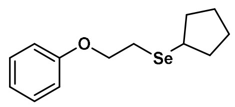

cyclopentyl(2-phenoxyethyl)selane (**5q**). The title compound was synthesized according to the General Procedure C, from *Se*-(2-phenoxyethyl) benzenesulfonoselenoate (102.4 mg, 0.30 mmol) and bromocyclopentane (134.1 mg, 96 μL, 0.90 mmol). Reaction time: 12 h. The product was purified by column chromatography on silica gel (Petroleum ether).

Pale yellow oil, 64.3 mg (79%).

^{1}H NMR (400 MHz, $CDCl_3$) δ 7.32 – 7.24 (m, 2H), 6.99 – 6.86 (m, 3H), 4.20 (t, J = 7.4 Hz, 2H), 3.35 (p, J = 6.9 Hz, 1H), 2.92 (t, J = 7.4 Hz, 2H), 2.12 – 2.03 (m, 2H), 1.75 (dtd, J = 11.4, 5.7, 2.9 Hz, 2H), 1.68 – 1.56 (m, 4H).

^{13}C NMR (100 MHz, $CDCl_3$) 158.5, 129.6, 121.0, 114.7, 68.5, 38.2, 34.7, 25.0, 22.1.

FT-IR (ATR) 2951, 2865, 1598, 1494, 1237, 749, 689 cm^{-1}.

HRMS (CI) m/z (M^+) calcd for $C_{13}H_{18}OSe$: 270.0523, found: 270.0536.

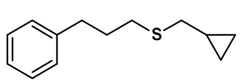

(cyclopropylmethyl)(3-phenylpropyl)sulfane (**5r**). The title compound was synthesized according to the General Procedure B, from *S*-(cyclopropylmethyl) benzenesulfonothioate (68.5 mg, 0.30 mmol) and (3-bromopropyl)benzene (65.7 mg, 51 μL, 0.33 mmol). Reaction time: 12 h. The product was purified by column chromatography on silica gel (Petroleum ether).

Pale yellow oil, 50.3 mg (81%).

^{1}H NMR (400 MHz, $CDCl_3$) δ 7.29 – 7.25 (m, 2H), 7.20 – 7.16 (m, 3H), 2.72 (t, *J* = 7.6 Hz, 2H), 2.59 (t, *J* = 7.3 Hz, 2H), 2.44 (d, *J* = 7.0 Hz, 2H), 1.92 (q, *J* = 7.7 Hz, 2H), 0.95 (dddd, *J* = 11.8, 8.0, 4.7, 1.8 Hz, 1H), 0.58 – 0.51 (m, 2H), 0.18 (dt, *J* = 6.2, 4.6 Hz, 2H).

^{13}C NMR (100 MHz, $CDCl_3$) 141.7, 128.6, 128.4, 126.0, 37.5, 35.0, 31.5, 31.4, 11.4, 5.4.

FT-IR (ATR) 2924, 2855, 1242, 743, 698 cm^{-1}.

HRMS (CI) m/z ($M+H^+$) calcd for $C_{13}H_{19}S$: 207.1207, found: 207.1199.

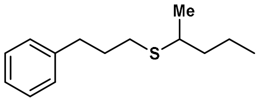

pentan-2-yl(3-phenylpropyl)sulfane (**5s**). The title compound was synthesized according to the General Procedure B, from *S*-(pentan-2-yl) benzenesulfonothioate (73.3 mg, 0.30 mmol) and (3-bromopropyl)benzene (65.7 mg, 51 μL, 0.33 mmol). Reaction time: 16 h. The product was purified by column chromatography on silica gel (Petroleum ether).

Pale yellow oil, 50.3 mg (81%).

^{1}H NMR (400 MHz, $CDCl_3$) δ 7.27 (t, J = 7.3 Hz, 2H), 7.18 (d, J = 7.3 Hz, 3H), 2.72 (t, J = 7.5 Hz, 2H), 2.53 (t, J = 7.4 Hz, 2H), 1.90 (p, J = 7.5 Hz, 2H), 1.57 – 1.38 (m, 4H), 1.24 (d, J = 7.5 Hz, 4H), 0.92 (dt, J = 13.4, 6.8 Hz, 3H).

^{13}C NMR (100 MHz, $CDCl_3$) 141.7, 128.6, 128.4, 126.0, 37.5, 35.0, 31.5, 31.4, 11.4, 5.4.

FT-IR (ATR) 2958, 2924, 1454, 1048, 743, 699 cm^{-1}.

HRMS (CI) m/z ($M+H^+$) calcd for $C_{14}H_{23}S$: 223.1520, found: 223.1522.

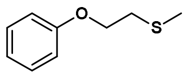

methyl(2-phenoxyethyl)sulfane (**5t**). The title compound was synthesized according to the General Procedure B, from *S*-methyl benzenesulfonothioate (56.5 mg, 0.30 mmol) and (2-bromoethoxy)benzene (60.3 mg, 0.33 mmol). Reaction time: 12 h. The product was purified by column chromatography on silica gel (Petroleum ether).

Pale yellow oil, 43.6 mg (86%).

^{1}**H NMR** (400 MHz, $CDCl_3$) δ 7.30 – 7.25 (m, 2H), 6.97 – 6.88 (m, 3H), 4.15 (t, J = 6.8 Hz, 2H), 2.87 (t, J = 6.8 Hz, 2H), 2.20 (s, 3H).

^{13}**C NMR** (100 MHz, $CDCl_3$) δ 158.6, 129.6, 121.1, 114.7, 67.4, 33.2, 16.3.

FT-IR (ATR) 2920, 1599, 1495, 1239, 1032, 751, 690 cm^{-1}.

HRMS (CI) m/z ($M+H^+$) calcd for $C_9H_{13}OS$: 169.0687, found: 169.0680.

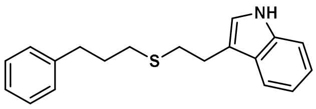

3-(2-((2-phenoxyethyl)thio)ethyl)-1H-indole (**5u**). The title compound was synthesized according to the General Procedure B, from *S*-(2-(1H-indol-3-yl)ethyl) benzenesulfonothioate (95.2 mg, 0.30 mmol) and (3-bromopropyl)benzene (65.7 mg, 51 μL, 0.33 mmol). Reaction time: 12 h. The product was purified by column chromatography on silica gel (0→5% ethyl acetate/Petroleum ether).

Pale yellow oil, 64.7 mg (73%).

^{1}**H NMR** (400 MHz, $CDCl_3$) δ 7.88 (s, 1H), 7.56 (d, J = 7.9 Hz, 1H), 7.27 (dd, J = 13.0, 7.8 Hz, 3H), 7.20 – 7.09 (m, 5H), 6.94 (d, J = 2.4 Hz, 1H), 3.07 – 2.96 (m, 2H), 2.88 – 2.79 (m, 2H), 2.69 (t, J = 7.6 Hz, 2H), 2.58 – 2.51 (m, 2H), 1.90 (p, J = 7.4 Hz, 2H).

^{13}**C NMR** (100 MHz, $CDCl_3$) δ 141.7, 136.3, 128.6, 128.5, 127.2, 126.0, 122.1, 121.7, 119.4, 118.7, 115.0, 111.3, 34.9, 32.8, 31.7, 31.3, 26.1.

FT-IR (ATR) 3417, 2918, 2850, 1455, 738, 698 cm^{-1}.

HRMS (CI) m/z (M^+) calcd for $C_{19}H_{21}NS$: 295.1395, found: 295.1397.

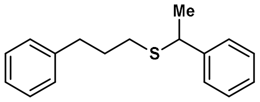

(1-phenylethyl)(3-phenylpropyl)sulfane (**5v**). The title compound was synthesized according to the General Procedure B, from *S*-(1-phenylethyl) benzenesulfonothioate (83.5 mg, 0.30 mmol) and (3-bromopropyl)benzene (65.7 mg, 51 μL, 0.33 mmol). Reaction temperature: 80 °C. Reaction time: 12 h. The product was purified by column chromatography on silica gel (Petroleum ether).

Pale yellow oil, 50.1 mg (75%).

^{1}**H NMR** (400 MHz, $CDCl_3$) δ 7.31 – 7.08 (m, 10H), 3.92 (q, J = 7.0 Hz, 1H), 2.60 (hept, J = 7.6, 7.0 Hz, 2H), 2.38 – 2.22 (m, 2H), 1.80 (q, J = 7.8 Hz, 2H), 1.55 (d, J = 7.0 Hz, 3H).

^{13}**C NMR** (100 MHz, $CDCl_3$) δ 144.2, 141.7, 128.5, 128.4, 127.3, 127.1, 125.9, 44.1, 34.9, 31.1, 30.8, 22.7.

FT-IR (ATR) 2923, 2854, 1492, 1452, 744, 696 cm^{-1}.

HRMS (CI) m/z (M^+) calcd for $C_{17}H_{20}S$: 256.1286, found: 256.1287.

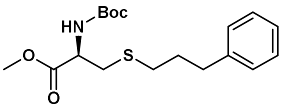

methyl N-(tert-butoxycarbonyl)-S-(3-phenylpropyl)-L-cysteinate (**5w**). The title compound was synthesized according to the General Procedure B, from methyl (R)-3-bromo-2-((tert-butoxycarbonyl)amino)propanoate (84.6 mg, 0.30 mmol) and *S*-(3-phenylpropyl) benzenesulfonothioate (96.5 mg, 0.33 mmol). Reaction temperature: 80 ºC. Reaction time: 16 h. The product was purified by column chromatography on silica gel (0→20% ethyl acetate/Petroleum ether).

Pale yellow oil, 75.4 mg (71%).

^{1}H NMR (400 MHz, $CDCl_3$) δ 7.29 – 7.25 (m, 2H), 7.20 – 7.15 (m, 3H), 5.52 – 5.24 (m, 1H), 4.52 (q, *J* = 5.6 Hz, 1H), 3.72 (s, 3H), 2.95 (dd, *J* = 5.4, 2.6 Hz, 2H), 2.69 (t, *J* = 7.6 Hz, 2H), 2.52 (t, *J* = 7.3 Hz, 2H), 1.88 (t, *J* = 7.5 Hz, 2H), 1.44 (s, 9H).

^{13}C NMR (100 MHz, $CDCl_3$) δ 171.6, 155.2, 141.3, 128.5, 128.5, 126.0, 80.2, 53.3, 52.6, 34.7, 34.5, 32.1, 31.1, 28.4.

FT-IR (ATR) 3373, 2976, 2928, 1746, 1713, 1496, 1161, 1053, 744, 699 cm^{-1}.

HRMS (CI) m/z (M^+) calcd for $C_{18}H_{27}NO_4S$: 353.1661, found: 353.1675.

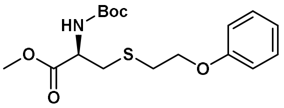

methyl N-(tert-butoxycarbonyl)-S-(2-phenoxyethyl)-L-cysteinate (**5x**). The title compound was synthesized according to the General Procedure B, from methyl (R)-3-bromo-2-((tert-butoxycarbonyl)amino)propanoate (97.2 mg, 0.30 mmol) and *S*-(2-phenoxyethyl) benzenesulfonothioate (96.5 mg, 0.33 mmol). Reaction temperature: 80 ºC. Reaction time: 16 h. The product was purified by column chromatography on silica gel (0→20% ethyl acetate/Petroleum ether).

Pale yellow oil, 66.7 mg (62%).

^{1}H NMR (400 MHz, $CDCl_3$) δ 7.28 (td, *J* = 7.3, 2.3 Hz, 2H), 6.98 – 6.88 (m, 3H), 5.45 (d, *J* = 7.9 Hz, 1H), 4.71 – 4.47 (m, 1H), 4.13 (t, *J* = 6.5 Hz, 2H), 3.75 (s, 3H), 3.09 (qd, *J* = 13.8, 5.2 Hz, 2H), 2.92 (td, *J* = 6.5, 3.7 Hz, 2H), 1.44 (s, 9H).

^{13}C NMR (100 MHz, $CDCl_3$) δ 171.6, 158.4, 155.3, 129.6, 121.2, 114.6, 80.3, 67.8, 53.5, 52.6, 35.1, 31.7, 28.4, 28.3.

FT-IR (ATR) 3373, 2976, 2928, 1711, 1496, 1240, 1161, 1030, 753, 691 cm^{-1}.

HRMS (CI) m/z ($M+H^+$) calcd for $C_{17}H_{26}NO_5S$: 356.1532, found: 356.1537.

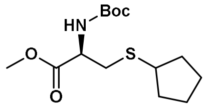

methyl N-(tert-butoxycarbonyl)-S-cyclopentyl-L-cysteinate (**5y**). The title compound was synthesized according to the General Procedure B, from methyl (R)-3-bromo-2-((tert-butoxycarbonyl)amino)propanoate (97.2 mg, 0.30 mmol) and *S*-cyclopentyl benzenesulfonothioate (80.0 mg, 0.33 mmol). Reaction temperature: 80 ºC. Reaction time: 16 h. The product was purified by column chromatography on silica gel (0→20% ethyl acetate/Petroleum ether).

Pale yellow oil, 57.7 mg (63%).

^{1}H NMR (400 MHz, $CDCl_3$) δ 5.35 (d, *J* = 7.7 Hz, 1H), 4.50 (q, *J* = 5.9 Hz, 1H), 3.73 (s, 3H), 3.10 – 3.05 (m, 1H), 2.95 (dd, *J* = 5.3, 2.9 Hz, 2H), 1.95 (q, *J* = 6.2, 5.7 Hz, 2H), 1.78 – 1.62 (m, 3H), 1.59 – 1.48 (m, 3H), 1.42 (s, 9H).

^{13}C NMR (100 MHz, $CDCl_3$) δ 171.8, 155.2, 80.1, 53.4, 52.6, 44.3, 34.1, 33.8, 33.8, 28.4, 24.8, 24.8.

FT-IR (ATR) 3364, 2956, 2869, 1747, 1714, 1498, 1161, 1051 cm^{-1}.

HRMS (CI) m/z (M^+) calcd for $C_{14}H_{25}NO_4S$: 303.1504, found: 303.1498.

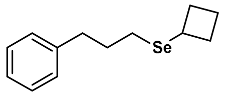

cyclobutyl(3-phenylpropyl)selane (**5z**). The title compound was synthesized according to the General Procedure C, from *Se*-cyclobutyl benzenesulfonoselenoate (82.6 mg, 0.30 mmol) and (3-bromopropyl)benzene (65.7 mg, 51 μL, 0.33 mmol). Reaction time: 12 h. The product was purified by column chromatography on silica gel (Petroleum ether).

Pale yellow oil, 50.2 mg (66%).

^{1}H NMR (400 MHz, $CDCl_3$) δ 7.27 (t, J = 7.5 Hz, 2H), 7.18 (dd, J = 7.2, 3.9 Hz, 3H), 3.67 (p, J = 8.0 Hz, 1H), 2.74 – 2.66 (m, 2H), 2.55 (t, J = 7.4 Hz, 2H), 2.38 (dtd, J = 11.2, 7.9, 3.1 Hz, 2H), 2.18 – 2.07 (m, 2H), 2.06 – 1.91 (m, 4H).

^{13}C NMR (100 MHz, $CDCl_3$) δ 141.7, 128.6, 128.4, 126.0, 36.1, 32.6, 32.6, 31.9, 22.9, 20.2.

FT-IR (ATR) 2961, 2931, 2854, 1496, 1453, 741, 698 cm^{-1}.

HRMS (CI) m/z ($M+H^+$) calcd for $C_{13}H_{19}Se$: 255.0646, found: 255.0651.

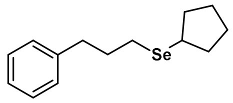

cyclopentyl(3-phenylpropyl)selane (**5aa**). The title compound was synthesized according to the General Procedure C, from *Se*-cyclopentyl benzenesulfonoselenoate (86.8 mg, 0.30 mmol) and (3-bromopropyl)benzene (65.7 mg, 51 μL, 0.33 mmol). Reaction time: 12 h. The product was purified by column chromatography on silica gel (Petroleum ether).

Pale yellow oil, 68.1 mg (85%).

^{1}H NMR (400 MHz, $CDCl_3$) δ 7.29 – 7.25 (m, 2H), 7.20 – 7.15 (m, 3H), 3.22 (p, J = 7.0 Hz, 1H), 2.74 – 2.68 (m, 2H), 2.58 (t, J = 7.4 Hz, 2H), 2.06 – 1.95 (m, 4H), 1.80 – 1.68 (m, 2H), 1.65 – 1.50 (m, 4H).

^{13}C NMR (100 MHz, $CDCl_3$) δ 141.7, 128.6, 128.4, 125.9, 37.5, 36.2, 34.6, 32.5, 25.0, 23.3.

FT-IR (ATR) 2953, 1932, 2857, 1452, 741, 698 cm^{-1}.

HRMS (CI) m/z ($M+H^+$) calcd for $C_{14}H_{21}Se$: 269.0803, found: 269.0801.

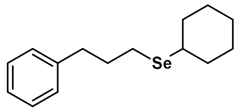

cyclopentyl(3-phenylpropyl)selane (**5ab**). The title compound was synthesized according to the General Procedure C, from *Se*-cyclohexyl benzenesulfonoselenoate (92.8 mg, 0.30 mmol) and (3-bromopropyl)benzene (65.7 mg, 51 μL, 0.33 mmol). Reaction time: 12 h. The product was purified by column chromatography on silica gel (Petroleum ether).

Pale yellow oil, 76.1 mg (90%).

^{1}**H NMR** (400 MHz, $CDCl_3$) δ 7.27 (dd, J = 8.1, 6.9 Hz, 2H), 7.22 – 7.13 (m, 3H), 2.88 (tt, J = 10.8, 3.7 Hz, 1H), 2.75 – 2.66 (m, 2H), 2.57 (t, J = 7.4 Hz, 2H), 1.98 (p, J = 7.5 Hz, 4H), 1.78 – 1.67 (m, 2H), 1.54 – 1.40 (m, 2H), 1.37 – 1.21 (m, 3H).

^{13}**C NMR** (100 MHz, $CDCl_3$) δ 141.7, 128.6, 128.4, 125.9, 38.7, 36.2, 34.8, 32.6, 27.0, 25.9, 21.9.

FT-IR (ATR) 2924, 2850, 1446, 740, 697 cm^{-1}.

HRMS (CI) m/z ($M+H^+$) calcd for $C_{15}H_{23}Se$: 283.0959, found: 283.0959.

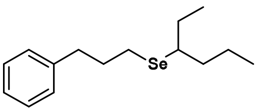

hexan-3-yl(3-phenylpropyl)selane (**5ac**). The title compound was synthesized according to the General Procedure C, from *Se*-(hexan-3-yl) benzenesulfonoselenoate (91.6 mg, 0.30 mmol) and (3-bromopropyl)benzene (65.7 mg, 51 µL, 0.33 mmol). Reaction time: 12 h. The product was purified by column chromatography on silica gel (Petroleum ether).

Pale yellow oil, 70.9 mg (83%).

^{1}**H NMR** (400 MHz, $CDCl_3$) δ 7.28 (dd, J = 8.0, 6.8 Hz, 2H), 7.22 – 7.15 (m, 3H), 2.80 – 2.67 (m, 3H), 2.54 (t, J = 7.4 Hz, 2H), 1.97 (p, J = 7.5 Hz, 2H), 1.71 – 1.53 (m, 4H), 1.52 – 1.33 (m, 2H), 0.98 (t, J = 7.3 Hz, 3H), 0.90 (t, J = 7.2 Hz, 3H).

^{13}**C NMR** (100 MHz, $CDCl_3$) δ 141.8, 128.6, 128.5, 126.0, 43.8, 37.8, 36.2, 32.6, 28.8, 22.3, 21.1, 14.1, 12.3.

FT-IR (ATR) 2957, 2927, 2870, 1454, 741, 697 cm^{-1}.

HRMS (CI) m/z ($M+H^+$) calcd for $C_{15}H_{25}Se$: 285.1121, found: 285.1125.

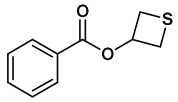

thietan-3-yl benzoate (**5ad**). The title compound was synthesized according to the General Procedure B, from methyl 1-bromo-3-((phenylsulfonyl)thio)propan-2-yl benzoate (124.6 mg, 0.30 mmol). Reaction concentration: 0.1 M. The product was purified by column chromatography on silica gel (0→20% ethyl acetate/Petroleum ether).

Pale yellow oil, 29.4 mg (50%).

^{1}**H NMR** (400 MHz, $CDCl_3$) δ 8.03 (d, J = 7.6 Hz, 2H), 7.58 (t, J = 7.3 Hz, 1H), 7.45 (t, J = 7.7 Hz, 2H), 5.86 (p, J = 7.9 Hz, 1H), 3.63 (td, J = 8.2, 2.0 Hz, 2H), 3.42 (td, J = 7.8, 1.9 Hz, 2H).

^{13}**C NMR** (100 MHz, $CDCl_3$) δ 165.2, 133.4, 129.8, 129.5, 128.5, 68.7, 35.2.

FT-IR (ATR) 2971, 2924, 1718, 1266, 1096, 710 cm^{-1}.

HRMS (CI) m/z ($M+H^+$) calcd for $C_{10}H_{11}O_2S$: 195.0480, found: 195.0486.

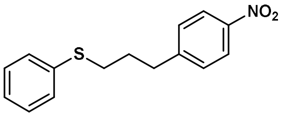

(3-(4-nitrophenyl)propyl)(phenyl)sulfane (**7a**). According to Liu's method[4], $Pd_2(dba)_3$ (4.6 mg, 0.005 mmol), NaO^tBu (72.1 mg, 0.75 mmol), Ruphos (4.7mg, 0.01 mmol) and 1-bromo-4-nitrobenzene (50.5 mg, 0.25 mmol) were added to a Schlenk tube equipped with a stir bar. The vessel was evacuated and filled with

argon (three cycles). Toluene (0.5 mL), H_2O (0.05 mL), and **3f** (83.5 mg, 0.30 mmol) were added in turn by syringe under an argon atmosphere. The resulting reaction mixture was stirred vigorously at 80 ºC for 24 h. The reaction mixture was then diluted with EtOAc, filtered through Celite with copious washings (EtOAc) and concentrated. The product was purified by column chromatography on silica gel (0→2% ethyl acetate/Petroleum ether).

Pale yellow solid, 49.3 mg (72%).

^{1}H NMR (400 MHz, $CDCl_3$) δ 8.13 (d, J = 8.5 Hz, 2H), 7.34 – 7.26 (m, 6H), 7.22 – 7.17 (m, 1H), 2.92 (t, J = 7.1 Hz, 2H), 2.87 (t, J = 7.6 Hz, 2H), 1.98 (p, J = 7.3 Hz, 2H).

^{13}C NMR (100 MHz, $CDCl_3$) δ 149.3, 146.6, 136.0, 129.6, 129.4, 129.1, 126.3, 123.8, 34.5, 33.0, 30.2.

FT-IR (ATR) 2955, 2923, 2856, 1509, 1340, 735, 689 cm^{-1}.

HRMS (CI) m/z ($M+H^+$) calcd for $C_{15}H_{16}NO_2S$: 274.0902, found: 274.0900.

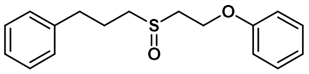

(S)-(3-((2-phenoxyethyl)sulfinyl)propyl)benzene (**7b**). According to Bahrami's method[5], in a round-bottomed flask (10 mL) equipped with a stir bar, a solution of **5a** (136.2 mg, 0.50 mmol) in CH_3CN (2.5 mL) was prepared. Aqueous 30% H_2O_2 (1 mmol, 0.1 mL) and Me_3SiCl (0.50 mmol, 44μL) were added and the mixture was stirred at 25 ºC for 10 min. After disappearance of the sulfide, the reaction mixture was quenched by adding H_2O (20 mL), extracted with EtOAc (3 × 10 mL). The organic layer was dried over Na_2SO_4, filtered, and concentrated. The product was purified by column chromatography on silica gel (20→100% ethyl acetate/Petroleum ether).

White solid, 120.7 mg (83%).

^{1}H NMR (400 MHz, $CDCl_3$) δ 7.26 (dt, J = 7.9, 4.2 Hz, 4H), 7.17 (t, J = 6.4 Hz, 3H), 6.96 (t, J = 7.4 Hz, 1H), 6.86 (d, J = 8.1 Hz, 2H), 4.34 (dq, J = 10.3, 5.6, 5.1 Hz, 2H), 3.09 (ddd, J = 13.5, 8.3, 5.2 Hz, 1H), 2.96 (dt, J = 13.6, 4.5 Hz, 1H), 2.75 (dt, J = 15.8, 7.9 Hz, 4H), 2.12 (dtd, J = 14.9, 10.8, 9.0, 5.1 Hz, 2H).

^{13}C NMR (100 MHz, $CDCl_3$) δ 157.8, 140.3, 129.5, 128.5, 128.4, 126.2, 121.4, 114.5, 60.3, 52.0, 51.7, 34.6, 24.2.

FT-IR (ATR) 2958, 2928, 1588, 1498, 1456, 1043, 743, 693 cm^{-1}.

HRMS (CI) m/z ($M+H^+$) calcd for $C_{17}H_{21}O_2S$: 289.1262, found: 289.1253.

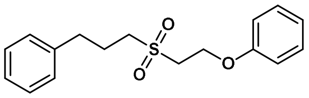

(3-((2-phenoxyethyl)sulfonyl)propyl)benzene (**7c**). In a round-bottomed flask (10 mL) equipped with a stir bar, a solution of **5a** (136.2 mg, 0.50 mmol) in CH_2Cl_2 (2.0 mL) was prepared. The solution was cooled to 0 ºC. A solution of *m*-CPBA (purity: 85%, 406.0 mg, 2.0 mmol) in CH_2Cl_2 (10.0 mL) was added dropwise and the mixture was stirred at 25 ºC. After disappearance of the sulfide, the reaction mixture was quenched by adding H_2O (20 mL), extracted with EtOAc (3 × 10 mL). The organic layer was dried over Na_2SO_4, filtered, and concentrated. The product was purified by column chromatography on silica gel (10→20% ethyl acetate/Petroleum ether).

White solid, 136.6 mg (89%).

^{1}**H NMR** (400 MHz, $CDCl_3$) δ 7.30 – 7.14 (m, 7H), 6.98 (t, J = 7.4 Hz, 1H), 6.74 (d, J = 8.2 Hz, 2H), 4.32 (t, J = 5.3 Hz, 2H), 3.32 (t, J = 5.3 Hz, 2H), 3.14 – 3.04 (m, 2H), 2.75 (t, J = 7.3 Hz, 2H), 2.24 – 2.14 (m, 2H).

^{13}**C NMR** (100 MHz, $CDCl_3$) δ 157.4, 139.9, 129.7, 128.6, 128.5, 126.4, 121.8, 114.3, 61.7, 54.2, 52.7, 34.3, 23.4.

FT-IR (ATR) 2977, 2931, 1588, 1127, 743, 688 cm^{-1}.

HRMS (CI) m/z ($M+H^+$) calcd for $C_{17}H_{21}O_3S$: 305.1211, found: 305.1203.

Kinetic Analysis Radical clock experiment

Supplementary Table 10. Order with respect to [$NiCl_2(PPh_3)_2$]

[$NiCl_2(PPh_3)_2$] / mol %	Δ[**3a**] Δt^{-1} / 10^{-4} s^{-1}	log(*c* / mol L^{-1})	log(Δ[**3a**] Δt^{-1} / s^{-1})
4.0	1.425	-2.176	-3.846
5.0	2.008	-2.079	-3.697
6.0	2.452	-2.000	-3.611
7.5	2.990	-1.903	-3.524

The reaction order with respect to [$NiCl_2(PPh_3)_2$] was examined using the initial rate method[6]. Under an atmosphere of nitrogen a mixture of [$NiCl_2(PPh_3)_2$] (4.0, 5.0, 6.0, 7.5 mol %), 2,2'-bipyridin (**L1**) (5.9 mg, 7.5 mol %) and manganese (41 mg, 0.75 mmol) in DMF (1.0 mL) was stirred at ambient temperature for 10 min. 1-Bromo-3-phenylpropane (**1a**) (100 mg, 0.50 mmol), *S*-phenyl benzenesulfonothioate (**2a**) (138 mg, 0.55 mmol) and DMF (2.0 mL) were added and the mixture was stirred at 30 °C for 5 h. Over the course of the reaction an *in situ* IR spectrum was acquired every minute. The initial rate was determined from the increase of the peak at 699 cm^{-1}. The absolute peak area was measured from 704 to 693 cm^{-1} with a two-point baseline at 704 and 693 cm^{-1}.

Supplementary Table 11. Order with respect to 1a

**1a** / mmol	Δ[**3a**] Δt^{-1} / 10^{-4} s^{-1}	log(*c* / mol L^{-1})	log(Δ[**3a**] Δt^{-1} / s^{-1})
0.40	2.015	-0.875	-3.696
0.45	2.042	-0.824	-3.690
0.50	2.008	-0.778	-3.697
0.60	1.988	-0.699	-3.702

The reaction order with respect to 1-Bromo-3-phenylpropane (**1a**) was examined using the initial rate method[6]. Under an atmosphere of nitrogen a mixture of [$NiCl_2(PPh_3)_2$] (16.4 mg, 25 μmol), 2,2'-bipyridin (**L1**) (5.9 mg, 38 μmol) and manganese (41 mg, 0.75 mmol) in DMF (1.0 mL) was stirred at ambient temperature for 10 min. 1-Bromo-3-phenylpropane (**1a**) (0.40, 0.45, 0.50, 0.60 mmol), *S*-phenyl benzenesulfonothioate (**2a**) (138 mg, 0.55 mmol) and DMF (2.0 mL) were added and the mixture was stirred at 30 °C for 5 h. Over the course of

the reaction an *in situ* IR spectrum was acquired every minute. The initial rate was determined from the increase of the peak at 699 cm^{-1}. The absolute peak area was measured from 704 to 693 cm^{-1} with a two-point baseline at 704 and 693 cm^{-1}.

Supplementary Table 12. Order with respect to 2a

**2a** / mmol	Δ[**3a**] Δt^{-1} / 10^{-4} s^{-1}	log(*c* / mol L^{-1})	log(Δ[**3a**] Δt^{-1} / s^{-1})
0.40	1.980	-0.875	-3.703
0.45	2.038	-0.824	-3.691
0.50	2.006	-0.778	-3.698
0.55	2.008	-0.737	-3.697

The reaction order with respect to *S*-phenyl benzenesulfonothioate (**2a**) was examined using the initial rate method[6]. Under an atmosphere of nitrogen a mixture of [$NiCl_2(PPh_3)_2$] (16.4 mg, 25 µmol), 2,2'-bipyridin (**L1**) (5.9 mg, 38 µmol) and manganese (41 mg, 0.75 mmol) in DMF (1.0 mL) was stirred at ambient temperature for 10 min. 1-Bromo-3-phenylpropane (**1a**) (100 mg, 0.50 mmol), *S*-phenyl benzenesulfonothioate (**2a**) (0.40, 0.45, 0.50, 0.55 mmol) and DMF (2.0 mL) were added and the mixture was stirred at 30 °C for 5 h. Over the course of the reaction an *in situ* IR spectrum was acquired every minute. The initial rate was determined from the increase of the peak at 699 cm^{-1}. The absolute peak area was measured from 704 to 693 cm^{-1} with a two-point baseline at 704 and 693 cm^{-1}.

Supplementary Table 13. Ratio of linear/cyclic products with respect to catalyst concentration.

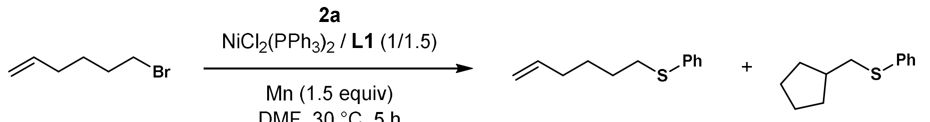

1e 3e 3e'

Entry	[$NiCl_2(PPh_3)_2$]	Yield / %	3e:3e'
1	5.0 mol %	83	3.5:1
2	7.5 mol %	76	9.5:1
3	10.0 mol %	79	>30:1

Under an atmosphere of nitrogen a mixture of [$NiCl_2(PPh_3)_2$] (5.0, 7.5 or 10.0 mol %), 2,2'-bipyridin (**L1**) (7.5, 11.3 or 15.0 mol %) and manganese (41 mg, 0.75 mmol) in DMF (1.0 mL) was stirred at ambient temperature for 10 min. 1-Bromohex-5-ene (**1e**) (82 mg, 0.50 mmol), *S*-phenyl benzenesulfonothioate (**2a**) (138 mg, 0.55 mmol) and DMF (1.5 mL) were added and the mixture was stirred at 30 °C for 5 h. Afterwards EtOAc (100 mL) was added, the mixture was washed with water (3 x 20 mL), dried over Na_2SO_4 and the solvent was removed. Purification of the residue by column chromatography on silica gel (*n*-hexane) yielded **3e** and **3e'** as a mixture. The **3e**:**3e'** ratio was determined by ^{1}H-NMR spectroscopy.

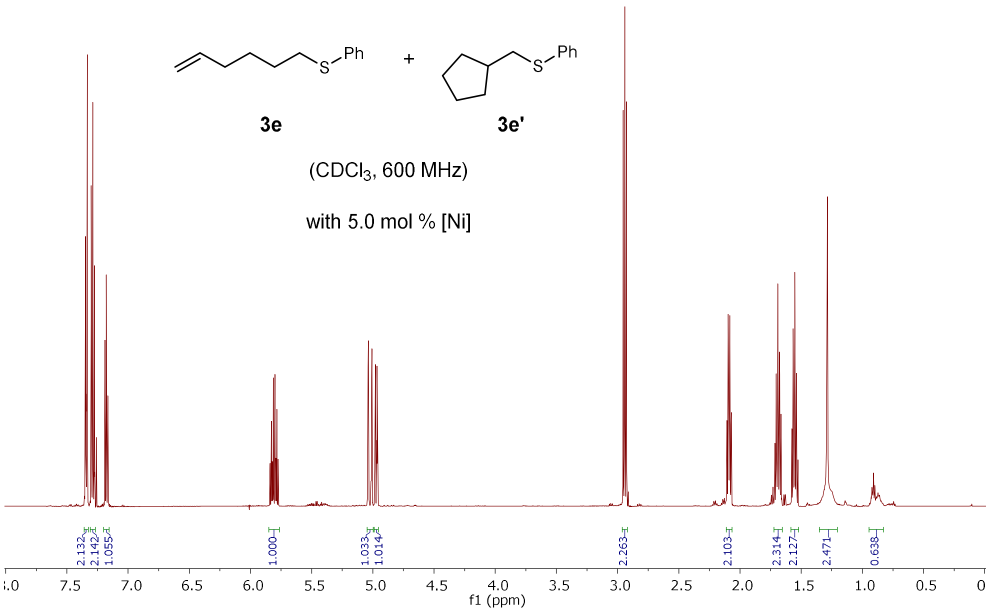

Supplementary Figure 1. NMR spectra for mixture of **3e** and **3e'** with 5.0 % [Ni].

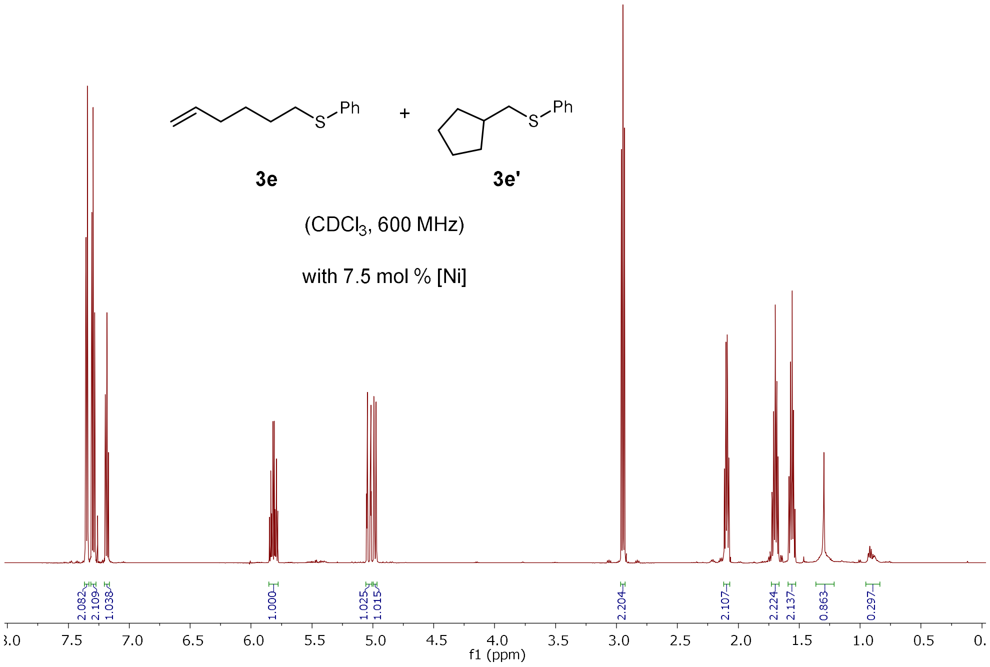

Supplementary Figure 2. NMR spectra for mixture of **3e** and **3e'** with 7.5 % [Ni].

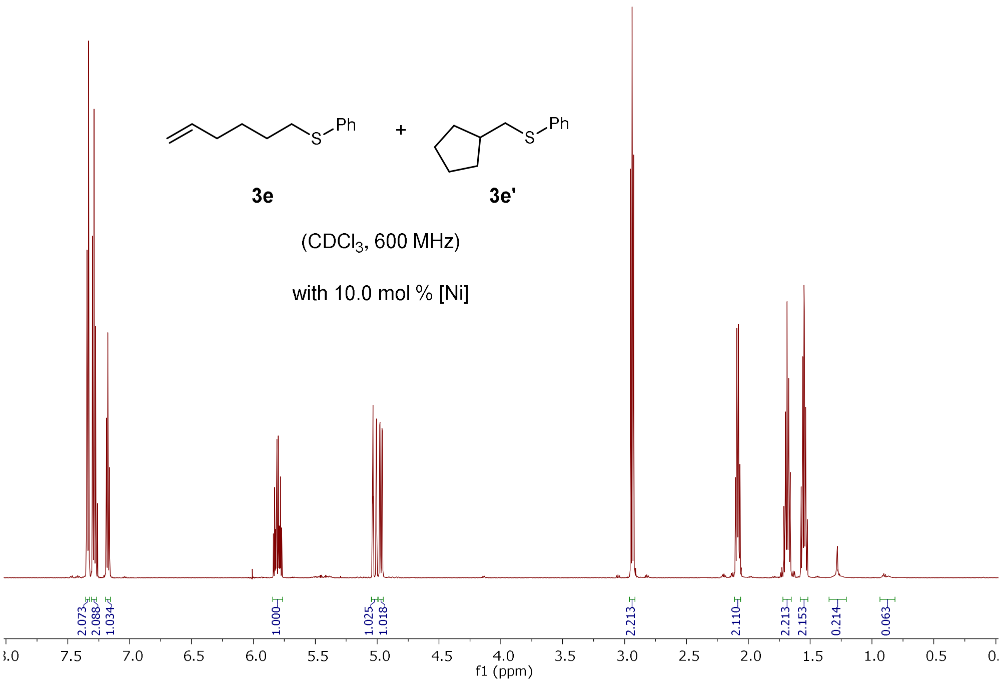

Supplementary Figure 3. NMR spectra for mixture of **3e** and **3e'** with 10.0 % [Ni].

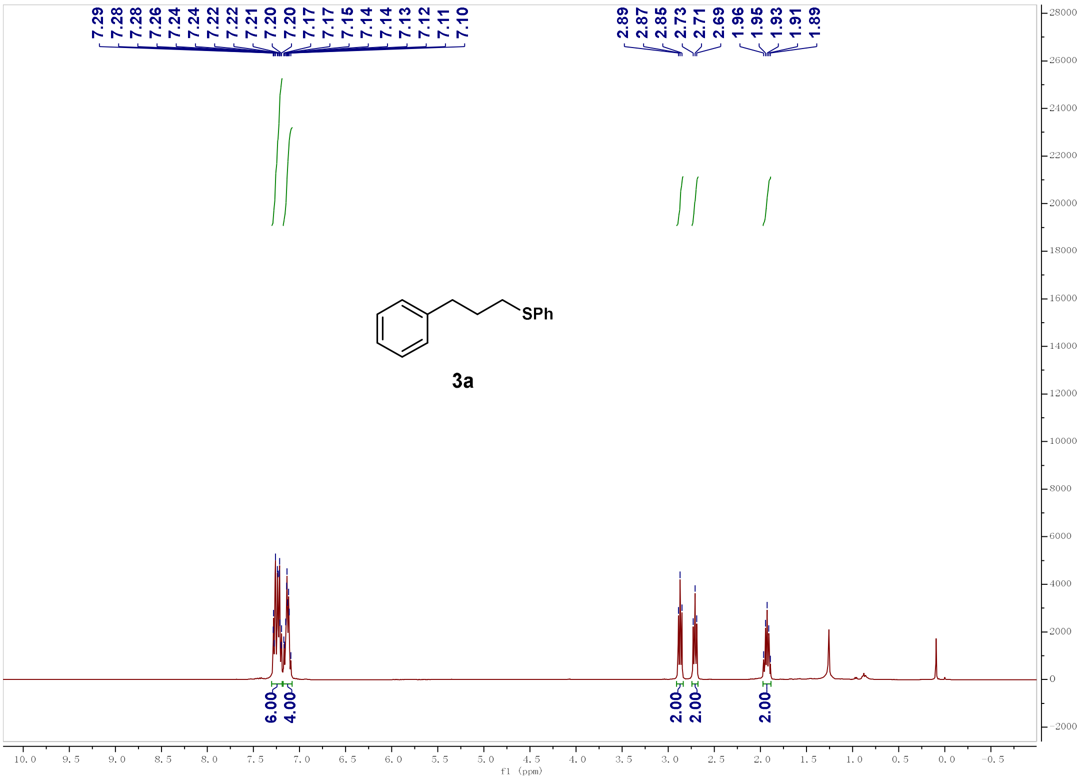

Supplementary Figure 4. ^{1}H NMR spectra for 3a

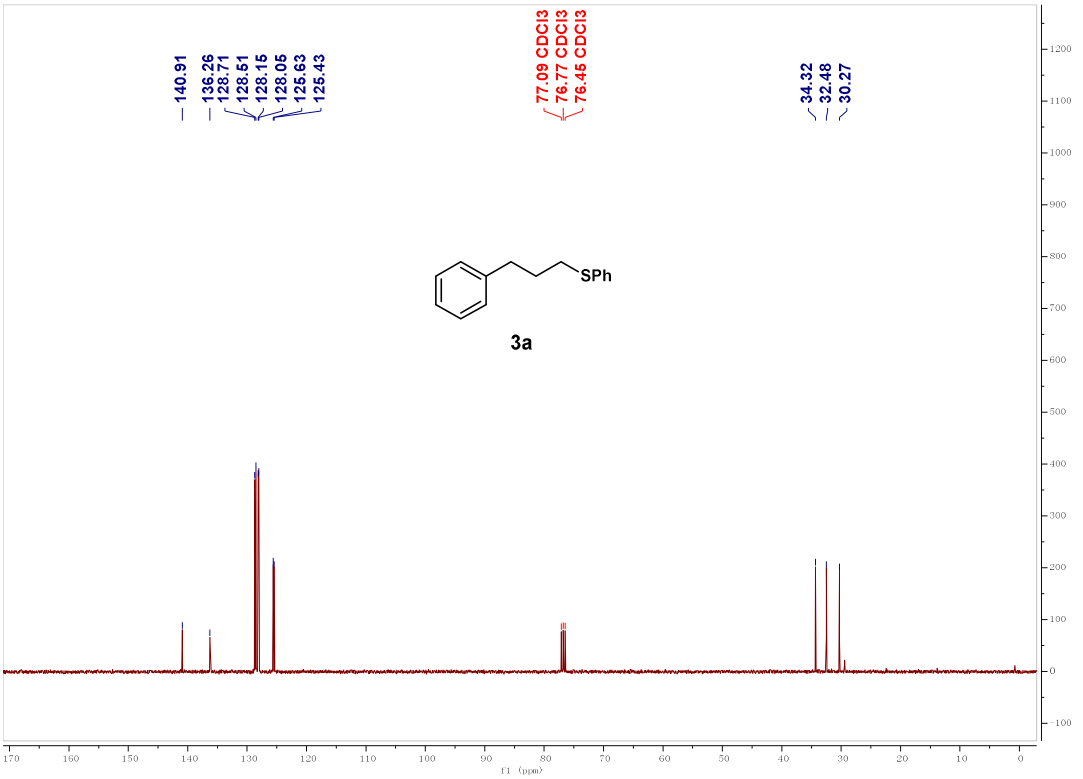

Supplementary Figure 5. ^{13}C NMR spectra for 3a

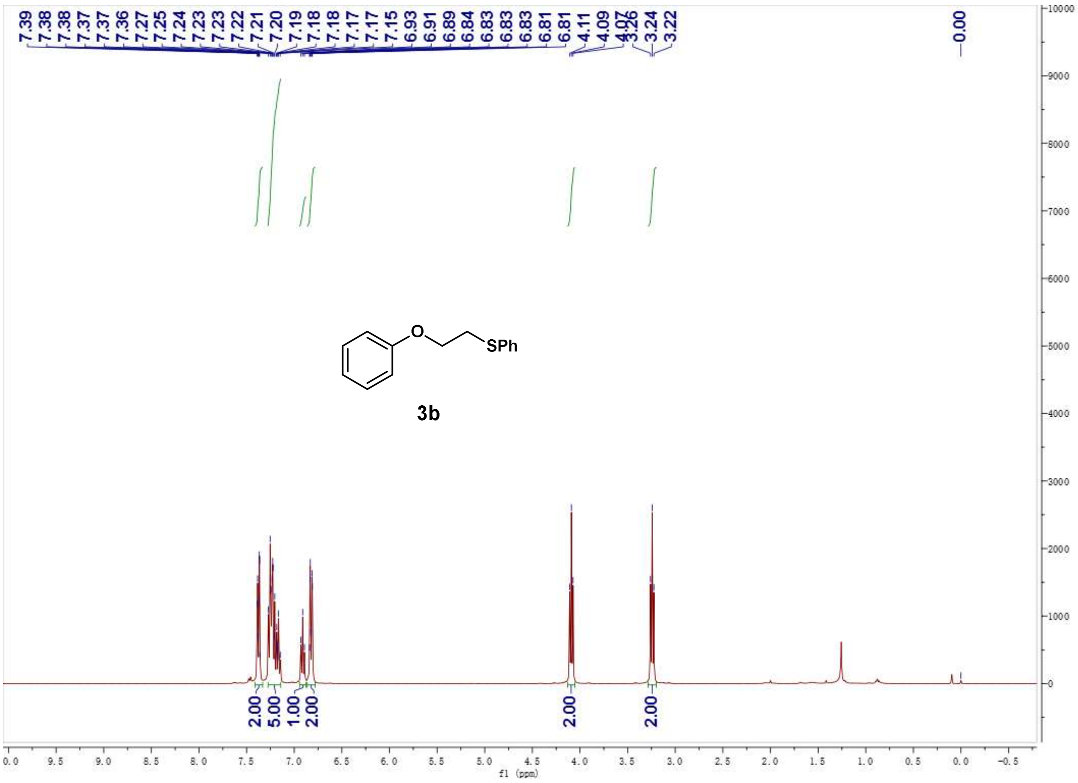

Supplementary Figure 6. ^{1}**H NMR spectra for 3b**

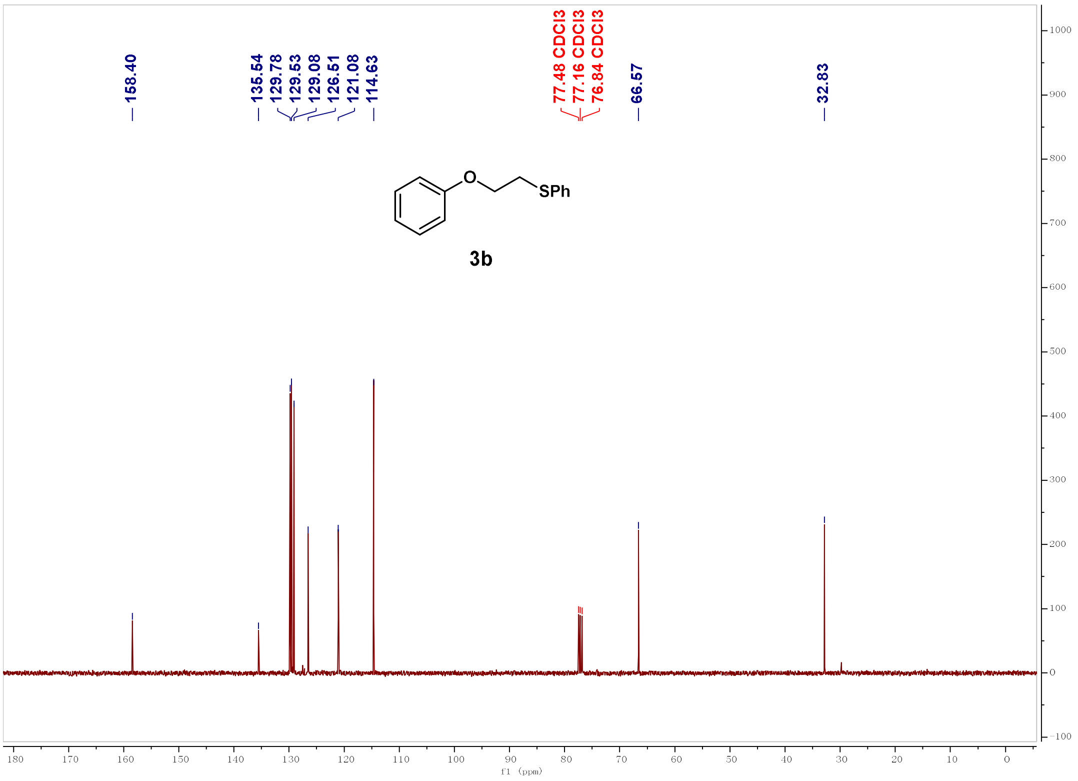

Supplementary Figure 7. ^{13}**C NMR spectra for 3b**

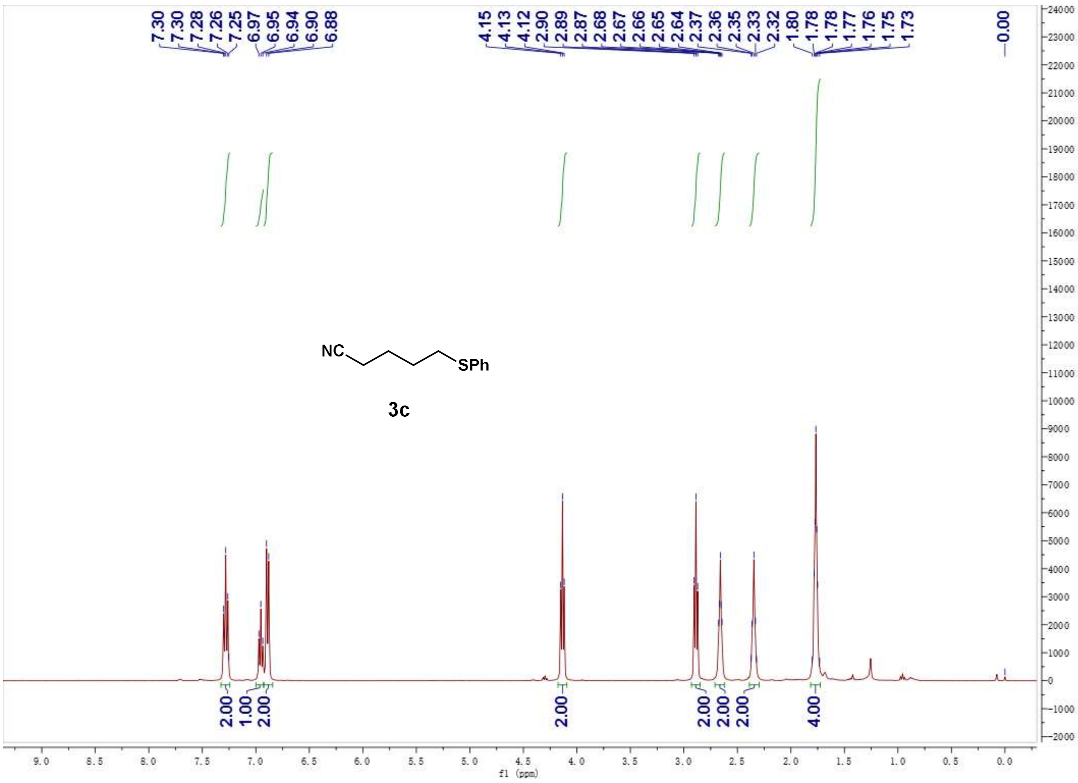

Supplementary Figure 8. ^{1}H NMR spectra for 3c

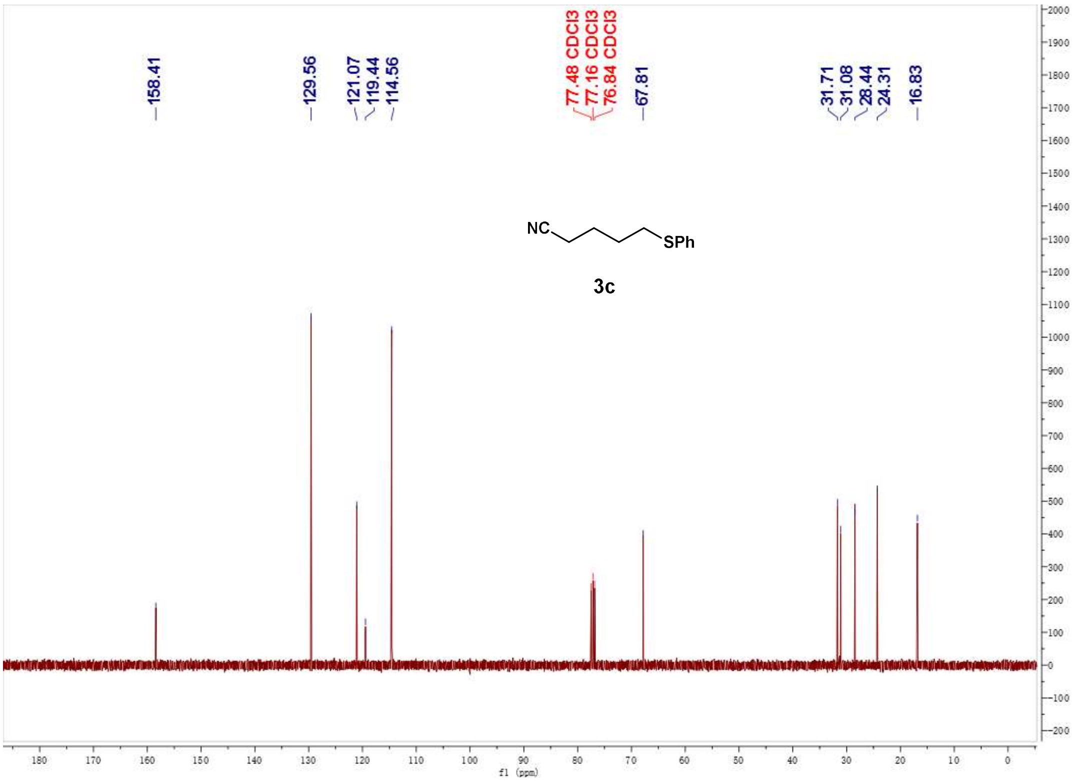

Supplementary Figure 9. ^{13}C NMR spectra for 3c

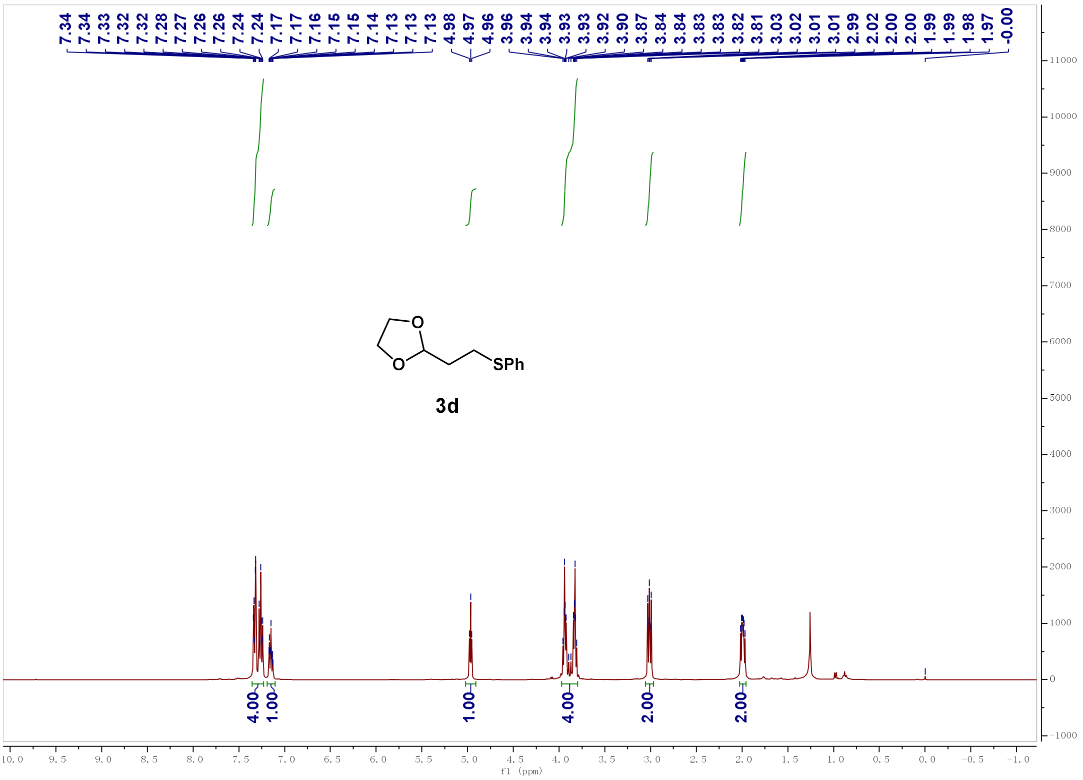

Supplementary Figure 10. ^{1}H NMR spectra for 3d

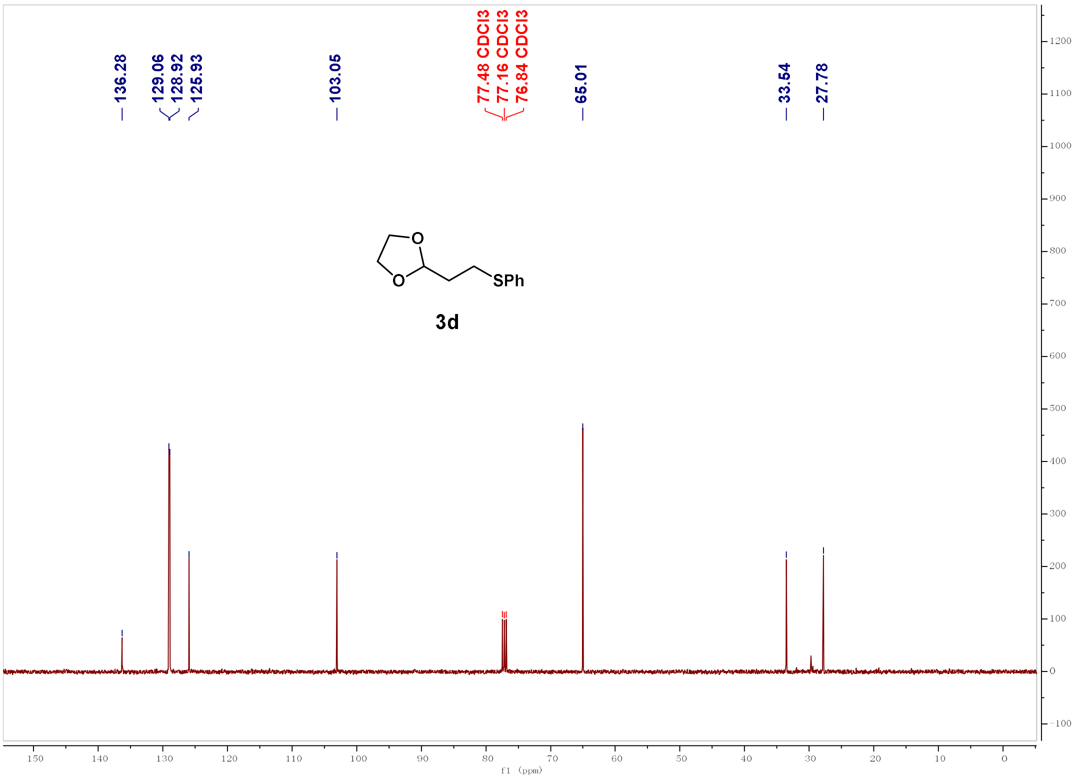

Supplementary Figure 11. ^{13}C NMR spectra for 3d

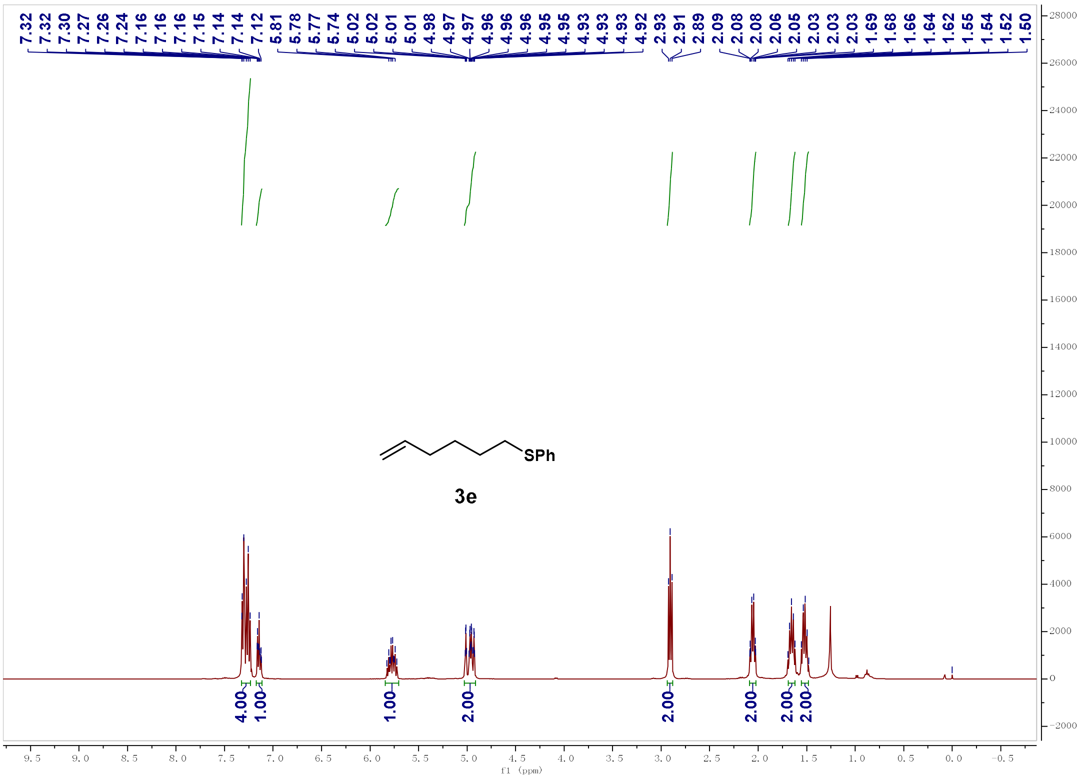

Supplementary Figure 12. ^{1}**H NMR spectra for 3e**

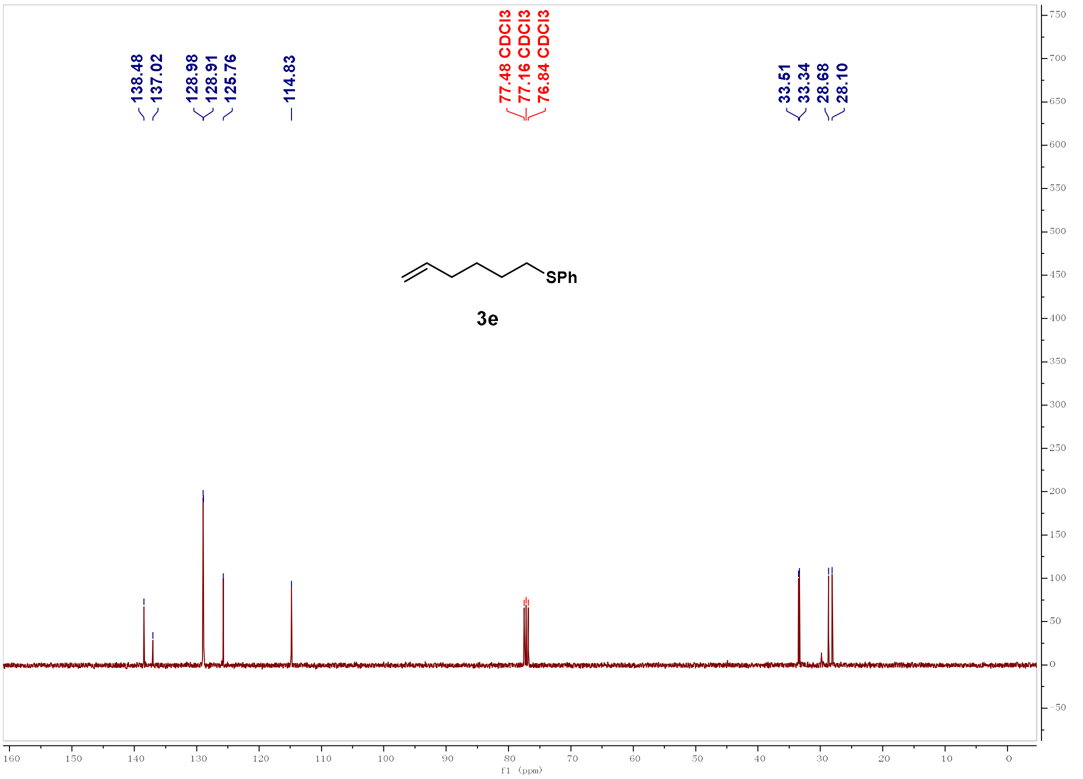

Supplementary Figure 13. ^{13}**C NMR spectra for 3e**

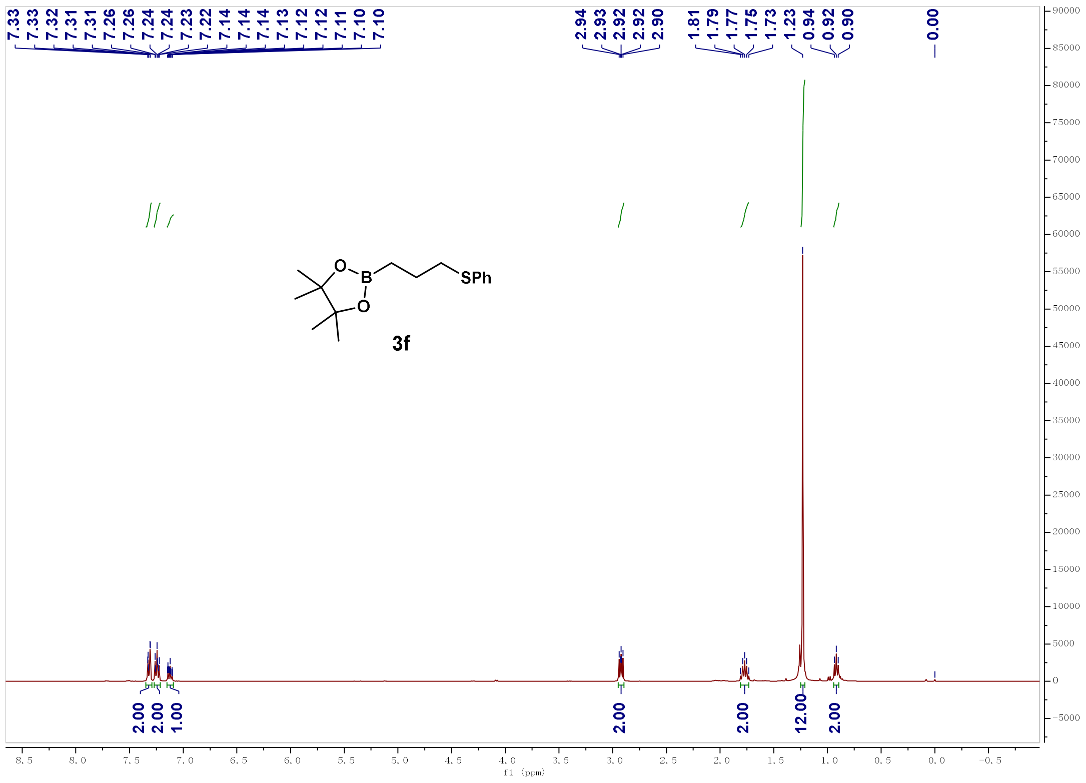

Supplementary Figure 14. ^{1}**H NMR spectra for 3f**

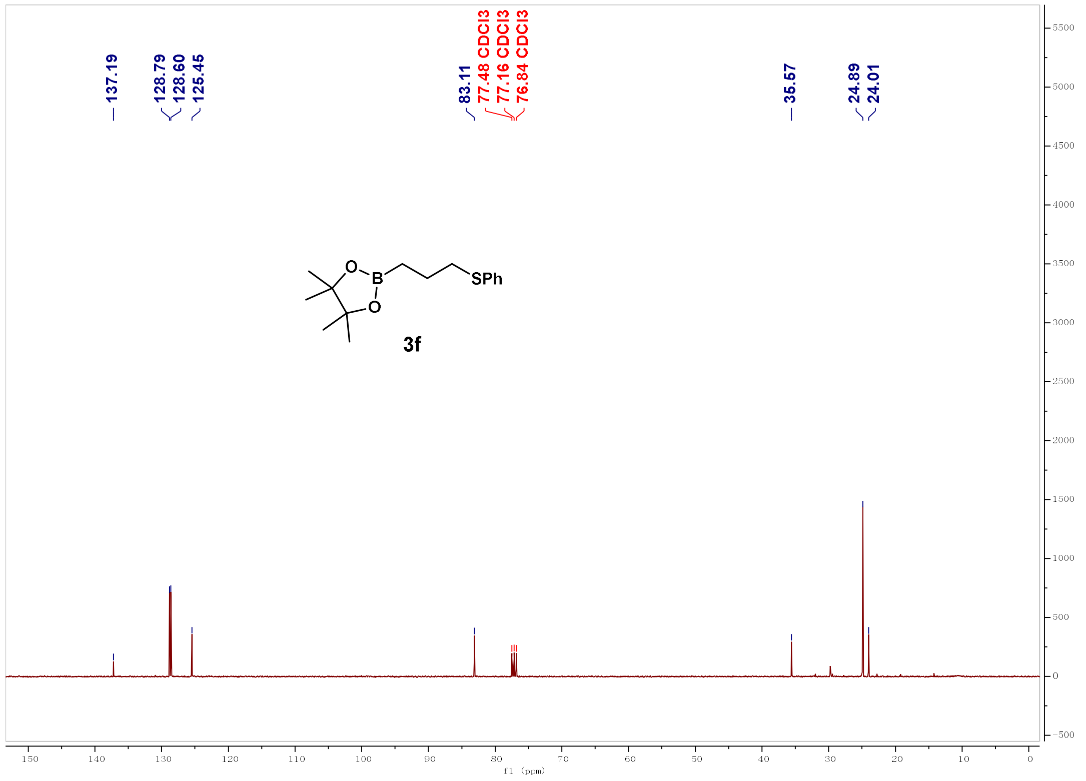

Supplementary Figure 15. ^{13}**C NMR spectra for 3f**

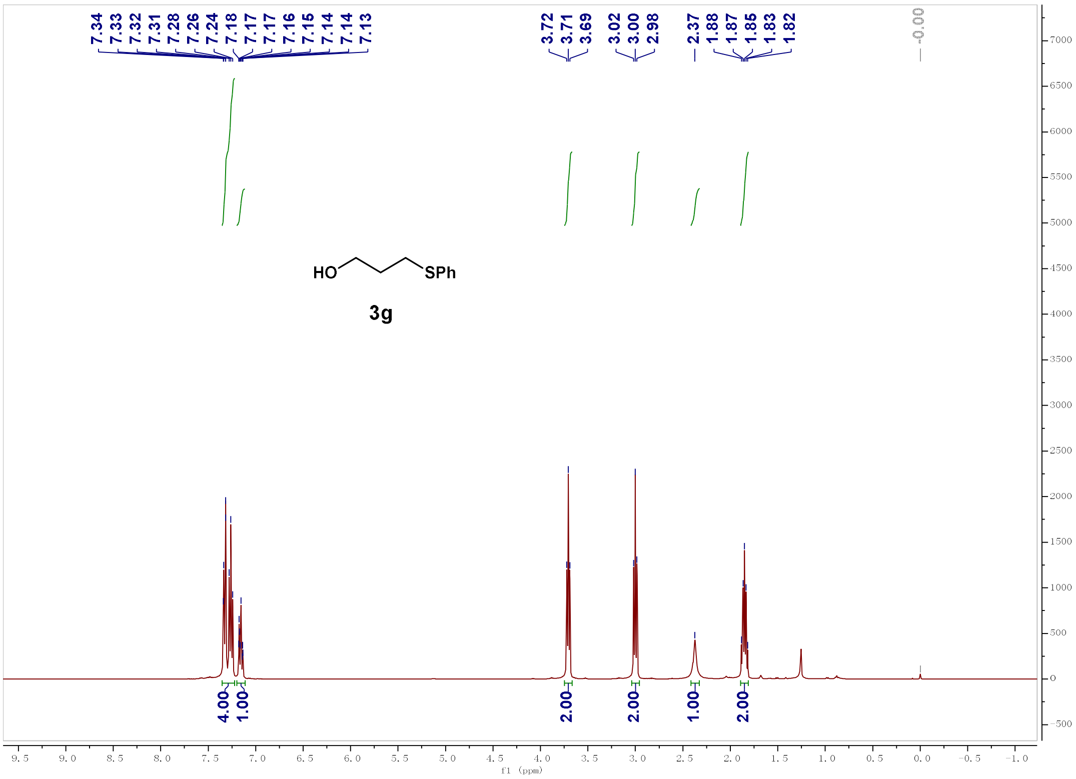

Supplementary Figure 16. ^{1}H NMR spectra for 3g

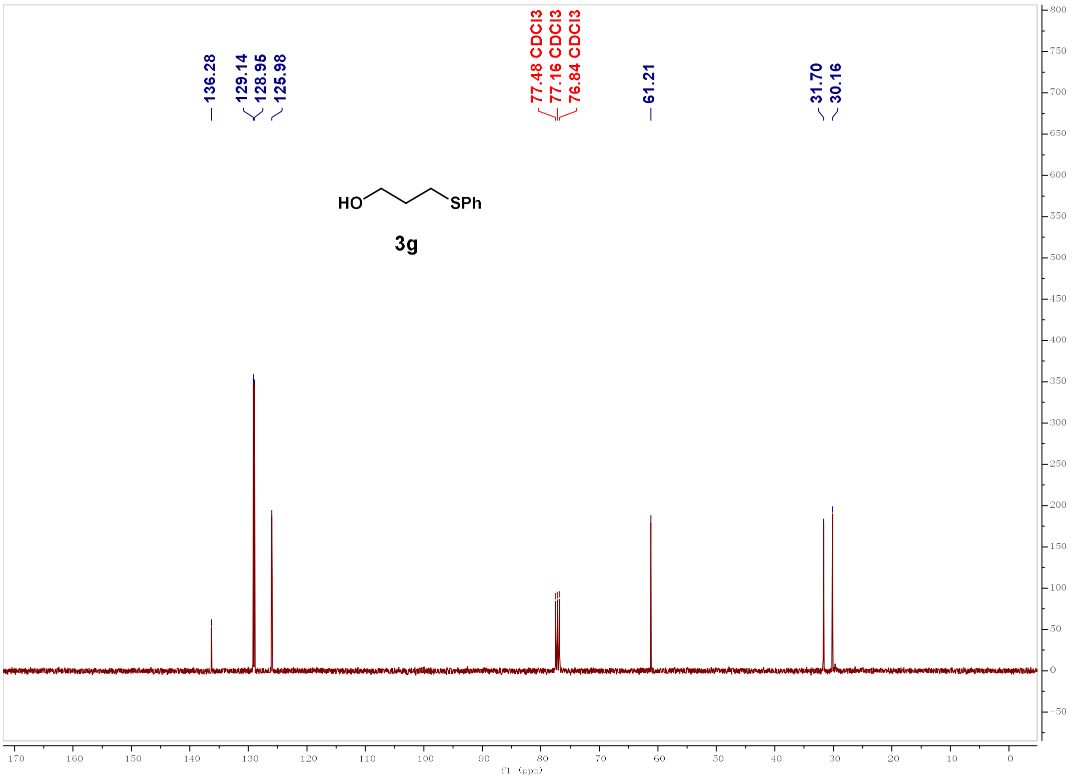

Supplementary Figure 17. ^{13}C NMR spectra for 3g

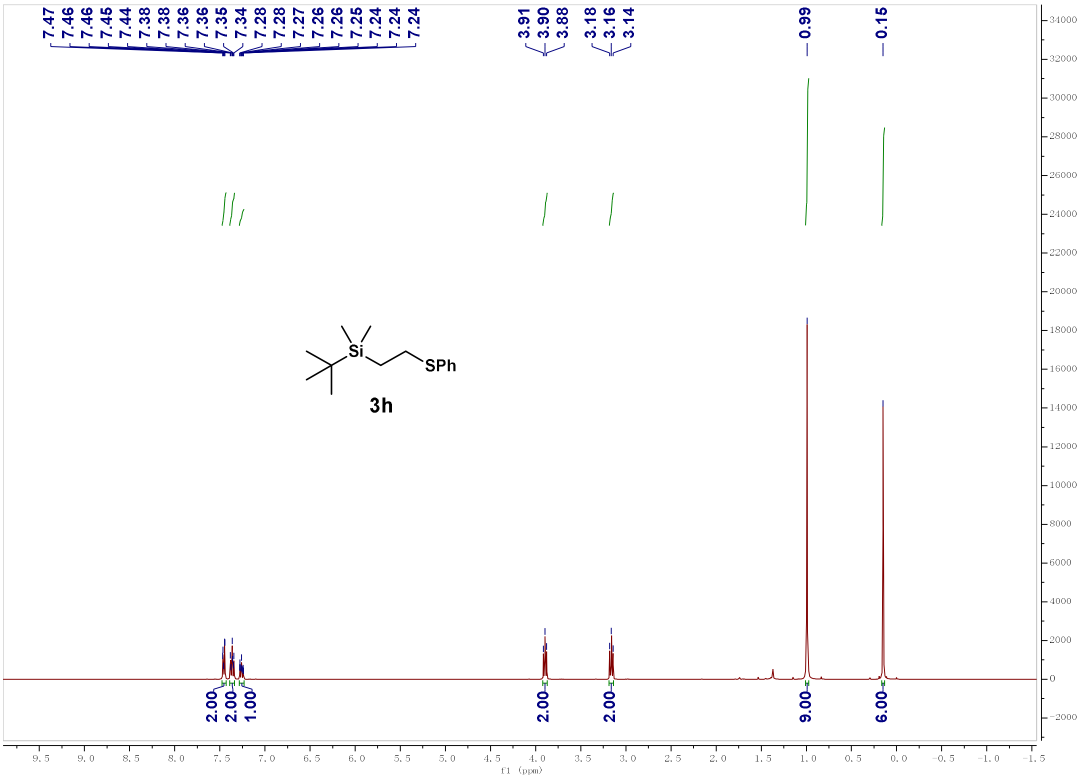

Supplementary Figure 18. ^{1}**H NMR spectra for 3h**

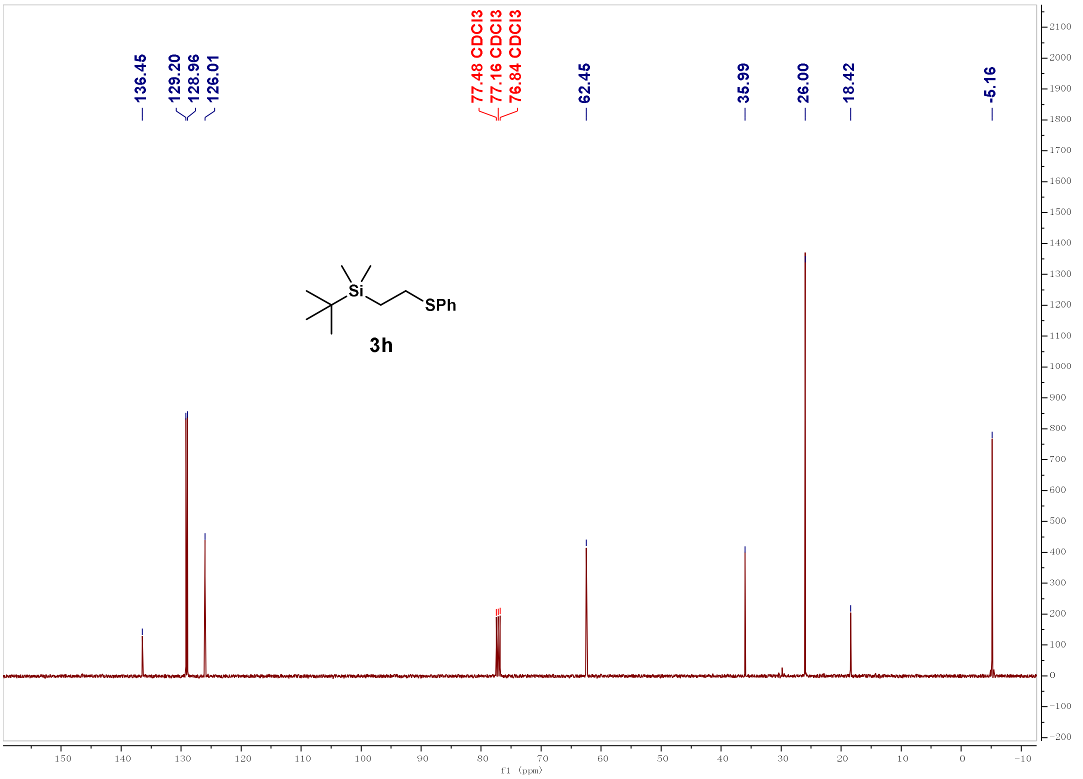

Supplementary Figure 19. ^{13}**C NMR spectra for 3h**

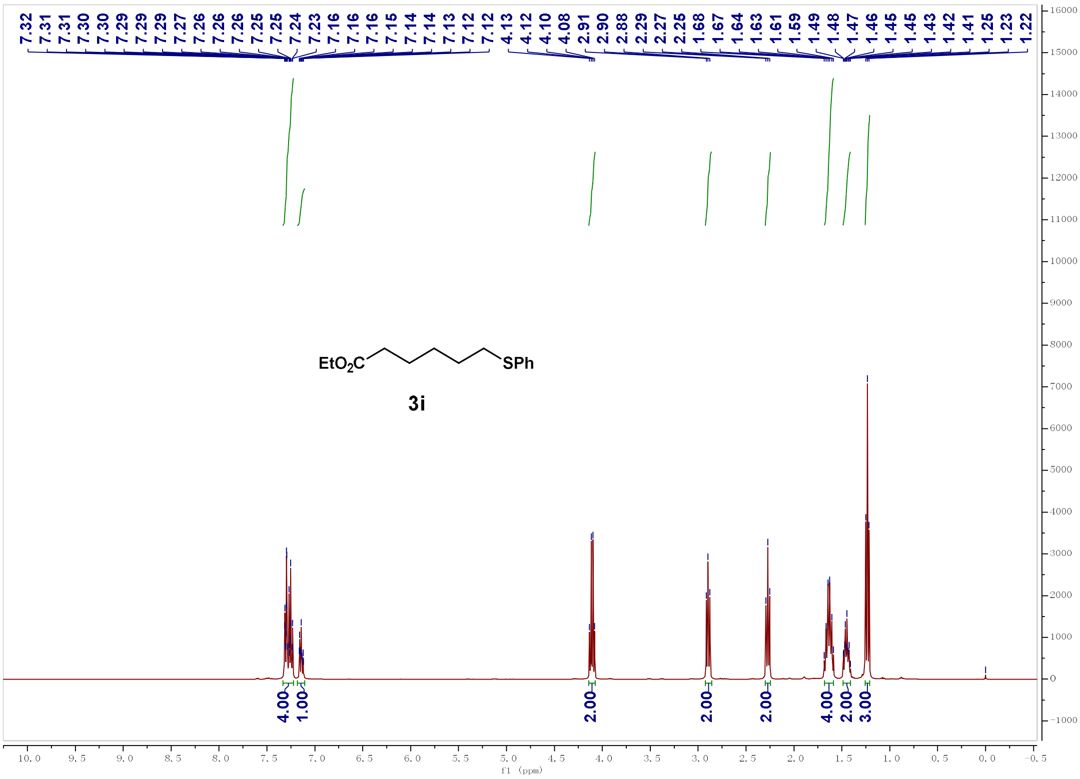

Supplementary Figure 20. ^{1}H NMR spectra for 3i

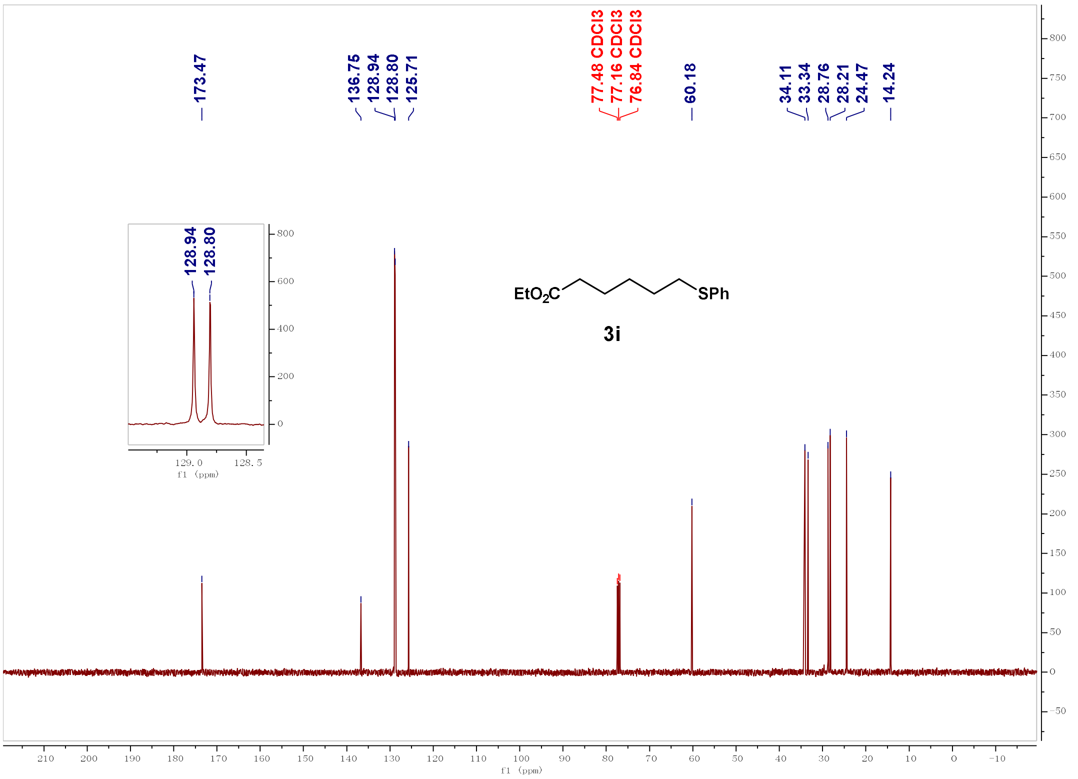

Supplementary Figure 21. ^{13}C NMR spectra for 3i

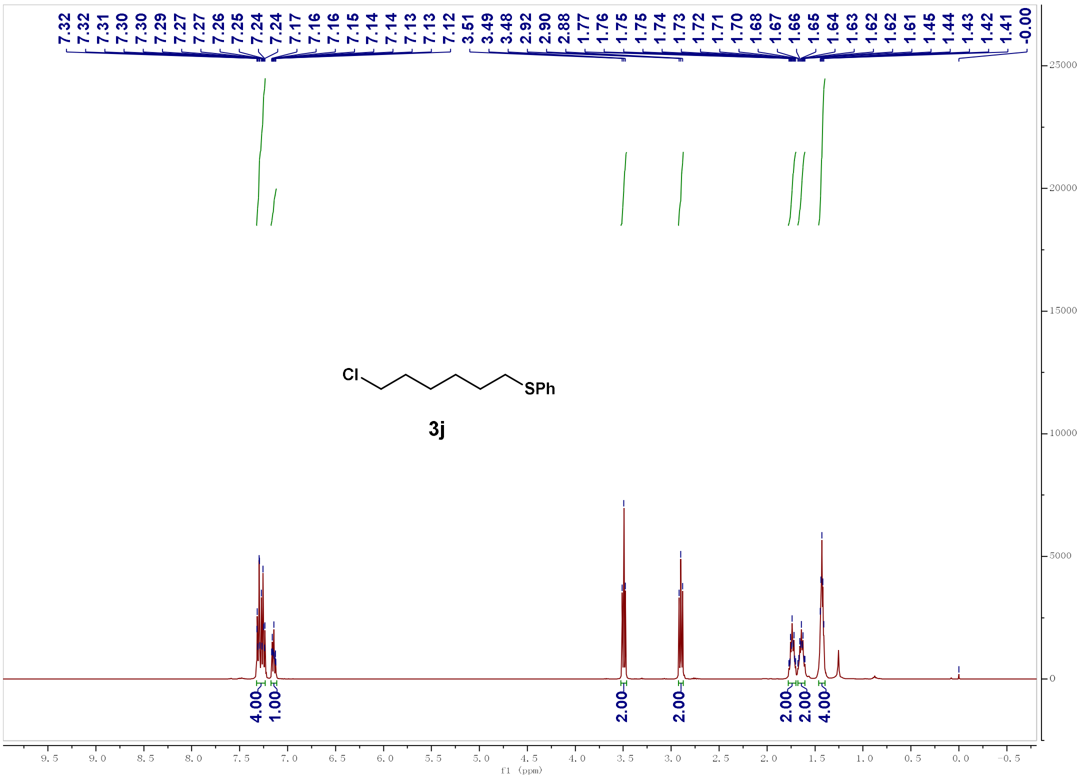

Supplementary Figure 22. ^{1}**H NMR spectra for 3j**

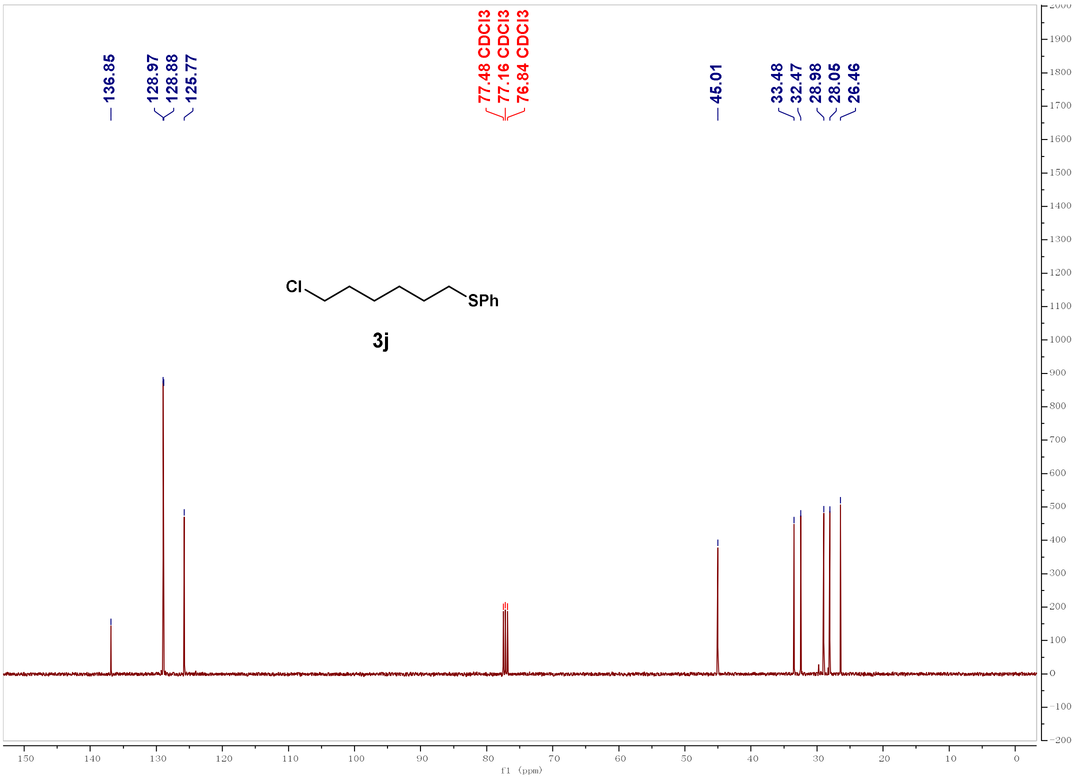

Supplementary Figure 23. ^{1}**H NMR spectra for 3j**

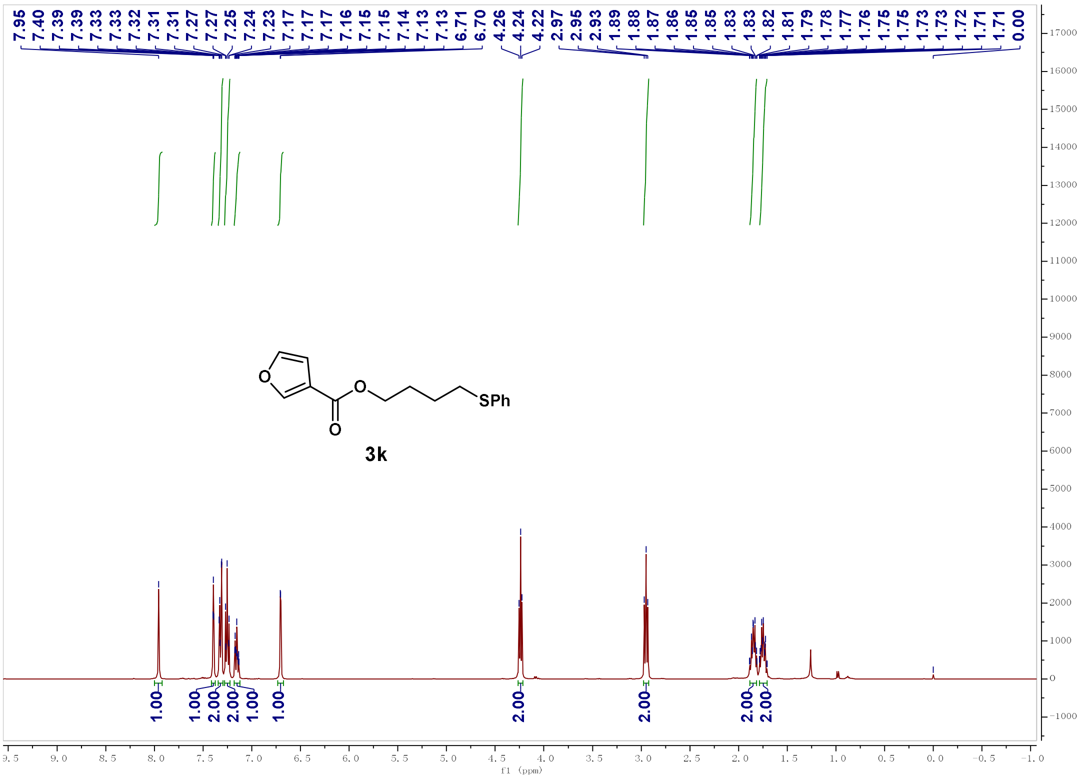

Supplementary Figure 24. ^{1}H NMR spectra for 3k

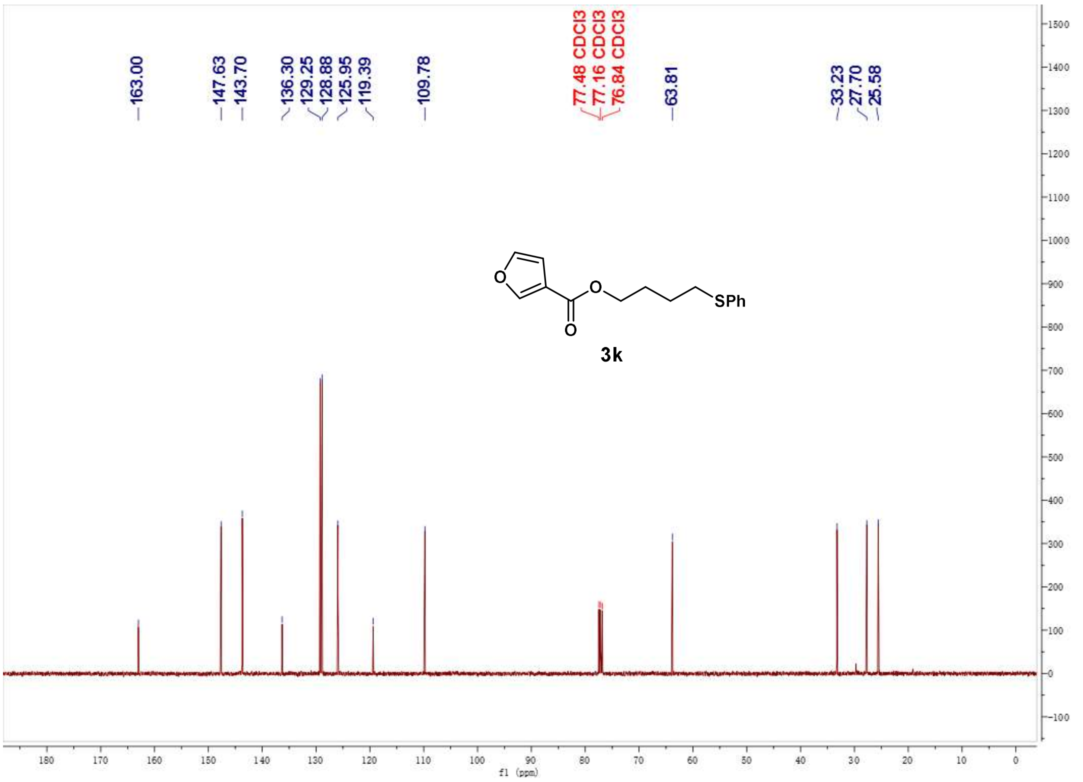

Supplementary Figure 25. ^{13}C NMR spectra for 3k

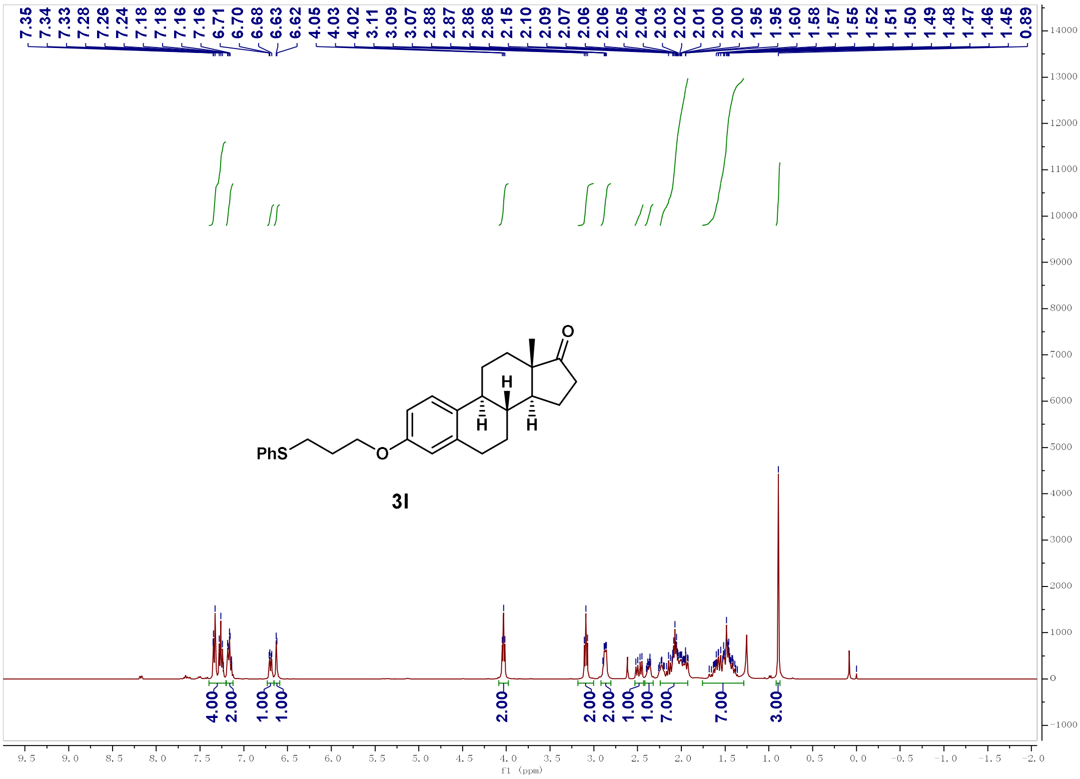

Supplementary Figure 26. ^{1}**H NMR spectra for 3l**

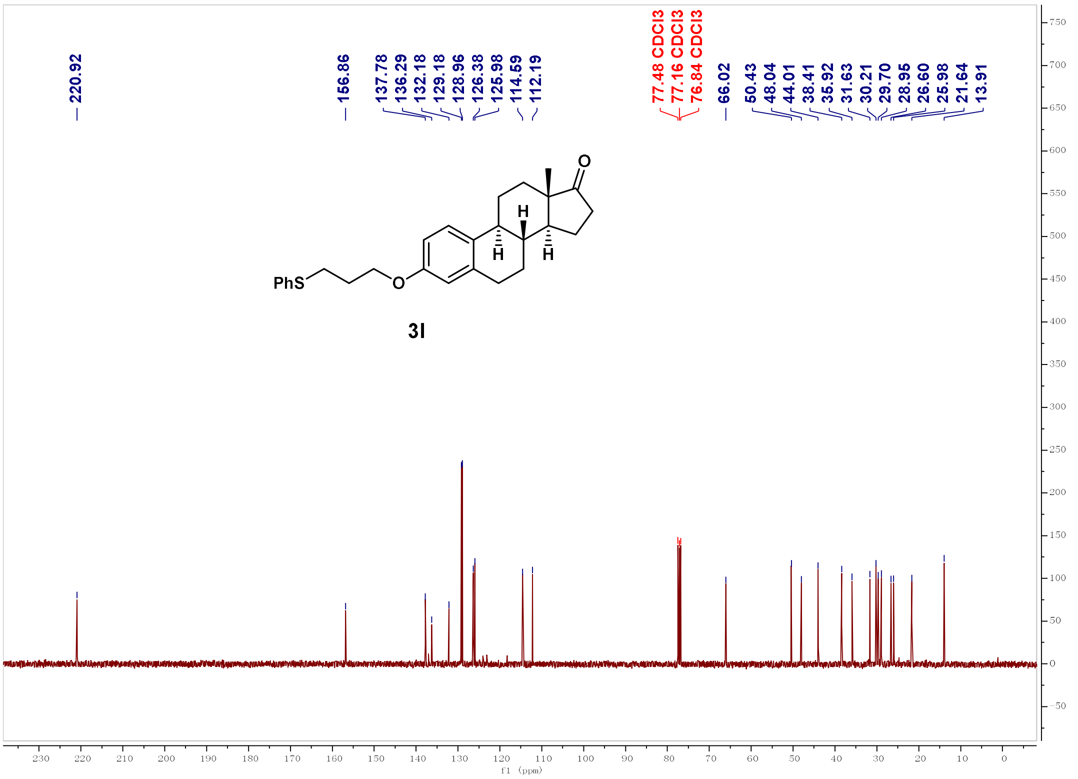

Supplementary Figure 27. ^{1}**H NMR spectra for 3l**

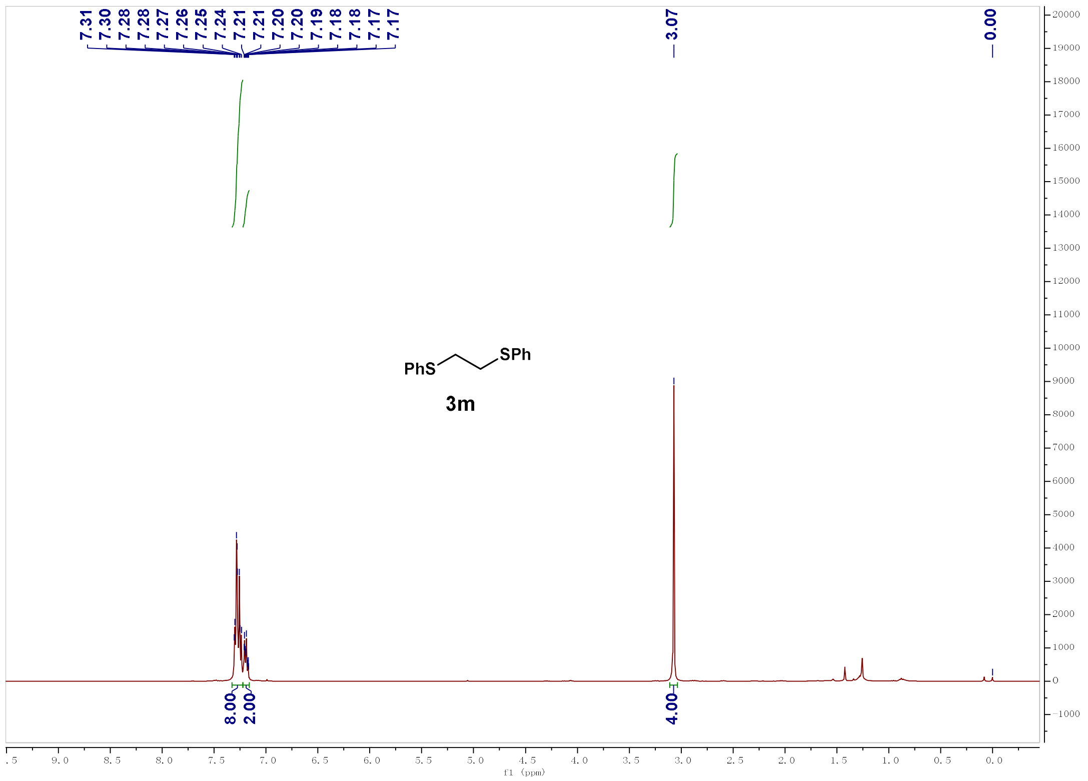

Supplementary Figure 28. ^{1}H **NMR spectra for 3m**

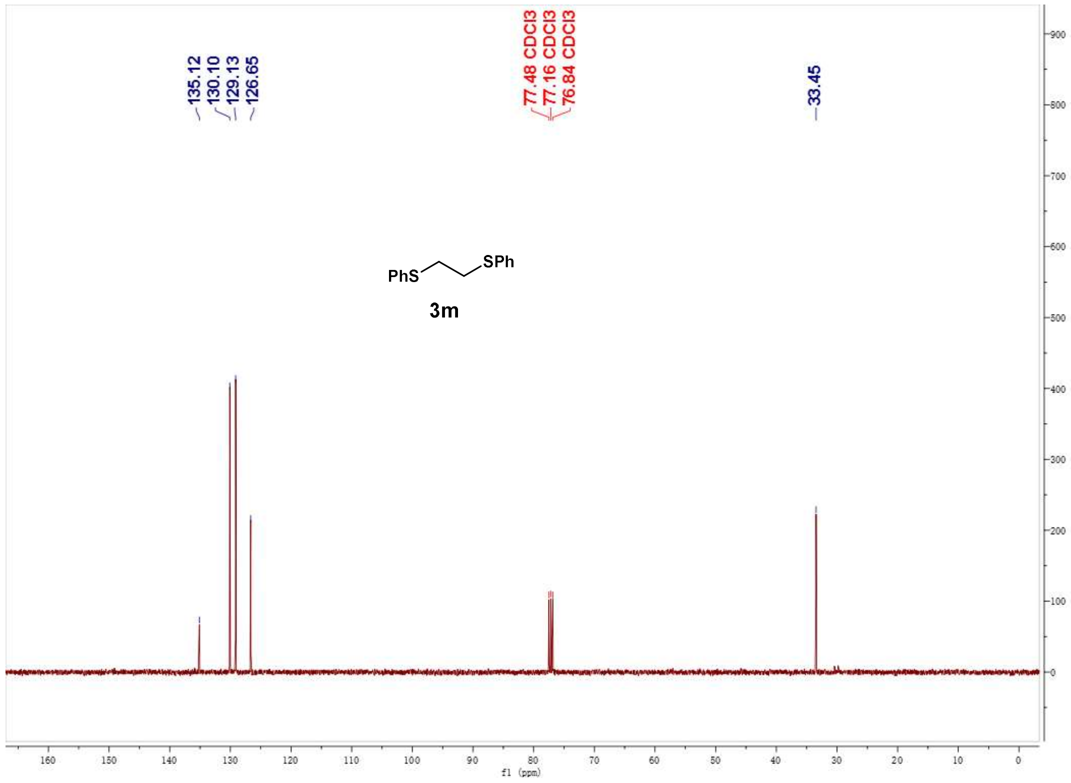

Supplementary Figure 29. ^{13}C **NMR spectra for 3m**

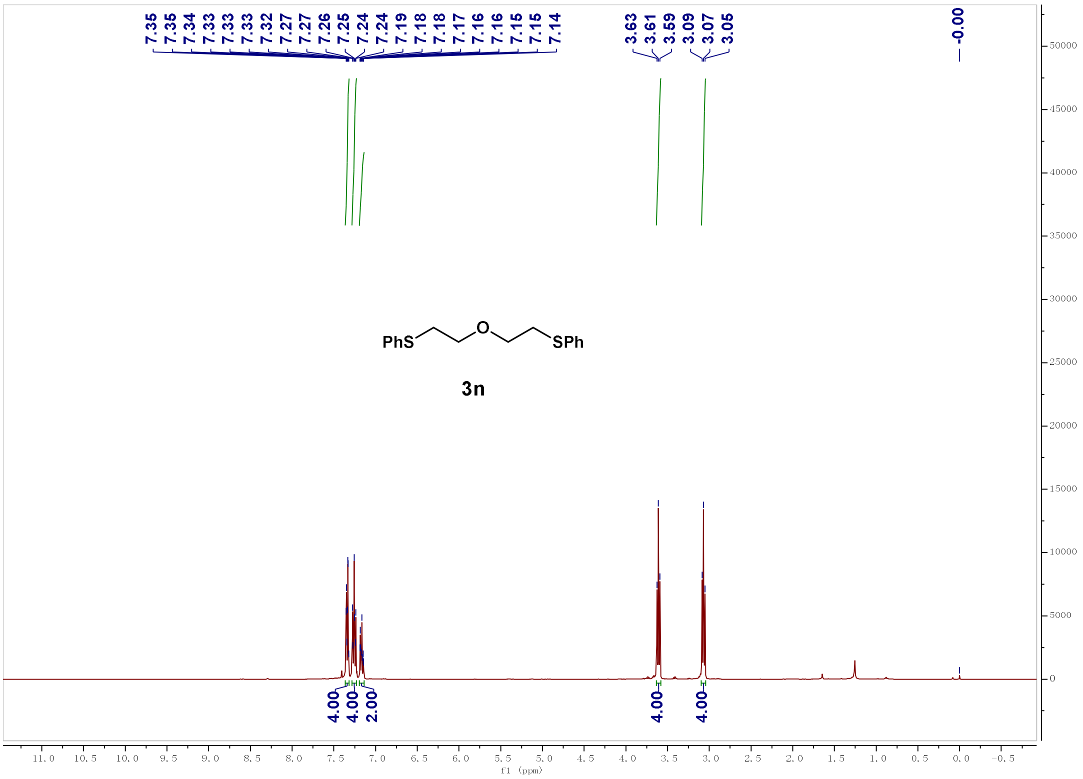

Supplementary Figure 30. ^{1}**H NMR spectra for 3n**

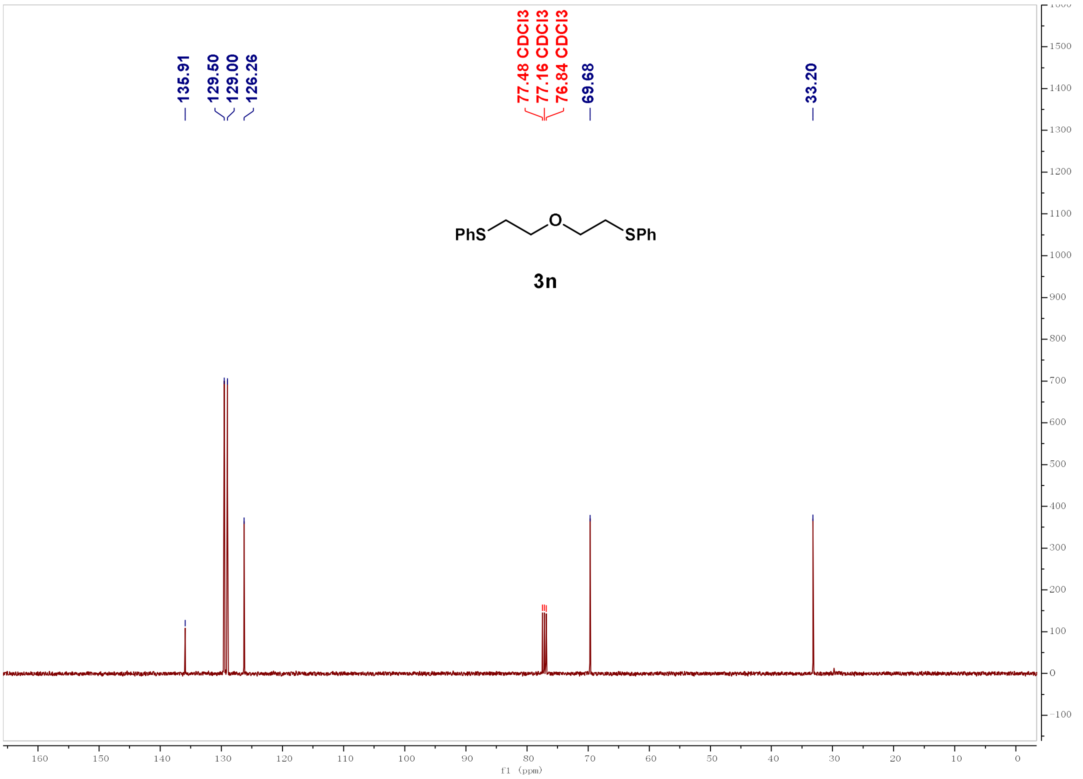

Supplementary Figure31. ^{13}**C NMR spectra for 3n**

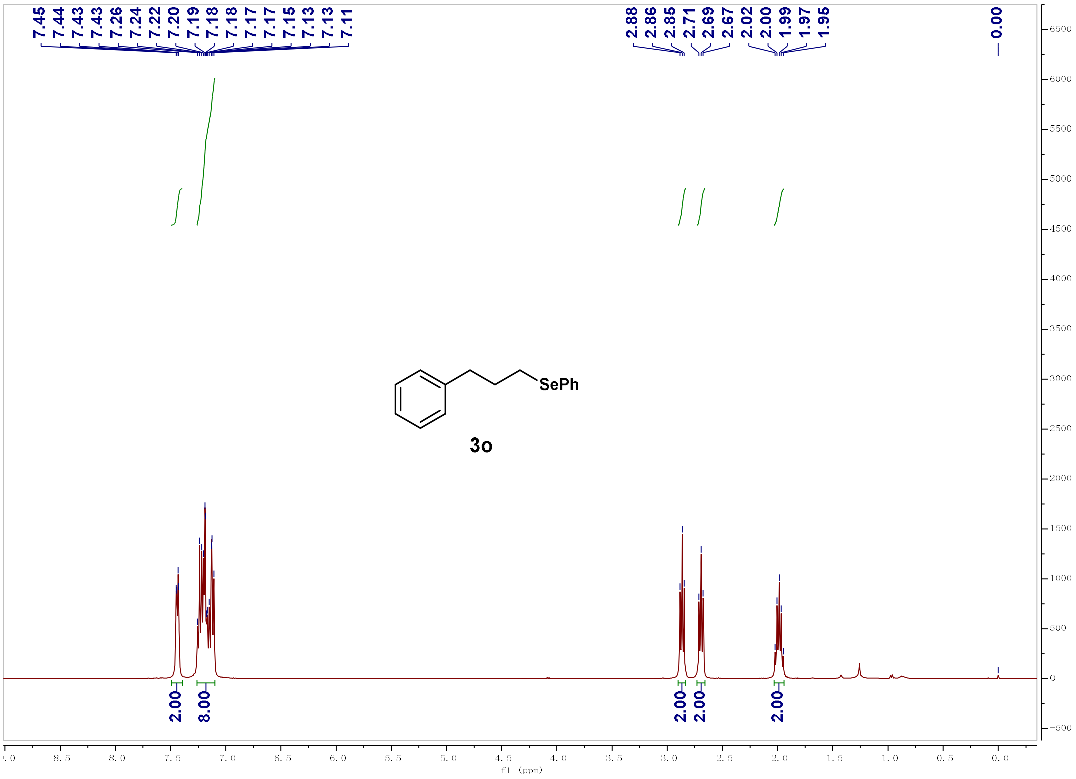

Supplementary Figure 32. ^{1}H NMR spectra for 3o

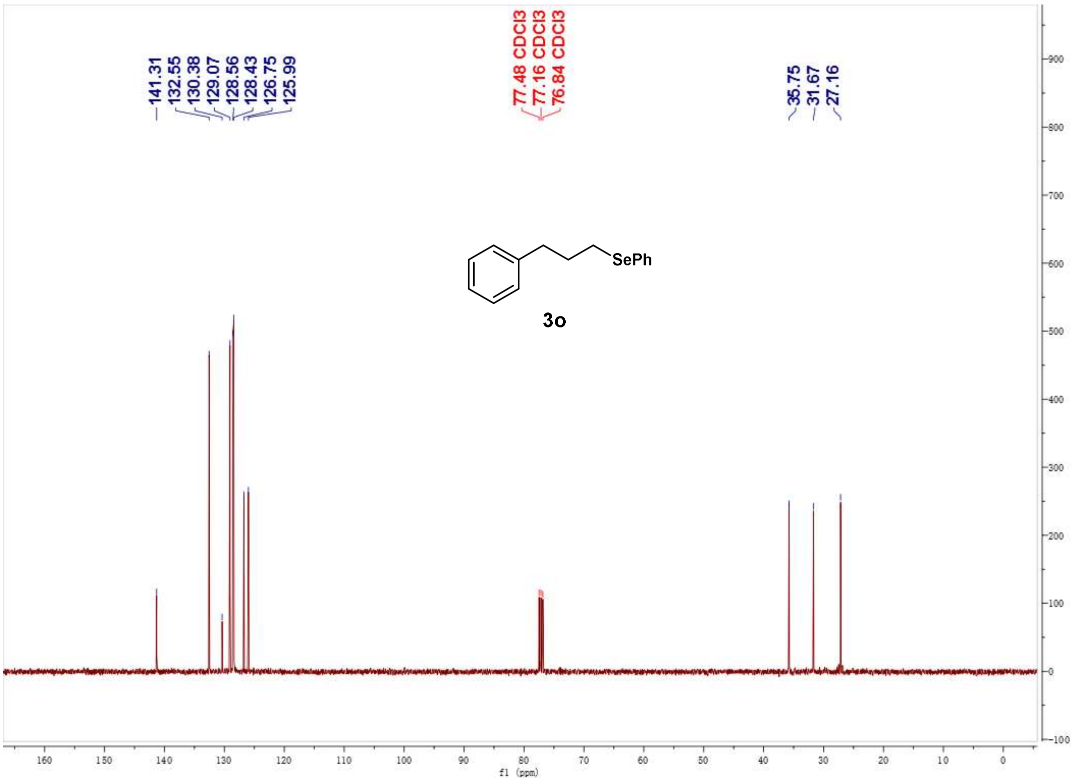

Supplementary Figure 33. ^{13}C NMR spectra for 3o

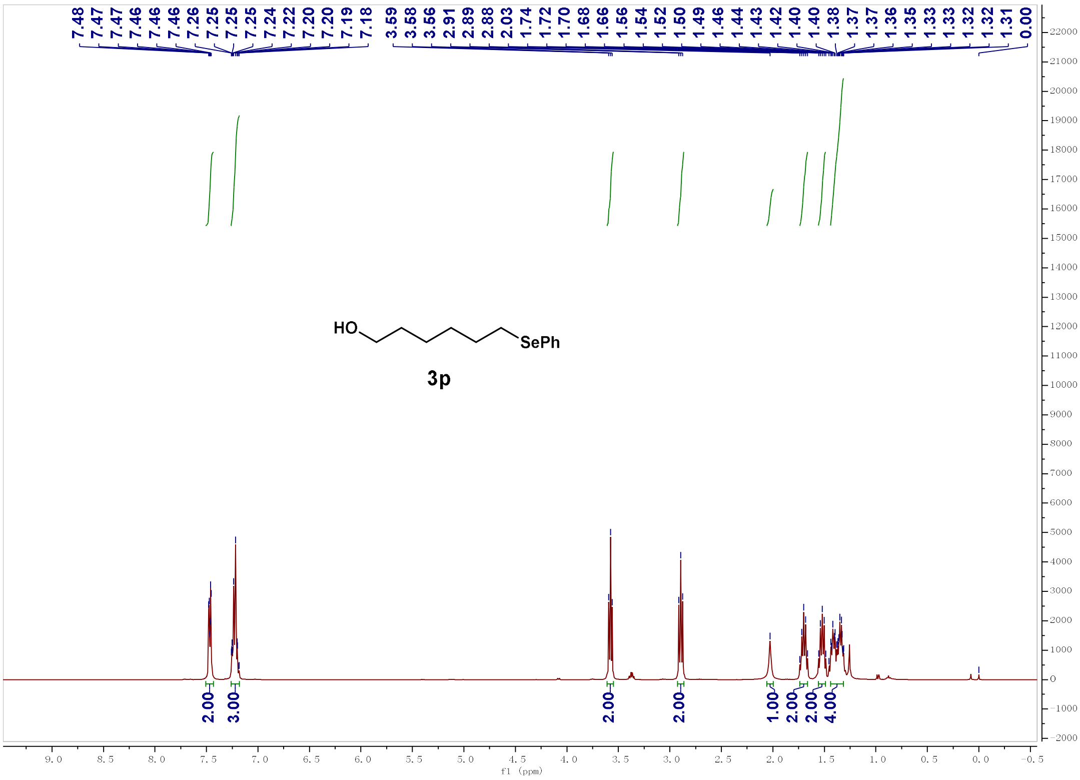

Supplementary Figure 34. ^{1}H NMR spectra for 3p

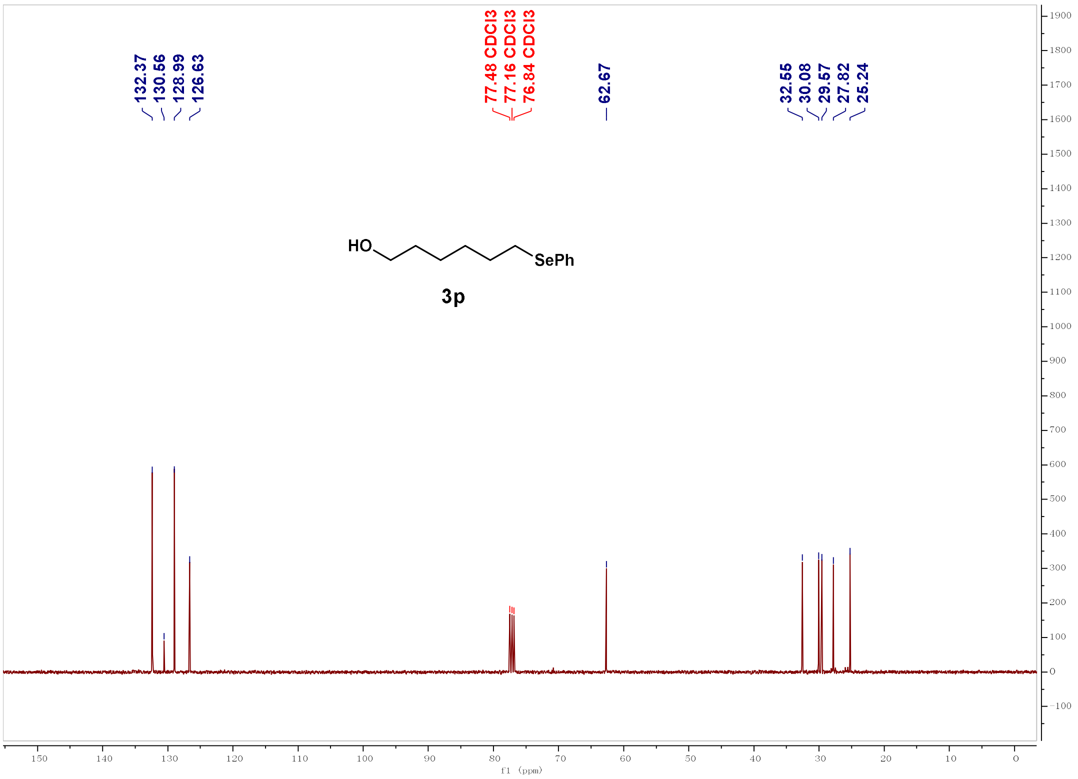

Supplementary Figure 35. ^{13}C NMR spectra for 3p

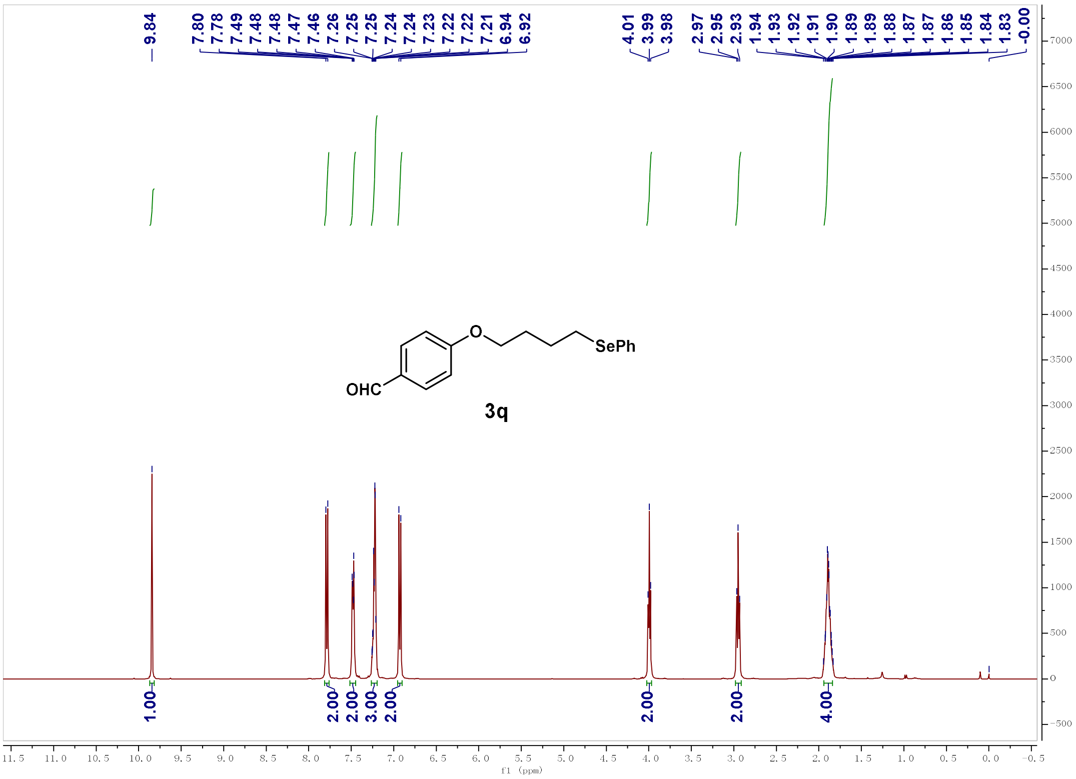

Supplementary Figure 36. ^{1}**H NMR spectra for 3q**

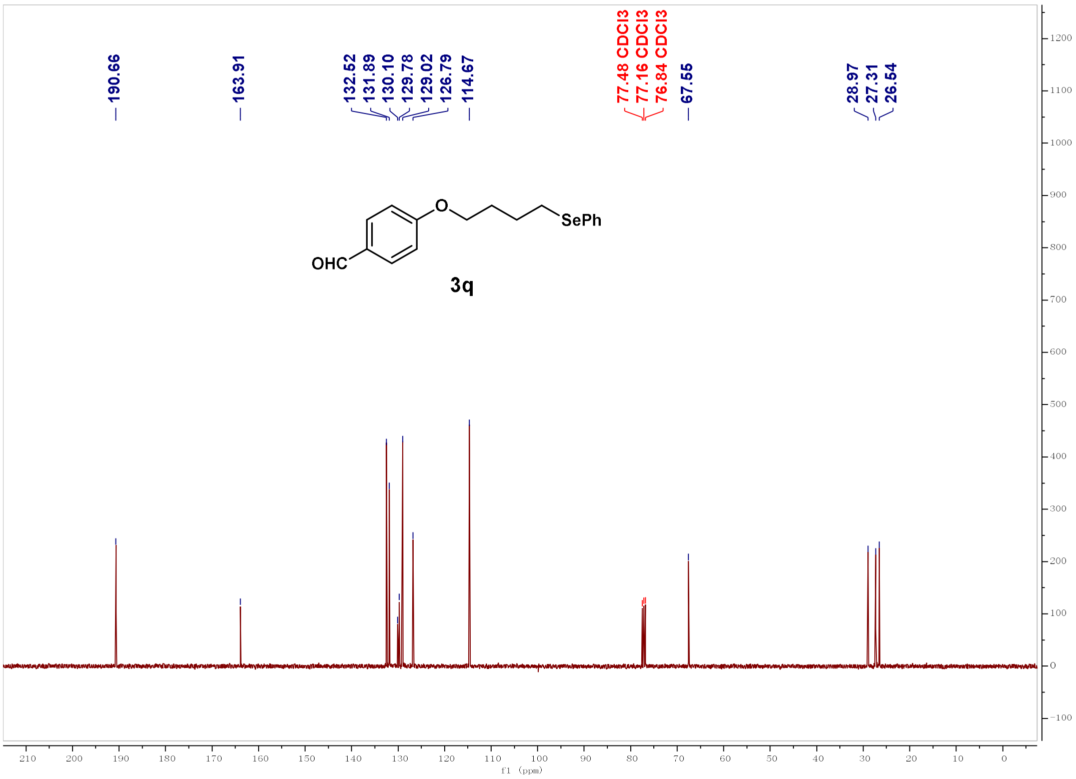

Supplementary Figure 37. ^{13}**C NMR spectra for 3q**

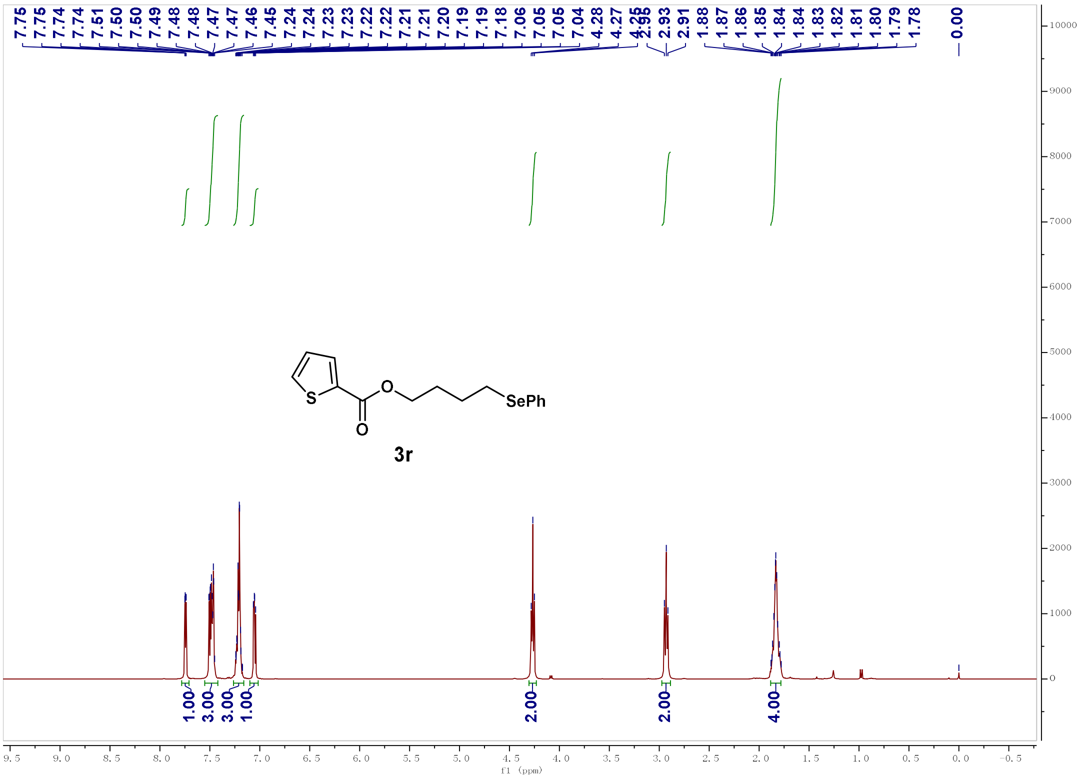

Supplementary Figure 38. ^{1}H NMR spectra for 3r

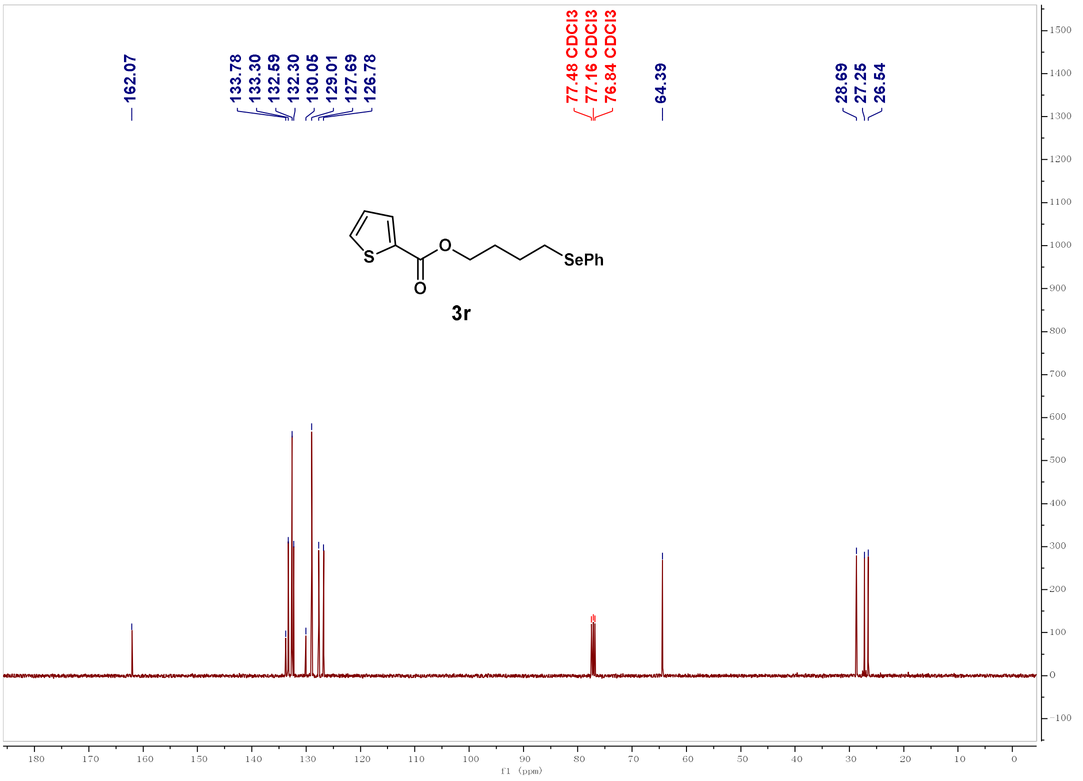

Supplementary Figure 39. ^{13}C NMR spectra for 3r

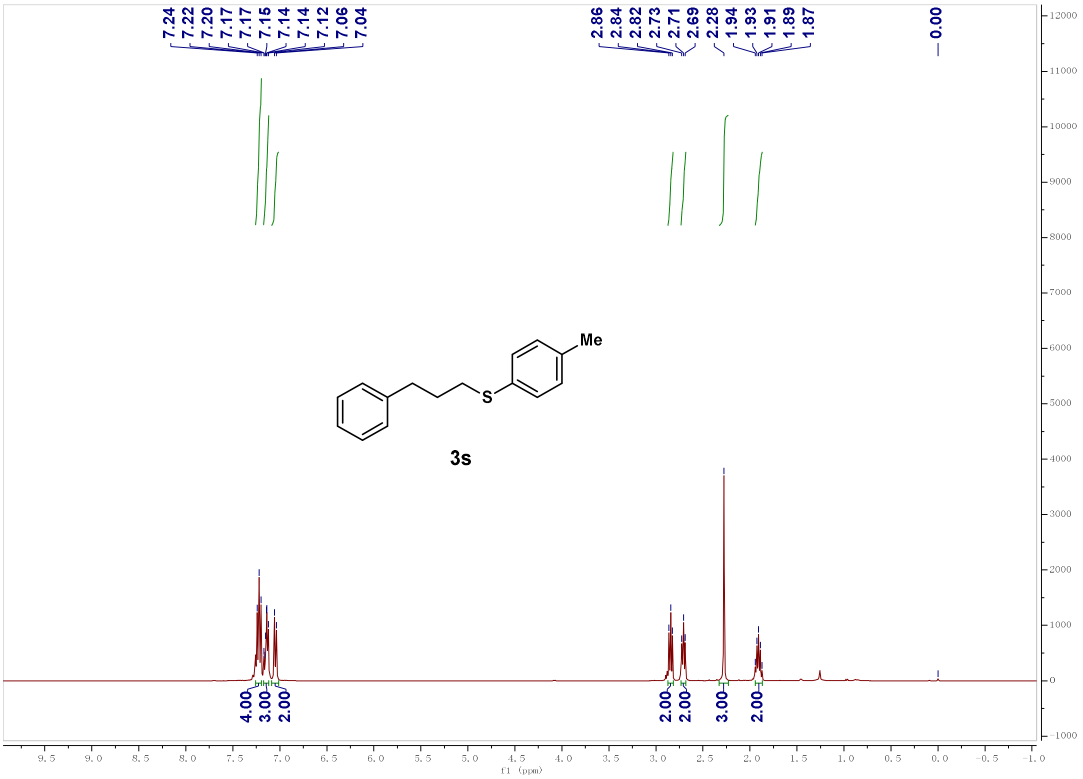

Supplementary Figure 40. ^{1}**H NMR spectra for 3s**

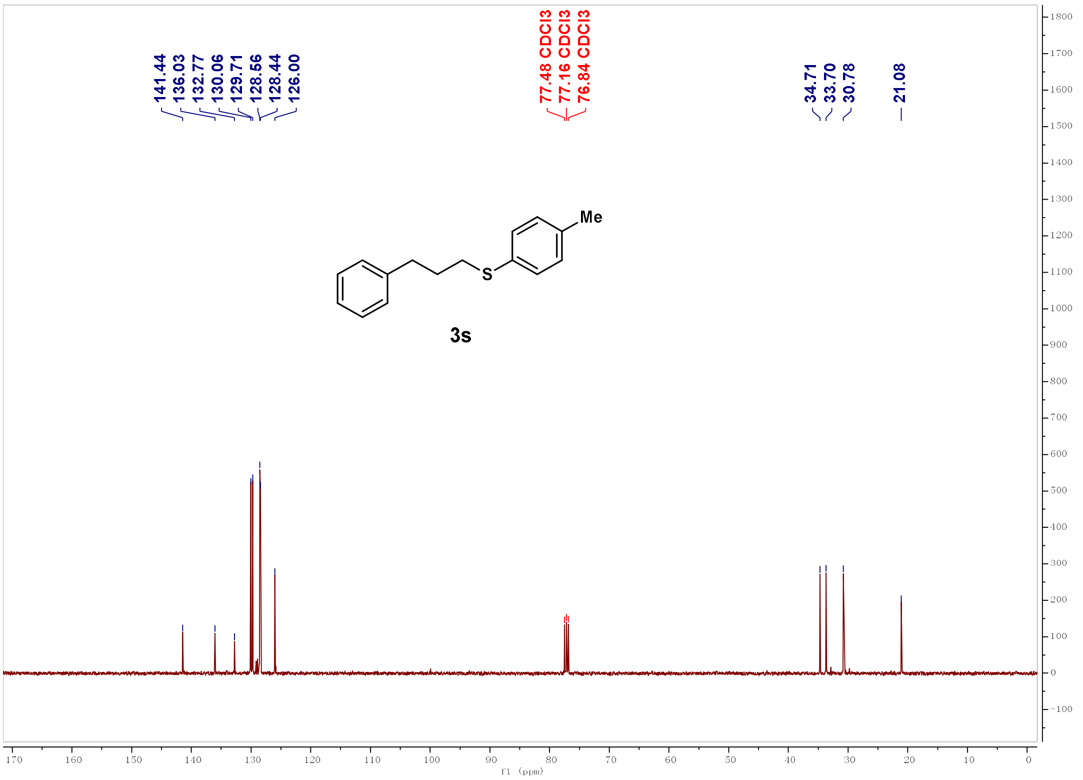

Supplementary Figure 41. ^{13}**C NMR spectra for 3s**

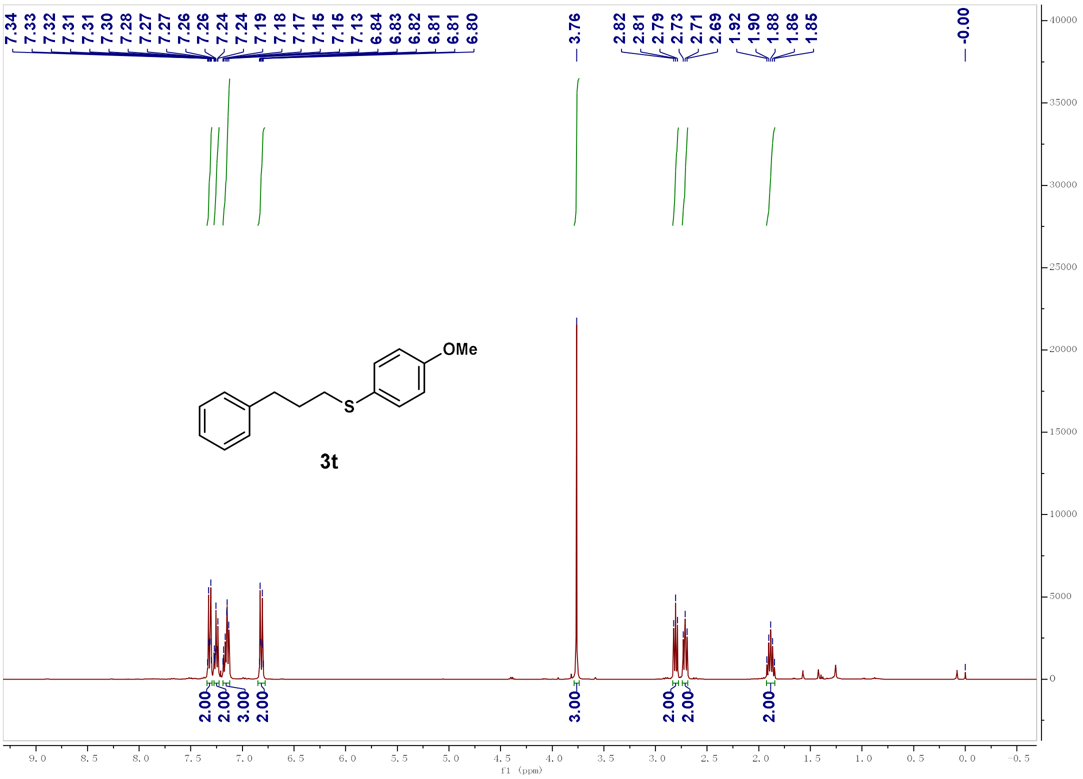

Supplementary Figure 42. ^{1}**H NMR spectra for 3t**

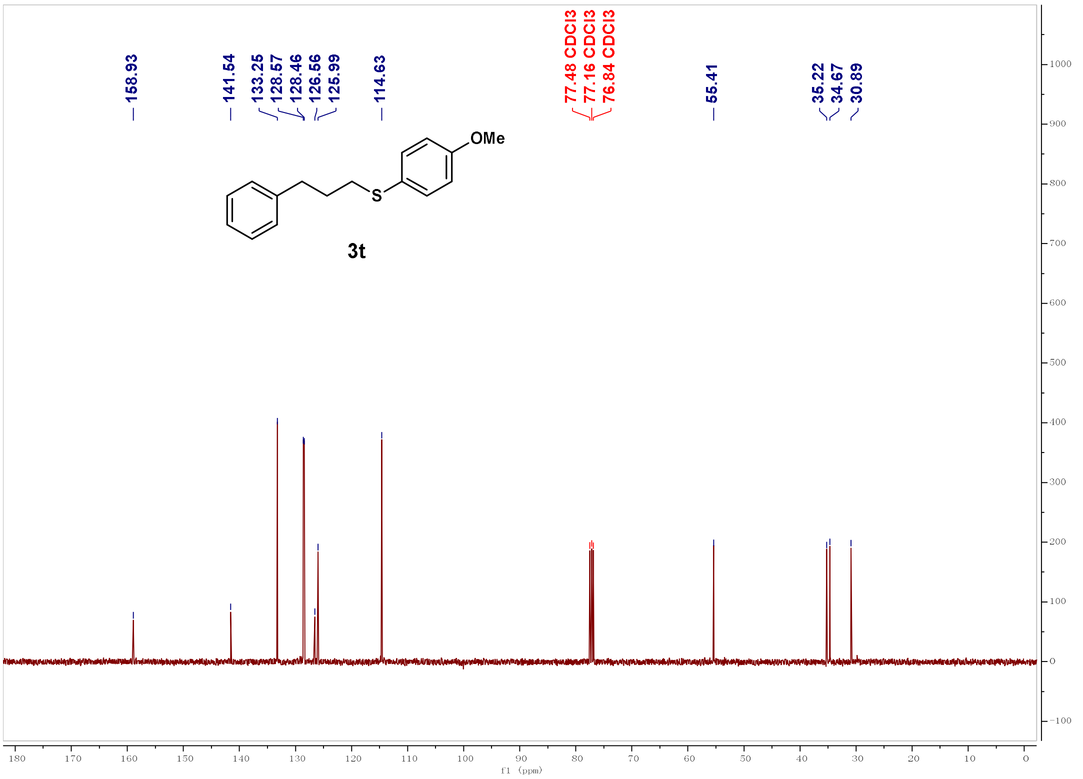

Supplementary Figure 43. ^{13}**C NMR spectra for 3t**

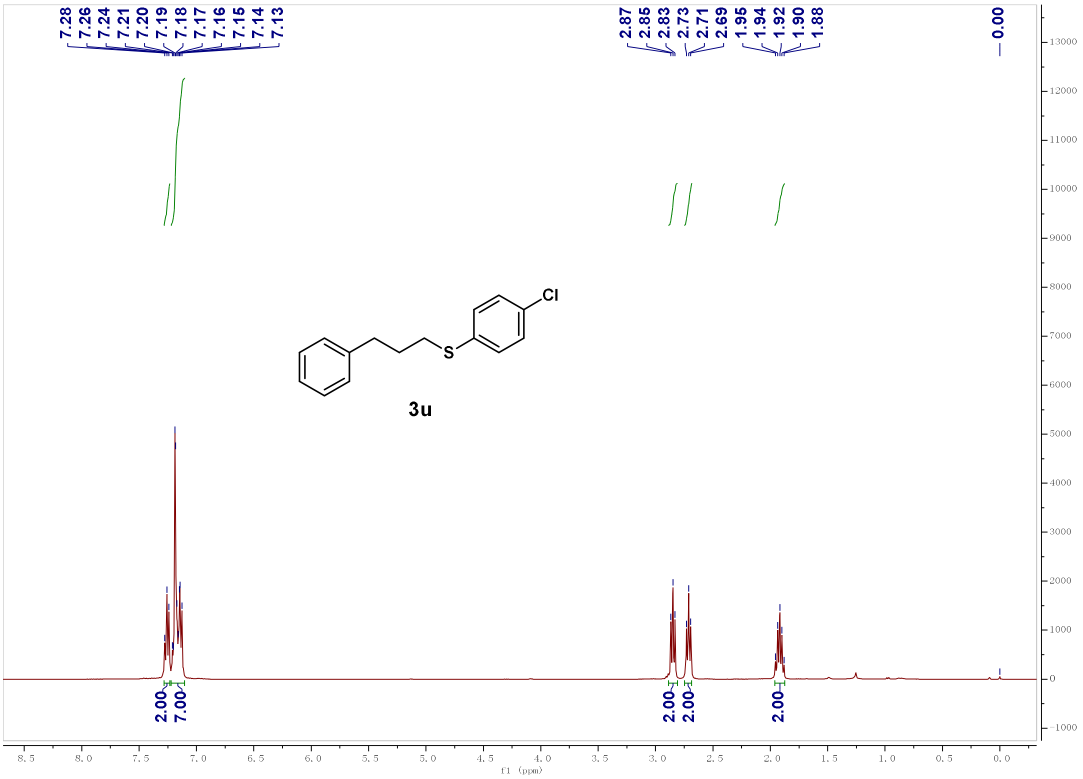

Supplementary Figure 44. ^{1}H NMR spectra for 3u

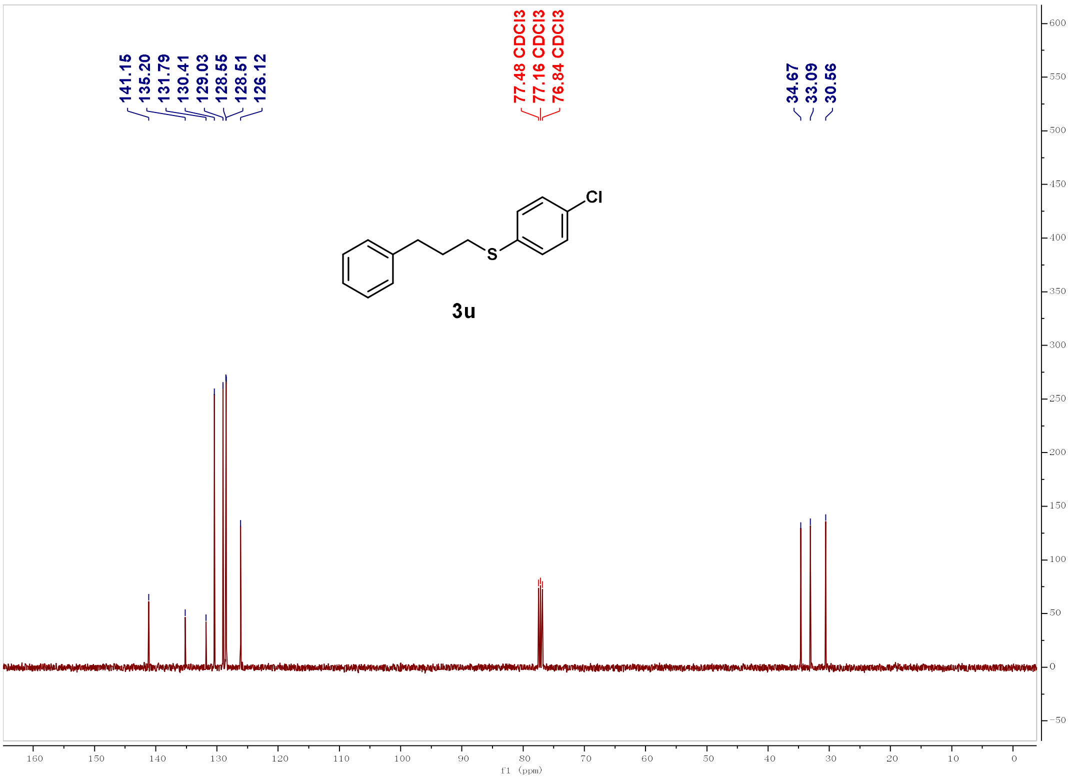

Supplementary Figure 45. ^{13}C NMR spectra for 3u

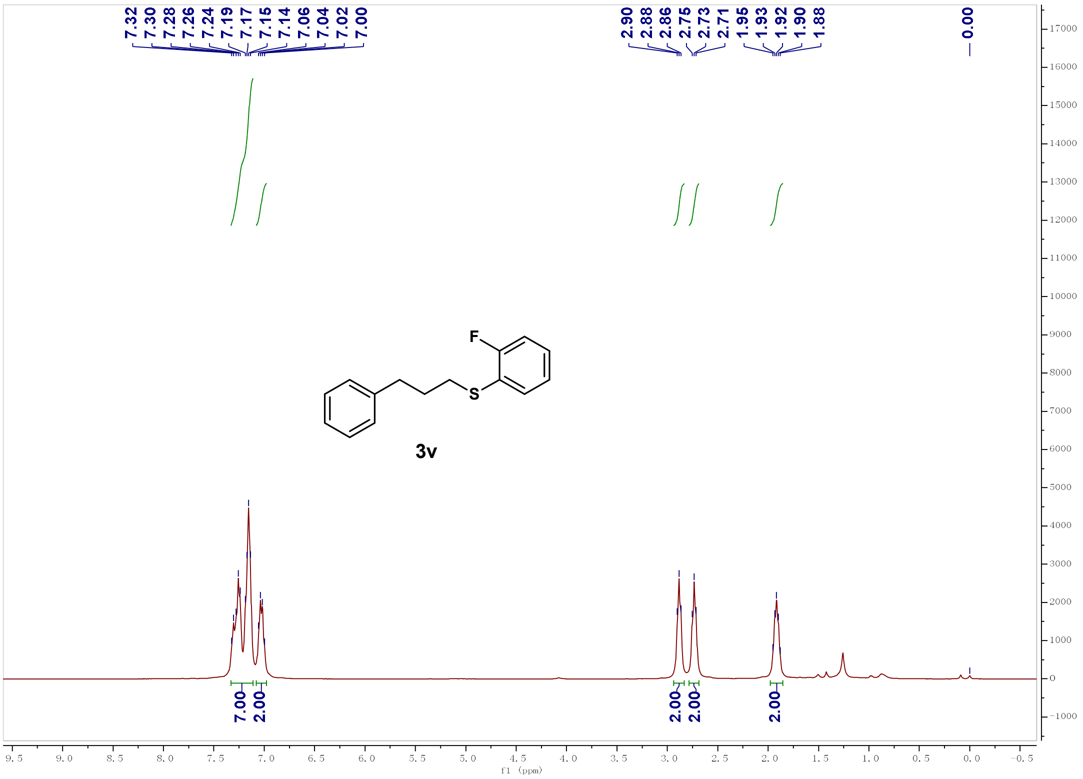

Supplementary Figure 46. ^{1}H NMR spectra for 3v

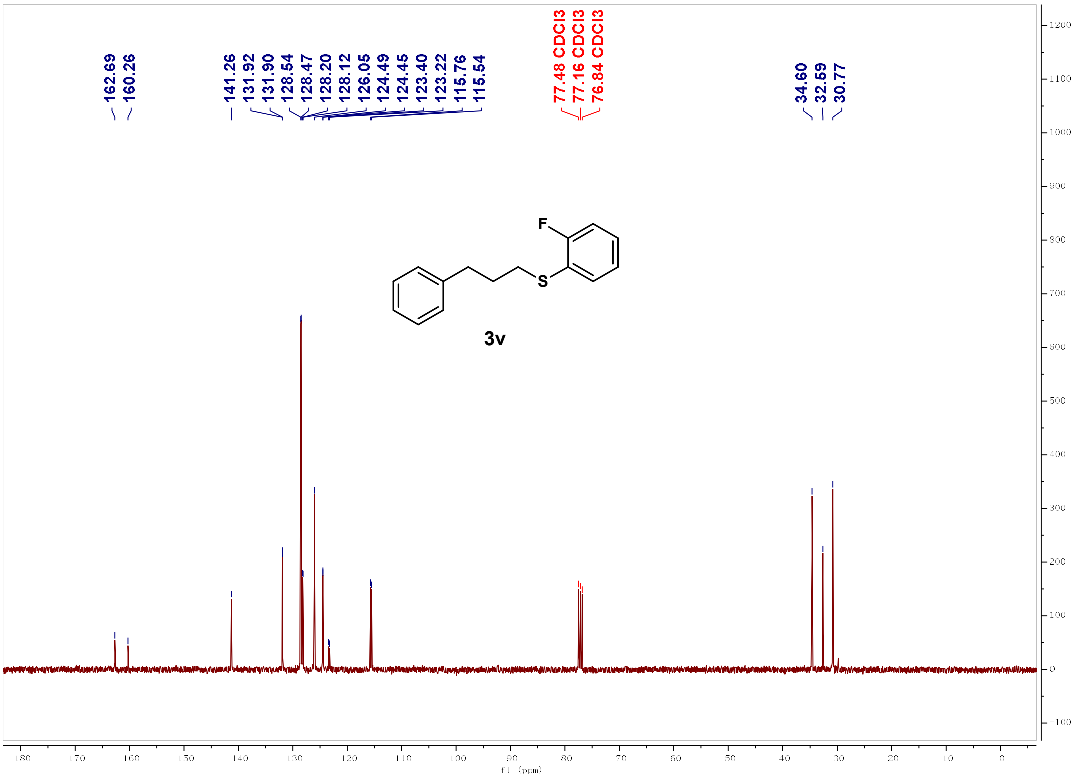

Supplementary Figure 47. ^{13}C NMR spectra for 3v

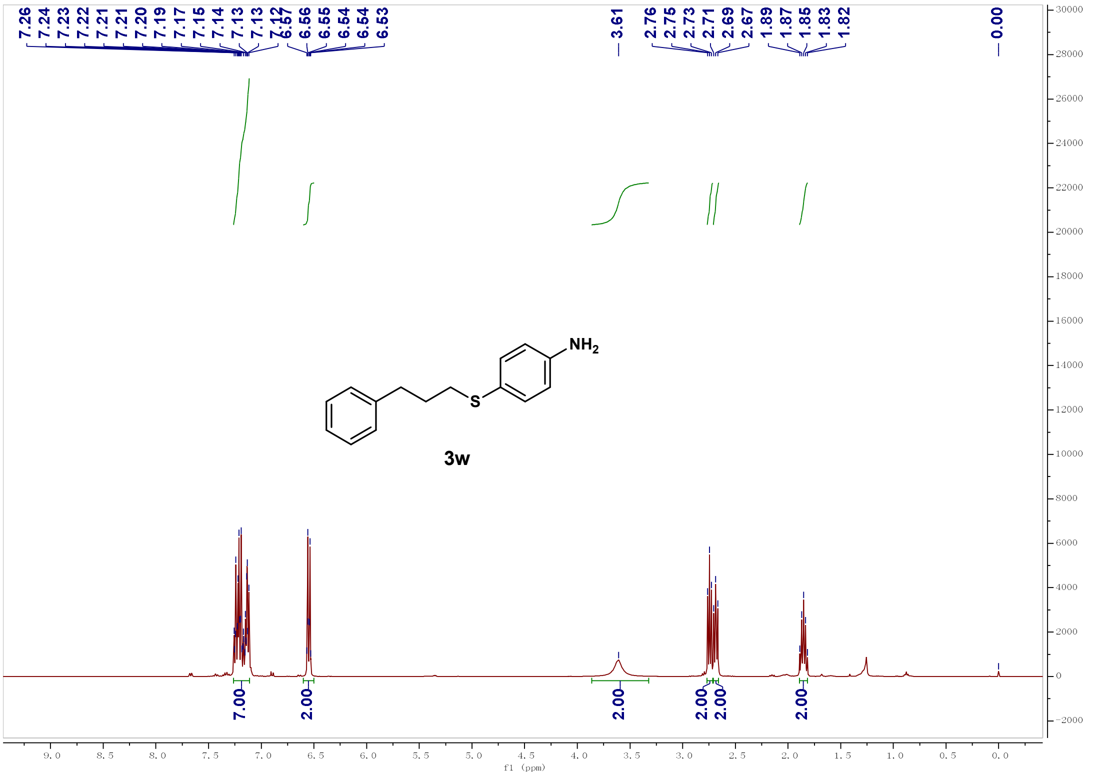

Supplementary Figure 48. ^{1}H NMR spectra for 3w

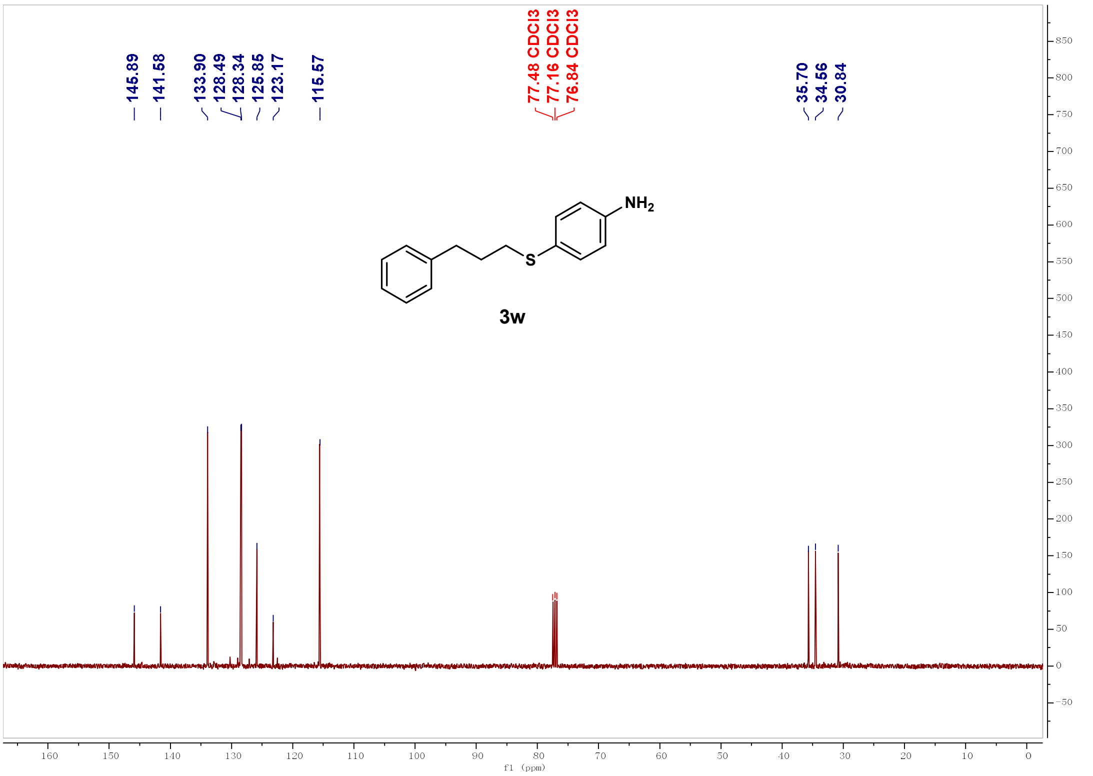

Supplementary Figure 49. ^{13}C NMR spectra for 3w

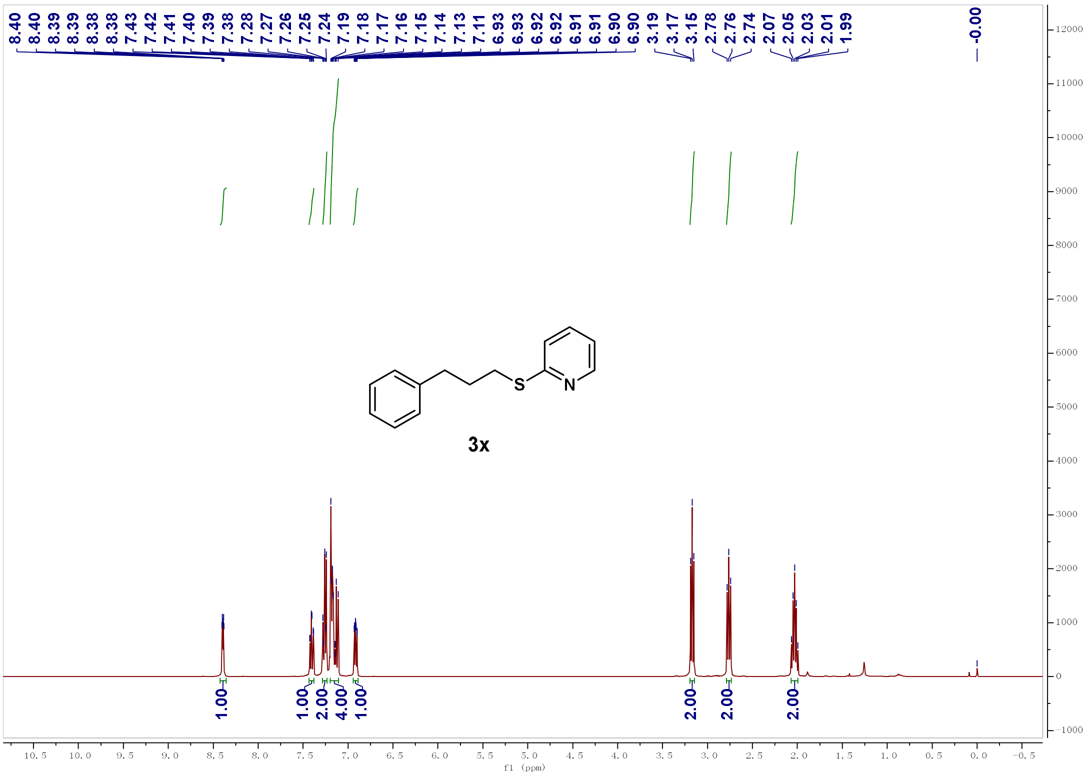

Supplementary Figure 50. ^{1}**H NMR spectra for 3x**

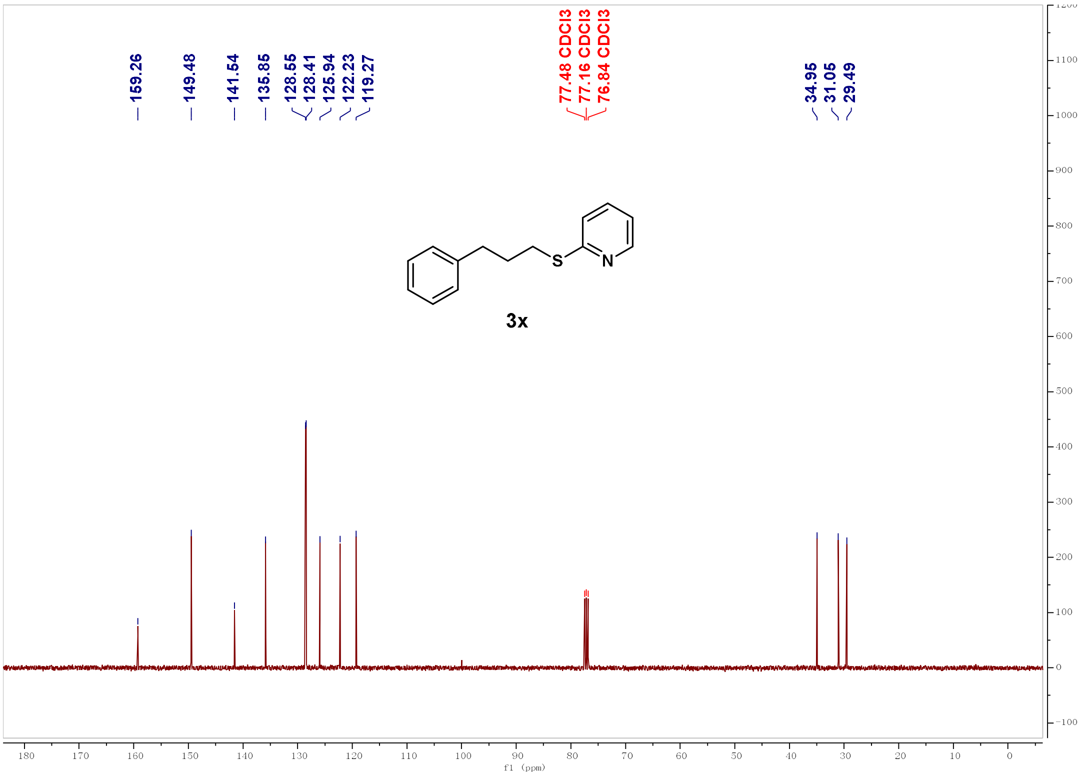

Supplementary Figure 51. ^{13}**C NMR spectra for 3x**

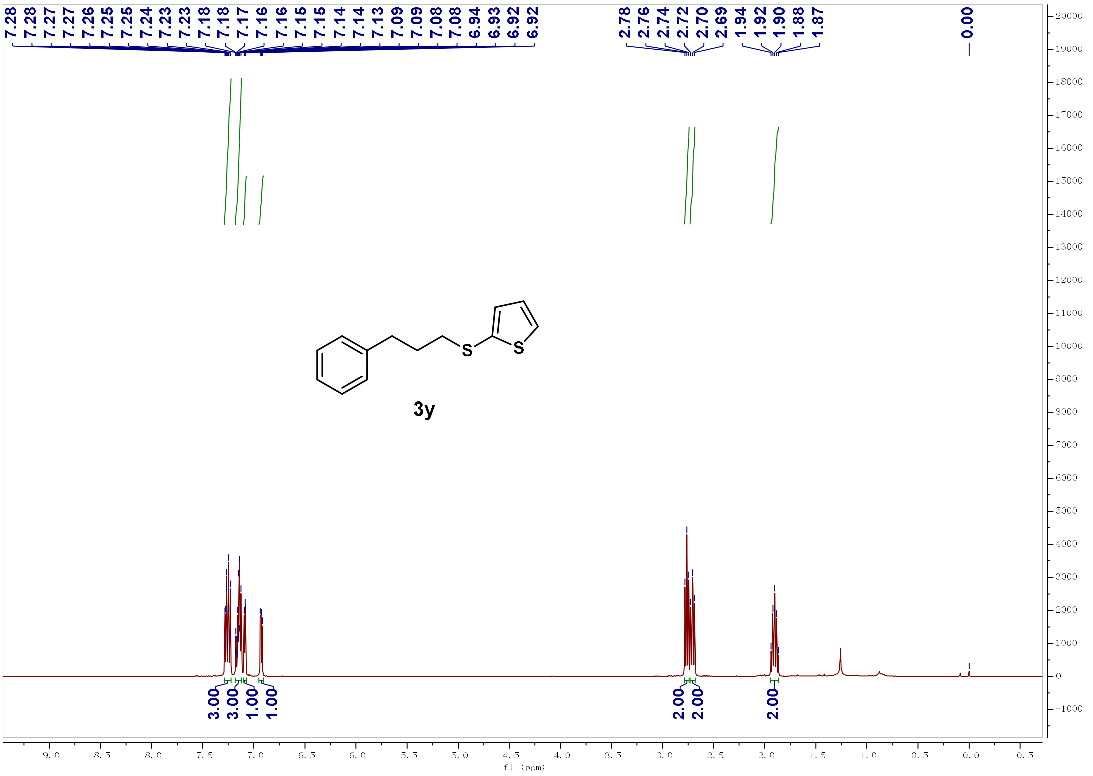

Supplementary Figure 52. ^{1}**H NMR spectra for 3y**

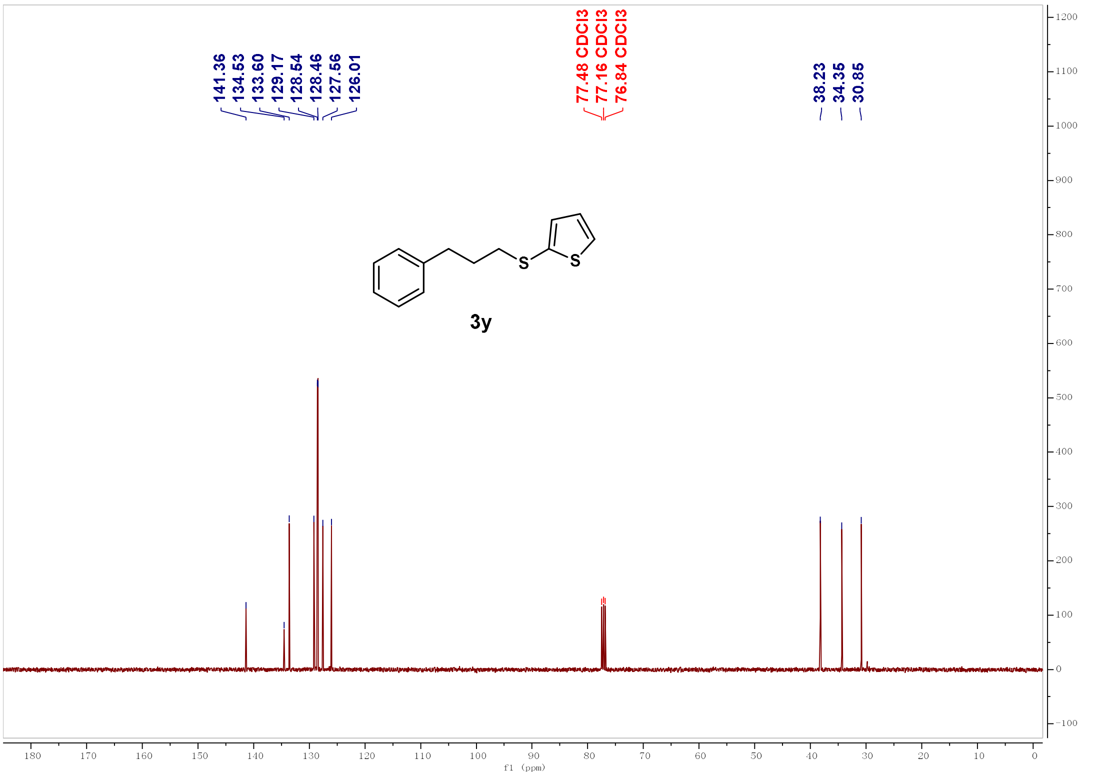

Supplementary Figure 53. ^{13}**C NMR spectra for 3y**

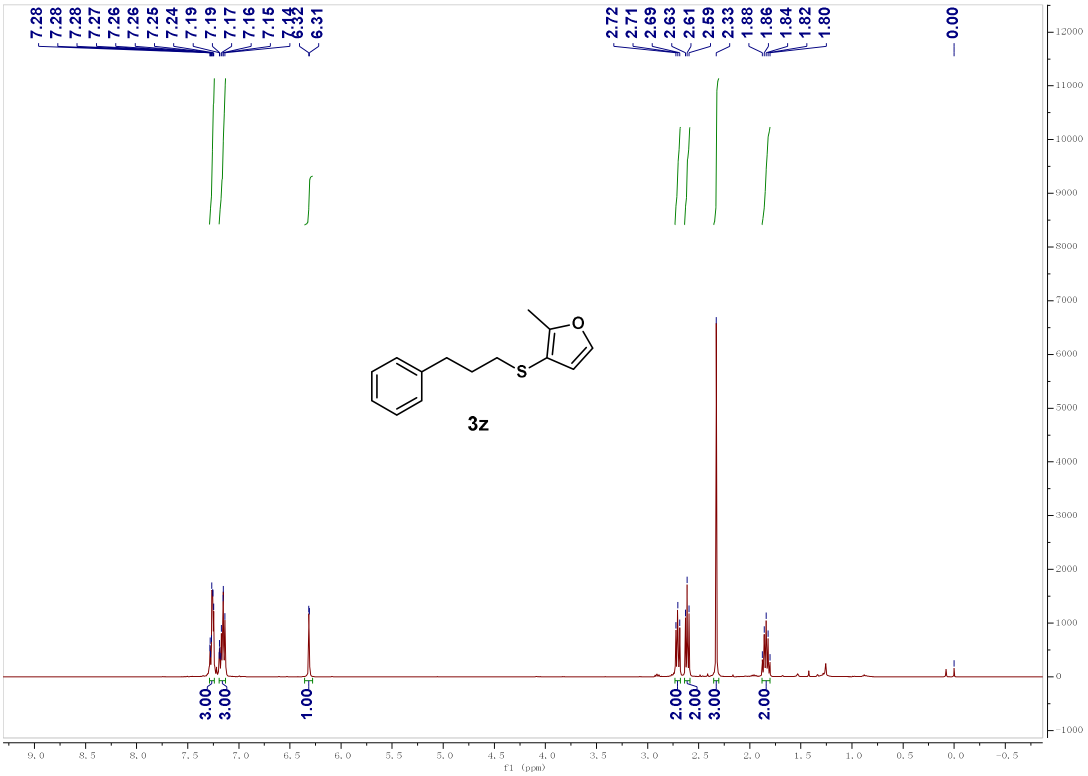

Supplementary Figure 54. ^{1}H NMR spectra for 3z

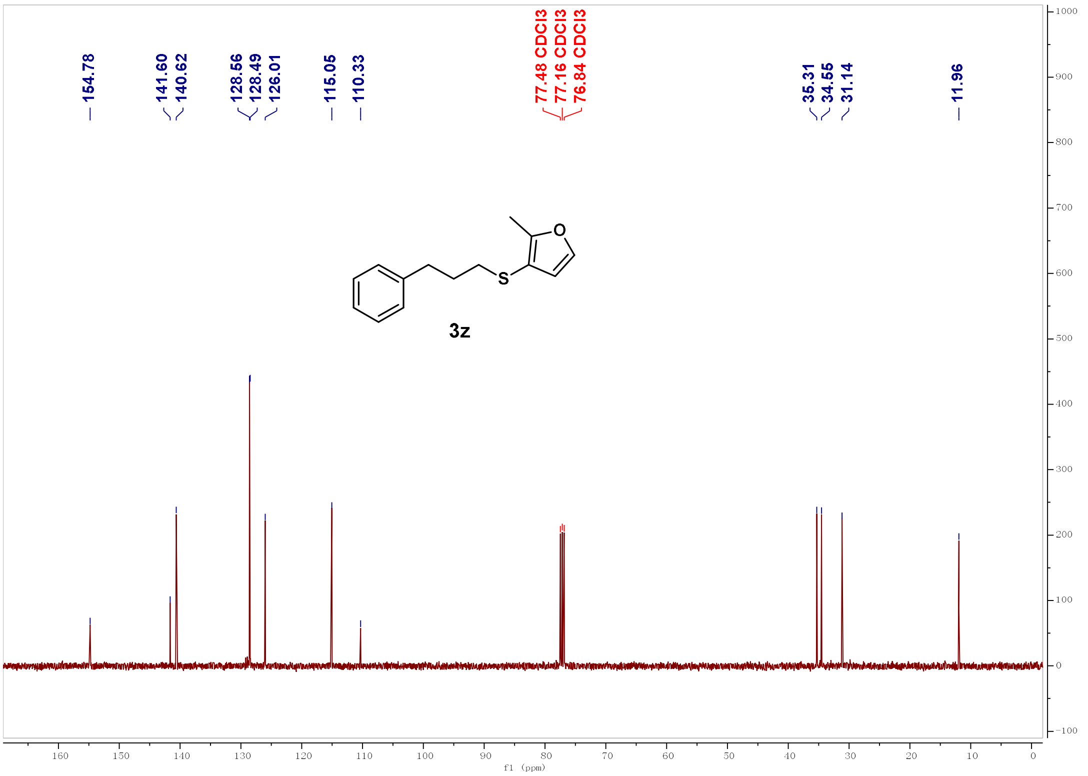

Supplementary Figure 55. ^{13}C NMR spectra for 3z

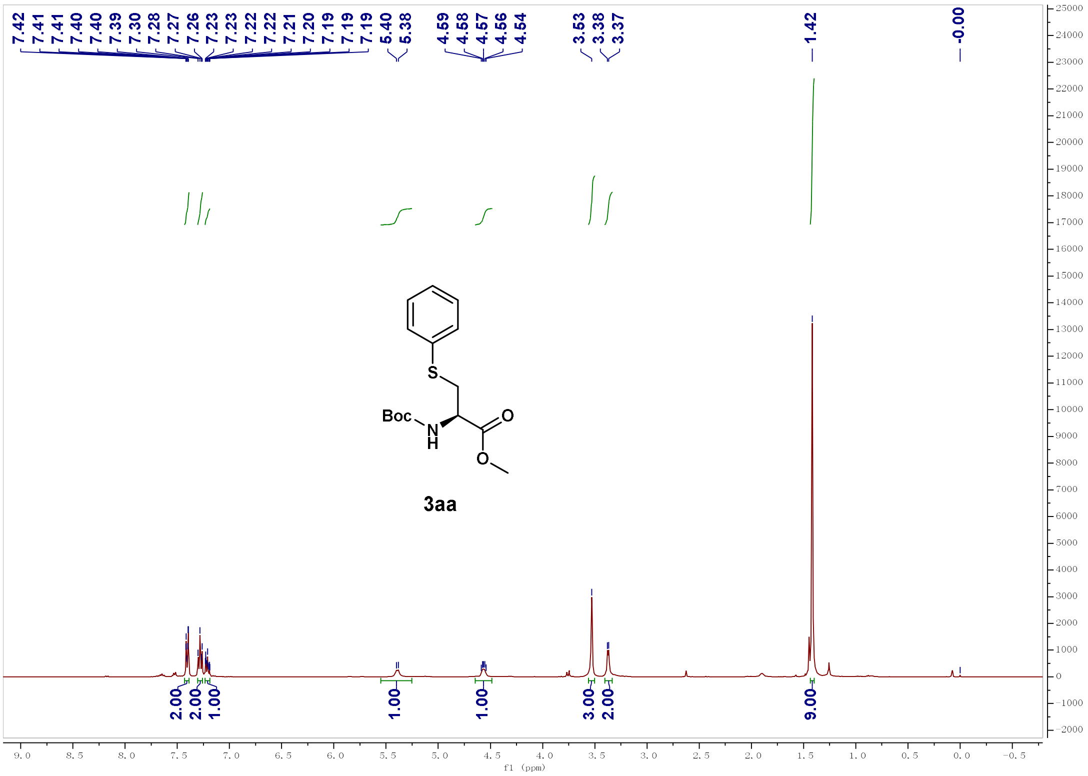

Supplementary Figure 56. ^{1}**H NMR spectra for 3aa**

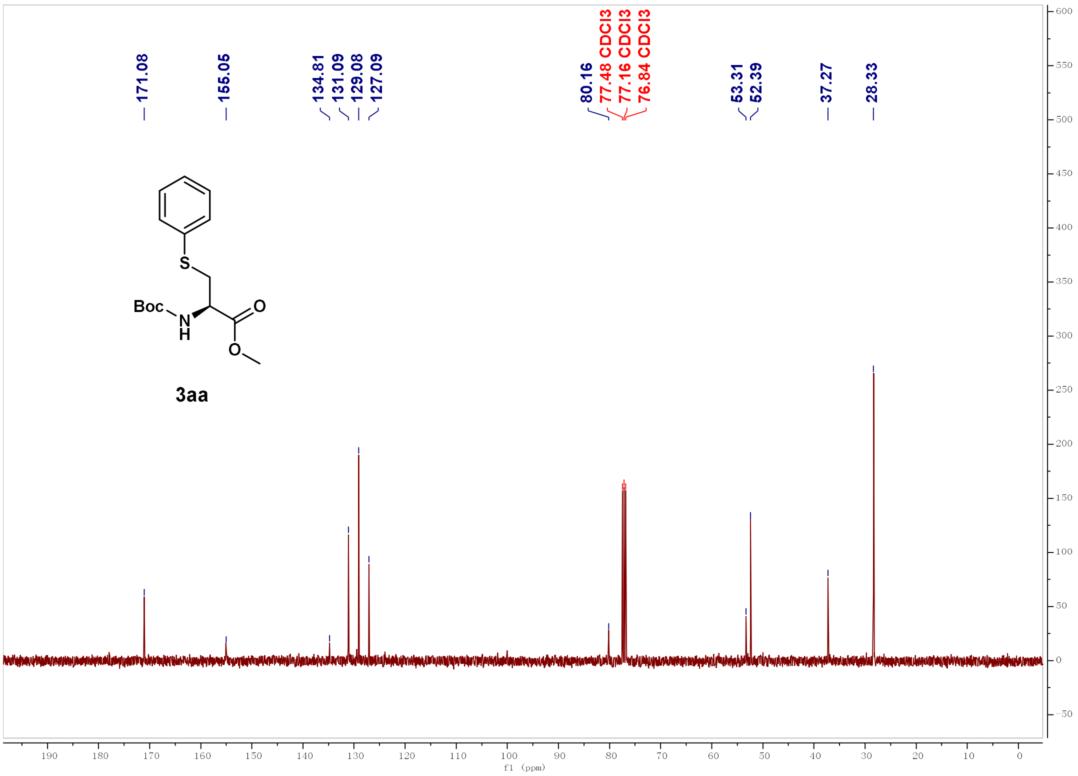

Supplementary Figure 57. ^{13}**C NMR spectra for 3aa**

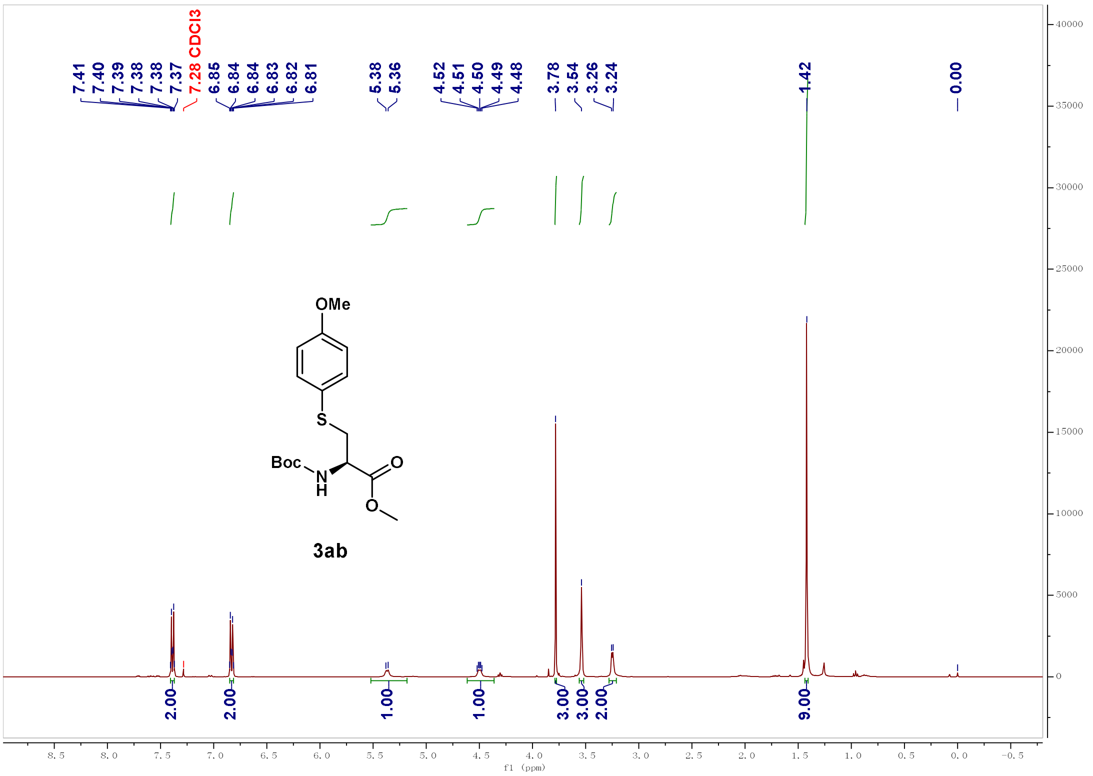

Supplementary Figure 58. ^{1}H NMR spectra for 3ab

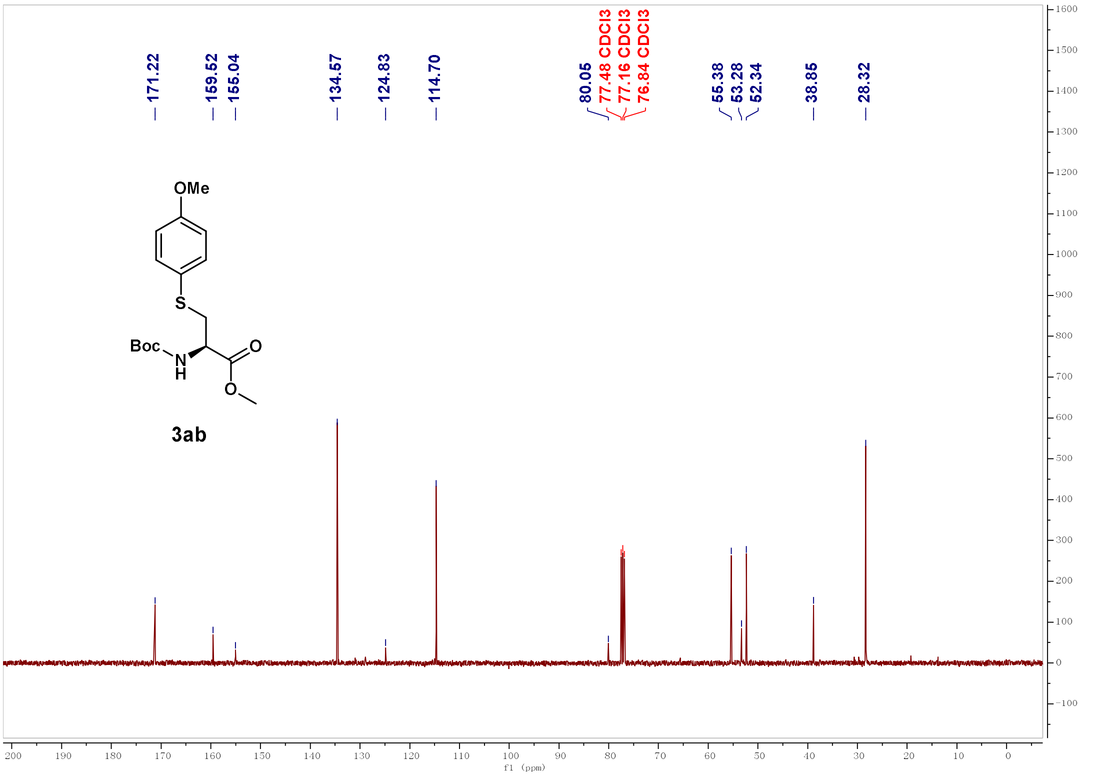

Supplementary Figure 59. ^{13}C NMR spectra for 3ab

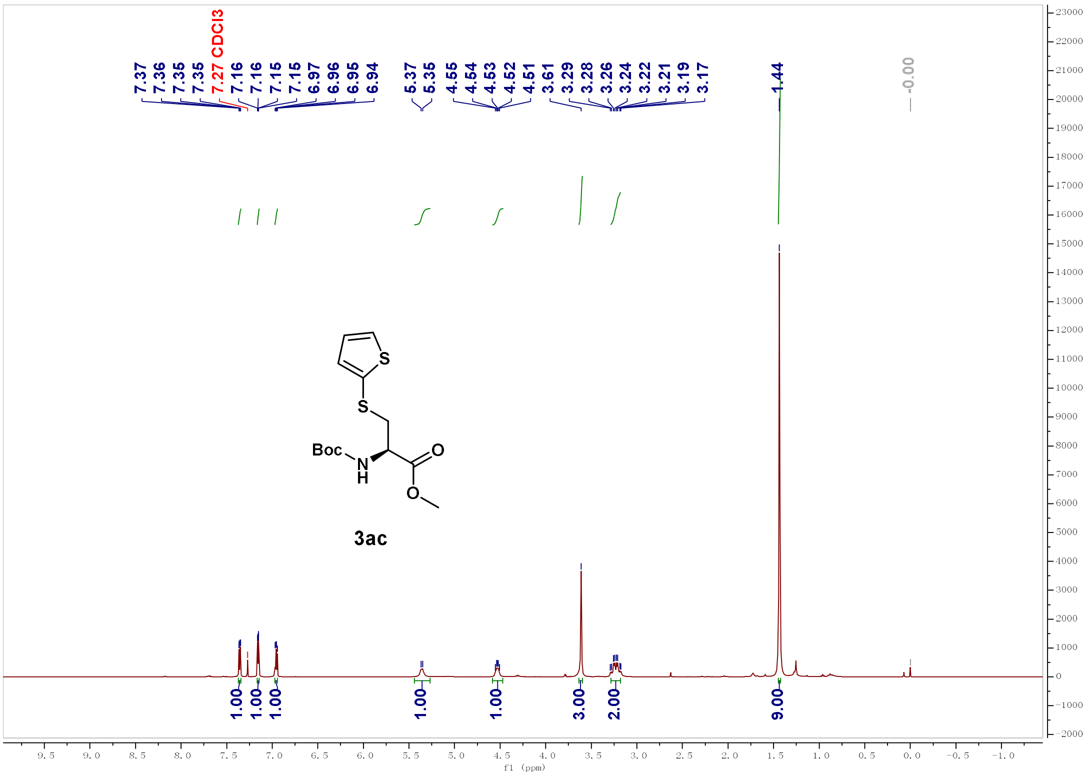

Supplementary Figure 60. ^{1}**H NMR spectra for 3ac**

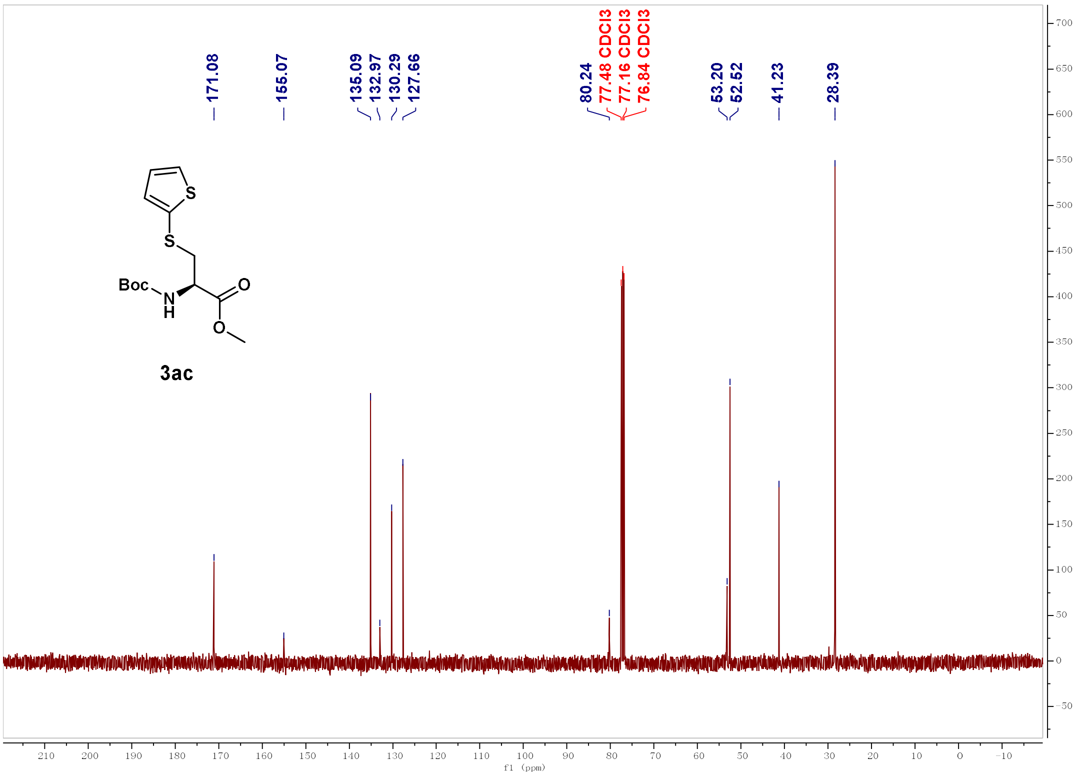

Supplementary Figure 61. ^{13}**C NMR spectra for 3ac**

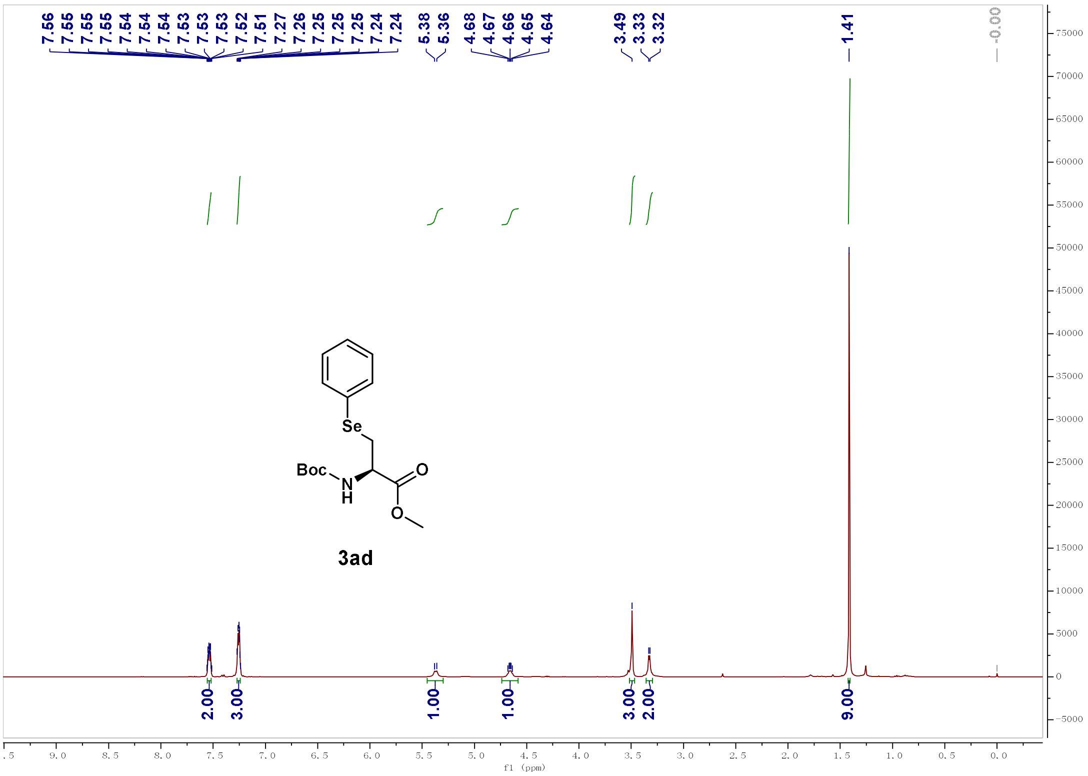

Supplementary Figure 62. ^{1}**H NMR spectra for 3ad**

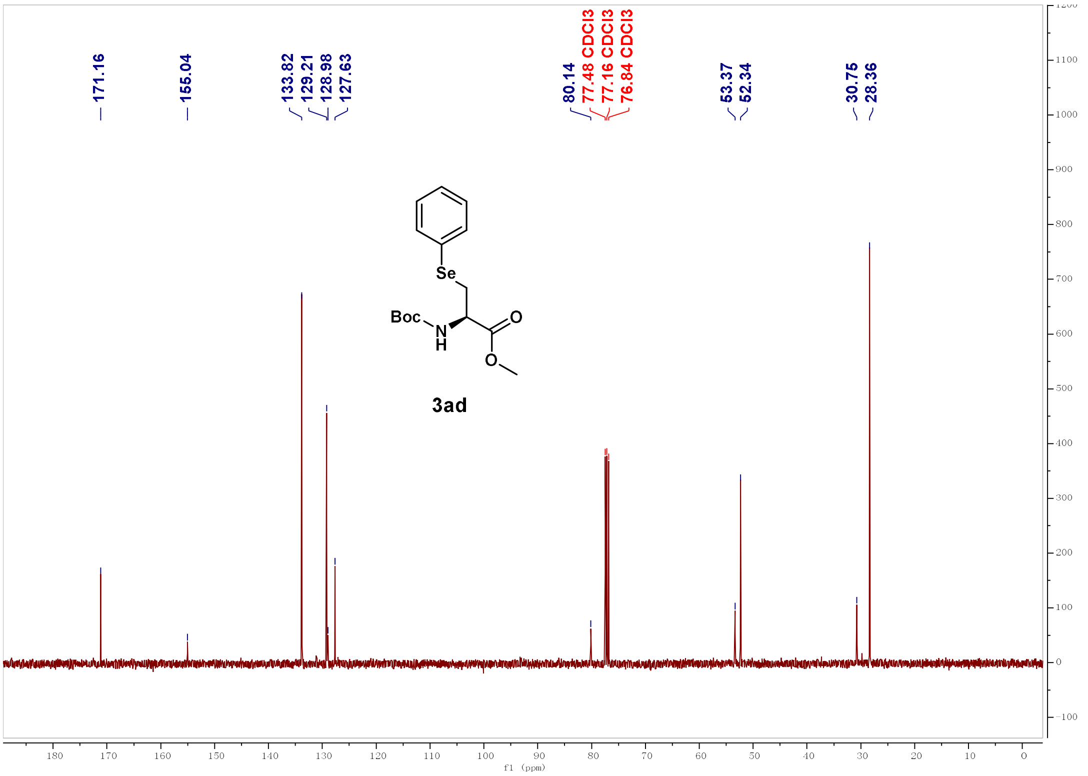

Supplementary Figure 63. ^{13}**C NMR spectra for 3ad**

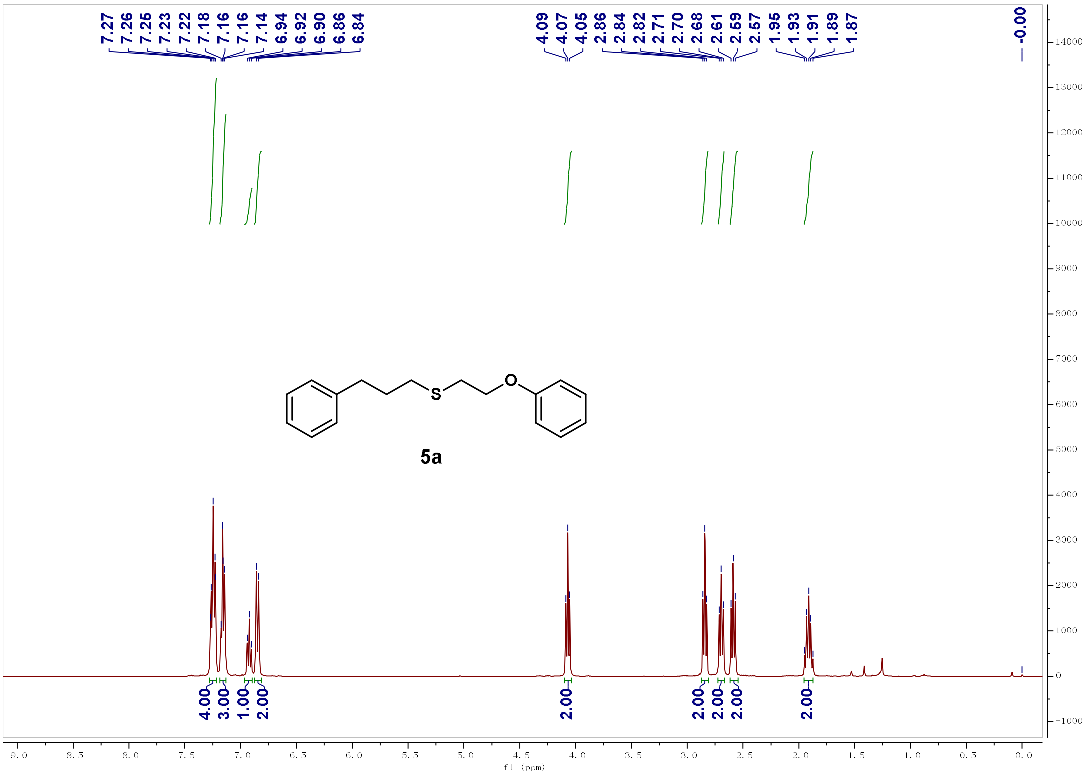

Supplementary Figure 64. ^{1}**H NMR spectra for 5a**

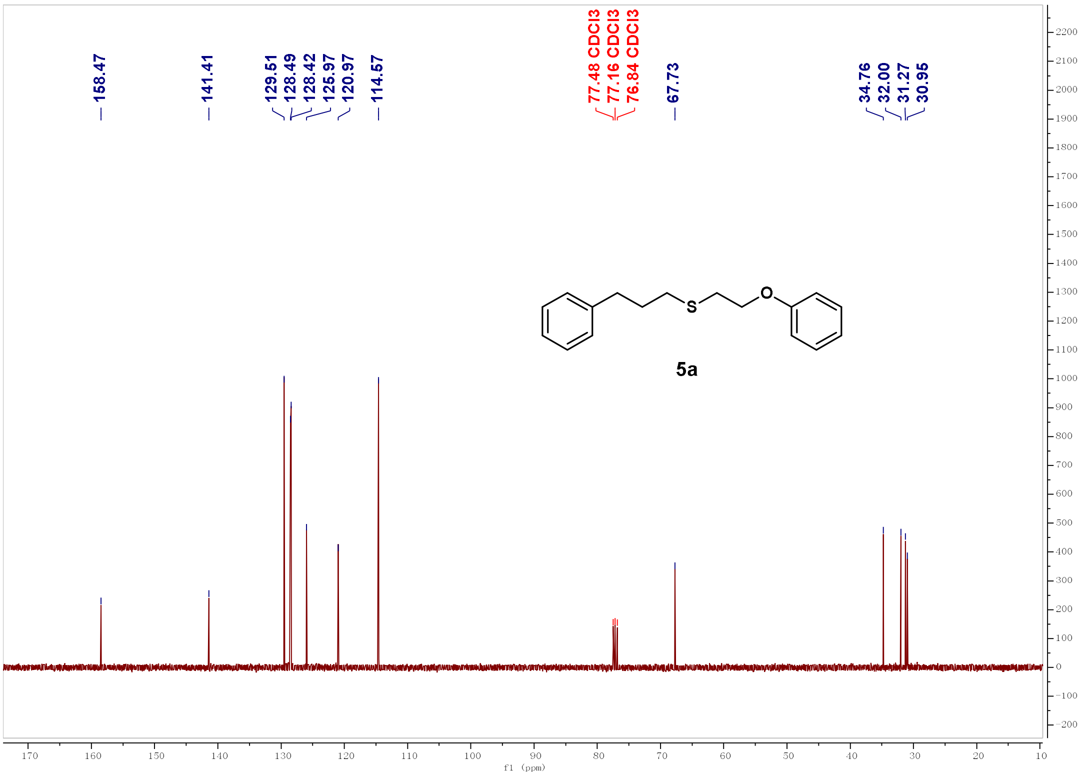

Supplementary Figure 65. ^{13}**C NMR spectra for 5a**

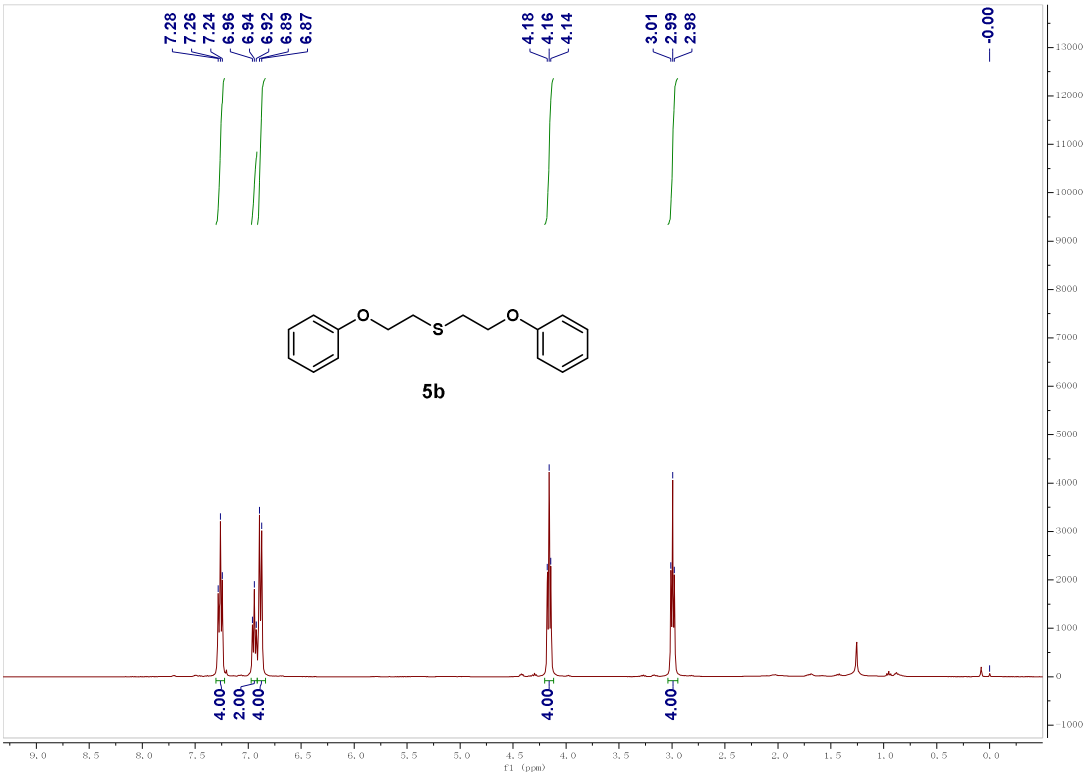

Supplementary Figure 66. ^{1}H NMR spectra for 5b

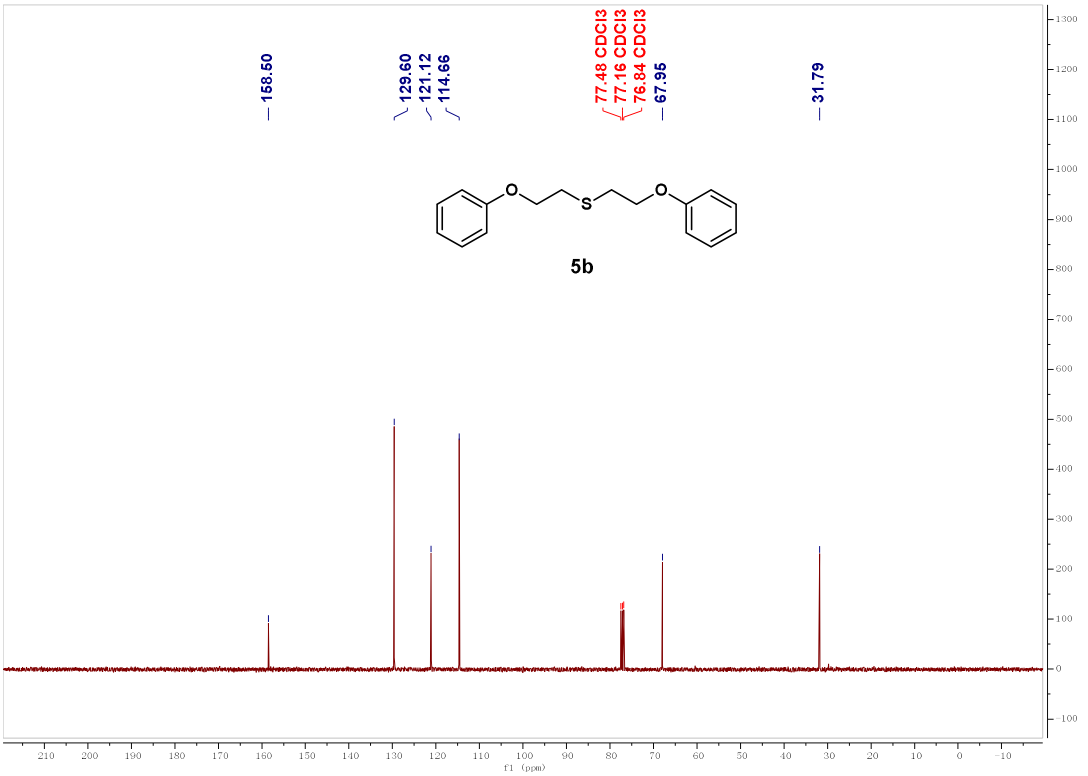

Supplementary Figure 67. ^{13}C NMR spectra for 5b

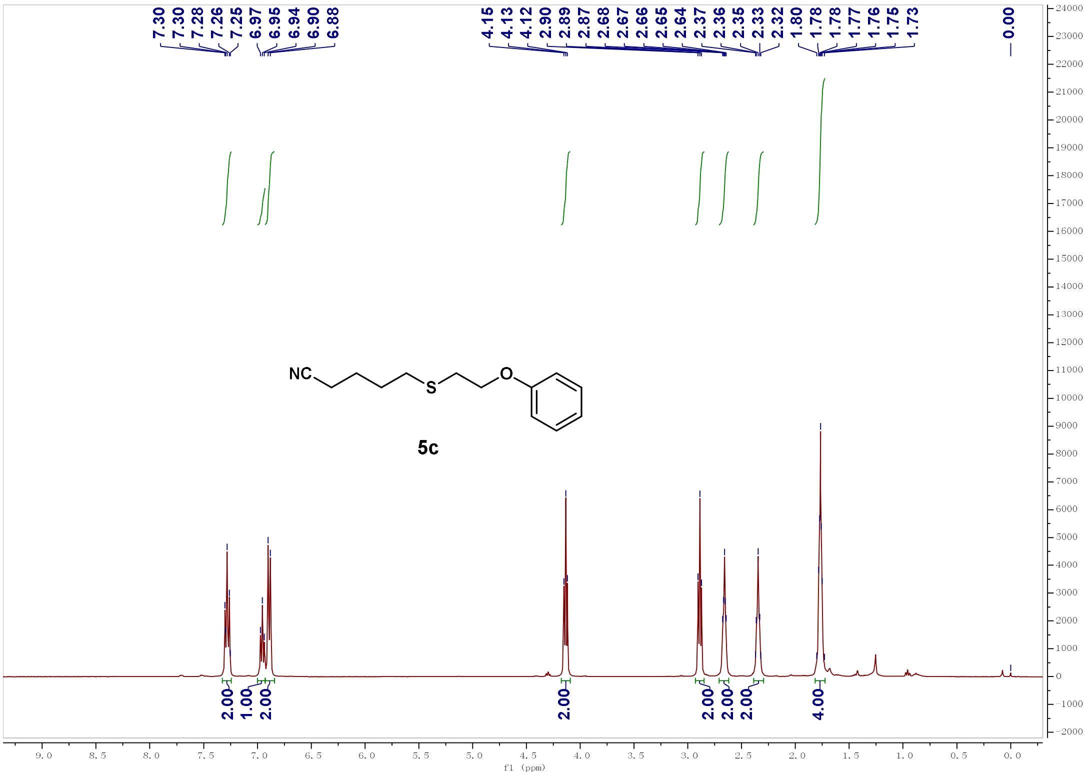

Supplementary Figure 68. ^{1}H NMR spectra for 5c

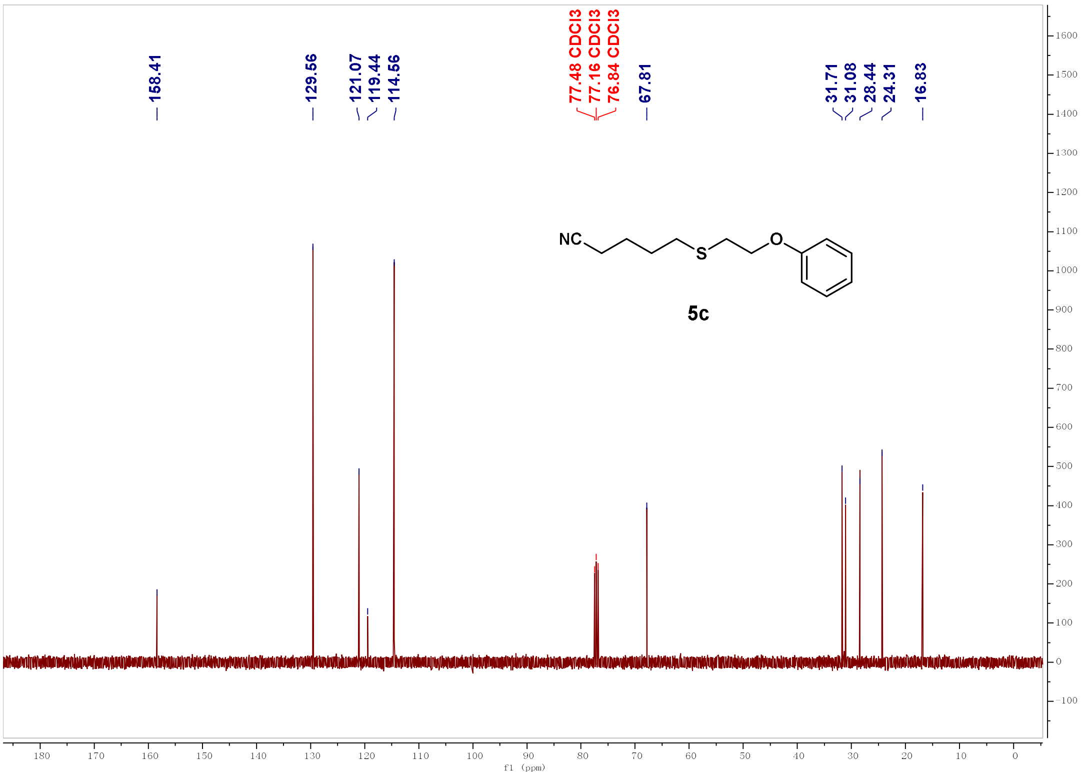

Supplementary Figure 69. ^{13}C NMR spectra for 5c

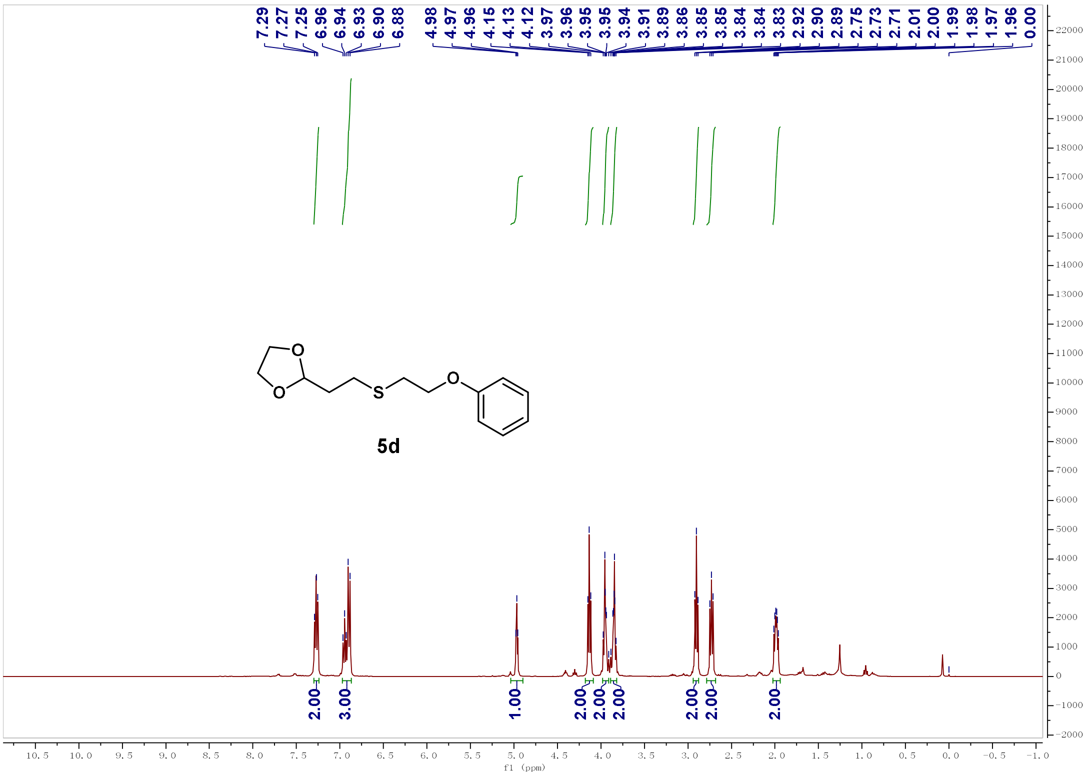

Supplementary Figure 70. **^{1}H NMR spectra for 5d**

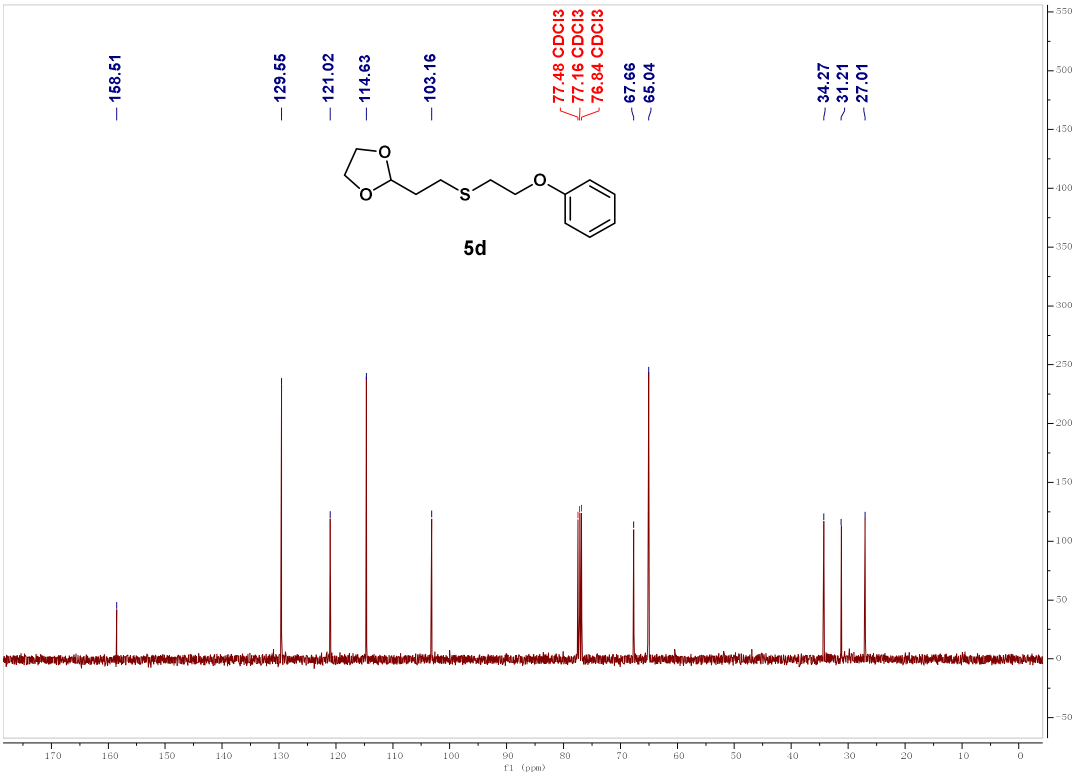

Supplementary Figure 71. **^{13}C NMR spectra for 5d**

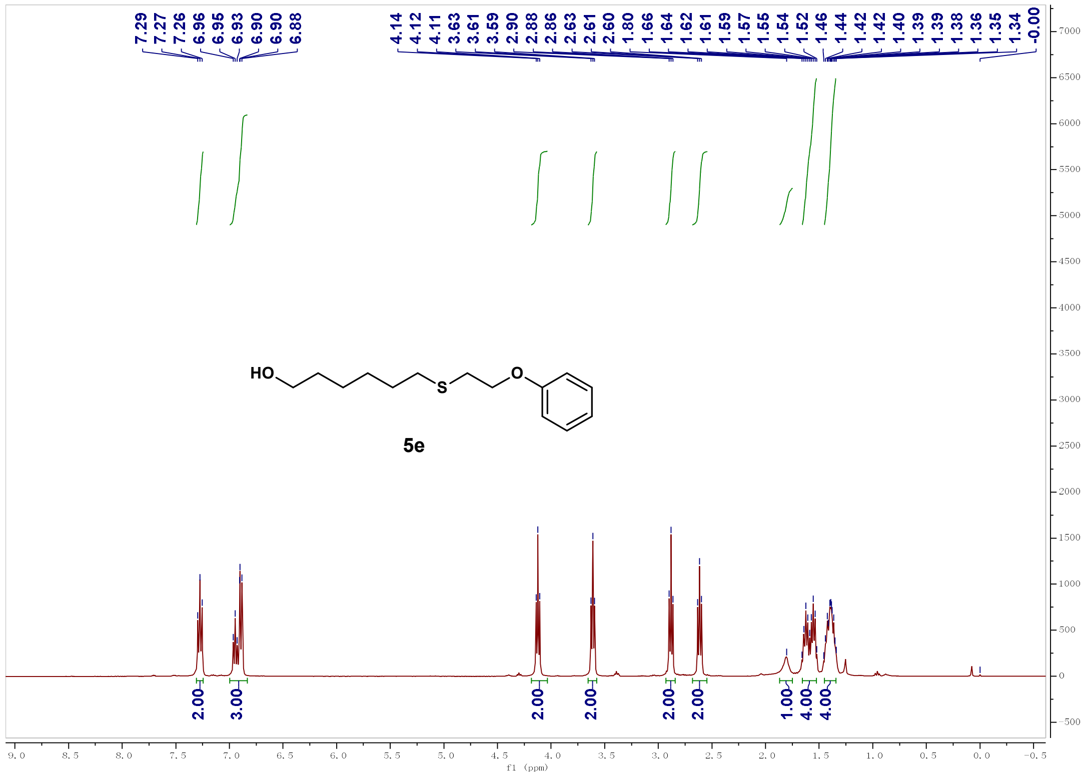

Supplementary Figure 72. ^{1}**H NMR spectra for 5e**

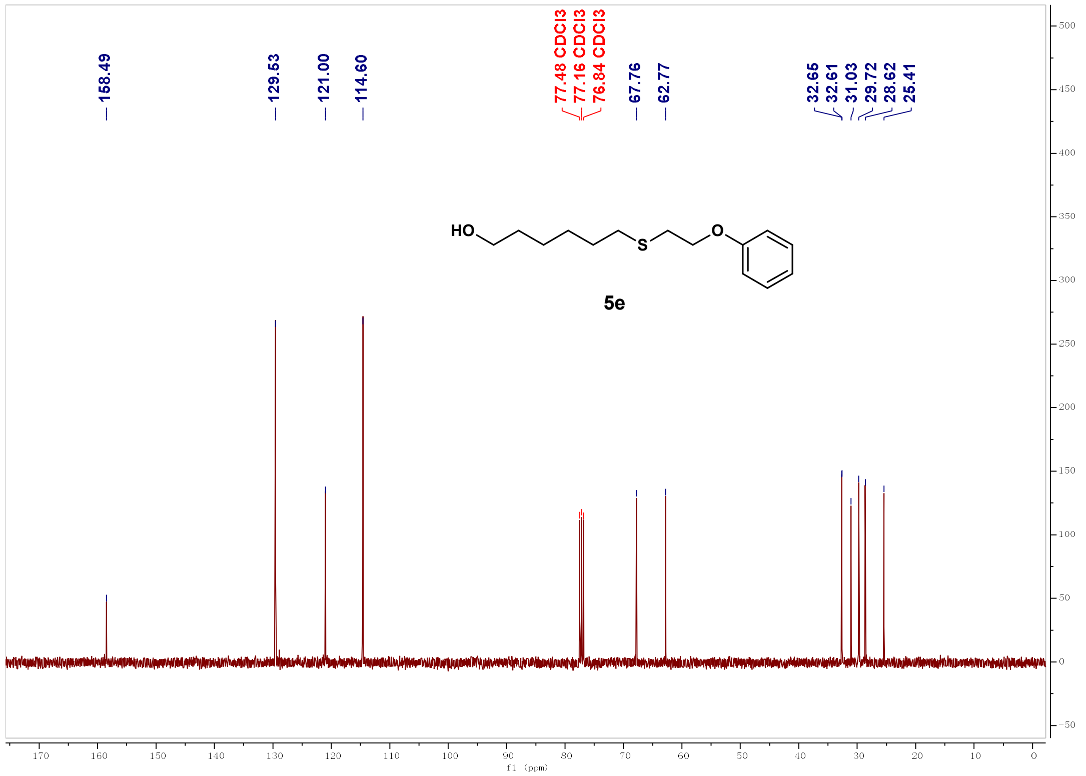

Supplementary Figure 73. ^{13}**C NMR spectra for 5e**

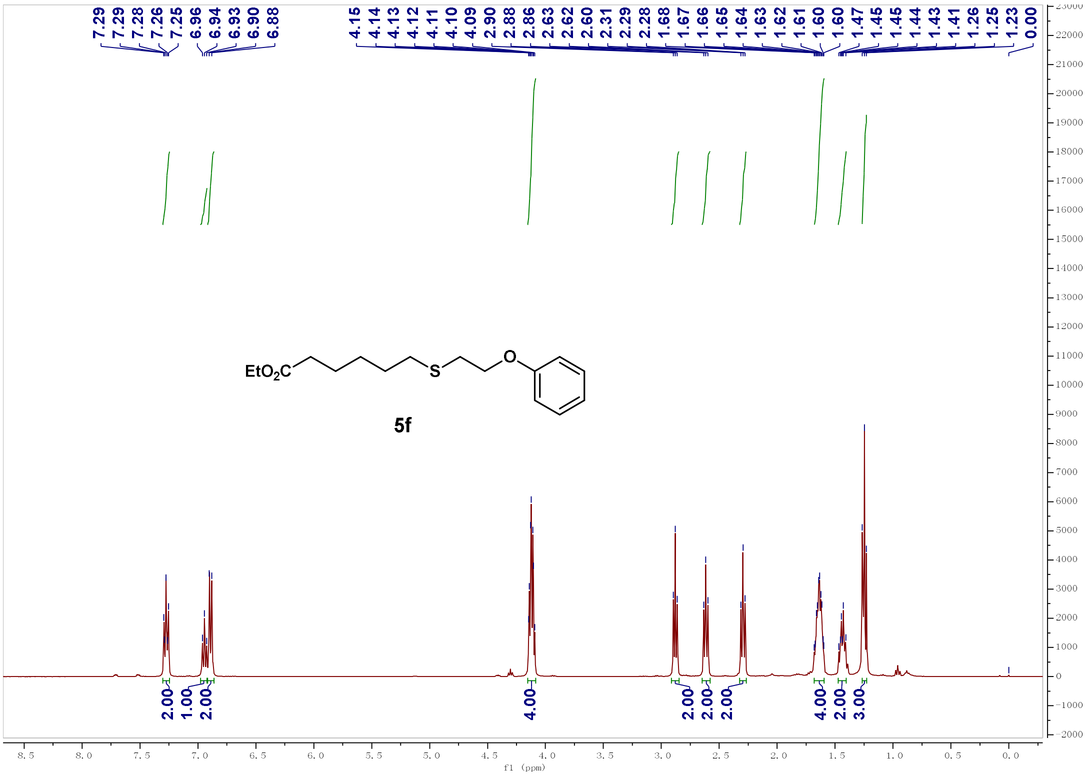

Supplementary Figure 74. ^{1}H NMR spectra for 5f

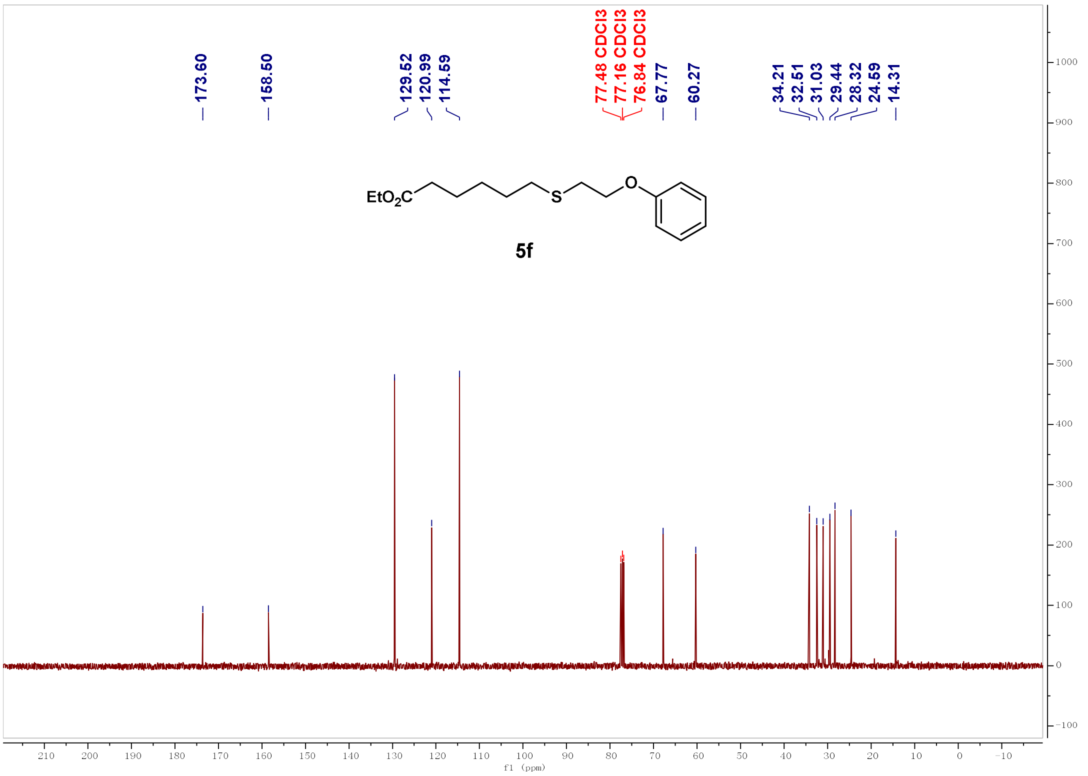

Supplementary Figure 75. ^{13}C NMR spectra for 5f

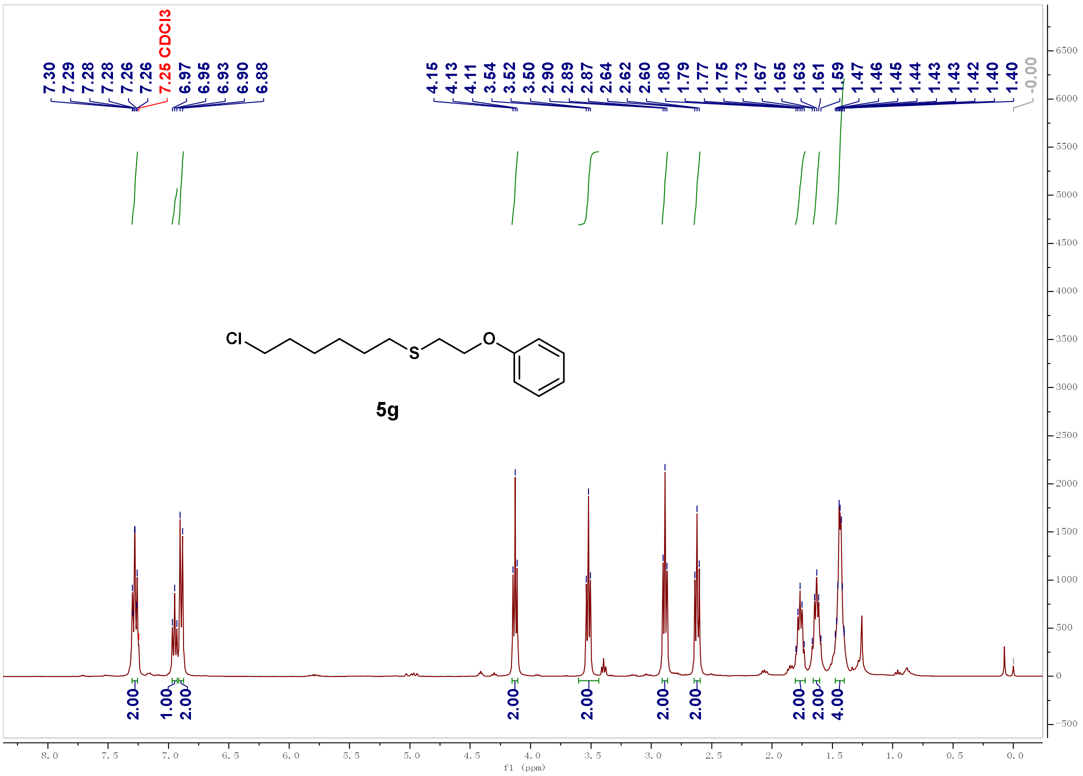

Supplementary Figure 76. ^{1}H NMR spectra for 5g

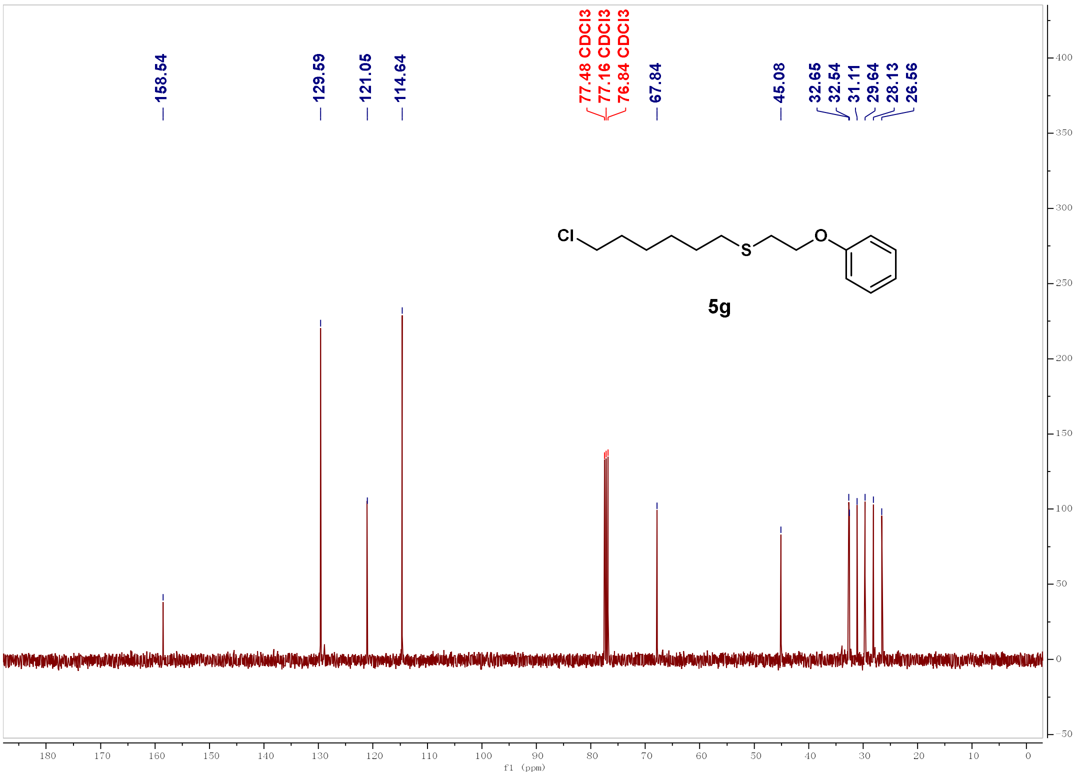

Supplementary Figure 77. ^{13}C NMR spectra for 5g

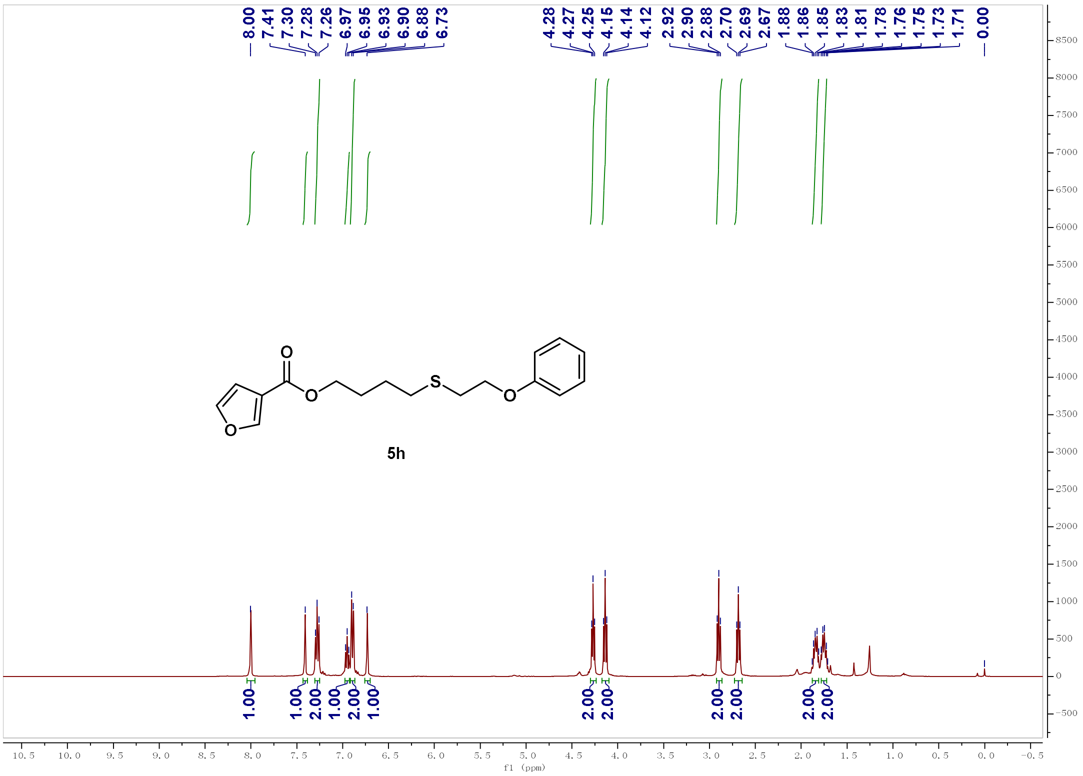

Supplementary Figure 78. ^{1}H NMR spectra for 5h

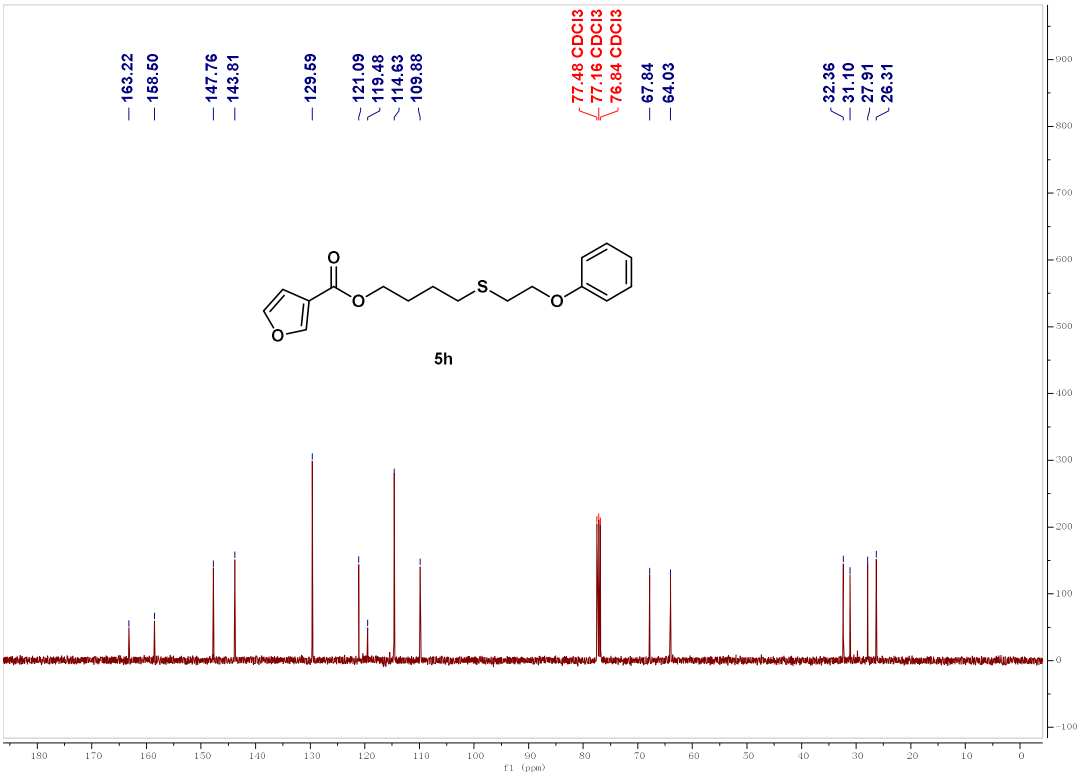

Supplementary Figure 79. ^{13}C NMR spectra for 5h

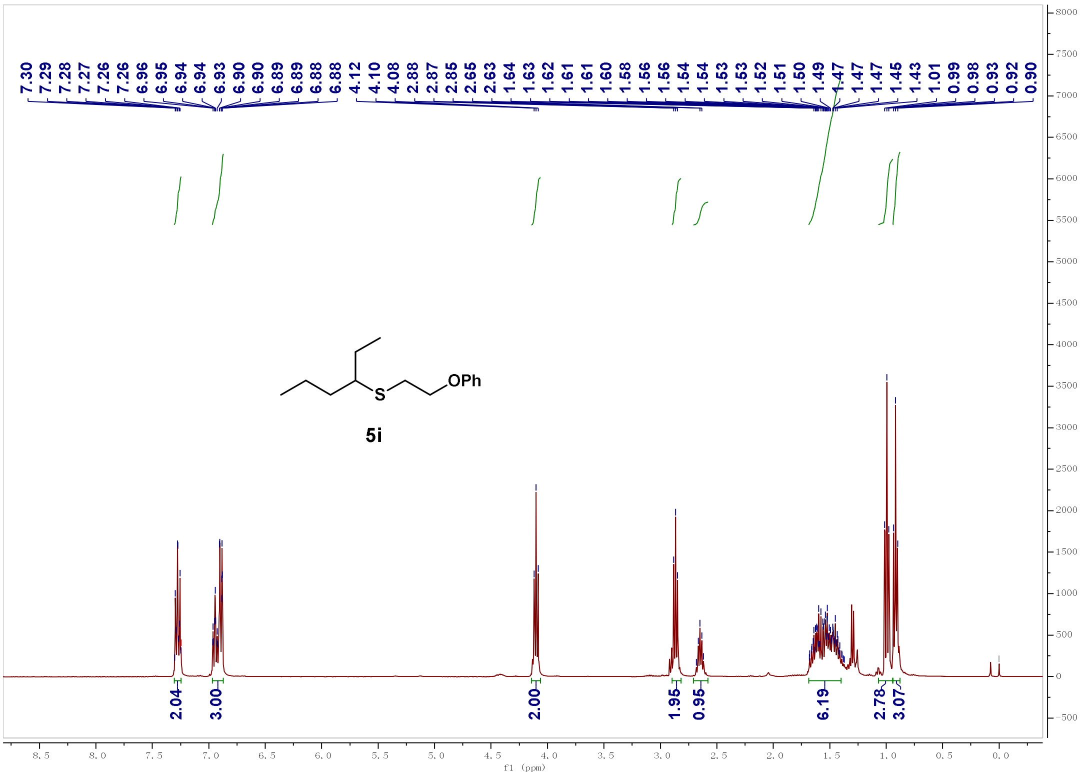

Supplementary Figure 80. ^{1}**H NMR spectra for 5i**

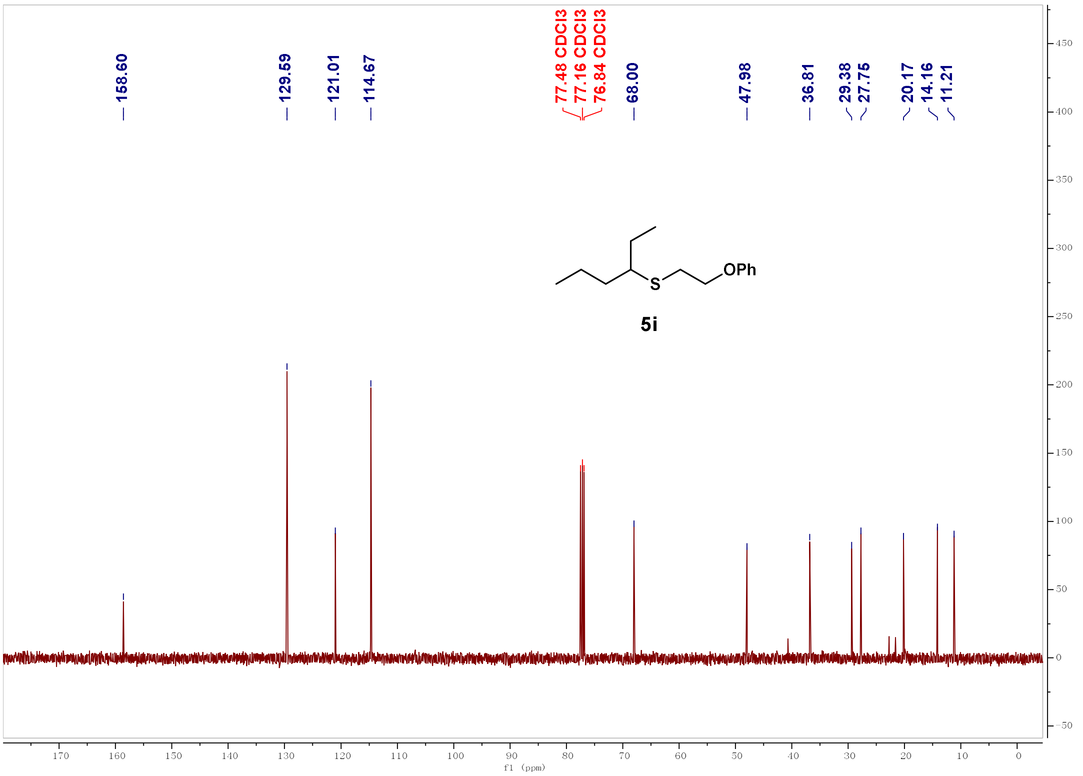

Supplementary Figure 81. ^{13}**C NMR spectra for 5i**

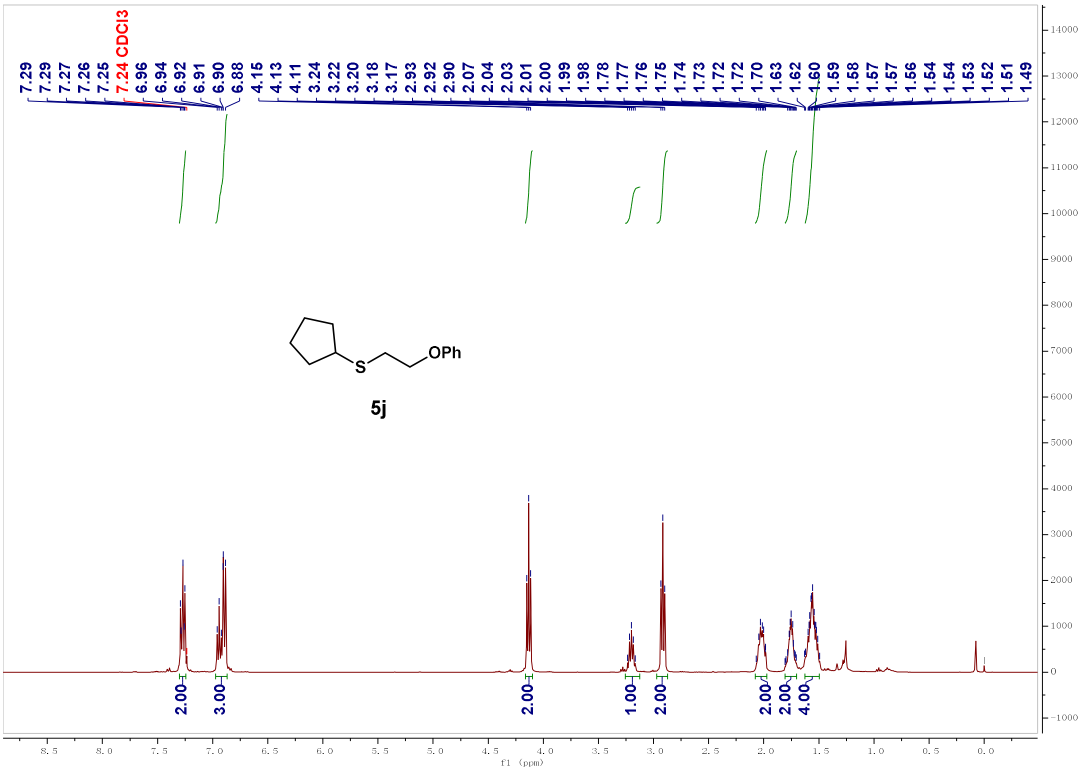

Supplementary Figure 82. ^{1}H NMR spectra for 5j

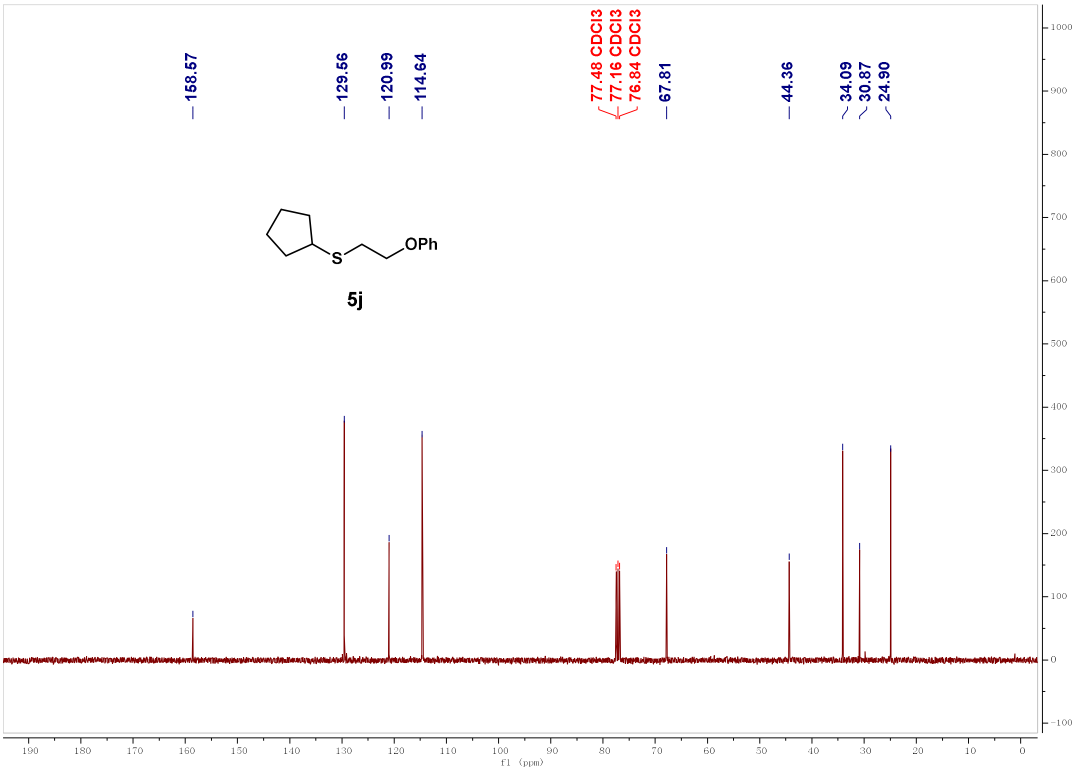

Supplementary Figure 83. ^{13}C NMR spectra for 5j

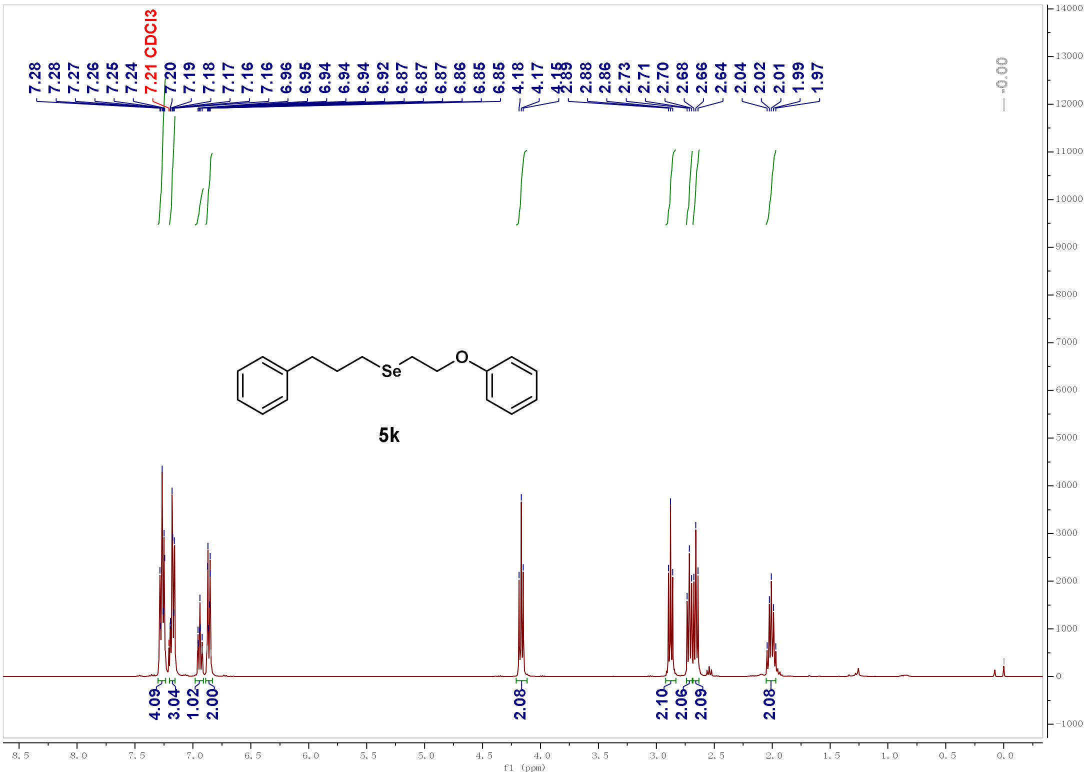

Supplementary Figure 84. ^{1}H NMR spectra for 5k

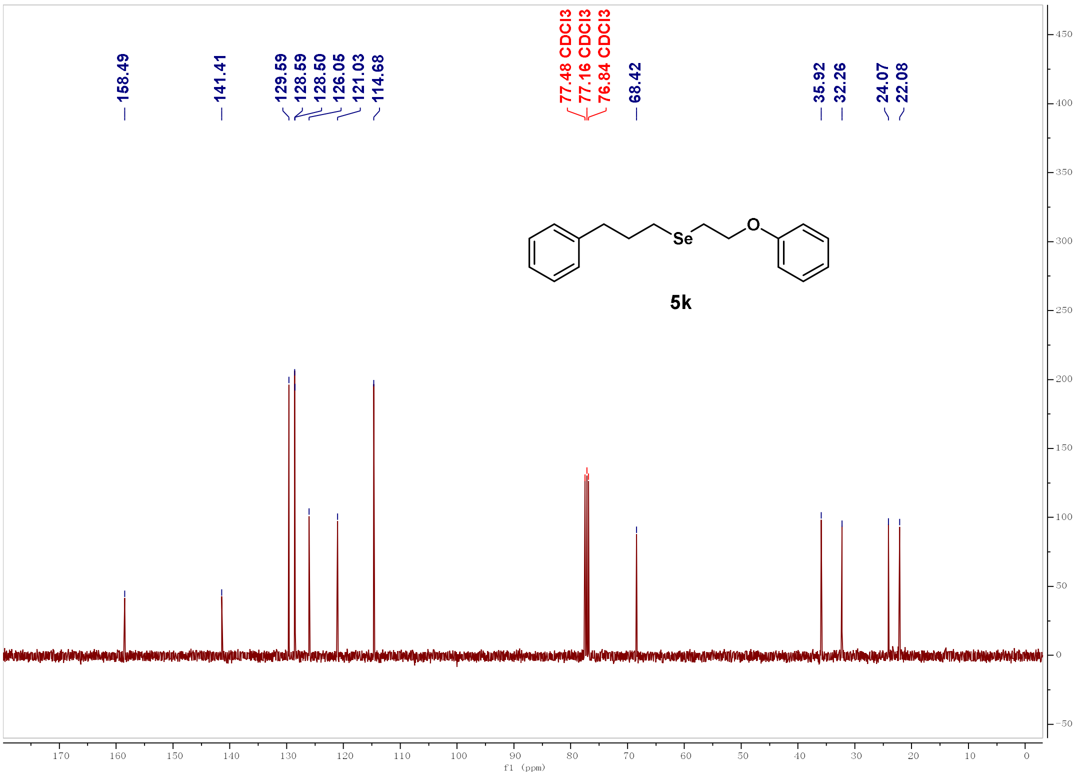

Supplementary Figure 85. ^{13}C NMR spectra for 5k

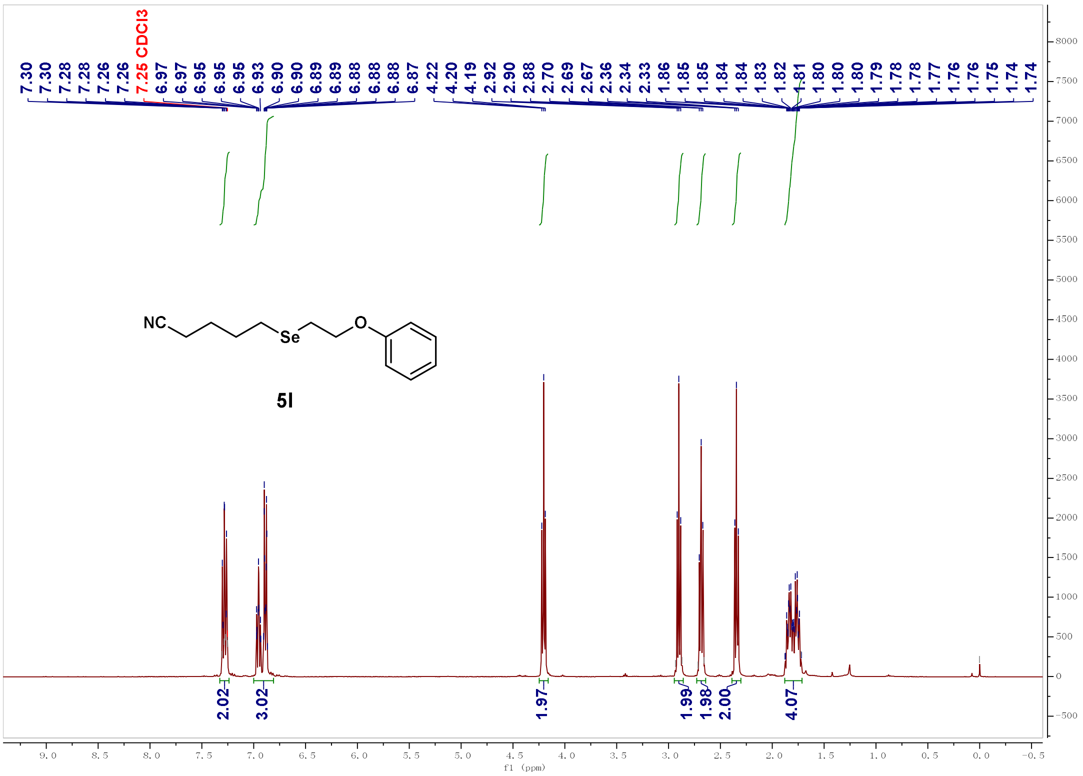

Supplementary Figure 86. ^{1}H NMR spectra for 5l

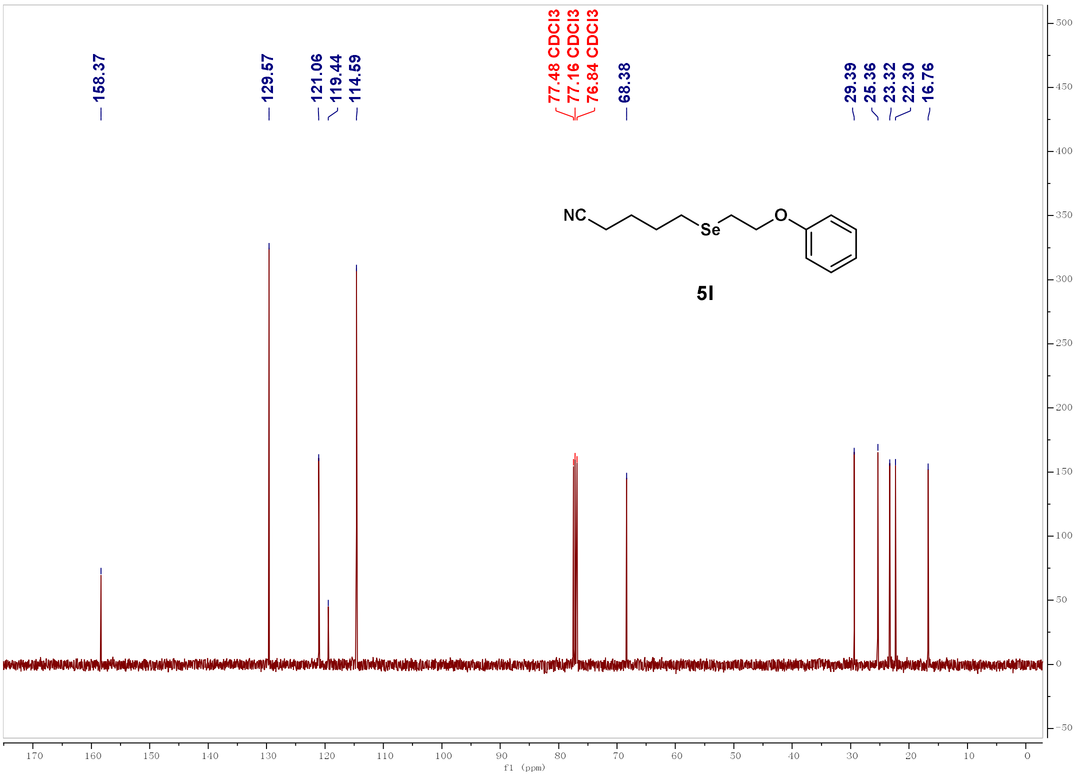

Supplementary Figure 87. ^{13}C NMR spectra for 5l

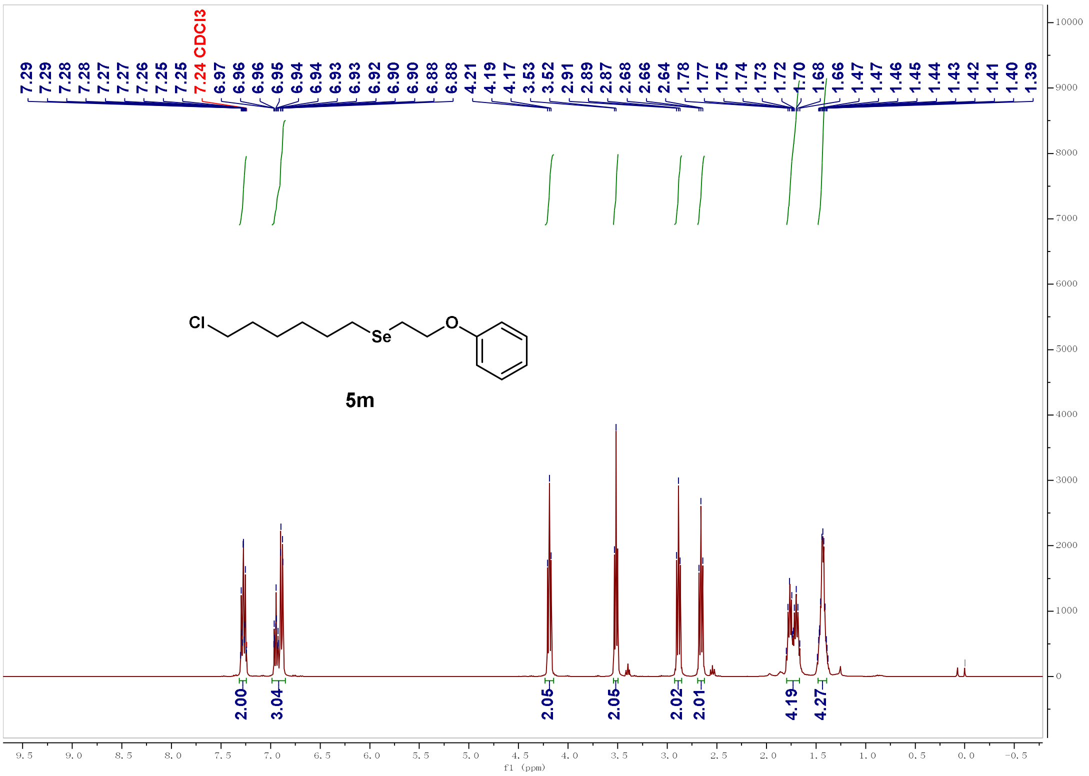

Supplementary Figure 88. ^{1}**H NMR spectra for 5m**

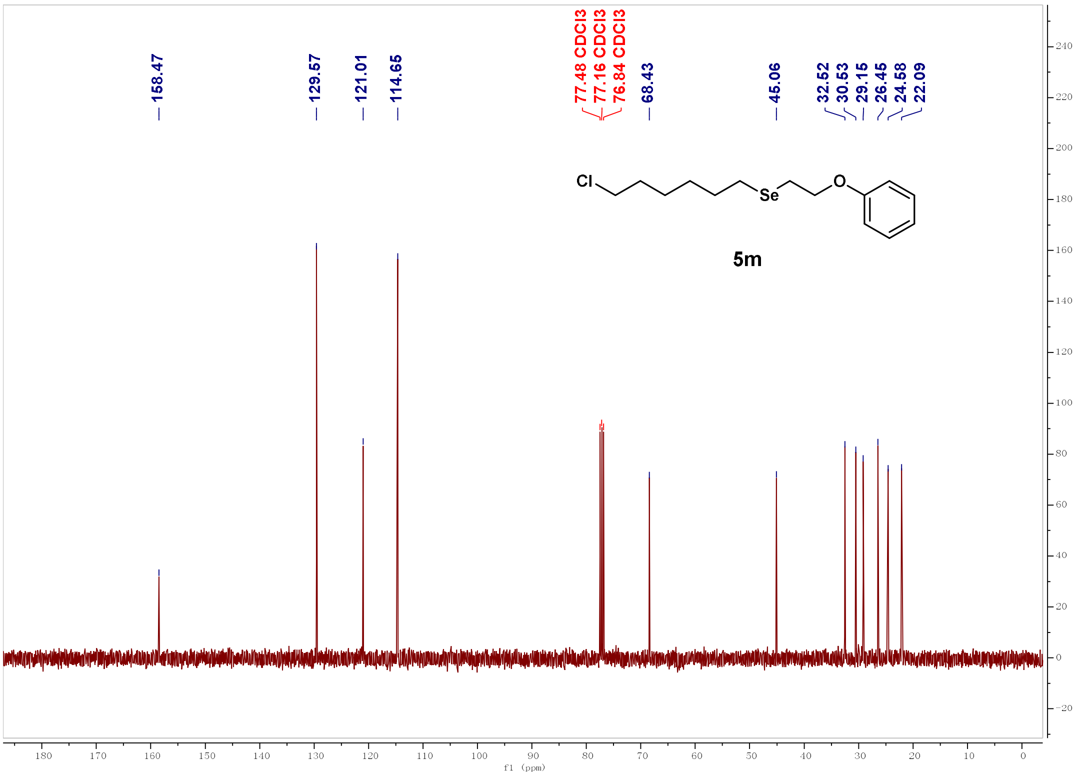

Supplementary Figure 89. ^{13}**C NMR spectra for 5m**

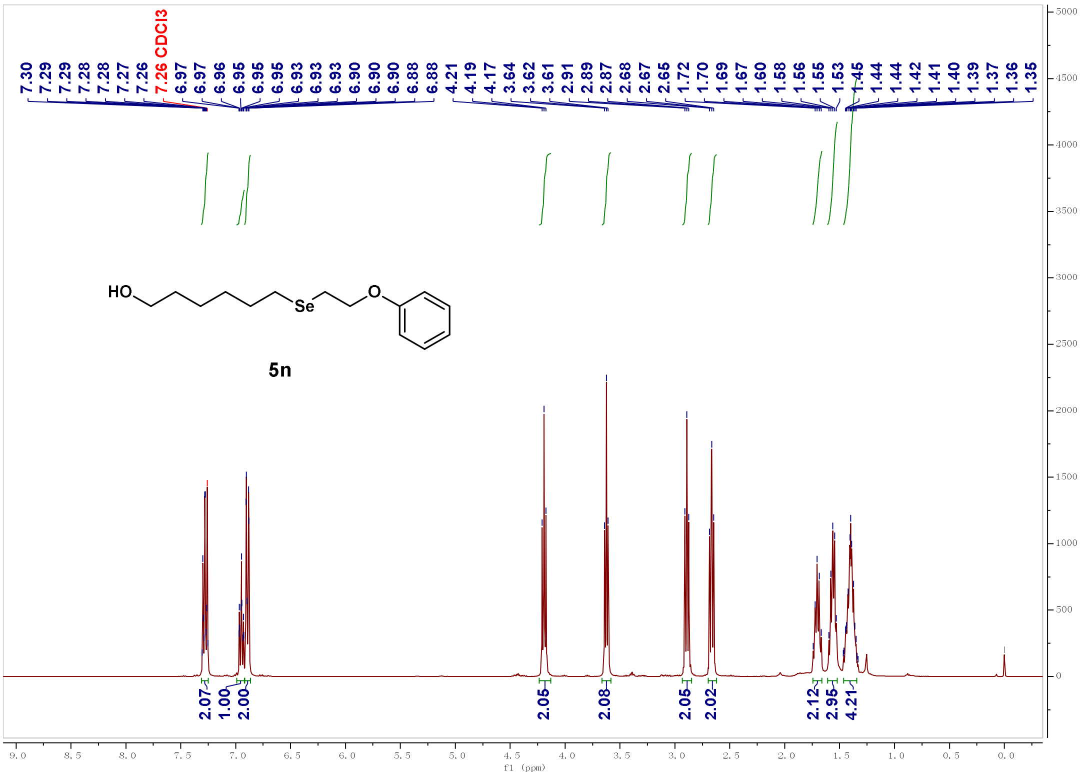

Supplementary Figure 90. ^{1}**H NMR spectra for 5n**

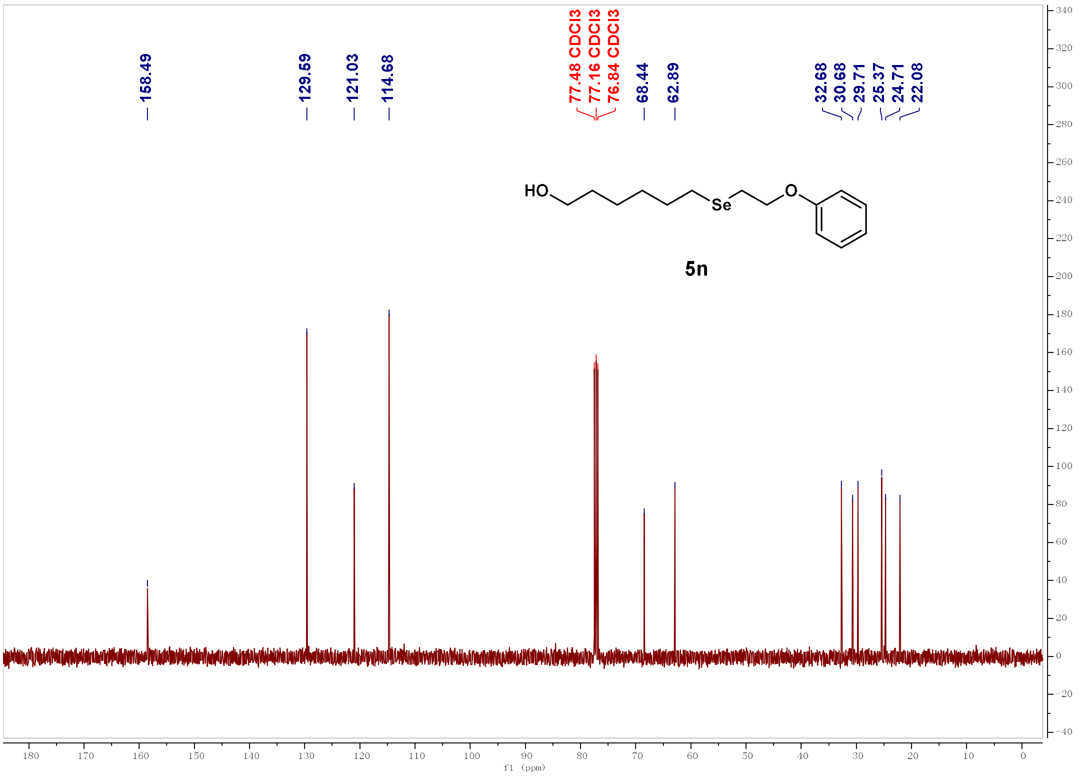

Supplementary Figure 91. ^{13}**C NMR spectra for 5n**

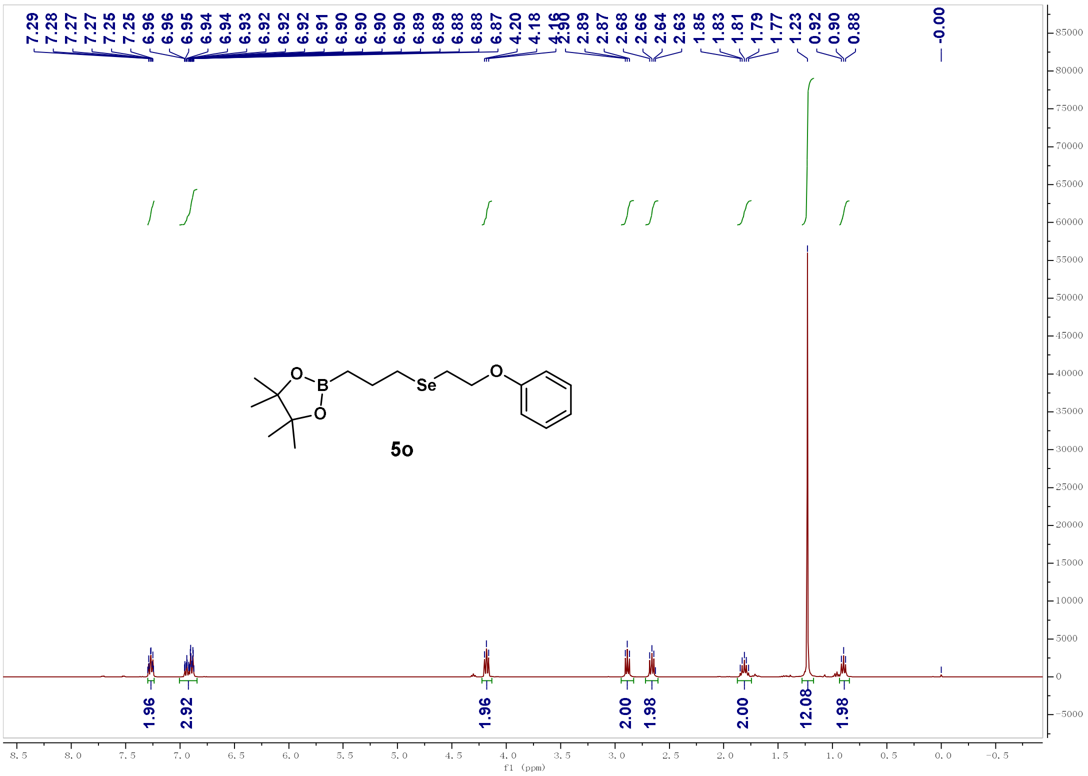

Supplementary Figure 92. ^{1}**H NMR spectra for 5o**

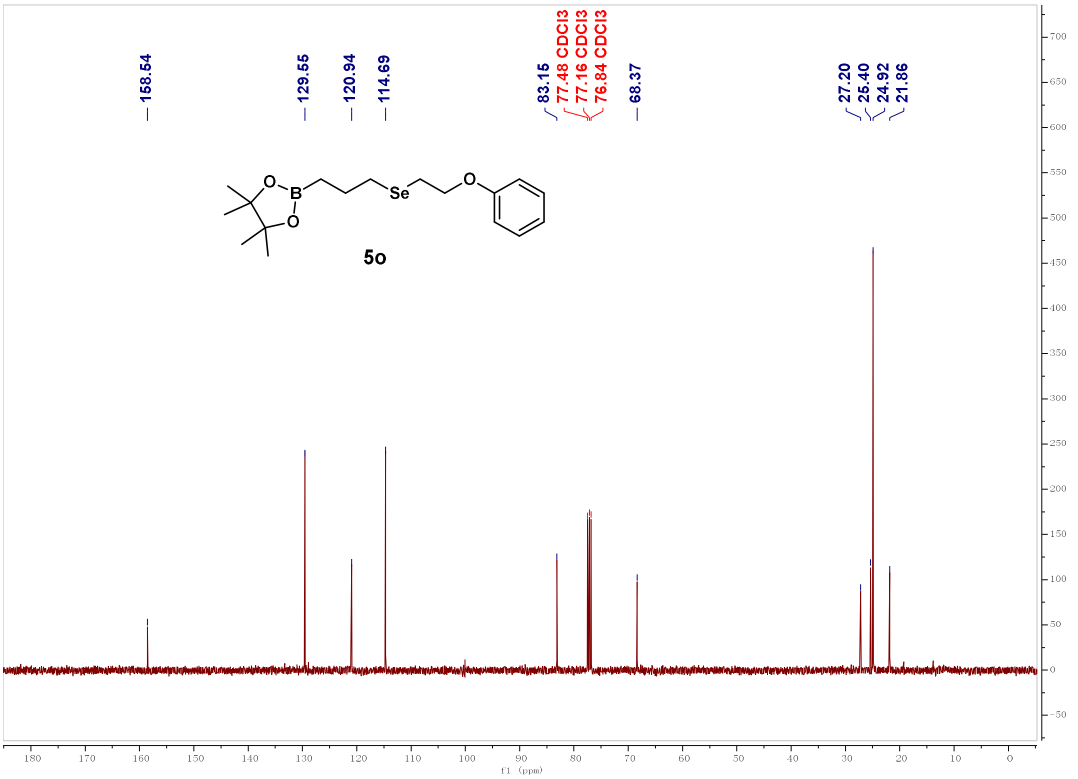

Supplementary Figure 93. ^{13}**C NMR spectra for 5o**

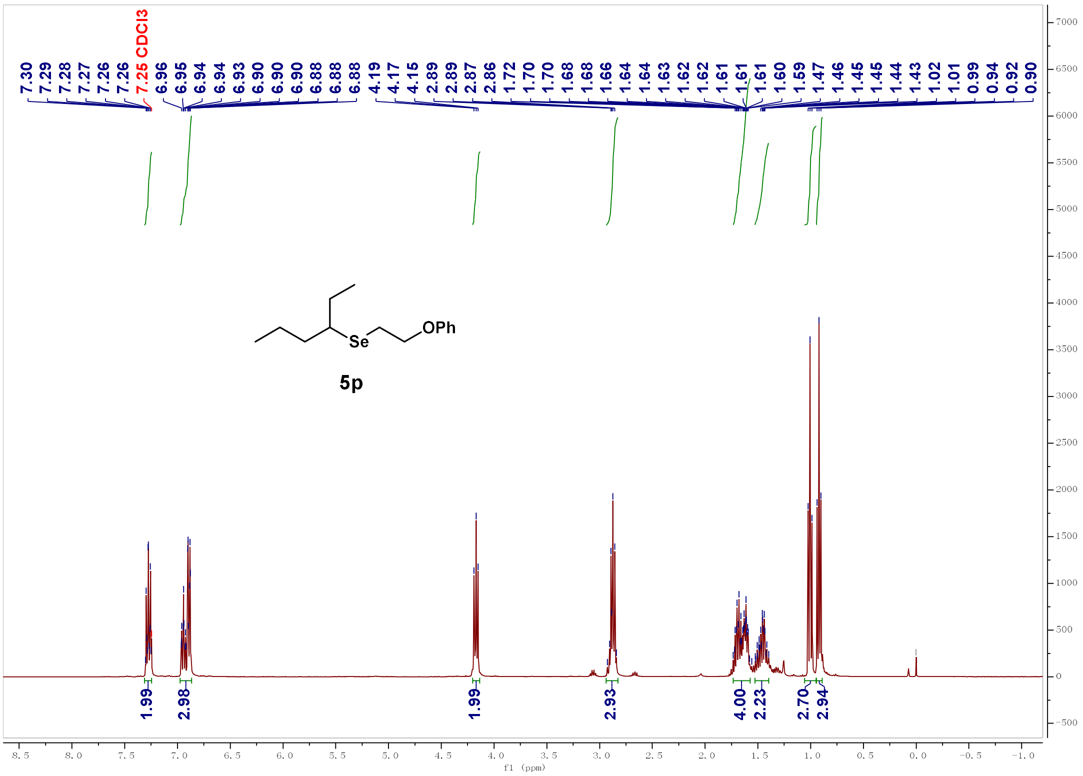

Supplementary Figure 94. ^{1}H NMR spectra for 5p

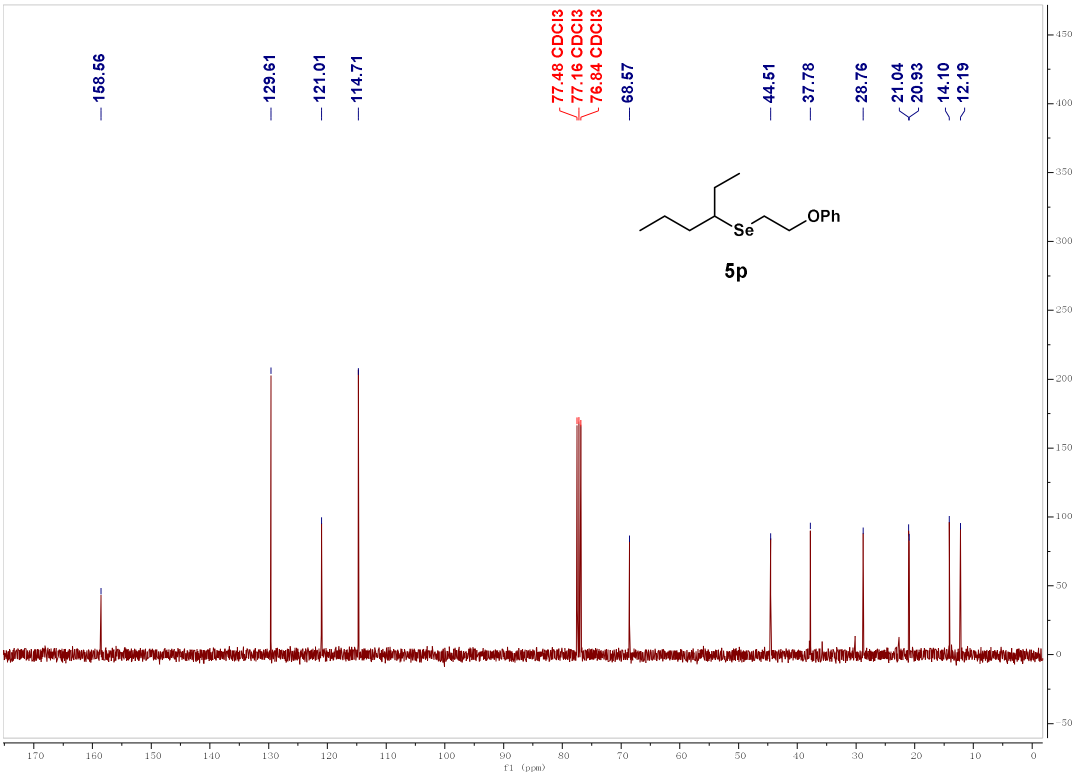

Supplementary Figure 95. ^{13}C NMR spectra for 5p

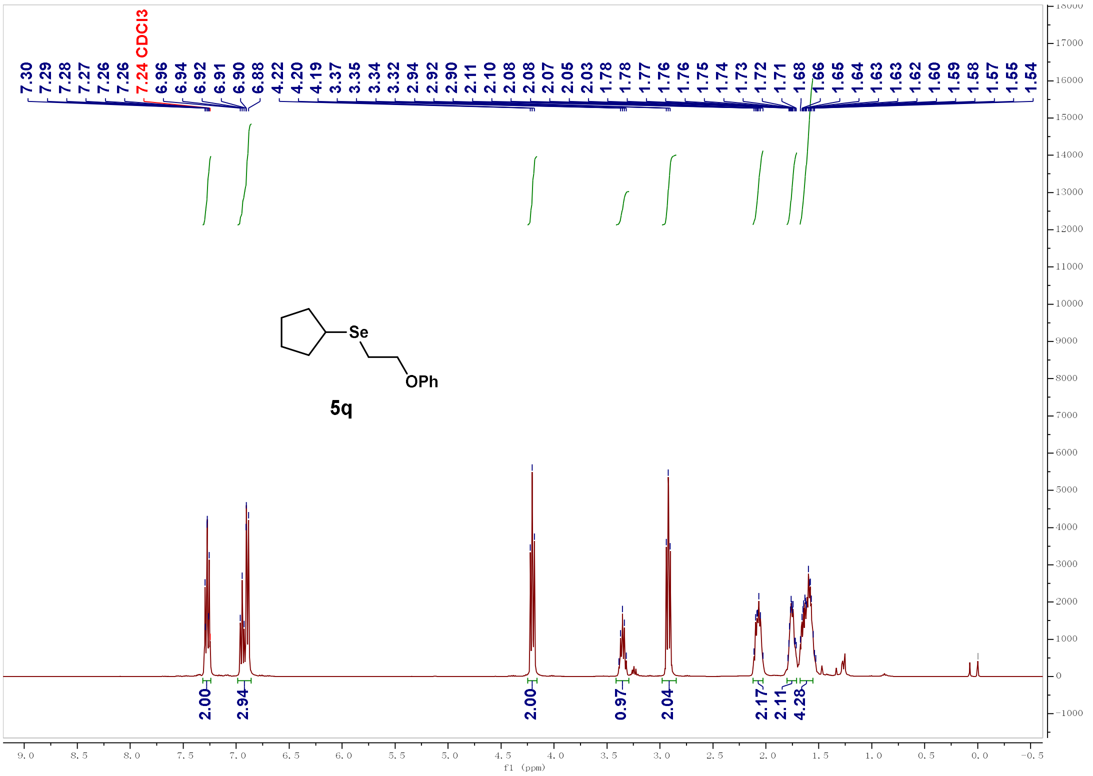

Supplementary Figure 96. ^{1}H NMR spectra for 5q

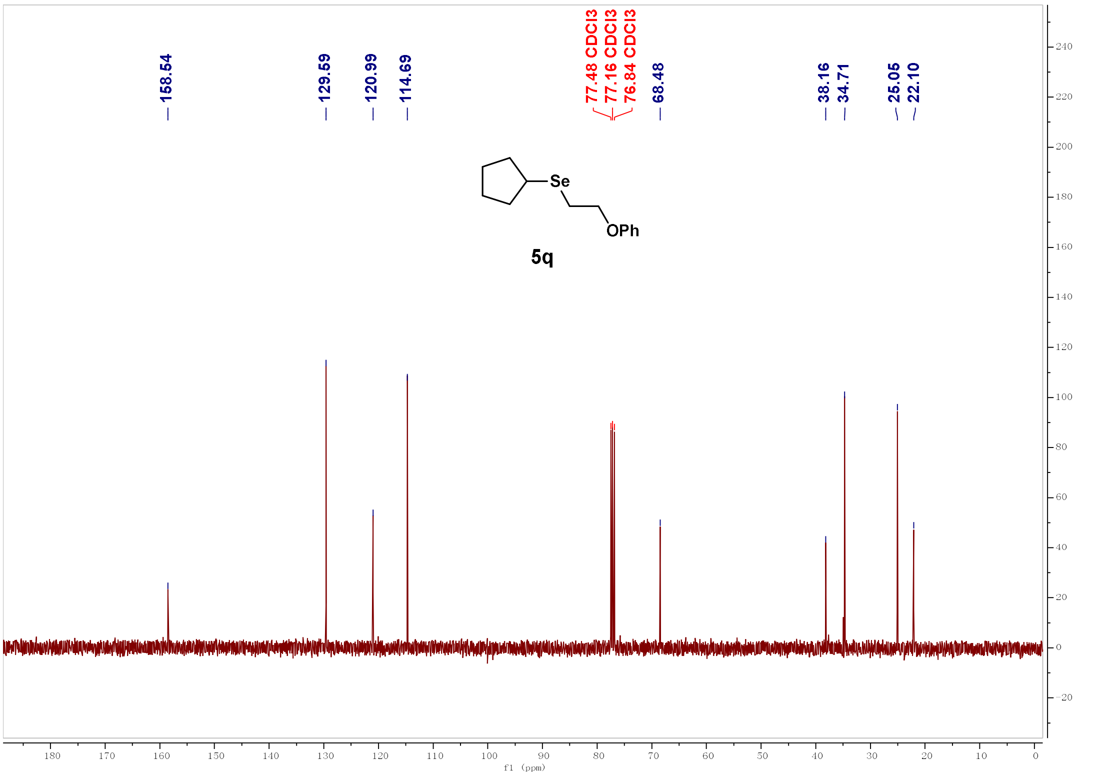

Supplementary Figure 97. ^{13}C NMR spectra for 5q

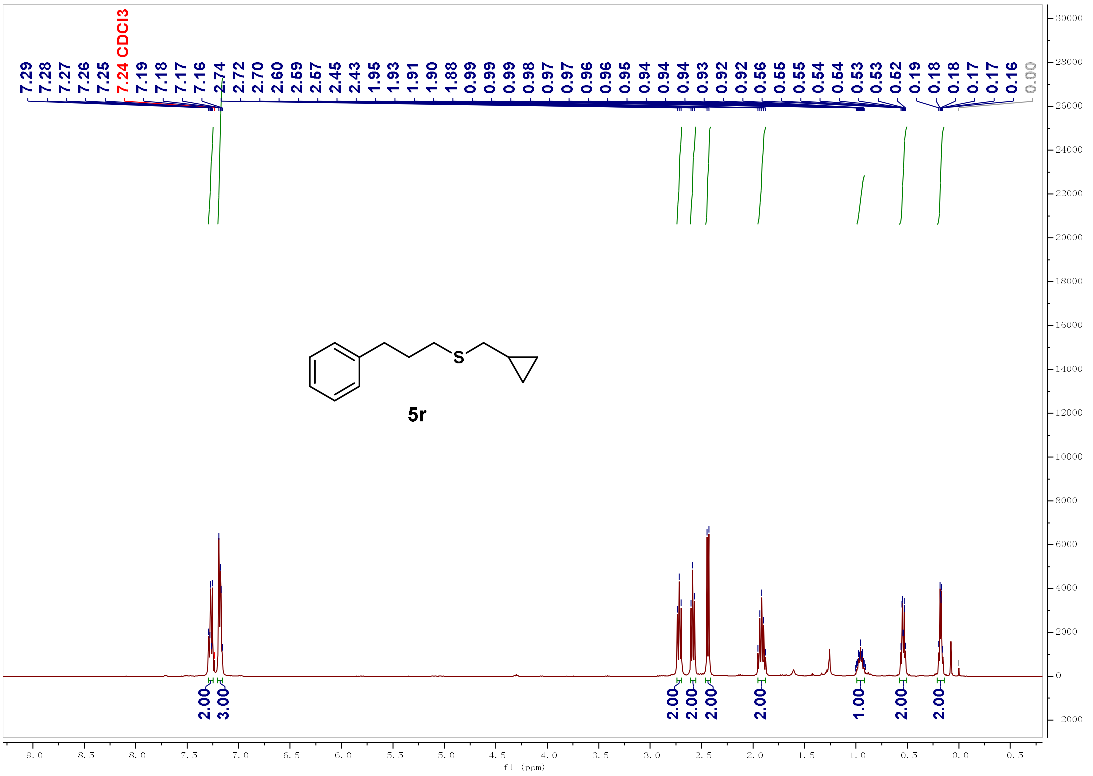

Supplementary Figure 98. ^{1}H NMR spectra for 5r

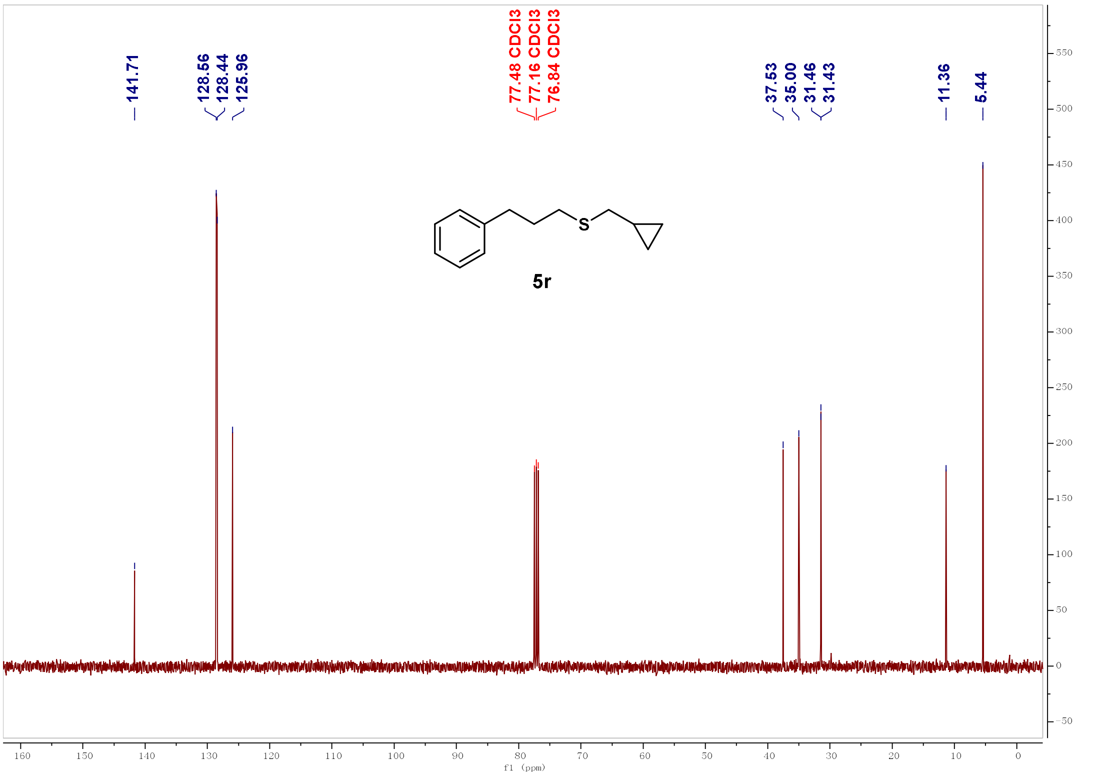

Supplementary Figure 99. ^{13}C NMR spectra for 5r

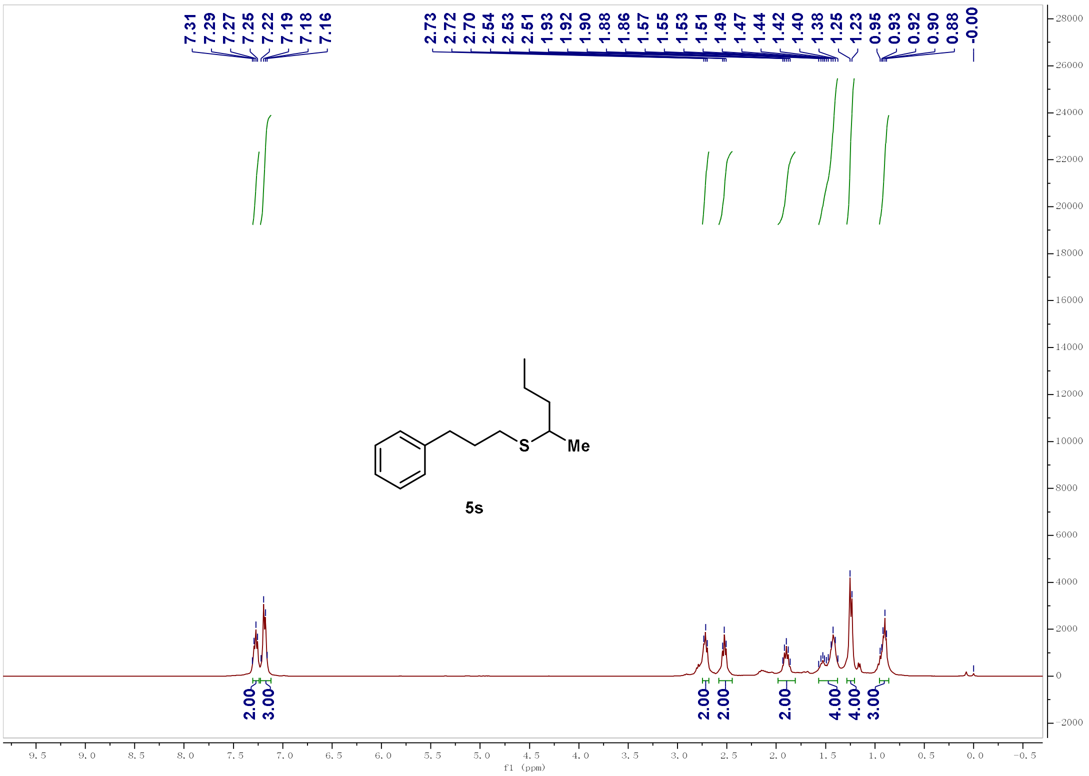

Supplementary Figure 100. ^{1}H NMR spectra for 5s

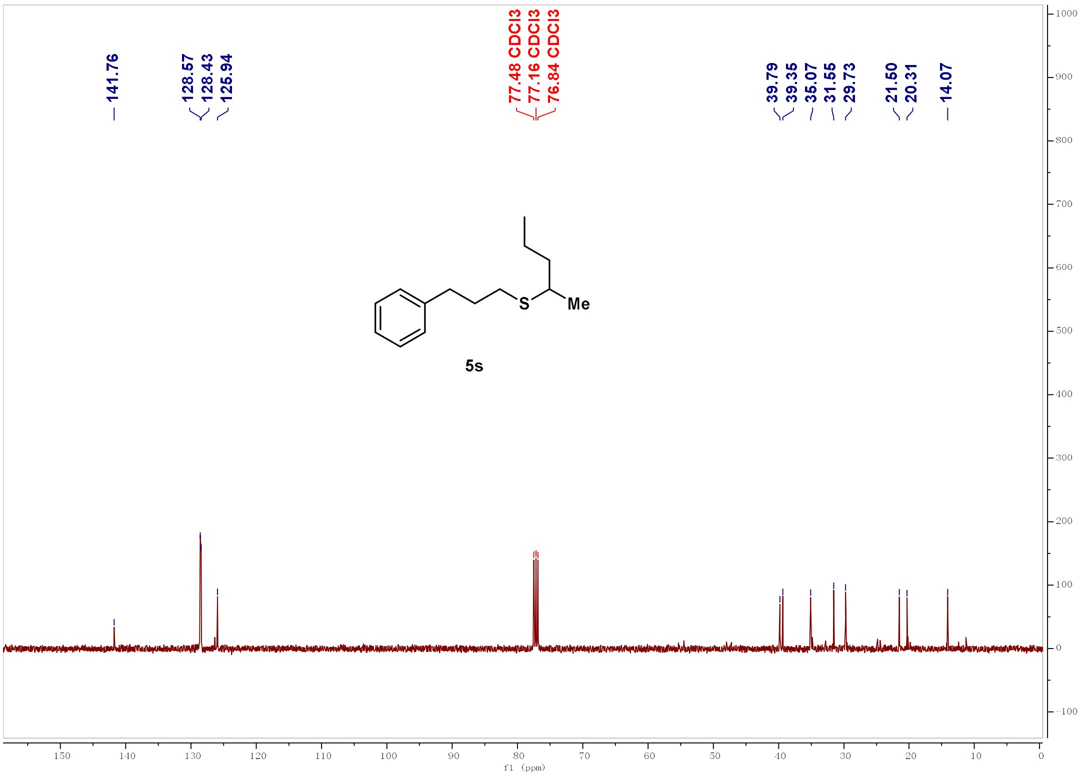

Supplementary Figure 101. ^{13}C NMR spectra for 5s

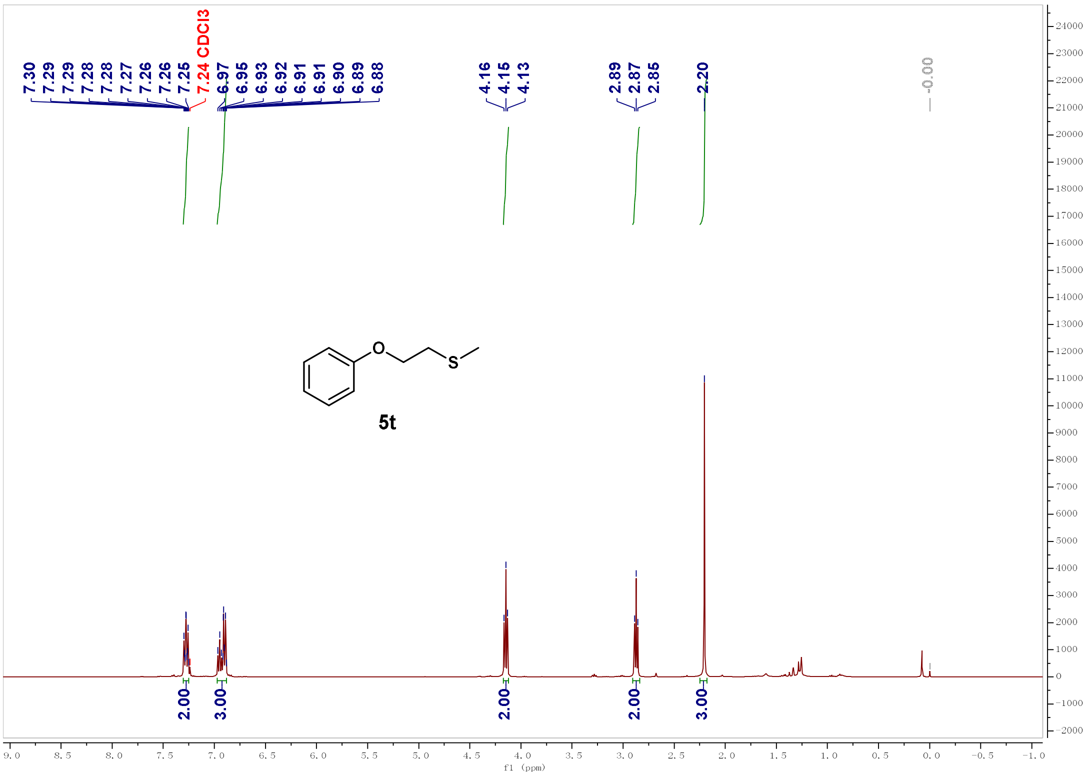

Supplementary Figure 102. ^{1}H NMR spectra for 5t

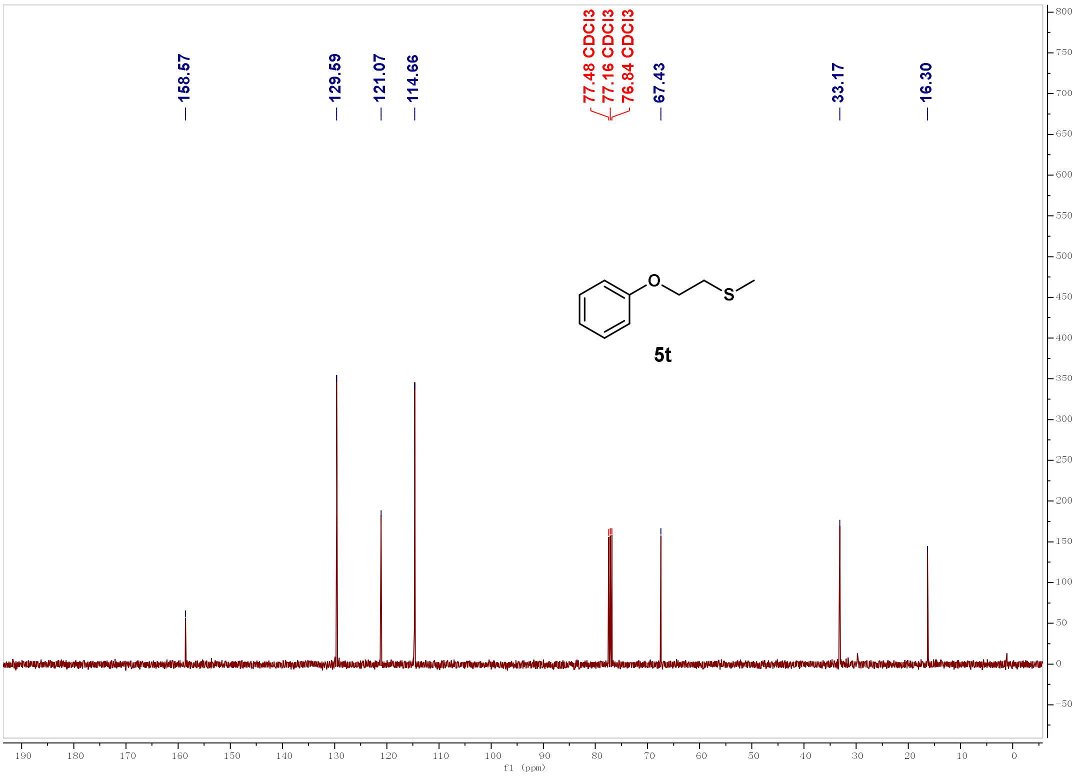

Supplementary Figure 103. ^{13}C NMR spectra for 5t

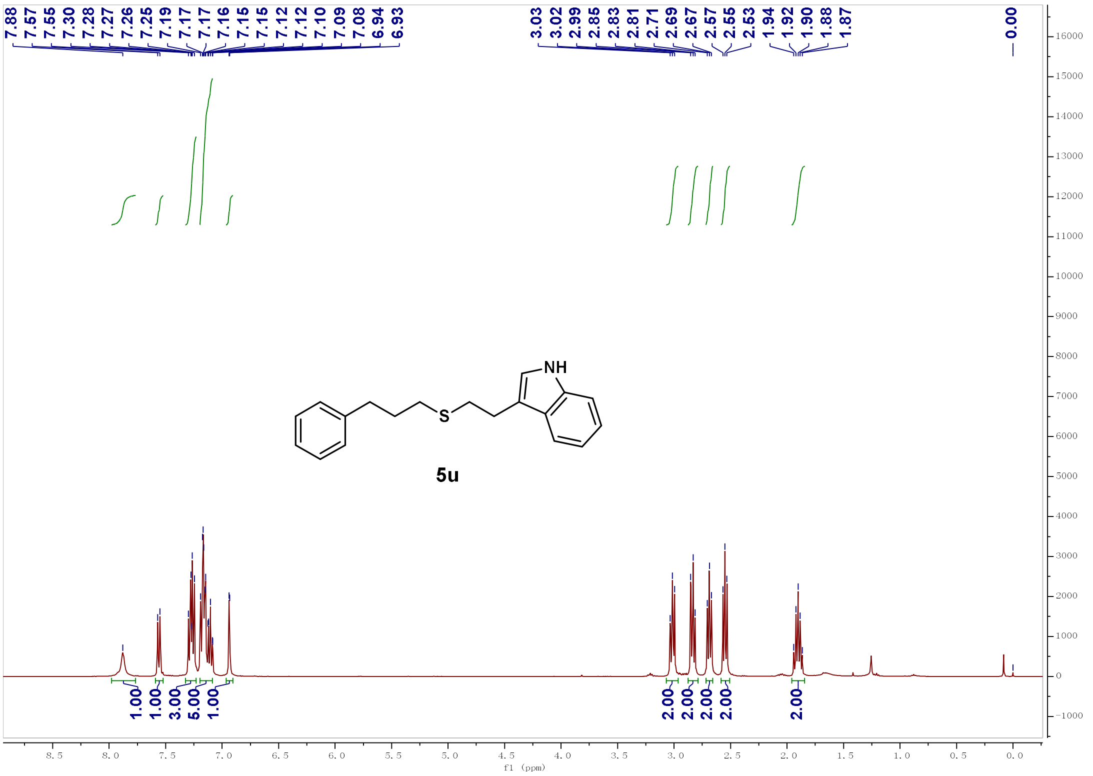

Supplementary Figure 104. ^{1}H NMR spectra for 5u

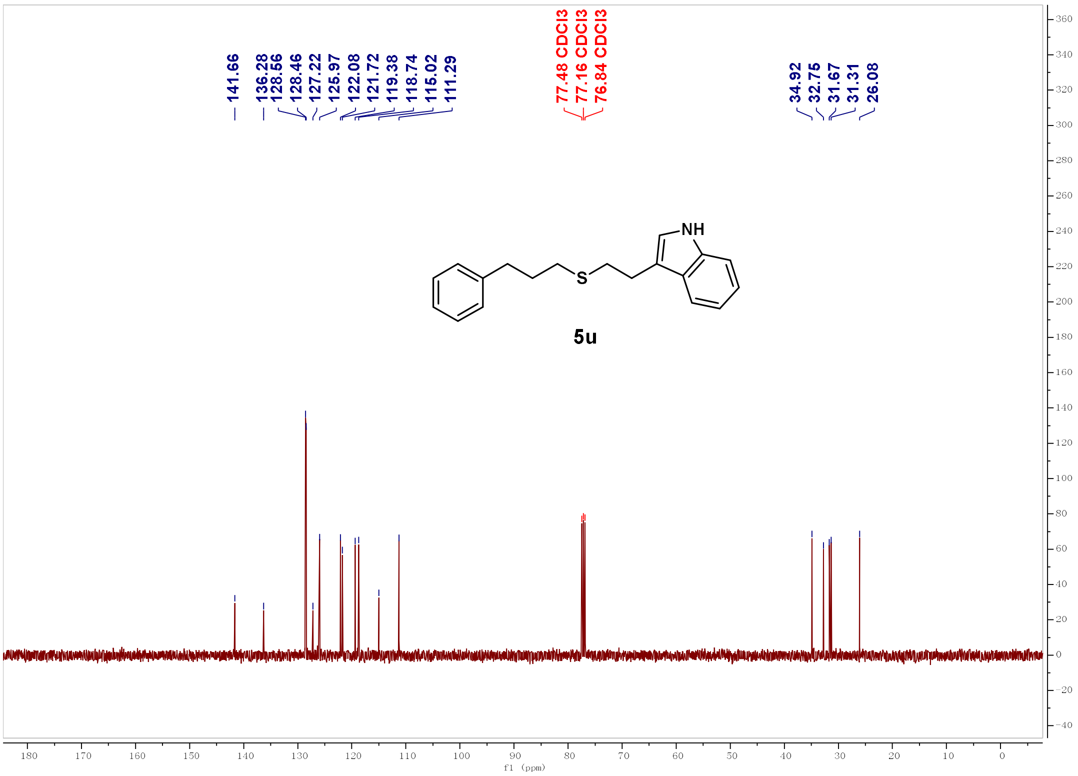

Supplementary Figure 105. ^{13}C NMR spectra for 5u

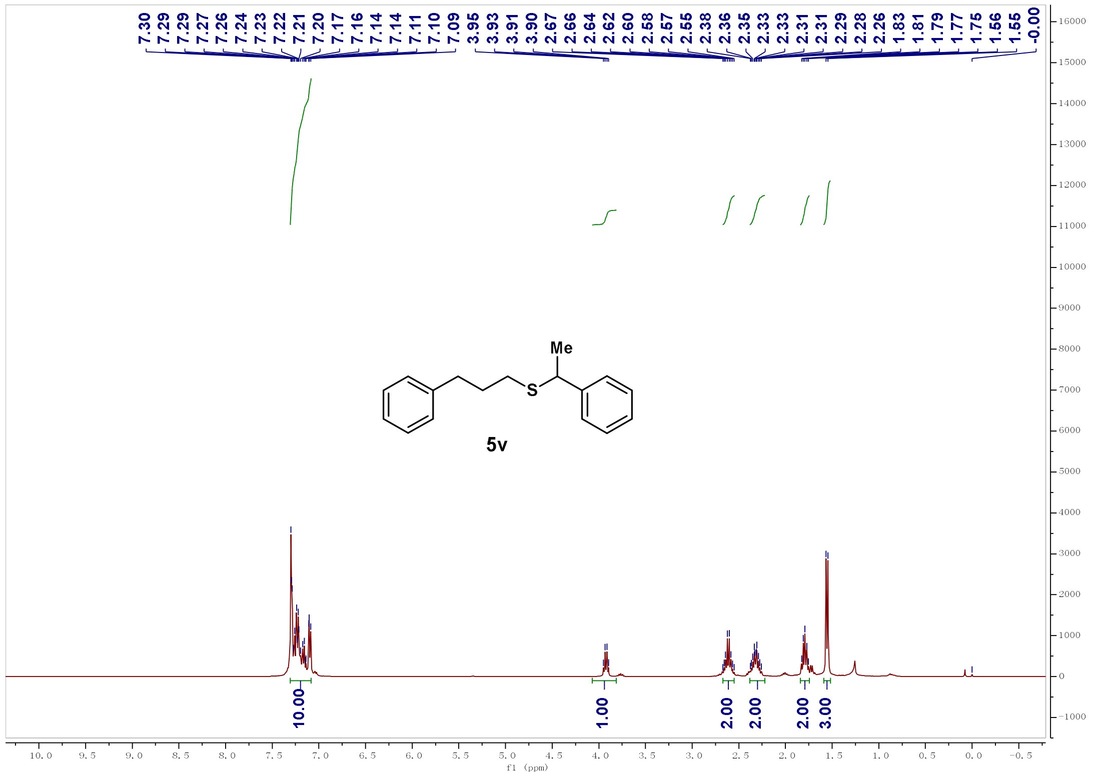

Supplementary Figure 106. ^{1}H NMR spectra for 5v

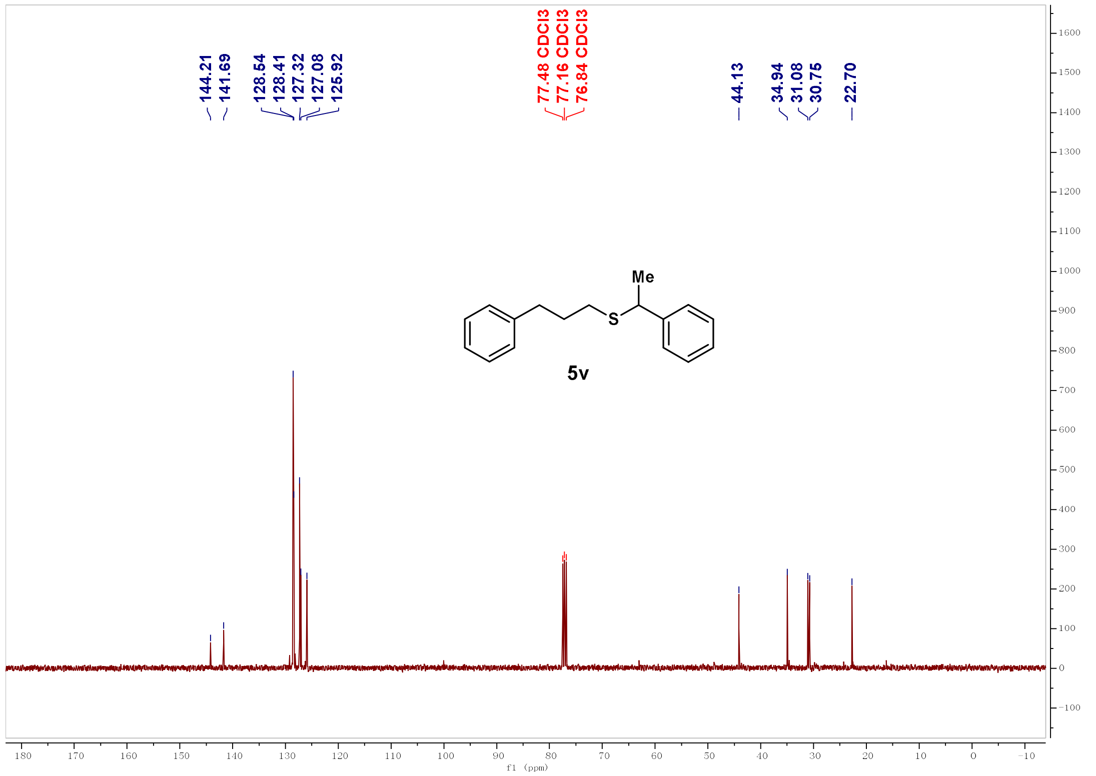

Supplementary Figure 107. ^{13}C NMR spectra for 5v

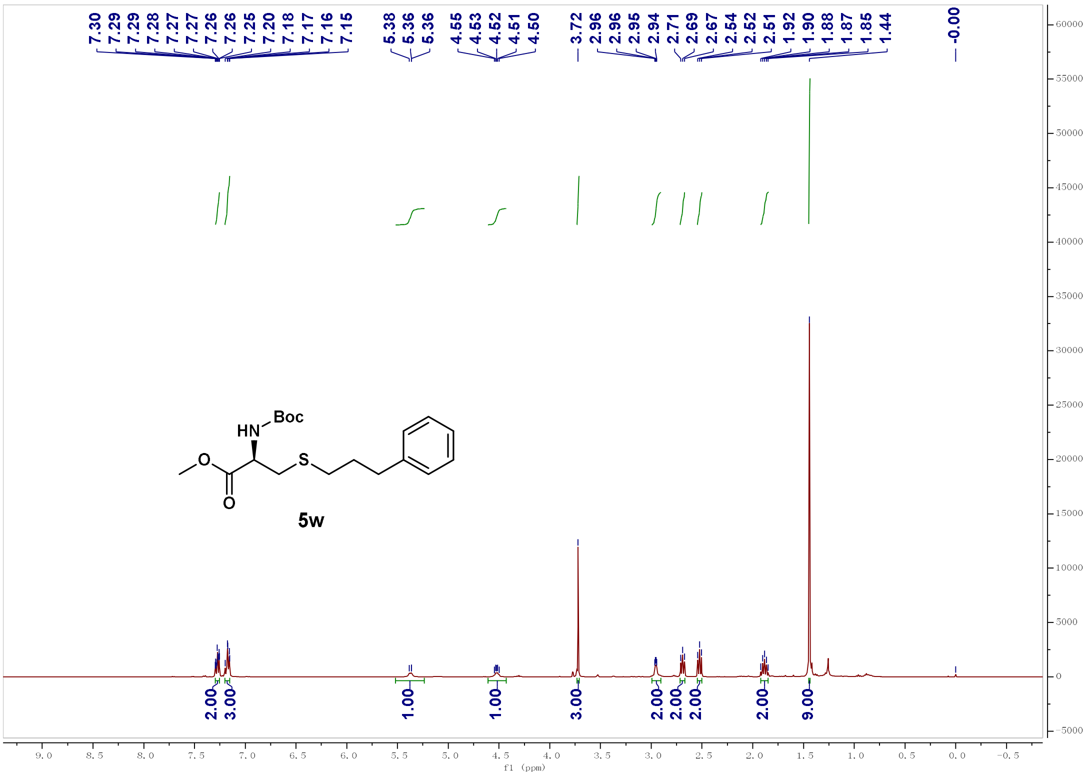

Supplementary Figure 108. ^{1}**H NMR spectra for 5w**

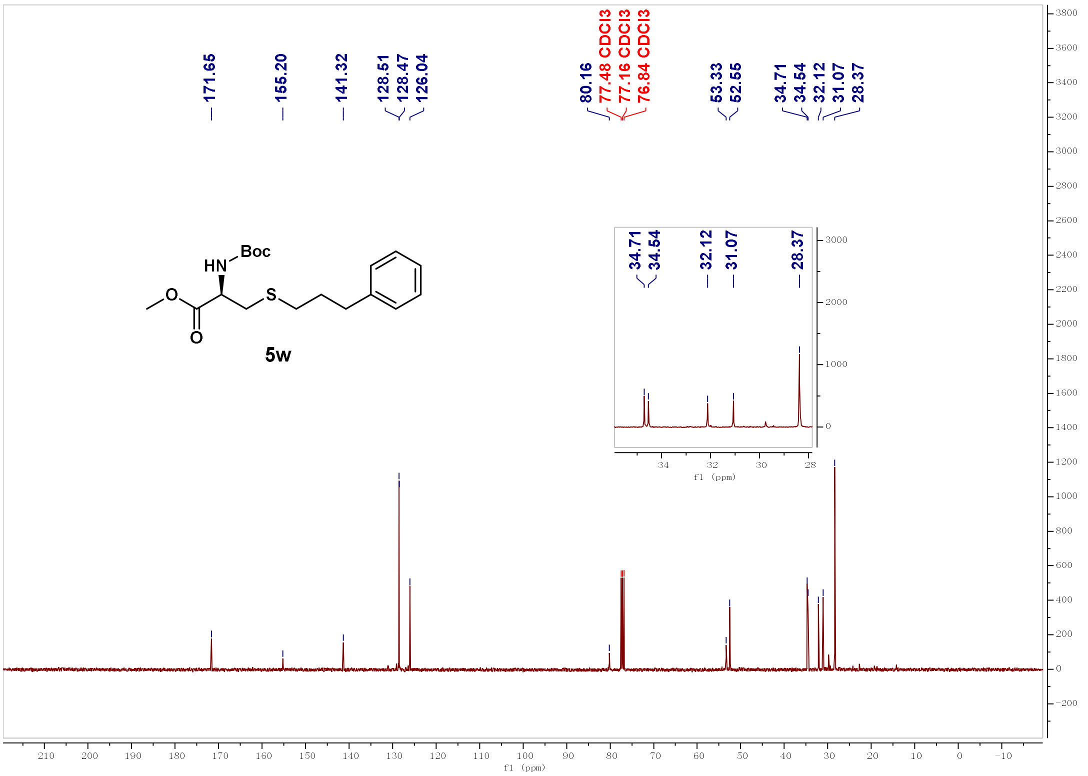

Supplementary Figure 109. ^{13}**C NMR spectra for 5w**

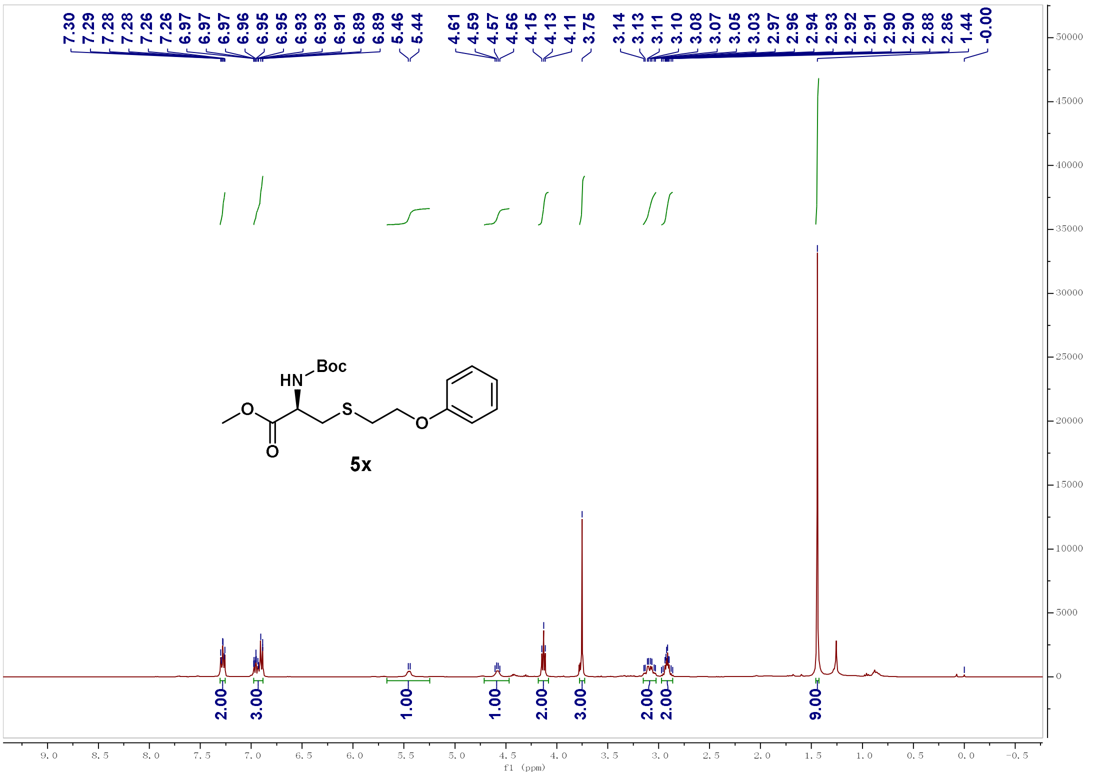

Supplementary Figure 110. ^{1}H NMR spectra for 5x

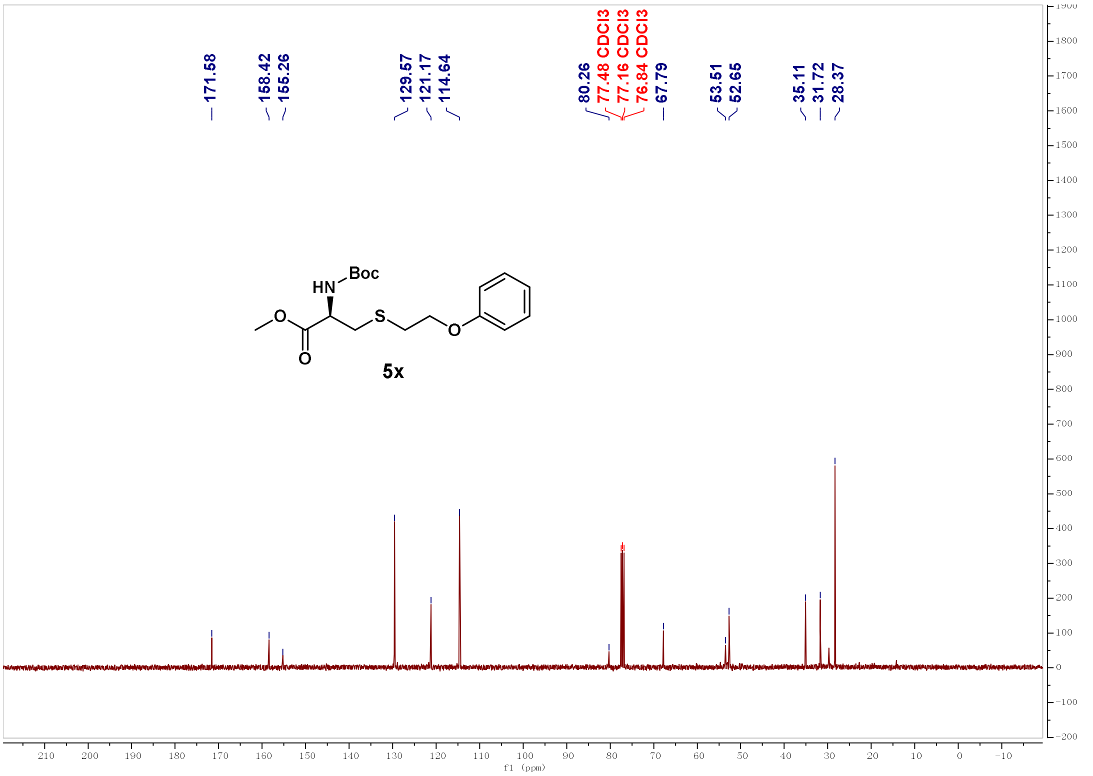

Supplementary Figure 111. ^{13}C NMR spectra for 5x

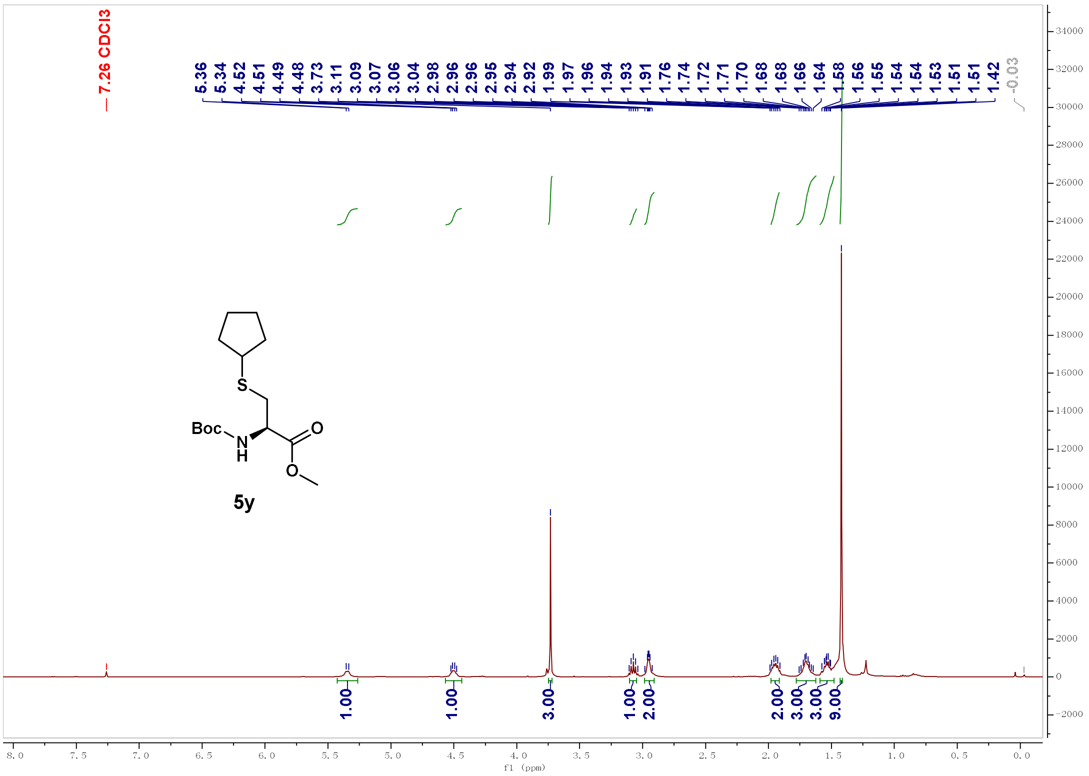

Supplementary Figure 112. ^{1}**H NMR spectra for 5y**

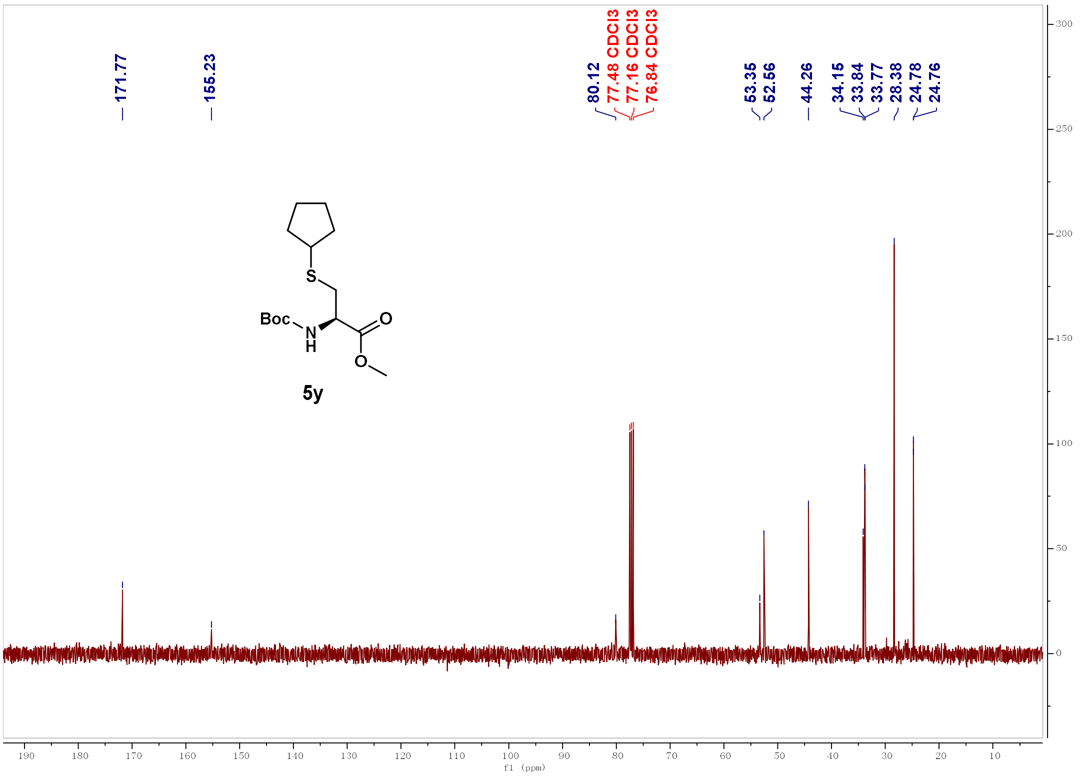

Supplementary Figure 113. ^{13}**C NMR spectra for 5y**

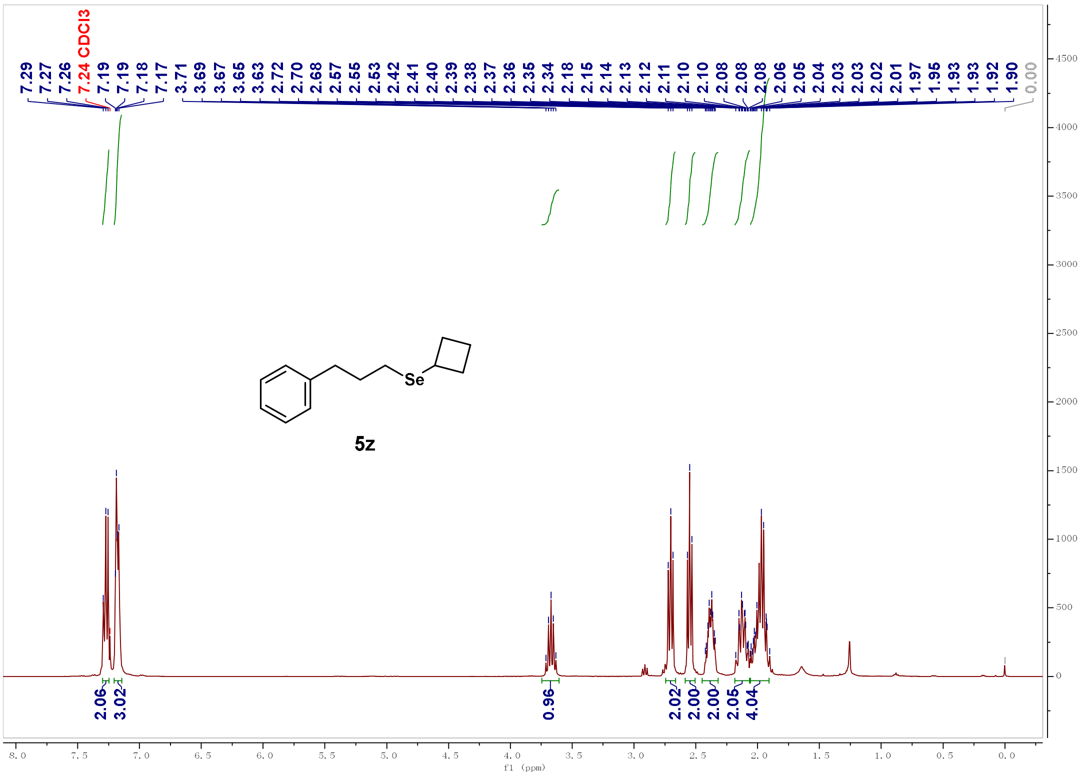

Supplementary Figure 114. ^{1}H NMR spectra for 5z

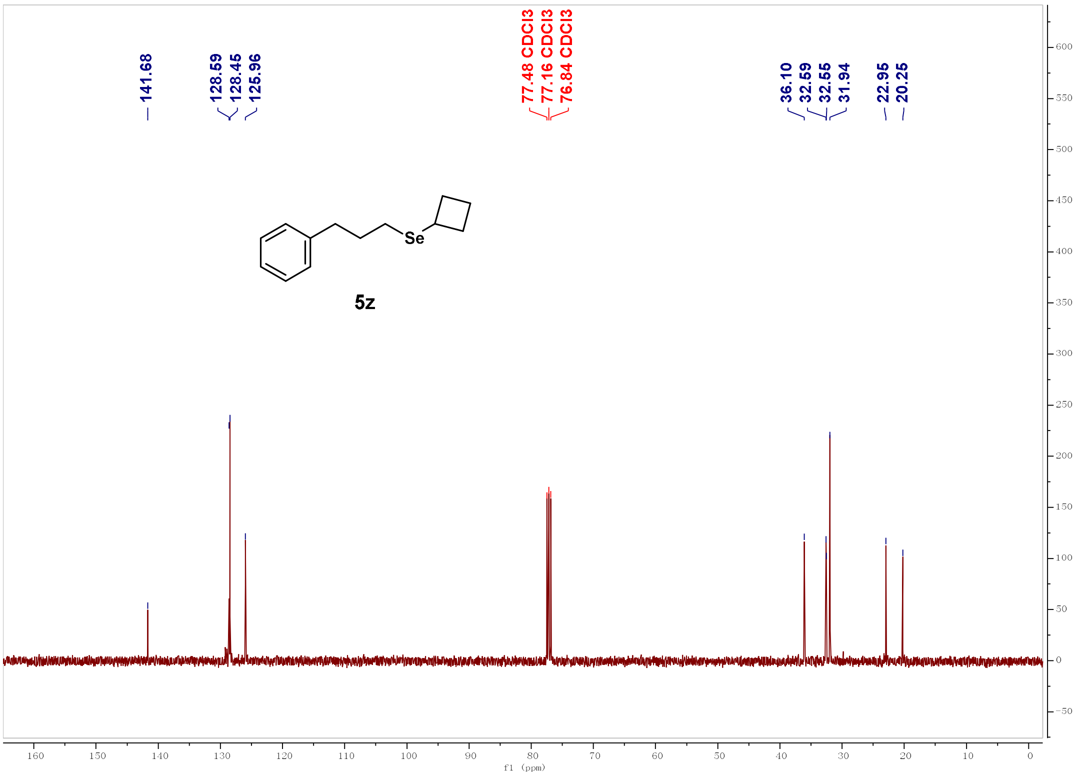

Supplementary Figure 115. ^{13}C NMR spectra for 5z

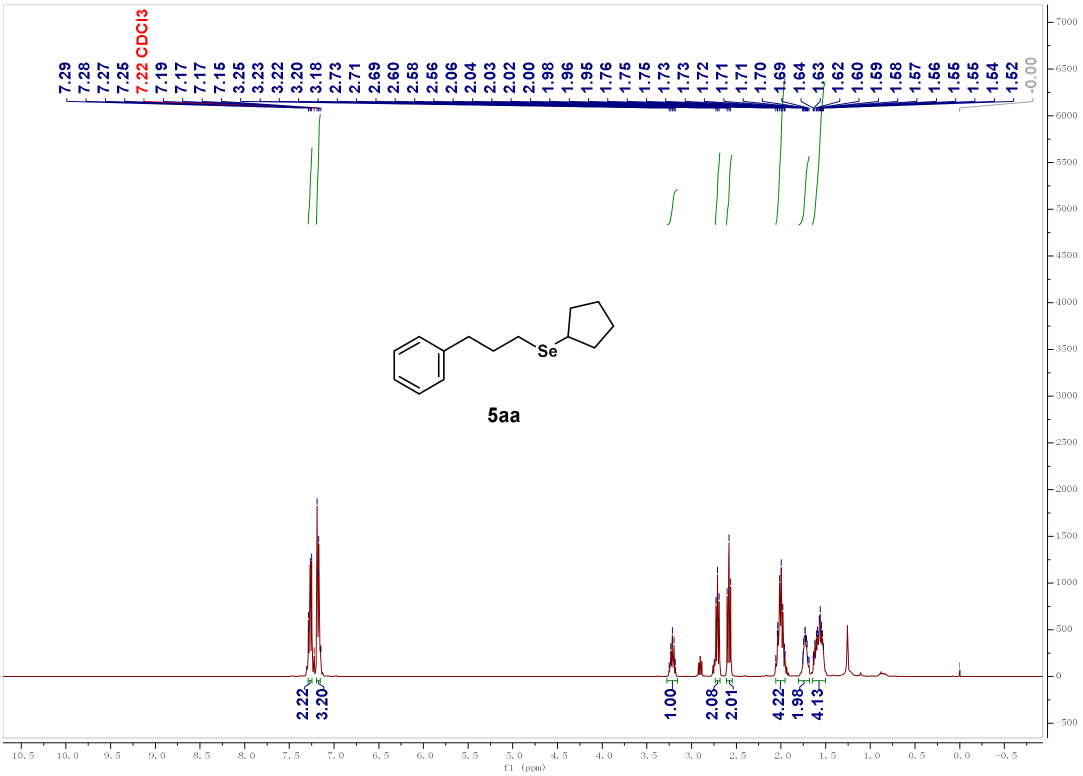

Supplementary Figure 116. ^{1}**H NMR spectra for 5aa**

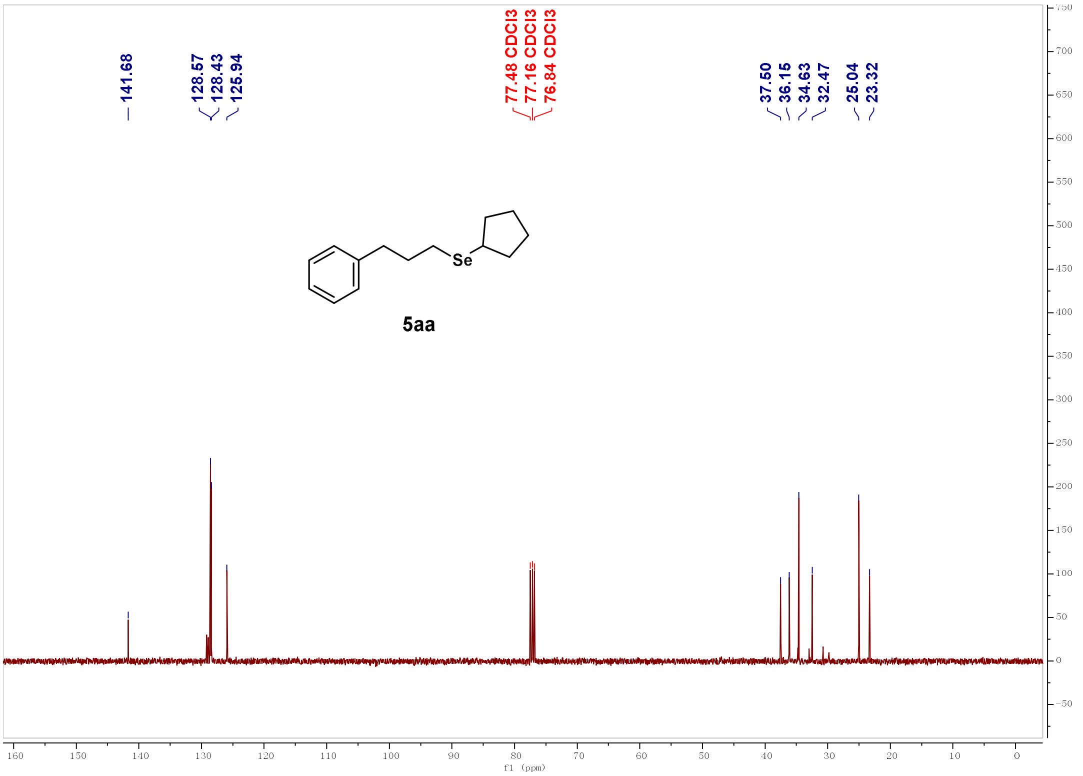

Supplementary Figure 117. ^{13}**C NMR spectra for 5aa**

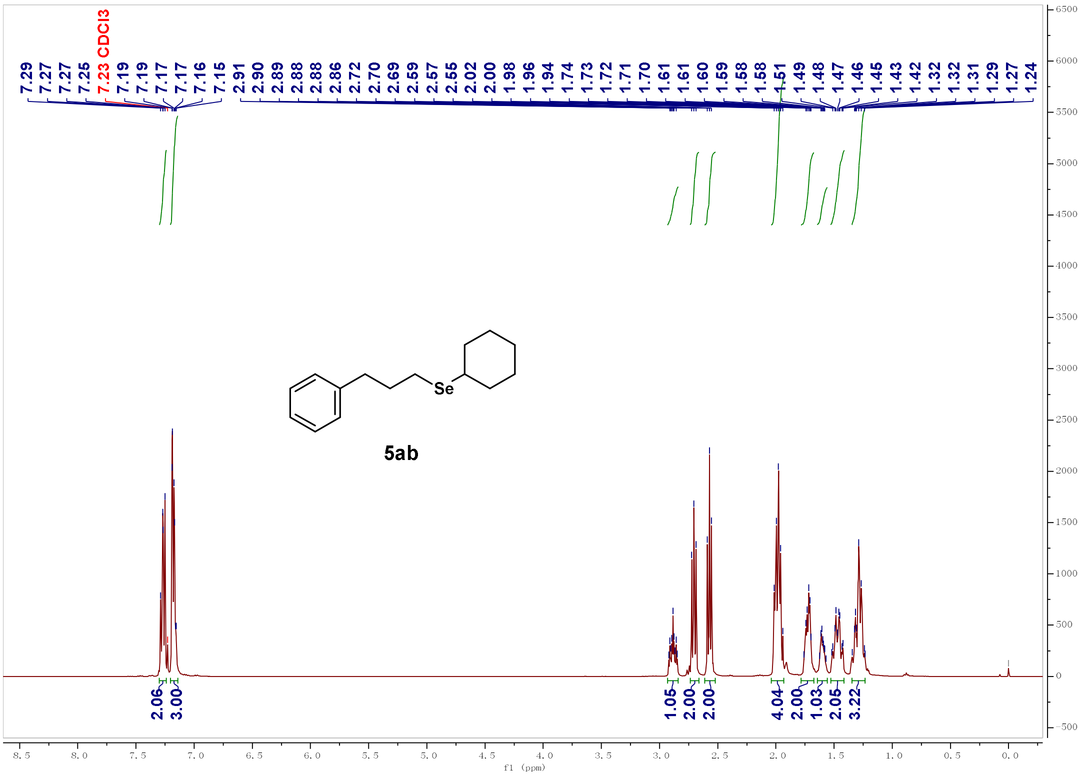

Supplementary Figure 118. ^{1}**H NMR spectra for 5ab**

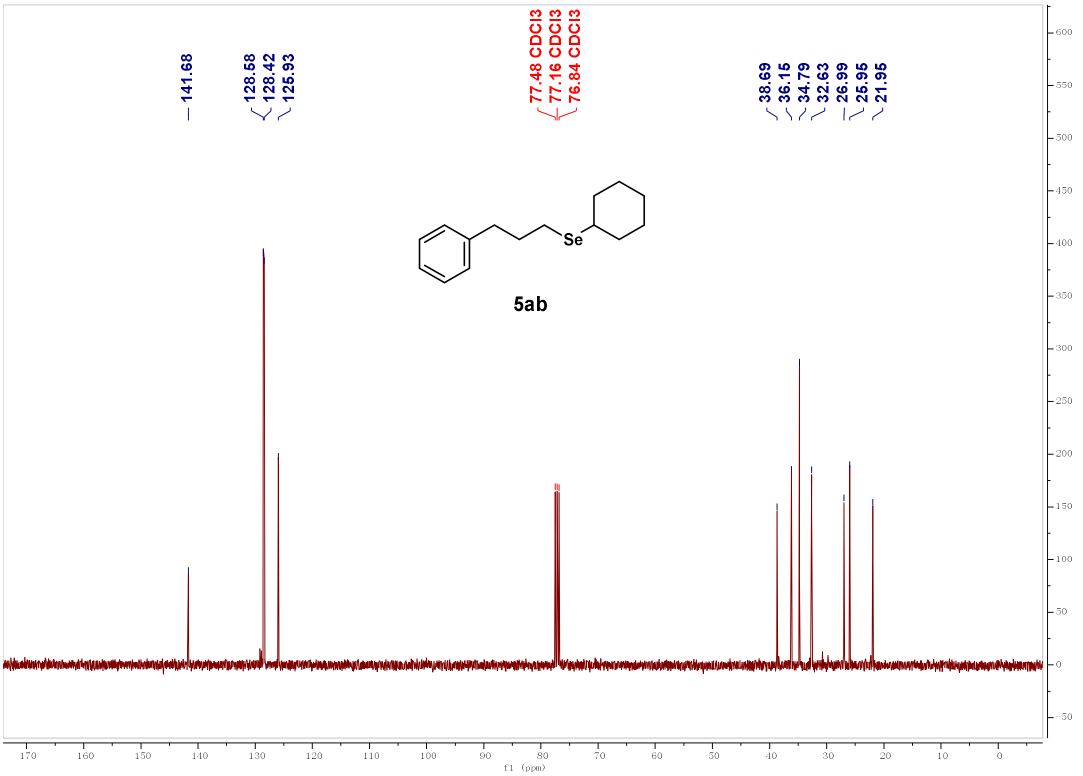

Supplementary Figure 119. ^{13}**C NMR spectra for 5ab**

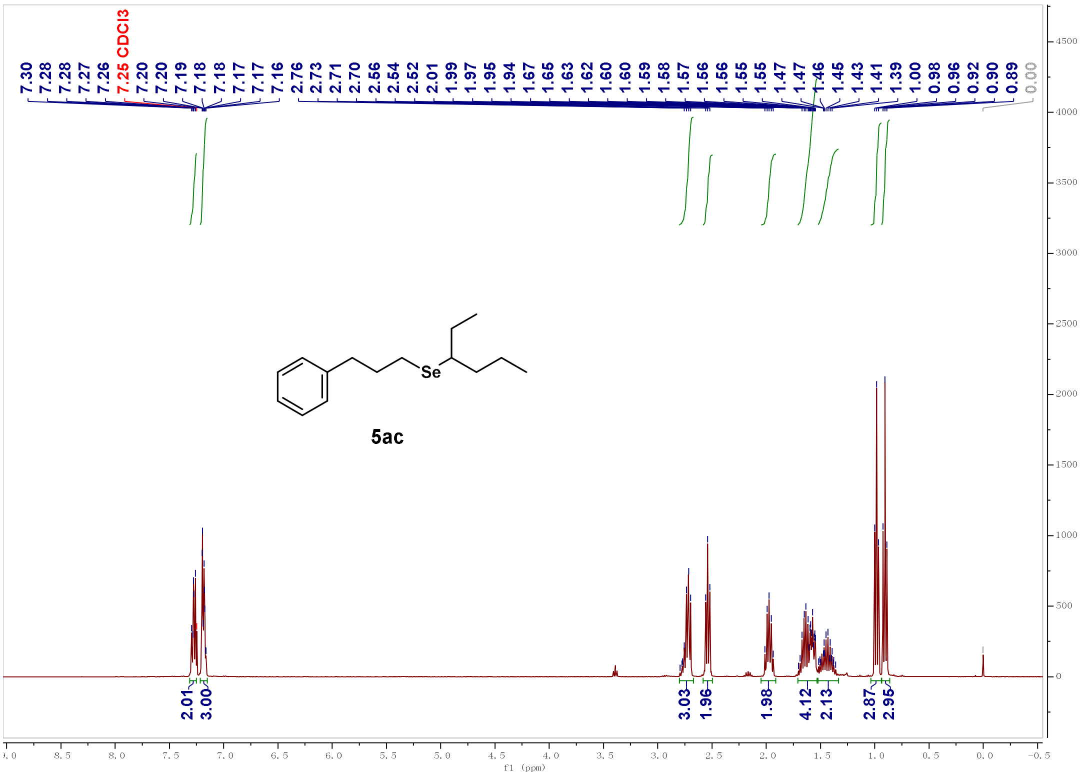

Supplementary Figure 120. ^{1}H NMR spectra for 5ac

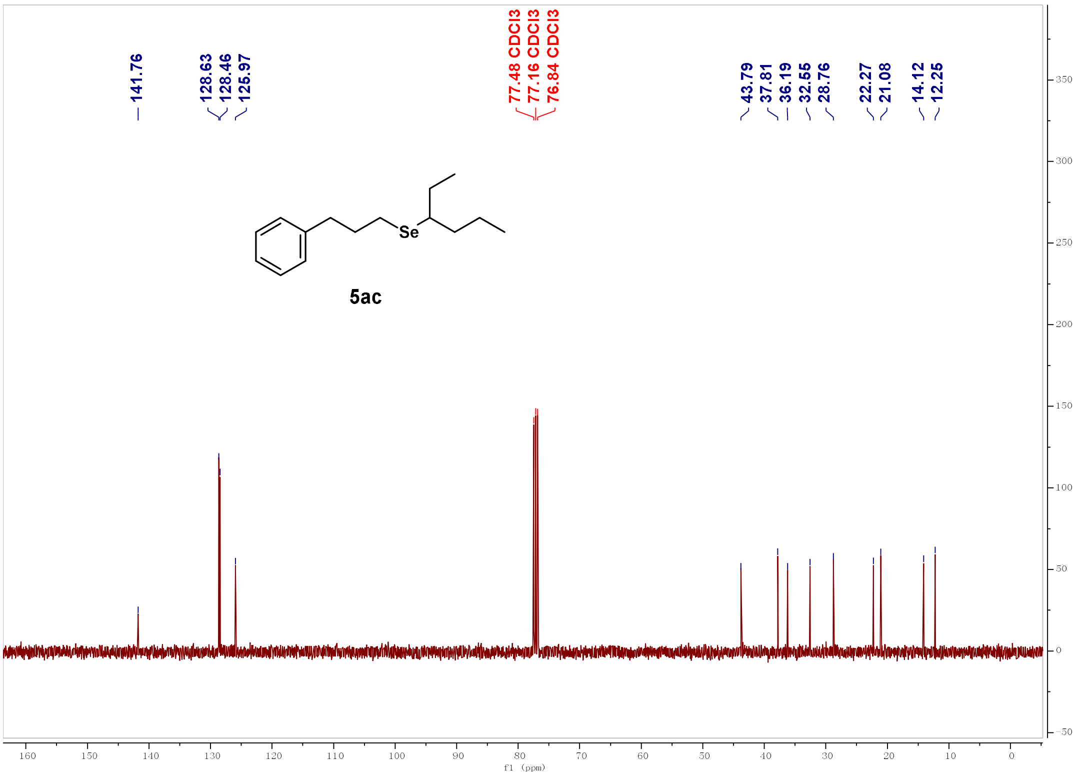

Supplementary Figure 121. ^{13}C NMR spectra for 5ac

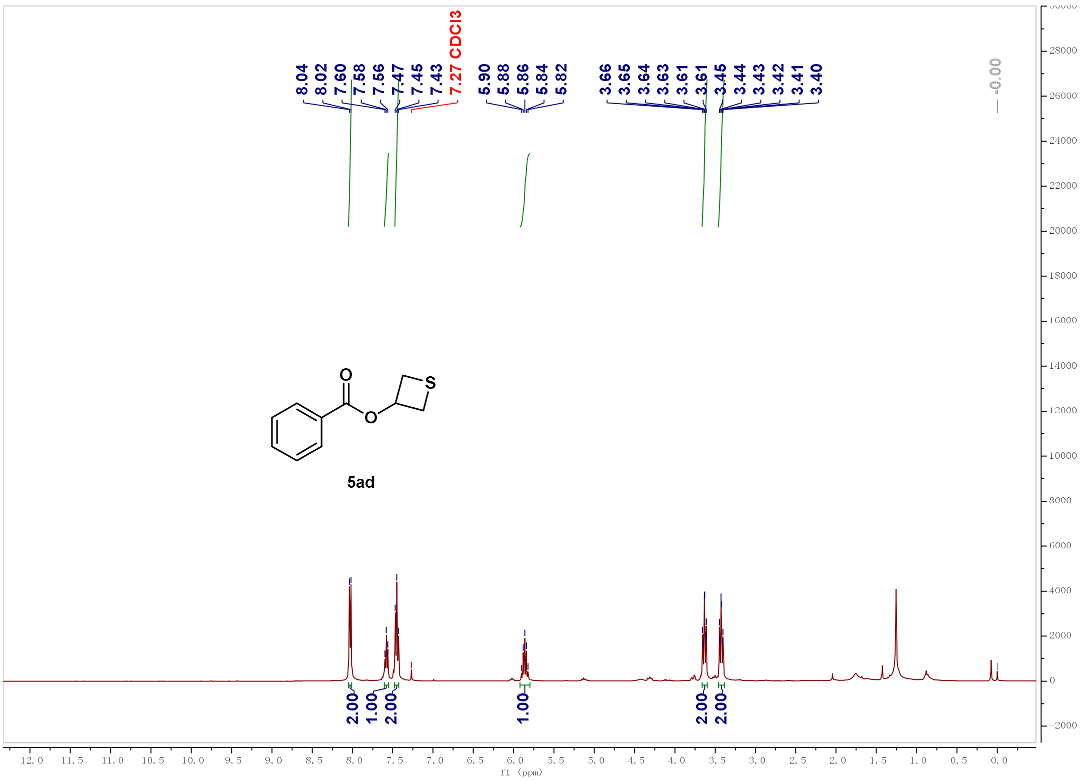

Supplementary Figure 122. **^{1}H NMR spectra for 5ad**

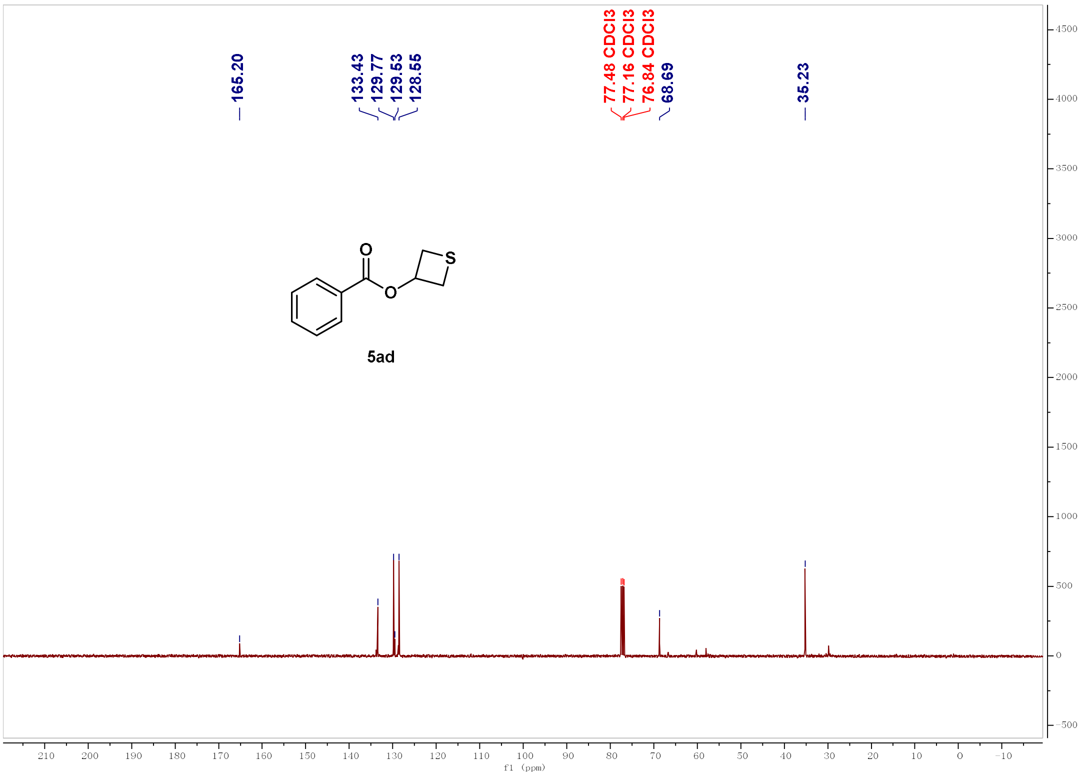

Supplementary Figure 123. **^{13}C NMR spectra for 5ad**

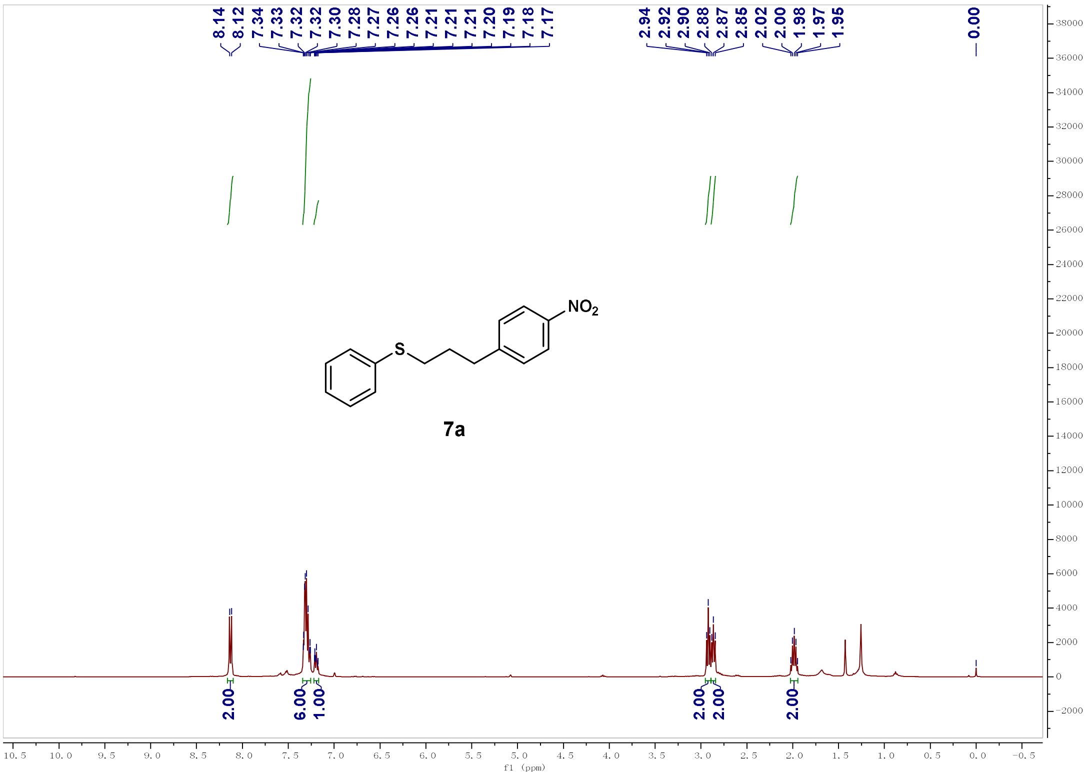

Supplementary Figure 124. ^{1}H NMR spectra for 7a

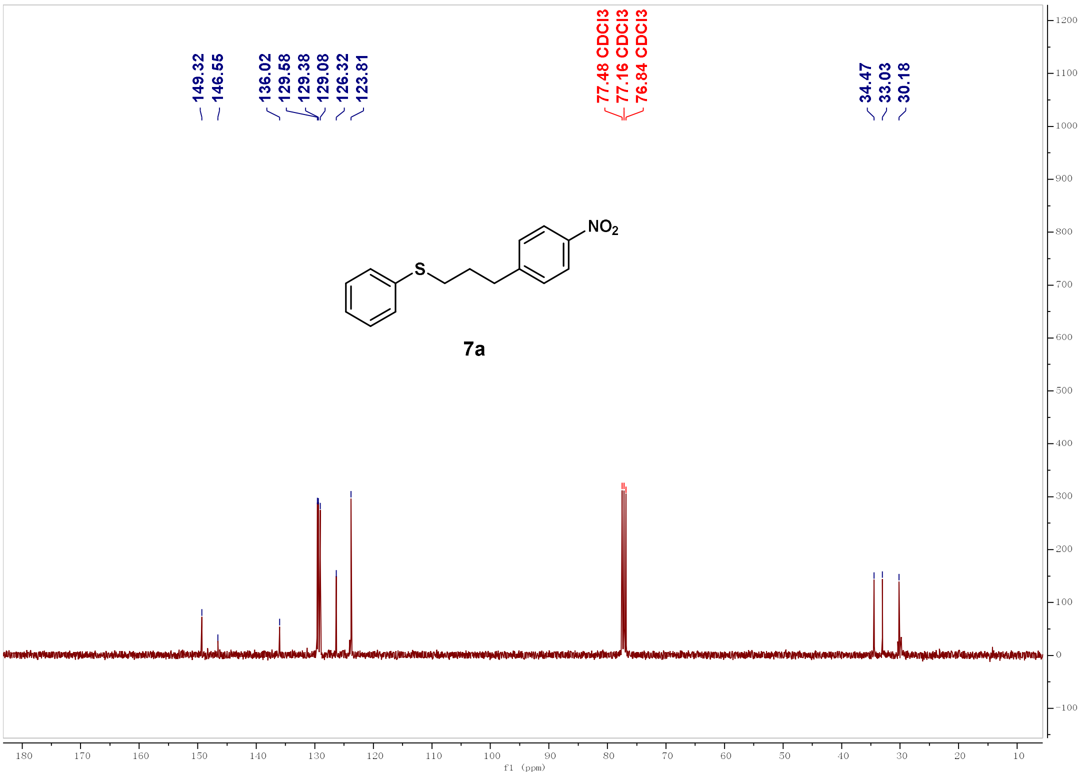

Supplementary Figure 125. ^{13}C NMR spectra for 7a

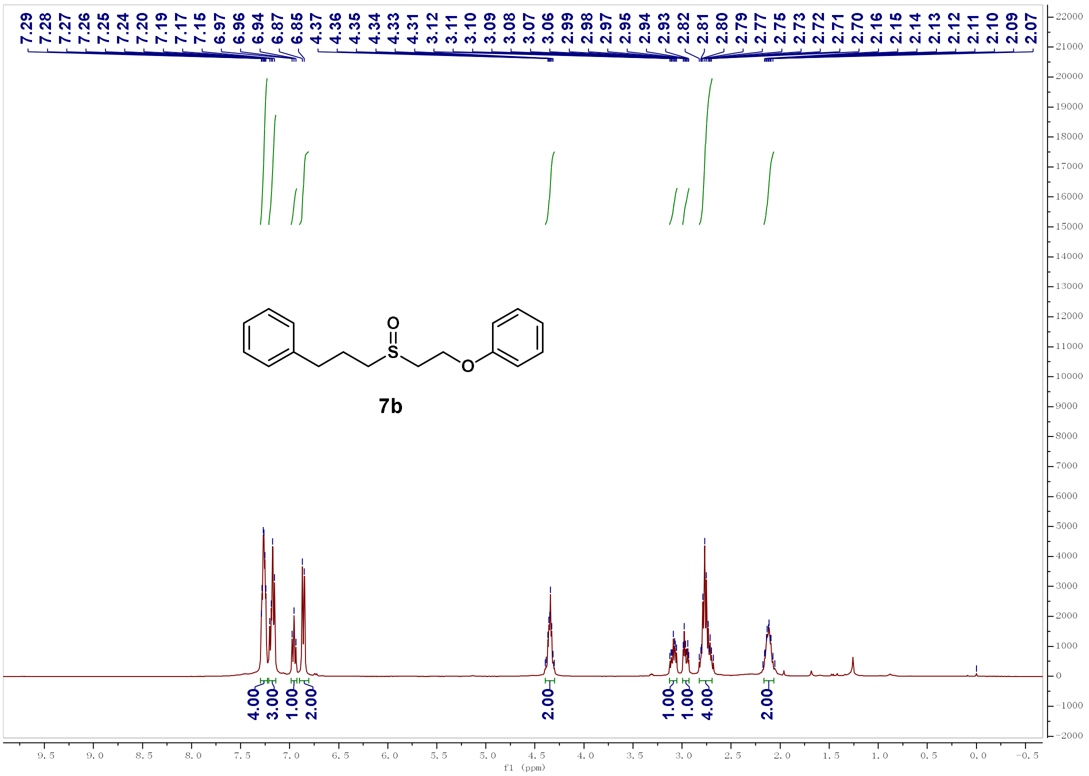

Supplementary Figure 126. ^{1}H NMR spectra for 7b

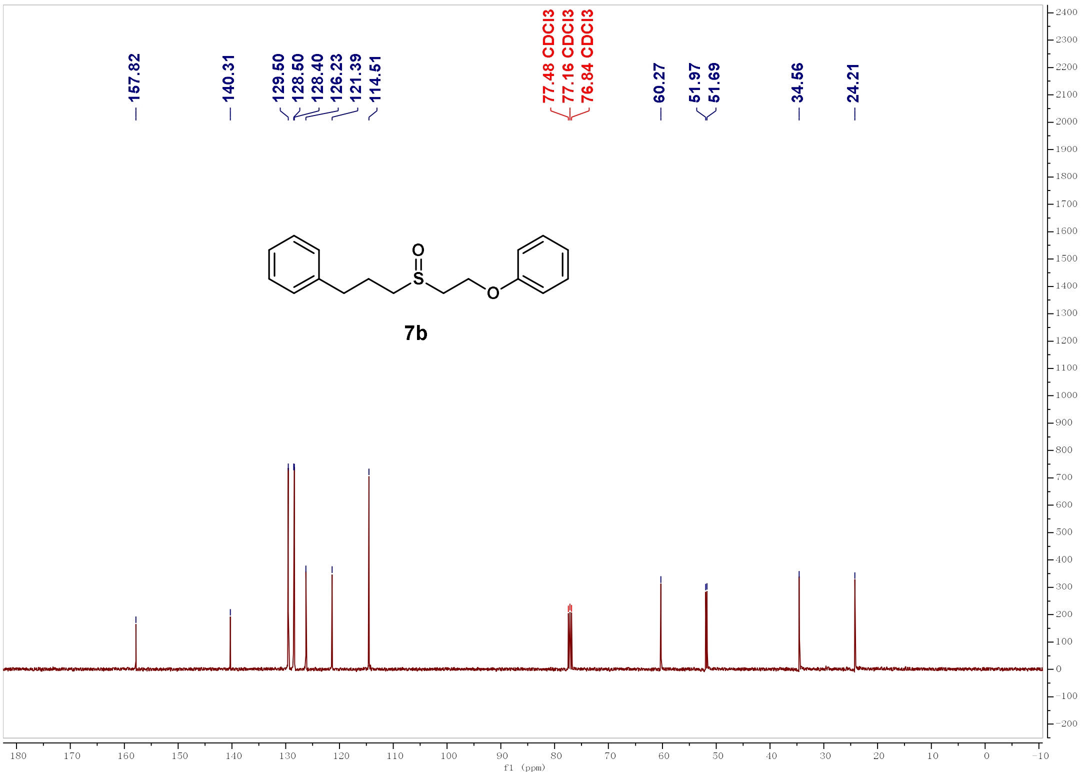

Supplementary Figure 127. ^{13}C NMR spectra for 7b

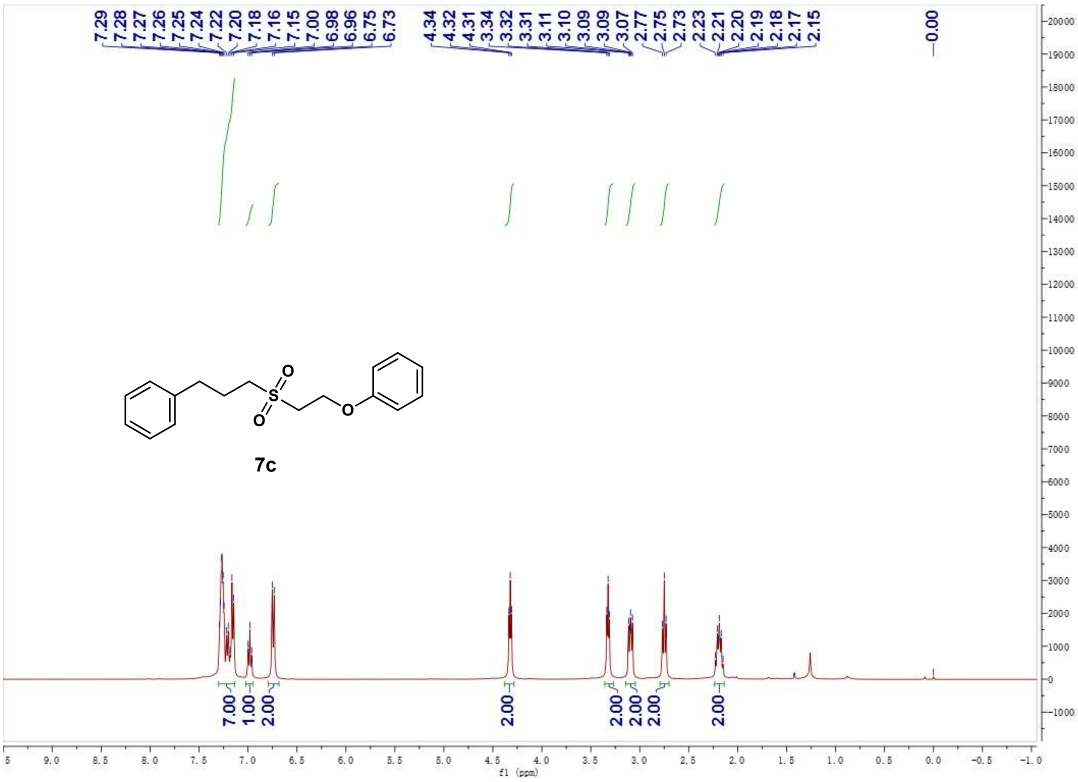

Supplementary Figure 128. ^{1}**H NMR spectra for 7c**

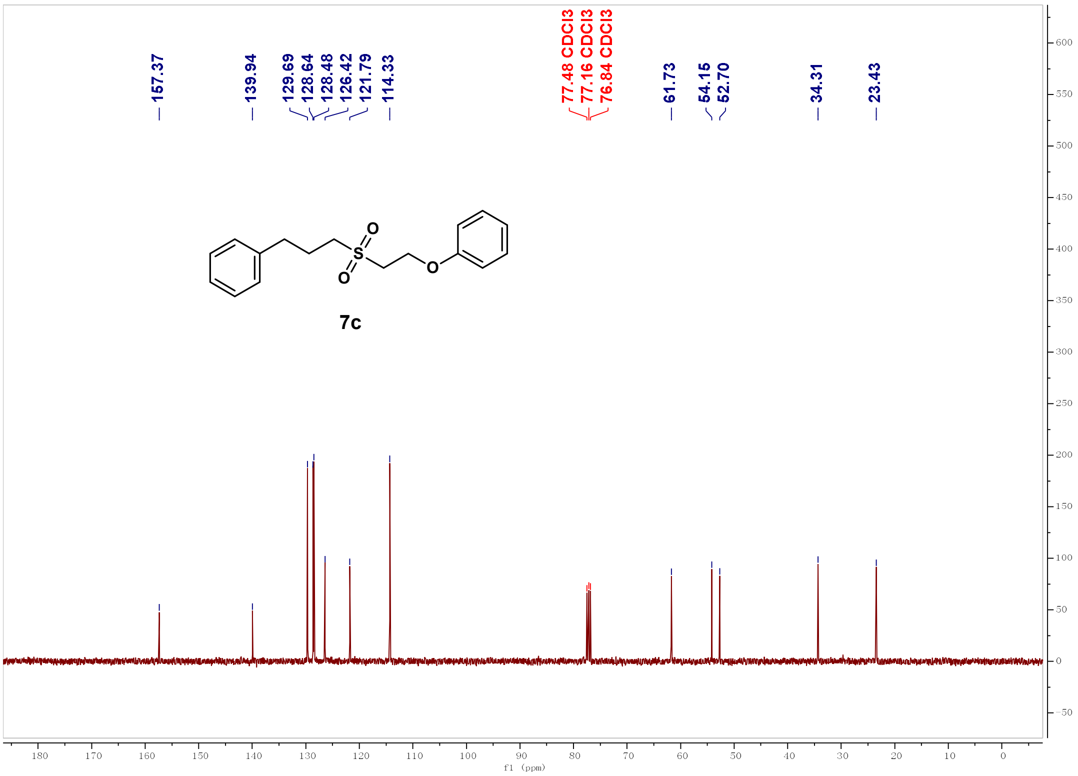

Supplementary Figure 129. ^{13}**C NMR spectra for 7c**

Supplementary References

1. Gamblin, D. P., Garnier, P., Ward, S. J., Oldham, N. J., Fairbanks A. J. & Davis, B. G. Glycosyl phenylthiosulfonates (Glyco-PTS): novel reagents for glycoprotein synthesis. *Org. Biomol. Chem.* **1**, 3642-3644 (2003).

2. Liang, G., Liu, M., Chen, J., Ding, J., Gao, W. & Wu, H. NBS-Promoted Sulfenylation of Sulfinates with Disulfides Leading to Unsymmetrical or Symmetrical Thiosulfonates. *Chin. J. Chem.* **30**, 1611-1616 (2012).

3. Stoll, A. H., Krasovskiy, A. & Knochel, P. Functionalized Benzylic Magnesium Reagents through a Sulfur–Magnesium Exchange. *Angew. Chem. Int. Ed.* **45**, 606-609 (2006).

4. Yang, C.-T., Zhang, Z.-Q., Tajuddin, H., Wu, C.-C., Liang, J., Liu, J.-H., Fu, Y., Czyzewska, M., Steel, P. G., Marder, T. B. & Liu, L. Alkylboronic Esters from Copper-Catalyzed Borylation of Primary and Secondary Alkyl Halides and Pseudohalides. *Angew. Chem. Int. Ed*. **51**, 528-532 (2012).

5. Bahrami, K., Khodaei, M. M., Yousefi, B. H., Arabi, M. S. TMSCl-promoted selective oxidation of sulfides to sulfoxides with hydrogen peroxide. *Tetrahedron Lett.* **51**, 6939-6941 (2010).

6. Espenson, J. H. *Chemical Kinetics and Reaction Mechanisms*, *2nd ed*., McGraw-Hill, New York, **1995**.
